# Rapid discovery of terpene tailoring enzymes for total biosynthesis†

**DOI:** 10.1039/d3sc04172g

**Published:** 2023-11-08

**Authors:** Yunlong Sun, Jennifer Gerke, Kevin Becker, Eric Kuhnert, Bart Verwaaijen, Daniel Wibberg, Jörn Kalinowski, Marc Stadler, Russell J. Cox

**Affiliations:** a Institute for Organic Chemistry, Leibniz Universität Hannover Germany russell.cox@oci.uni-hannover.de; b CeBiTec, University of Bielefeld Germany; c Helmholtz-Zentrum für Infektionsforschung Braunschweig Germany

## Abstract

Twenty oxygenated aristolochene congeners were rapidly synthesised by combining genes from four different fungal pathways in the fungal host organism *Aspergillus oryzae*. Compounds produced in a single step include the natural product hypoxylan A and an epimer of guignaderemophilane C. A new fungal aromatase was discovered that produces phenols by oxidative demethylation.

## Abstract 2

Twenty oxygenated aristolochene congeners were rapidly synthesised by combining genes from four different fungal pathways in the fungal host organism *Aspergillus oryzae*.

## Graphical abstract

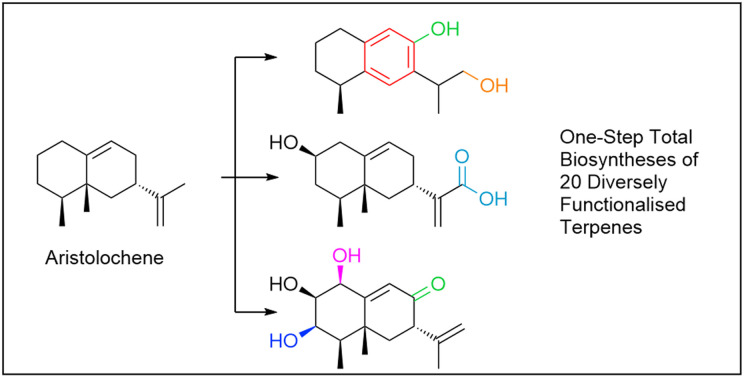

Advances in heterologous expression methodology in plants, fungi and bacteria mean that biosynthetic gene clusters (BGCs) encoding the biosynthesis of specialised metabolites can be rapidly assembled and expressed.^1^ In turn, many natural products can be produced by total biosynthesis. For example the modern antibacterial pleuromutilin has been produced in a single process by expression in the fungal host *Aspergillus oryzae*.^2^ Such processes compete very favourably with total chemical synthesis where the best route to pleuromutilin currently involves a minimum of 16 chemical steps and many reagents and solvents.^3^ However, total chemical synthesis is much more flexible than total biosynthesis because it can be used for the creation of many different structural variations. Natural biosynthetic processes normally involve the production of a specific skeleton, followed by tailoring processes. Current total biosynthesis strategies, such as that used during the total biosynthesis of pleuromutilin, follow the natural pathway exactly and rational structural variation is currently difficult.

Many biosynthetic pathways start from the same core carbon skeleton and then diverge by the function of different tailoring enzymes. It is likely that biosynthetic pathways evolve by the gain and loss of genes that encode these enzymes.^4^ A clear example of this comes from the four compounds sporogen AO-1 1a (*A. oryzae*^5^ and *A. flavus*^6^), hypoxylan A 2 (*Hypoxylon rickii*),^7^ eremoxylarin D 3 (*Xylaria hypoxylon*),^8^ and PR-toxin 4 (*Penicillium roquefortii*)^9^ that are all built from the aristolochene skeleton *via* different tailoring reactions. Sporogen AO-2 1b has been isolated from a plant source, but may be fungal in origin.^10^ Aristolochene 5 is produced in a single step by the terpene cyclase aristolochene synthase from farnesyl-diphosphate (FPP).^11^ Remarkably, even among these four aristolochene-derived compounds, twelve out of the fifteen carbons derived from FPP are oxidatively modified.

We hypothesised that it should be possible to mix-and-match tailoring enzymes from these related biosynthetic pathways to rapidly access differently oxidised aristolochenes. For this to be possible it would require some flexibility in substrate selectivity of the tailoring enzymes. Specifically, we hypothesised that pathways may be more web-like than exclusively linear (Scheme 1B). We rationalised that this could be likely since natural processes involving evolution by gain or loss of biosynthetic genes between related pathways also require flexibility in substrate selectivity. For example, we have shown that web-like routes are responsible for later oxidative tailoring steps during cytochalasan biosynthesis.^12–14^

**Scheme 1:**
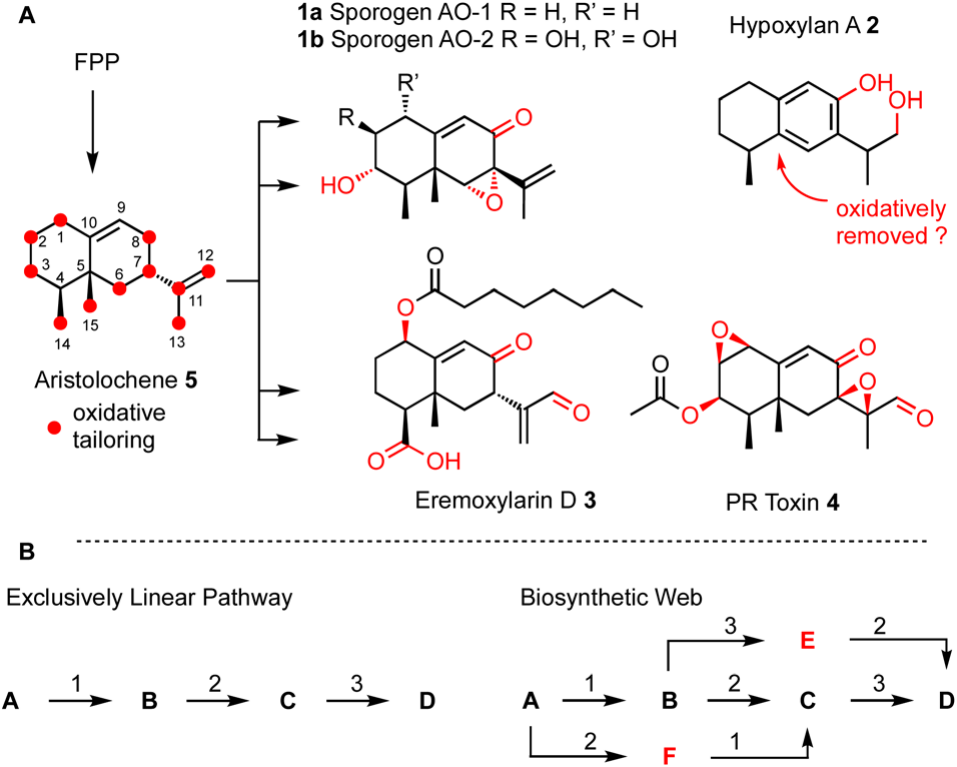
(A) Biosynthesis of aristolochene terpenoids in fungi; (B) comparison of linear and web-like biosynthetic routes.

We therefore set out to explore the catalytic potential of the oxidative tailoring enzymes of these metabolites using heterologous expression as our synthetic tool.

## Results

The fully-sequenced genomes of *A. oryzae* and *P. roquefortii* that produce sporogen AO-1 1a and PR-toxin 4 respectively are publicly available. We fully sequenced the genomes of *Hypoxylon rickii* and *Xylaria hypoxylon* that produce 2 and 3 respectively using combined Illumina and Oxford nanopore technologies.

**Fig. 1:**
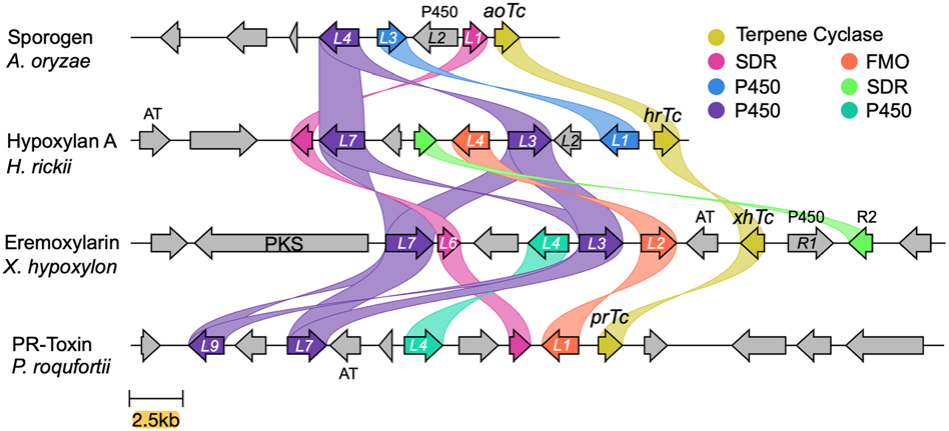
Clinker^15^ analysis of the four BGCs responsible for the biosynthesis of modified aristolochenes. PKS, polyketide synthase; AT, acyl transferase.

Assembled genomes were automatically annotated, and where necessary, manual annotation was performed to remove ambiguities using a previously described informatic pipeline.^15,16^ The biosynthetic gene cluster (BGC) encoding the biosynthesis of PR toxin 4 in *P. roquefortii* (*prL1*–*prL9*) is already known.^17^ Using this BGC we searched the genomes of the other three organisms using Cblaster.^18^ This revealed a single BGC in each organism likely to be responsible for the biosynthesis of each of the compounds. BGC-comparison was performed using CLinker (Fig. 1).^15^ This revealed that each BGC encodes the expected terpene cyclase, plus differing numbers of redox tailoring enzymes. General gene occurrence patterns supported our hypothesis that the BGCs do, in fact, encode the biosynthesis of each compound. For example, sporogen AO-1 1a is the simplest compound and its putative BGC (*aoTc*–*aoL4*) consists of the fewest genes. In the case of the PR-toxin BGC (*prR1*–*prL9*), the presence of an acetyl transferase corresponds with the presence of the 3-*O*-acetyl group. The putative eremoxylarin BGC (*xhR3*–*xhPKS*) encodes a polyketide synthase (PKS), most likely responsible for the range of polyketides esterified at *O*-1, while the proposed hypoxylan A 2 BGC (*hrTc*–*hrL8*) encodes an additional SDR (HrL5) that could be associated with its more reduced sidechain. All BGCs also encode transcription factors and transporters that show low sequence homologies, likely reflecting their different hosts and biological contexts.

All compounds 1–4 contain a ketone (or phenolic oxygen) at C-8. This could be introduced *via* initial oxygenation to form an allylic alcohol, followed by oxidation by a short-chain dehydrogenase-reductase (SDR). Consistent with this idea, all four BGCs encode a common P450 monooxygenase (AoL4, HrL7, PrL9 and XhL7) and a common SDR (AoL1, HrL8, PrL2 and XhL6) that may catalyse these steps.

*A. oryzae*, our standard expression host, is a domesticated derivative of *A. flavus* in which most specialized metabolite BGCs are inactive.^19^ This is convenient for heterologous expression experiments because newly introduced biosynthetic pathways do not have to compete for precursors with endogenous pathways, and newly created compounds are easier to detect against a clean metabolic background. Under our standard fermentation conditions *Aspergillus oryzae* NSAR1 does not produce sporogen AO-1 1a. This may be because the terpene cyclase is damaged or not expressed. However RT-PCR analysis showed that *aoL4* (P450) and *aoL1* (SDR) are transcriptionally active. We therefore expressed the *H. rickii* terpene cyclase gene (*hrTc*) under the control of the inducible *A. oryzae amyB* promoter (*P*_*amyB*_) in *A. oryzae* NSAR1.

**Fig. 2:**
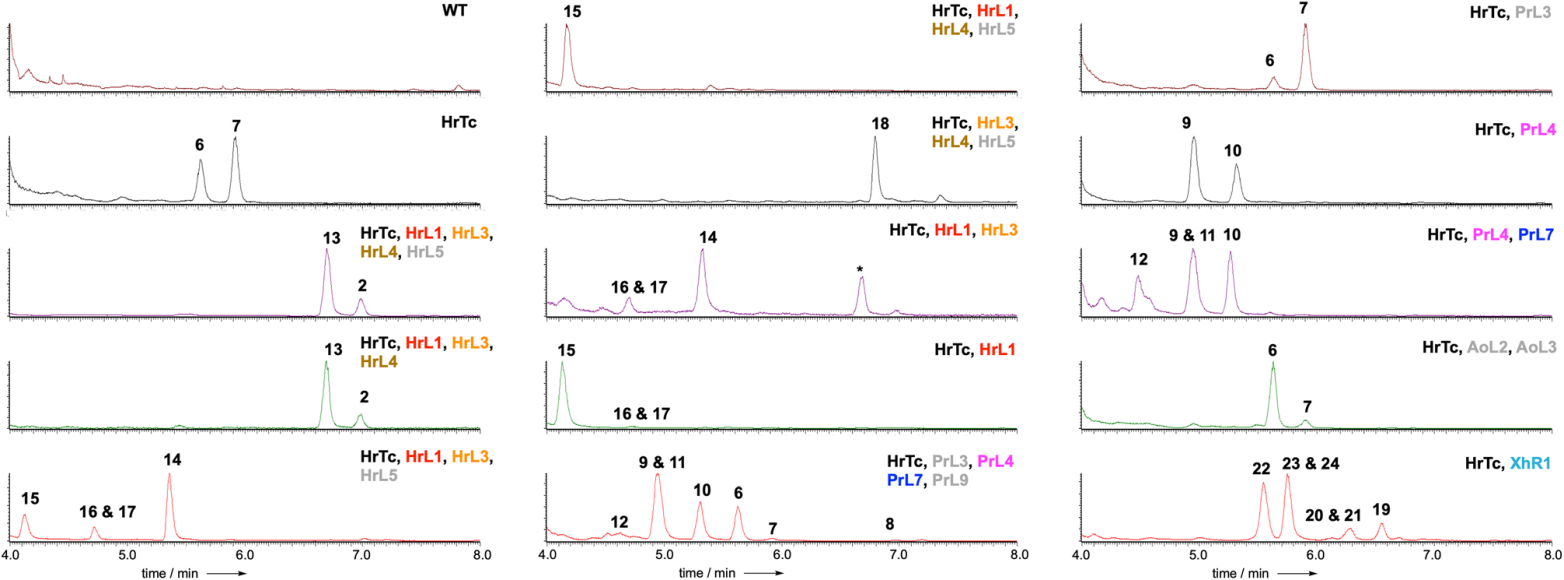
ELSD chromatograms obtained from extracts of *A. oryzae* NSAR1 expressing the indicated proteins. * = unrelated compound. Colour coding as Scheme 2. Grey proteins appear inactive (*e.g.* HrL5) or redundant (*e.g.* P450 PrL9 = P450 AoL4 that is already expressed in the host).

**Scheme 2:**
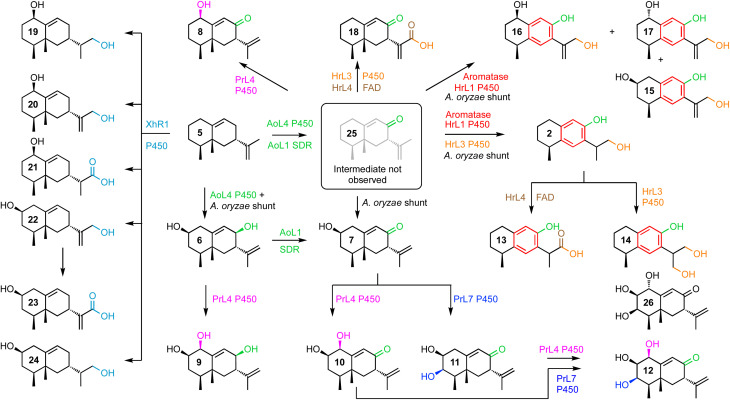
Compounds isolated and fully characterized after biosynthetic gene expression in *A. oryzae* showing expressed proteins and putative shunt steps.

LCMS analysis of transformants grown under inducing conditions (Fig. 2) showed the production of two new compounds that were purified, fully characterised by NMR, and shown to be 2,8-dihydroxy aristolochene 6 (5.0 mg L^−1^)^20^ and 2-hydroxy, 8-oxo-aristolochene 7 (7.2 mg L^−1^, Scheme 2). In all cases the absolute stereochemistry was assumed to match 5 itself, and relative stereochemistry was determined *via J* and nOe correlations.

PR-toxin 4 from *Penicillium roquefortii*, (*pr*) is oxidised at C-1 and C-3. Coexpression of *hrtc* and *prL4* resulted in production of the 1-hydroxylated congeners 8 (4.2 mg L^−1^), 9 (6.0 mg L^−1^) and 10 (4.7 mg L^−1^). Further addition of *prL7* to this system provided the 3-hydroxylated congeners 11 (6.0 mg L^−1^) and 12 (3.5 mg L^−1^). In contrast, hypoxylan A 2 from *Hypoxylon rickii* (*hr*) is not oxidised at positions 1-3, but features a phenolic ring and loss of C-15. In order to investigate the reactions leading to this skeleton, we coexpressed all of the biosynthetic genes from the *hr* BGC. This provided 2 itself (8.5 mg L^−1^), plus the corresponding carboxylic acid 13 (15.0 mg L^−1^). Omission of the FAD-dependent dehydrogenase encoded by *hrL4* led to the diol 14 (4.0 mg L^−1^) and no more highly oxidised analogs. Compound 15 (5.0 mg L^−1^), in which the phenol is present, was formed by coexpression of *hrTc* and *hrL1* alone. This was accompanied by C1-alcohol epimers 16 and 17 (3.0 mg L^−1^ combined). Coexpression of *hrTc* with *hrL3* and *hrL4* created the carboxylic acid 18 (5.6 mg L^−1^).

Finally, coexpression of *xhR1* with *hrTc* provided six new aristolochene congeners 19 (4.0 mg L^−1^), 20 + 21 (3.0 mg L^−1^), 22 (4.0 mg L^−1^) and 23 + 24 (10.0 mg L^−1^) respectively. These are predominantly oxidised at C-13, but unoxidised at C-8, suggesting that early oxidation at C-13 may prevent the *A. oryzae* C-8 oxidases from recognising these as substrates.

## Discussion

The strategy of mining four BGCs that all process the same terpene skeleton for functional tools for future total biosynthesis projects proved highly effective. The host organism *A. oryzae* itself apparently already expresses catalysts (AoL1 and AoL4) that form the 8-hydroxyl and 8-oxo functionalities.

This is in common with the other three BGCs that must all also form the 8-ketone 25 in their respective biosynthetic pathways, as early common steps. Compound 25 (Scheme 2) has already been shown by Dickschat and coworkers to be an intermediate in the pathway to PR-toxin 4.^14^ However, this compound is reported to be highly unstable, and we did not directly observe 25 in our experiments.

Oxidative enzymes from the PR-toxin (*pr*) BGC can hydroxylate at the 1- and 3-positions, while enzymes from the *H. rickii* (*hr*) hypoxylan A 2 pathway catalyse redox processes on the isopropenyl side-chain carbons 11-13.

**Scheme 3:**
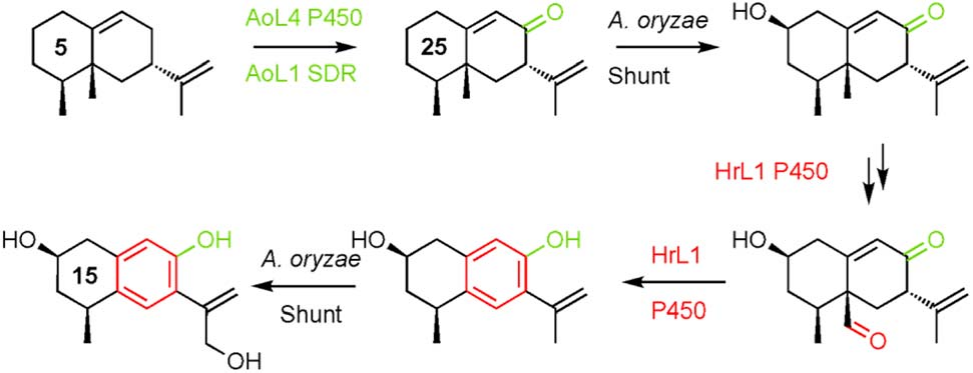
Combination of *A. oryzae* sporogen and shunt enzymes and *H. rickii* aromatase activity to reach 15.

Remarkably, cytochrome P450 monooxygenase HrL1 behaves as an aromatase (Scheme 3), catalysing the oxidative elimination of the C-15 methyl to create the distinctive phenolic nucleus of hypoxylan A 2. Aromatase is an important enzyme involved in the biosynthesis of estrogens in vertebrates, but it is not known in other organisms, and to the best of our knowledge this is the first fungal example.

These experiments show that achieving total synthesis using the easily accessible genetic resources of fungi is an attractive prospect. Here we report the first total synthesis of hypoxylan A 2 in a single process. More complex compounds were also produced. For example, triol 12 is a C-1 epimer of the known fungal natural product guignaderemophilane C 26 (ref. 21) that has been accessed by total chemical synthesis in eleven steps.^22^ In this case, combination of genes from *H. rickii*, *P. roquefortii* and the host organism *A. oryzae* allowed the synthesis of the epimer 12 in a single process.

*A. oryzae* is a widely-used host organism for pathway expression and engineering.^23^ Normally it is regarded as a clean host that does not interfere with engineered pathways. However in this project *A. oryzae* becomes a rather active host. It is clear that AoL4 (P450) and AoL1 (SDR) are active. This is beneficial since the 8-oxo functionality is required by all pathways to 1–4. However, observation of 2-hydroxylation was not anticipated and many of the observed products feature this substitution. Other shunts are exemplified by the putative route to 15 that involves unplanned hydroxylation at C-2 and C-13 in addition to the aromatase chemistry catalysed by HrL1.

Although it is currently impossible to predict the substrate selectivity or mode of reaction of any given tailoring enzyme from its sequence alone, such predictability will be required if total biosynthesis is to approach the flexibility of total chemical synthesis. However, in the absence of such predictions, our results show that harvesting and screening tailoring genes from pathways that utilise the same carbon skeleton is an effective way of finding catalysts for use in total biosynthesis. Twenty compounds were derived from manipulation of only 6 genes. It is likely that many other compounds could be synthesised by the same methodology, since only a fraction of available genes and combinations were coexpressed. Given that aristolochene-type terpenes are among the most common in fungi^24^ it is also likely that very many other BGCs could be used to expand the diversity yet further. The same strategy should also be effective for other classes of specialized metabolites, and as more BGCs are discovered and linked to particular compounds it will become increasingly easy to design and build effective total biosynthetic routes to target compounds. It is also likely that tailoring enzymes with inherently flexible substrate selectivities obtained from biosynthetic webs could be engineered by directed evolution^25^ more easily than enzymes with high selectivies from exclusively linear pathways. Such engineered enzymes already find important roles in shortening total chemical synthesis routes to natural products.^26^ Finally, these results lend significant support to ideas that gain and loss of biosynthetic genes contributes to fungal specialized metabolite diversity and evolution.

## Data availability

Annotated genome sequence files are available at: https://doi.org/10.6084/m9.figshare.23683644.v1 (*Xylaria hypoxylon*); and https://doi.org/10.6084/m9.figshare.23652825.v1 (*Hypoxylon rickii*).

## Author contributions

YS and KB did all experimental work. JG, EK, BV, DW and JK sequenced and annotated genomes and provided data analysis. MS provided organisms and RJC, EK and MS obtained funds. RJC drafted the MS and led the project. All authors were involved in polishing the draft MS.

## Conflicts of interest

There are no conflicts to declare.

## Supplementary Material

SC-014-D3SC04172G-s001
