# Supplementary material for: Rapid discovery of terpene tailoring enzymes for total biosynthesis
Source: Chem Sci. 2023 Nov 8;14(46):13463–7. doi: 10.1039/d3sc04172g (PMC10686045; doi:10.1039/d3sc04172g)
Supplement: SC-014-D3SC04172G-s001 [file SC-014-D3SC04172G-s001.pdf]

Electronic Supplementary Material (ESI) for Chemical Science.
This journal is © The Royal Society of Chemistry 2023

Rapid Discovery of Terpene Tailoring Enzymes for Total Biosynthesis

Yunlong Sun,[a] Jennifer Gerke,[a] Kevin Becker,[a] Eric Kuhnert,[a] Bart Verwaaijen,[b] Daniel Wibberg,[b] Jörn Kalinowski,[b] Marc Stadler[c] and Russell J. Cox*[a]

Electronic Supplementary Information

a. Institute for Organic Chemistry, Leibniz Universität Hannover.

b. CeBitec, University of Bielefeld.

c. Helmholtz-Zentrum für Infektionsforschung, Braunschweig.

Contents

1. Cloning Procedures **3**

1.1 Genome Sequences and Annotation 3

1.2 Oligonucleotides and General PCR 3

1.3 RT-PCR for AoL4 and AoL1 3

1.4 Preparation of Competent Yeast 4

1.5 Yeast Recombination 4

1.6 Plasmid Selection and Generation by *E. coli* 5

2. Constructed Vectors **12**

3. Transformation and Selection of *A. oryzae* **14**

3.1 Preparation of Protoplasts 14

3.2 Transformation and Selection 14

4. Fermentation, Analysis and Compound Purification **16**

4.1 Fermentation and Extraction Protocols 16

4.2 Analytical and Preparative LC-MS 16

4.3 HRMS and NMR 16

5. Compound Characterization **19**

Compound 2 20

Compound 5 25

Compound 6 26

Compound 7 31

Compound 8 36

Compound 9 41

Compound 10 46

Compound 11 51

Compound 12 56

Compound 13 61

Compound 14 66

Compound 15 71

Compound 16, 17 76

Compound 18 82

Compound 19 87

Compound 20, 21 92

Compound 22 100

Compound 23, 24 105

6. References **113**

1. Cloning Procedures

1.1 Genome Sequences and Annotation

De novo gene predictions and annotations were made with the Funnanotate package v1.8.9 (Jonathan M. Palmer, https://github.com/nextgenusfs/funannotate) using the following database versions; MEROPS v12.0, Uniprot release 2022_01, dbCan 10.0, pfam v35.0, GO release 2022-01-13, MIBiG v1.4, Interpro 87.0, BUSCO outgroups v1.0, gene2product v1.75, Eggnog_DB v5.0.2.

Annotated genome sequence files are available at:

1. *Xylaria hypoxylon*: https://doi.org/10.6084/m9.figshare.23683644.v1
2. *Hypoxylon rickii*: https://doi.org/10.6084/m9.figshare.23652825.v1

1.2 Oligonucleotides and General PCR

Oligonucleotides for PCR and RT-PCR were designed using Geneious and synthesized by SigmaGenosys and Eurofins. The PCR experiments were conducted using Q5® high-fidelity DNA polymerase (New England Biolabs) for the DNA fragment for heterologous expression, and OneTaq® DNA polymerase was used for colony PCR. The gDNA from candidate fungi was extracted using a GeneEluteTM Plant Genomic DNA Miniprep Kit (Sigma Life Science). The protocol of gDNA extraction was described in our previous literature. [1] The hypoxylan A BGC DNA fragments were cloned from cDNA of *Hypoxylon rickii* without introns. The DNA fragments of the PR-toxin BGC were directly cloned from the gDNA of *Penicillium roquefortii* with introns. The DNA fragments of the sporogen AO BGC were cloned from the gDNA of *A. oryzae* NSAR1. The exons DNA fragments of *xhr1* were cloned from the *Xylaria hypoxylon* gDNA and then connected to a CDS sequence.

1.3 RT-PCR for AoL4 and AoL1

The mycelia of *A. oryzae* NSAR1 and *A. oryzae* NSAR1 transformants containing HrTc were collected after being cultured in DPY medium for 5 days, then quickly frozen in liquid nitrogen and ground into powder. The RNA was extracted using Quick-RNA Fungal/Bacterial Microprep Kit (ZYMO), and the High Capacity RNA-to-cDNA™ Kit was used to reverse RNA to cDNA. The protocol of RNA extraction and RNA reserve experiment was described in our previous literature. [2] The resulting cDNA was used as the templates for the amplification of *aol4* and *aol1* by 450-500 bp (Figure S1).

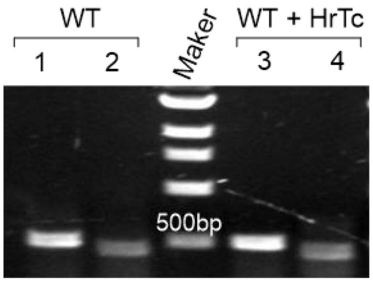

1. The band of AoL4 in WT
2. The band of AoL1 in WT
3. The band of AoL4 in WT+HrTc
4. The band of AoL1 in WT+HrTc

Figure S1. RT-PCR for the expression level of AoL4 and AoL1. Primers RT-PCR-*aoL4*-F and RT-PCR-*aoL4*-R were used for AoL4; RT-PCR-*aoL1*-F and RT-PCR-*aol1*-R were used for AoL1

1.4 Preparation of Competent Yeast

All subsequent experiments utilised four vectors (pTYGs) that had been manipulated with different selection markers (△*argB*, *sC*$^-$, *adeA*$^-$, *niaD*$^-$) for selection in *A. oryzae NSAR1*. The pTYGs vectors are equipped with the *2μ* origin and the *colE1* gene which facilitate replication in *Saccharomyces cerevisiae* and *E. coli* respectively. The auxotrophy *URA3* gene and *carB* resistance gene were responsible for selection in *Saccharomyces cerevisiae* and *E. coli* respectively. The *ccdB* suicide gene was used as also a selection marker in *E. coli*. Every pTYGs vector includes four promoter and terminator combinations (P/T*amyB*, P/T*adh*, P/T*gpdA*, and P/T*eno*). All four empty plasmids can be restricted by *Asc*I between P/T*adh*, P/T*gpdA*, and P/T*eno* respectively, and P/T*amyB* could be restricted by *Not*I for yeast recombination.

Yeast was cultivated on YPAD agar at 30 °C for three days. A single colony was selected and incubated overnight in 10 mL YPAD media at 30 °C, 200 rpm. The 10 mL YPAD media was transferred to a 250 mL Erlenmeyer flask containing 40 mL fresh YPAD medium and incubated at 30 °C with 200rpm shaking for additional 4 hours. To collect the cells, the medium was centrifuged at 3,000 g for five minutes. The pellet was rinsed with 25 mL of ddH_2O and centrifuged, then this step was repeated. The pellet was suspended in a Falcon tube containing a total of 5ml Lithium acetate (LiOAc, 0.1 M) and each 50 µL was transferred into a separate 1.5 mL Eppendorf reaction tube. For immediate use, each sample was pelleted at 21,000 g for 15 s and the pellet was used for the yeast transformation. For cell stocking, instead of using LiOAc in the initial stage, FCC solution was used to stock frozen competent yeast cells. The pellet was suspended in 5 mL of FCC solution before being divided into 50 µL portions and placed into individual 1.5 mL Eppendorf reaction tubes. Samples were stored at -80 °C. For thawing, samples were first incubated on ice and then centrifuged for 15 s at 21,000 g. The cells were employed for the yeast transformation process after the FCC solution was withdrawn.

1.5 Yeast Recombination

The following components were added to the pellet in order:
1. 50 µL ssDNA;
2. 36 µL 1 M LiOAc;
3. 34 µL DNA mix with linearized plasmid and appropriate inserts;
4. 240 µL PEG solution;

The uncut plasmid served as a positive control, whereas the linearized plasmid served as a negative control. The particulate was suspended in the transformation mixture and incubated at 30 °C for 30 minutes with shaking by 300 rpm, followed by 42 °C for 40 minutes. The cells were pelleted at 13,000 g for 60s to get the pellet, and the supernatant was removed. The pellet was suspended in 200 µL ddH_2O and distributed on selective SM-Ura plates, which were then incubated at 30 °C for three days. The extraction of yeast plasmid was performed utilising the ZymoprepTM Yeast Plasmid Miniprep II kit (Zymo Research, Orange, California, USA).

Media and solution

SM-URA Agar

0.17 % (w/v) Yeast nitrogen base (Sigma Aldrich); 0.50 % (w/v) Ammonium sulfate (Roth); 2.00 % (w/v) D(+)-Glucose Monohydrate (Roth); 0.077 % (w/v) Complete supplement mixture minus Uracil (Sigma Aldrich); 1.50 % (w/v) Agar (Duchefa Biochemie)

YPAD Agar/medium

1.00 % (w/v) Yeast extract (Duchefa Biochemie); 2.00 % (w/v) Tryptone (Duchefa Biochemie); 2.00 % (w/v) D (+)-Glucose Monohydrate (Roth); 0.03 % (w/v) Adenine (Roth); 1.50 % (w/v) Agar (Duchefa Biochemie)

FCC solution: 5% (v/v) glycerol; 10% (v/v) DMSO in ddH_2O
PEG solution: 50% (w/v) polyethylene glycol 3350 in ddH_2O
ssDNA: 2 mg/mL salmon sperm DNA in TE buffer, denatured at 98 °C for 5 minutes

1.6 Plasmid Selection and Generation by *E. coli*

After the total plasmids extraction of yeast, all the plasmids were transferred to *E. coli* competent cells. 50 μL *E. coli* competent cells (Top10 or *ccdB* Survival TM 2 T1R, Thermo Fisher Scientific, USA) was used to mix with the yeast total plasmids and incubated on ice for up to 25 min. Following a heat shock of 90 seconds at 42 °C, the cells were subsequently transferred on ice for 3 minutes. Subsequently, 500 µL of SOC medium was introduced to the cells. The cells were subjected to incubation at 37 °C, 200 rpm for 1 hour. Subsequently, they were evenly distributed onto LB agar plates that were supplemented with antibiotics. The plates were then left to incubate overnight at 37 °C. 14 colonies of each plasmid were chosen and suspended in 14 tubes containing 10 µL ddH_2O as a template for colony PCR. Different sets of primers were used to detect all of the genes in each plasmid. Three positive colonies of each plasmid were selected and grown overnight in a 50 mL LB medium containing antibiotics. The *E. coli* cells were harvested by centrifuge. A NucleoSpin Plasmid Kit (MACHEREY-NAGEL) was used to get the pure plasmid. A DNA sequencing kit (Eurofins Genomics) was used to confirm the sequences of all plasmids.

Media and antibiotics

LB Agar/medium

0.50 % (w/v) Yeast extract (Duchefa Biochemie); 1.00 % (w/v) Tryptone (Duchefa Biochemie); 0.50 % (w/v) Sodium chloride (Roth or VWR); 1.50 % (w/v) Agar (Duchefa Biochemie)

SOC medium

0.50 % (w/v) Yeast extract (Duchefa Biochemie); 2.00 % (w/v) Tryptone (Duchefa Biochemie); 0.06 % (w/v) Sodium chloride (Roth or VWR); 0.02 % (w/v) Potassium chloride (Roth); To be added: 25 mM final concentration Magnesium chloride hexahydrate 2M (Roth); 1.0 % final concentration D(+)-Glucose Monohydrate 20 % (Roth)

Carbenicillin

Stock concentration is 50 $mg\cdot mL^{-1}$ in ddH_2O, working concentration is 50 $\mu g\cdot mL^{-1}$;

Chloramphenicol

Stock concentration is 30 $mg\cdot mL^{-1}$ in ethanol, working concentration is 30 $\mu g\cdot mL^{-1}$

Table S1 Oligonucleotide sequences

Primer	Sequence (5'- 3')	Purpose
Pamy-hrtc-F	CTGAACAATAAACCCCACAGCAAGCTCCGAATGGCGCCAATGGTCGACGA	HrTc forward
Pamy-hrtc-R	ACTCTCCACCCTTCACGAGCTACTACAGATTCAGGTGGCCGGCTGCACAT	HrTc reverse
Padh-hrl4-F	TTCTTTCAACACAAGATCCCAAAGTCAAAGATGCTCTCCTACATGCTCCC	HrL4 forward
Padh-hrl4-R	TTTCATTCTATGCGTTATGAACATGTTCCCTTAATTCAATGGAAACCCTC	HrL4 reverse
Pgpd-hrl5-F	ACAGCTACCCCGCTTGAGCAGACATCACCGATGGTTAAGTTGGACTTCGA	HrL5 forward
Pgpd-hrl5-R	TACGACAATGTCCATATCATCAATCATGACCTACGTCAACCAGGGCTTGA	HrL5 reverse
Peno-hrl8-F	CGACTGACCAATTCCGCAGCTCGTCAAAGGATGGCATCACTCAGGATTGA	HrL8 forward
Peno-hrl8-R	CAGGTTGGCTGGTAGACGTCATATAATCATTTAGCGGAAGTCTGTCGCCG	HrL8 reverse
Pamy-hrl3-F	CTGAACAATAAACCCCACAGCAAGCTCCGAATGGAGGAAGGCTTGATTGC	HrL3 forward
Pamy-hrl3-R	ACTCTCCACCCTTCACGAGCTACTACAGATTCAGGACGAGAGCGCATCTC	HrL3 reverse
Padh-hrl1-F	TTCTTTCAACACAAGATCCCAAAGTCAAAGATGTCGTCGACTCCCGTGCT	HrL1 forward
Padh-hrl1-R	TTTCATTCTATGCGTTATGAACATGTTCCCTTAGACTGCCGCTTTCCGCC	HrL1 reverse
Pgpd-hrl7-F	ACAGCTACCCCGCTTGAGCAGACATCACCGATGTTCGGAACAGATGCTCT	HrL7 forward
Pgpd-hrl7-R	TACGACAATGTCCATATCATCAATCATGACTTAGTGCTCTACCAACTTTG	HrL7 reverse
Padh-prl3-F	TTCTTTCAACACAAGATCCCAAAGTCAAAGATGGCCGTGATCTTCTCCTC	PrL3 forward
Padh-prl3-R	TTTCATTCTATGCGTTATGAACATGTTCCCTCAGGCCTTGAGACGTCTTT	PrL3 reverse
Pgpd-prl4-F	ACAGCTACCCCGCTTGAGCAGACATCACCGATGAACGCCTCAAAGTTGCC	PrL4 forward
Pgpd-prl4-R	TACGACAATGTCCATATCATCAATCATGACCTAGCCAGTCTTGACGTCTG	PrL4 reverse
Pgpd-prl7-F	ACAGCTACCCCGCTTGAGCAGACATCACCGATGACCATATCTCCAATCCC	PrL7 forward
Pgpd-prl7-R	TACGACAATGTCCATATCATCAATCATGACCTATGCCTTGAGTGTGCTAC	PrL7 reverse
Peno-prl9-F	CGACTGACCAATTCCGCAGCTCGTCAAAGGATGGATGCACTCGAGGCTCC	PrL9 forward
Peno-prl9-R	CAGGTTGGCTGGTAGACGTCATATAATCATCTAATCGACTTCTGTGTTTA	PrL9 reverse
Padh-aol3-F	TTTCTTTCAACACAAGATCCCAAAGTCAAAATGTTTGAGGGCGCCTACAC	AoL3 forward
Padh-aol3-R	TTCATTCTATGCGTTATGAACATGTTCCCTCTATGTGCACCTTCTCCGTT	AoL3 reverse
Pgpd-aol2-F	TAACAGCTACCCCGCTTGAGCAGACATCACATGACTCTAATATCGCTGTC	AoL2 forward
Pgpd-aol2-R	ACGACAATGTCCATATCATCAATCATGACCCTACTGCGCACTTAATCTCA	AoL2 reverse
RT-PCR-aol4-F	TGTTTTCAATACAATGGAACGATTC	RT-PCR for AoL4
RT-PCR-aol4-R	GGCGAATGTGGCATACTTCCACTGG	RT-PCR for AoL4
RT-PCR-aol1-F	AGGAGGAAGCTCAGGGATTGGCTGG	RT-PCR for AoL1
RT-PCR-aol1-R	GCCTAATTTCGTTGCACTATAGACC	RT-PCR for AoL1
Padh-2XhR1-F1	TTTCTTTCAACACAAGATCCCAAAGTCAAAATGACAGCCAAAATGTTCGA	CDS part1 forward
Padh-2XhR1-R1	TAGAGTCTATCAACAAACGA	CDS part1 reverse
Padh-2XhR1-F2	TGCAAGAAAGTCGTTTGTTGATAGACTCTATCGTCGGTTTGGTGATACGC	CDS part2 forward
Padh-2XhR1-R2	GCATAGCACTCTTGGGCATT	CDS part2 reverse
Padh-2XhR1-F3	AGATGTTTGGAATGCCCAAGAGTGCTATGCACATATATCGCAACGACAAG	CDS part3 forward
Padh-2XhR1-R3	TCGCGTTGCCGTTCTATAGTGATGGTGCCACACGCGGAGATGAGCTGGTA	CDS part3 reverse
Padh-2XhR1-F4	TGGCACCATCACTATAGAAC	CDS part4 forward
Padh-2XhR1-R4	GCTGTAAGAACGAACAGATC	CDS part4 reverse
Padh-2XhR1-F5	TGCAAGCGAGGATCTGTTCGTTCTTACAGCTGCAACCGCAAATGCTATGC	CDS part5 forward
Padh-2XhR1-R5	AGGTTGGCTGGTAGACGTCATATAATCATACTAAGAATGCTTTACAGTGG	CDS part5 reverse
PamyB_S-F	CATGCTTGGAGGATAGCAACCG	For sequencing, located in PamyB
PamyB_S-R	ACTCCAACTGTACATCAAACTCA	
Padh plugF	ATTCACCACTATTATTCCCACCCTATAATA	For PCR fragments to close the *AscI* cloning site without targeting DNA and also for sequencing
Padh plugR	GAGACGAAACAGACTTTTTCATCGCTAAAA	
PgdpA plugF	CTTTTCTTTTCTCTTTCTTTTCCCATCTTC	
PgpdA plugR	TACGACAATGTCCATATCATCAATCATGAC	
Peno plugF	CTTCTTAAATATCGTTGTAACTGTTCCTGA	
Peno plugR	CGAAGTATATTGGGAGACTATAGCTACTAG	

Table S2 Gene annotations of PR-toxin BGC

Locus tag	Label	Predicted protein function	Domain hits
S02g001477	PrL10	Oxidoreductase BOA17	17beta hydroxysteroid dehydrogenase-like
S02g001475	PrL9	Cytochrome P450 monooxygenase ORF11	CYP60B-like
S02g001474	PrL8	Transcription factor ORF10	GAL4-like Zn_2Cys_6 binuclear cluster DNA-binding domain
S02g001473	PrL7	Cytochrome P450 monooxygenase ORF9	CYP60B-like
S02g001472	PrL6	Eremophilane O-acetyltransferase ORF8	Transferase
S02g001470	PrL5	Hypothetical protein	DUF3237 domain-containing protein with a beta-barrel structure
S02g001469	PrL4	Cytochrome P450 monooxygenase ORF6	CYP56-like
S02g001468	PrL3	Cytochrome P450 monooxygenase ORF5	CYP56-like
S02g001467	PrL2	Short-chain dehydrogenase/reductase prx4	Insect-type alcohol dehydrogenase (ADH)-like, classical (c) SDRs
S02g001466	PrL1	FAD-dependent monooxygenase Prx3	FAD/FMN-containing dehydrogenase
S02g001465	PrTc	Aristolochene synthase	Non-plant Terpene Cyclases, Class 1
S02g001464	PrR1	Short-chain dehydrogenase/reductase Prx1	Retinol-DH_like_SDR_c_like
S04g000591	PrR2	Short-chain dehydrogenase/reductase Prx7	Insect-type alcohol dehydrogenase (ADH)-like, classical (c) SDRs
S04g000592	PrR3	Short-chain dehydrogenase/reductase Prx6	Insect-type alcohol dehydrogenase (ADH)-like, classical (c) SDRs
S04g000593	PrR4	MFS-type transporter Prx5	Fungal trichothecene efflux pump (TRI12) of the Major Facilitator Superfamily of transporters
S04g000594	PrR5	Up-frameshift suppressor 2 homolog	Middle domain of eukaryotic initiation factor 4G, Up-frameshift suppressor 2
S04g000595	PrR6	Unnamed protein product	/
S04g000595	PrR7	Plasmid maintenance protein 2	Forkhead-associated (FHA) domain found in *Saccharomyces cerevisiae* plasmid maintenance protein

Table S3 Gene annotations of sporogen AO1 BGC

Locus tag	Label	Predicted protein function	Domain hits
AO090011000096	AoL7	Transcription factor	Fungal_TF_MHR
AO090011000097	AoL6	Drug resistance transporter	MFS_Azr1_MDR_like; efflux_EmrB
AO090011000098	AoL5	Unnamed protein product	/
AO090011000099	AoL4	Cytochrome P450 monooxygenase ORF9	CYP60B-like; CypX; p450
AO090011000100	AoL3	Cytochrome P450 monooxygenase BraC	CYP60B-like; CypX; p450
AO090011000101	AoL2	Cytochrome P450 monooxygenase TpcC	CYP60B-like; CypX; p450
AO090011000102	AoL1	Short-chain dehydrogenase Prx4	FabG; ADH_SDR_c_like; adh_short
AO090011000103	AoTc	Aristolochene synthase	Terpene_cyclase_nonplant_C1; Terpene_syn_C_2

Table S4 Gene annotations of hypoxylan A BGC

Locus_tag	Label	Predicted protein function	Domain hits
Hr2g6175	HrL10	Trichothecene 8-O-acetyltransferase	Transferase
Hr2g6176	HrL9	Unnamed protein product	/
Hr2g6177a	HrL8	Short-chain dehydrogenase Prx4;	FabG; ADH_SDR_c_like
Hr2g6177b	HrL7	Cytochrome P450 monooxygenase ORF11	CYP60B-like; CypX; p450
Hr2g6177c	HrL6	Transcription factor ORF10	Zn(II)$_2$Cys$_6$ transcription factor
Hr2g6178	HrL5	Short-chain dehydrogenase EriB	Rossmann-fold NAD(P)-binding domain
Hr2g6179	HrL4	FAD-dependent monooxygenase Prx3	FAD_binding_4
Hr2g6180	HrL3	Cytochrome P450 monooxygenase ORF9	CYP60B-like; CypX; p450
Hr2g6181	HrL2	SAT4 family membrane protein	No putative conserved domains
Hr2g6182	HrL1	Cytochrome P450 monooxygenase BraC	CYP503A1-like; CypX
Hr2g6183	HrTc	Aristolochene synthase	Terpene_cyclase_nonplant_C1; Terpene_syn_C_2

Table S5 Gene annotations of eremoxylarin D BGC

Locus_tag	Label	Predicted protein function	Domain hits
7855_g	XhL9	Cytosine/purine transport protein FcyB	CodB; SLC-NCS1sbd_CobB-like
7856_g	XhL8	Highly reducing polyketide synthase AzaB	PKS_KS; PS-DH; PKS_KR; Enoyl_red; PP-binding; PKS_MT
7857_g	XhL7	Cytochrome P450 monooxygenase ORF11	CYP60B-like; CypX; p450
7858_g	XhL6	Short-chain dehydrogenase Prx4	FabG; ADH_SDR_c_like
7859_g	XhL5	MFS-type transporter AstH, drug resistance transporter	MFS_Azr1_MDR_like; Efflux_EmrB
7860_g	XhL4	Cytochrome P450 monooxygenase ORF6	CYP56-like
7861_g	XhL3	Cytochrome P450 monooxygenase ORF9	CYP60B-like; CypX; p450
7862_g	XhL2	FAD-dependent monooxygenase Prx3	FAD_binding_4
7863_g	XhL1	Eremophilane O-acetyltransferase Prx11	Transferase; PLN02663
7864_g	XhTc	Aristolochene synthase	Terpene_cyclase_nonplant_C1; Terpene_syn_C_2
7865_g	XhR1	Cytochrome P450 monooxygenase EfuG	CYP7_CYP8-like; CypX
7866_g	XhR2	Short-chain dehydrogenase eriB	Retinol-DH_like_SDR_c_like; PRK06197
7867_g	XhR3	C6 finger domain transcription factor AclZ	GAL4-like Zn(II)$_2$Cys$_6$; Fungal Zn(2)-Cys(6)

Protein Sequences

HrTc
MAPMVDEYVSEPEPEVLLTQKKTKPAATSAQATLIVPSSDLTAQIHPRHEKVIAEVDGYFLQHWPFPDGKARKKFLA
AGFSRVTCLYFPKALDDRIHHACRLLTLLFLIDDILEHMSLEDGRAYNERLMPLFRGTVLPDRSIPVEWISYDLWESM
RAHDKGMADEIIEPVFTFMRAQTDSTRLTEMGLGQYLDYRERDVGKALLAALMRFSMALTVPSPDLELVRPVDRN
CSKHLSVVNDIWSYEKEVLAAQTLHEEGGMLCTAVAVFSKEAEISPEASKRVLYHLCREWELEHRTLVAKVLAQKDT
PVLRAYLQGLEFQMSGNELWSRTTLRYVQPAT

HrL1
MSSTPVLDTLLSTRLFNDSVAWASSLDKNDIKGLLLLTTVTLIIWRYLSQPKQRLVPGIPVIGGASSKDRIKNRERFRH
DSQAMLKEGYFRNNVDGGFFYVPSPLGERLMLPIRYLEDLKTAPMDKVDFVGTFIEMFEGKYTTFGSRSTLHPRTVK
ADLNQHLPDVMMDVQDEIAECFDEIFPKCSETEWTEVPLVDVITRIVARVSSRMFGGPELSRNSSWVAASIAFAID
GYIGAQKLKRYPEFLKALVVRFIPEVRNLAKYYQEAENAALPMLAAREHMTERPKDLLAWMQEAAVGEEKDHRFIA
GILLKISFAAIHTTAAANSQLIFDLCANPELIPMLREEYEKVANEDGKIGKRGFFQMHIMDSIMKESQRFNPLLLITFER
IVTEDWRLSDGFVIPAHTNIGVPAQAIAMDPKLHPNPETFDGLRFMKLREATNDPAVKGKAQFAAANPQSMAFG
YGRHACPGRFFASDEIKAITMYLLNHYEIKFAEGQTRPKSMEVETQFLPDHAATILCKRRKAAV

HrL3
MEEGLIAMLKAHWVRVTGTLVIGYCLSHIVYNLYFHPLAKFPGPFLARSTLLWRFKNTMGGRQHRVFRVEHQKYG
DVFRVSPNELSFASVQSYRDIYGFPPAGQAQFIKSDFYDVFGSGFKTGCIGSERDPKVHAAKKKHLLAAFSPRSLAAQ
ESIIQRCTDAFVAKVGPLSRKDKAGIDMTKWFEMNAFDLLGEMAFGESFGCIAEEKHHFWIDLILTHLREIVLVDNLR
RFRILATLGKLLLPSLATKVRATHQQYSRDKVQRRLESKSPRQDFFTNIVAKVKSGEVGLEELTAHASTLIIAGGETTAT
TLTAALYYILRAPEVRSKLTDEIRGRYKSYDEIDSASALQLPYLQAVINEALRVHPSGAHGFQRISPGATVDGHWVPA
GTEVYTSTWSVSHDKRYFEDPDAFRPERWVDPESKDIKEASQPFSLGYRACIGRSFAFVEMSLVLAKLFYSYDMELV
DGDLDWETQSRHWVMWWKAPVRIRARDALSS

HrL4
MLSYMLPRLLFLQLGLLALSAMALSVSRITGKESNLGRLQEGATGACDALSAALPDHVFWPDSSDYLNQSQNVWS
QTCVKTPQCVFEPADVAALSQGLKLIHDAESQFAFRAGGHMPVPGAQSLDDGVMISASKFNTNNLSADGSIASVG
PGQTWMDVYQWLAPRGLAINGGRFPSVGVGGLLVGGGMGYFSGTQGWAVDDIVGWQVVLWNGTVLELVDA
AQDPNADLAWALRGGSNLFGLVTRFDVRTFPVTSAFGGLTVWSSAAGPEVLGSLASFMAPGGGVDDPKAHIDVF
SGISFPNGTPTLEYYNIALYLGGEEGPAALENFTAIPEDVTVSNGVAVHDSWTAIPQQLDSFSTRALRNLFYAISFEAT
EESVKLYNKTIVENAVEMISDVKGLTTYAVYQPISKKFLEASKAKGSNVLGLDPDADGSFIAGIVLSMWENAEDDEAV
LEFSRVSAQQIREQTEALGLHNTFTYLGDSAQGQSPFKSYANGKNLERLRAIRDKYDPDGFLERYLHRGFPLN

HrL5
MVKLDFDLKRDVPELTGKVILITGGTNGLGAATAKMLASRSPAKIYITGRNESSAQRVIAGIKSTGSTTEVVWIGCDH
TKLETVKEAADTILARETRLDVLMANAGIMALPPGLTKDGYELHFGINHVAHALLIRKLLGLLQKTVKEHGEARIIPIA
SLALVLAPRKHGIVFDDLKTTQAYWLLGKWQRYAQSKLANLIYGRELARRYPEILTVVVDPGPSNTGLVSGLGFLDK
MIVYMGNINRFLDDDQGHLNQVWAVGVPKDKVKHGEFYEPVGKLTTGYTHWCLDQKLAEKLWDWTEEQLKPW
LT

HrL7
MFGTDALGPVAHHVISWTYQHLWILLAGIIVAKILYNGIYNVYFHPLSRFPGPRLAACSNVSFSKAFLRGRQPYETLRL
HRRYGPVVRVAPNELSFNTAQSFRDIYGFRPGHKTFVKSTFYEGGSFAAKGVSSIVSERDPVVHGQMRRLLSHAFSN
SSLLEQEELVTESVDQFVRRMRGNVCERVDIADLLERMAFDIIGNLAFGETFGALDTDERHPWIAITMGALTKGALV
DSFKRFPMLASIVKLIMHKQIAALIDDTNKNEDLAIKLVKRRVSLPITRKDFLTRILAQRAQVDADPDSKHQHKVSDVE
LAAHVSDFVLAGSETTSTVLSTATYYLLKTPSVYEKLASEIRSTFRTAGEINEATTRDLVYLNAVCKEAMRIYAPLPLGLP
REVPEGGDTVDGHFLPAGTIVATNPIAASLDSTNFTDPLSFKPERWLDGYQGEDDLEASQPFSLGPRGCLGKSLGW
MEMRQTLAKLIWNFDLELADPDLDWQRDSRMYTLWSKPHLHVRAKLVEH

HrL8
MASLRIDEYSIPDLTDKVAVITGGSSGIGFAAAKILLSKNATVHVLDVNAPSGASPGDIEPWQSWAAFHFHRCNVTS
WAEQLAAFDAIGRVDMVFANAGVSEPHDYFTHESLVDGREPSYGGLVDVNFVGVLHTVTLARRSMRAHGVREGS
VVITISAVAYAPEQSLPVFAATKIALVGLVRSLRSTMLAQEGITINGVAPAATVTSLLPPHLAAPIVAMGLPISNAGFV
GRALVYAATAKQDRKVEVYGKESDSELWLKENQERYNGRVILTLGDTYSELEGPIADLRPFWFGQENLRLTRAQQA
ATDFR

PrL3
MAVIFSSSALDSHTHVDKHLVRRPIRDLENNVDMLDHLSRVLTLSYQYHALGTAIALFACACAYALVAPRQPPKFPA
PQLYDETGPIDLIALDKTIREGFQNYKGKYFTLKEAHGETVILPTKFMEELKALPDNMLNLDDEIDERFLSEHSLFTTTS
VGGRISTVVNSVKNELTKTLGHLMGDIHEEVVYSFQELIPPCDDWTEIDIQSKLVRIVALVSGRIFVGLPMSRNQEYL
DCIIEFTLNVFFAVPEIRAYPRLLRWTSRYLNTKVRAVHKSLATMRRLMAPIIAGTKQQLEMGTGPHNMCAWNIKN
SNQKERDSLNIQAQMQLATSMAAIHTTSMTVTNAIFDLAARPEYLQPLRDELQDLRATEPLPYLNKSSMPKLRKLDS
FLKESHRLSPISLLNMRRKIVQPITLHDGTVLQPGMHIAFPLHQVSNDEDLWENPSQFDGFRFQKLRDLPGNESKYQ
FTATGTNNLDFGYGVHACPGRFFAANEIKMILVHLIDNFDFKFKGDIGRPDSLWTPGGYHPDPSVRVLLKRRLKA

PrL4
MNASKLPLGSFVGTTLLLFILYKLVKLAYYVGQAKKTGLPYTLVPVLETEFLGKLLTPLIRPLFTSRLSRGEGWPRWIRFS
ILDWAWEEKRRVHEELGDVFLLVSPEGLICYTADADMCWDVMNRRNEFLKPRDKYKVLEPYGPNVATTEGKAYNF
HVRITAPPFNDGSGANDLVWNEASDQARALMESWSQENTTRDLSLDINRLTLAVISYTGFGKRLDFETEVSDLRNKI
PPGYKMSLHHALHLVTTFMVKILLIPKWIMKMTSMKEIAIAHGELEKYMREMIRTETAKLSKDSEYQSADAKGNLLT
SVLRASAFEAKAAGRKQAFSEDEVLGNLFLYLLAGYETTANAMTYGFITLALRQDLQDRIIQEVDGVYAEAAAEGRTS
LNYTDDFEKFQYTYGFMYEVFRLYPGVCIITKMVPKDTTITVYPENNSPQQHVLPAECRVYLNVNAVHYHERYWPD
PWALKPDRWMGTIGVTPNSRPNKKVVAQDKSRQVRGTLMTFSGGARACLGRKFTQSEYISVLATVLGKYRIVLGE
GMDAKVVKQEIDHLAAGTVTLAPLKYVKLALKKRTDVKTG

PrL7
MTISPIPGLLFVYDQPPHSIYVLPFVISAAALCYFIGLIVFNLWFHPLARFPGPLLARSTLLWRMRMTLKGRIHRSIEAG
HQKYGPVLRVAPNELSFASVSSWKSIYGHRPGGMIPTKSEFYDMYGSGFNSLCIGSERDPEKHRQMKSFLSAAFSTK
ALLEQEPLVSQTVDAFITRLGNDGGSETKGLDMTKWTEMVAFDILGEMAFGQSFECIIRGEPHYWQEMILKHLYFIT
VADNLRRLPFALTLARFLAPVLTAVRNKHSQFTRDKVAERMTNKNLRKDFMSNLISKVESGEVDREEMTAHASTLII
AGGETVATFLAATVYYLLKTPEVYKAMREEIRNRFPTYESINATSAQQLPYLQAVINEGLRIYPPGSQGFPRLSPGLAI
DGEWIPEGTEIYTSAWTVTHNPQMFKDPMKFDPNRWLNEKSTDIKESSQPFSLGPRGCLGRNFALMELNLILSKLC
WKYDMELMDQSLDWEGQSKVHVMWDKPALTVRFHSVDGSTLKA

PrL9
MDALEAPELLLLPHLSALTPKTGFLIGLALAITAYCYYRHTQHPLYKFPGPRSAAWSNVLYCYYYIQGRQPFKLLELHN
QYGSVVRTAPNDLSFNTAGAFRDIYNFRPGHETFIKSDWYDGGVFADKAHSIVSEREPGKHGHMRKYLSHAFSDKS
LKAQEPLIDEVVNEFVSQLDVFGSKKGGIDIVVWFNLATFDIIGSLAFGESFGGVKSGEVHPWISRIITAIGQSALADA
FKRFPKFATTFKWLFPKAIEKMLEDTAGHENYTISLIDKRLSNPSTRPDFLTRMLENRPEDLTDVQIAAHASDFVIAGS
ETTATTLSCIVYYLTKNPSVYQKATQEIRDRFERFEDINSTAASQLKYLHALALEAMRIYPPLPLALPRVVPKGGDTIDG
HFVAEGTIVSVNPVAACLSTKNFDAPLEFRPERWLESDLVDDHEASQPFSMGPRACLGRNLAWIELSLLLSKMLWV
YDIELLNTEVD

AoL3
MFEGAYTTLGTHSRLLPQVVRAQLNQYLPDVLPEIQFKVALQIHREIPFNAITECFAKSDWTVINVTELMAVLVARV
SSRMFGGPALSQNREWIEASLRFAHDGFNAAQKLKMWPDTLKFIGQHFIPEVRSIKNTYKIAERAIIPLLDEREVDKS
KKAHDLLTWMYDQAQGAEKDKKFIAGTLLKVSFAAYHTSAAAPTQLLFDIAAMPEHIAPLLGEYLSAPRDNNQNIS
VKGFAQMVKLDSIMKESQRFNPLLLLTFERIIKRDFTLSDGVVIPANTWIGCAAQAIGMDRKLYPDPDTFDAFRFVA
KEEATATSTSVPATKAHYTSANPGSMAFGYGQHACPGRFFAMMEIKAIIGEILSRFEMRLADGEMRPPSVTFETQH
LPHPAGKVLFKRRRCT

AoL2
MTLISLSLLALSLWIIIRVLVIIYRLAWHPLARFPGPKFAAATSAYEFYFDAIKGGQYTFEIGHMHKKYGPIVRISPHELHI
NDPGFIEELYPGPGKPRDKYAYATGQFGIPDVCSLVLTPYDLSSVFGAVSHDLHRMRRGALSPFFSKAAVTKLEPVIYS
AVDKLISRIEEVVESTGFVDLTMAFSCMTTDIVTQYAFAESSRFLENPDFTPNFHEAILAGTRMGSWARHFPILFPVL
RSIPIDILSRMSPETGVFLRWQESMRKKVSEIWQDQSALPVKDKNVSPFGSTIFHELFHSDMPDSEKHPGRMWQE
GQIVIGAGTETTAWTLTATTFFILDNPNILSKLRKELAATMPNRYEKPSCRELEALPYLIIIQEGLRLSFGVATRLQRINS
EAPMIFRQKKTNDTIEEKVWEIPTGTPVGMTAALVHLNPELFPDPHEFRPERWLDQDGQLHRGLDKYILSFSRGSR
QCIGINLAYSELYMGIGILIRRLGDRMQLFETDRTDVDMVEDCFVPVPRRESNGVRVRLSAQ

XhR1
MTAKMFDEYGLTLWLEAAPQHWKLSVGFGILLIPILTFIITTIISWIGWANNRHGREPPLNPYWIPFFGNWMSFLLA
RKSFVDRLYRRFGDTPVTVKLGHAKAYLLTKPESYGPILRDTKSCTNKAFAVVIMEQMFGMPKSAMHIYRNDKSGV
GAIPFPGSTIPAHLRVWHHHYRTATRYLQGDSLRHLSGEVVKHLSDELAKIDPNDPVDSGEWVDVPDFFTWWTHR
YFAATITALCGPHLIALNPGFVEDFWDYLNSWPSISKFYPRILAPKAYGARQRILDGIKRWHSHARQHSDYRHNGAD
APAWDEYWGSVWFKVRQRWGQDTGGMNDDALASEDLFVLTAATANAMPMAFWNLIEVYNDPALLGRIQAEL
SNAIVPPDANEDKLPYRFDINAITSSPLLQSVYAEVLRMRVSLFHNRSPTQGDYSLGPYKFKQGGLVCVSTNIASNHA
YTWRNRIDGGQRPLDKFWADRFLVSNPKDNNTMKFSTDGLDGAWIPYGGGALMCPGRHLAKQEMMSGVAIFD
AYFDMKLVKGVPRMDDGFFALGAQPPGEPVPVRMRRKVGISPVGTATTVKHS

2. Constructed Vectors

All the plasmids in this study were constructed by yeast recombination and selected in *E. coli*. The sequenced vectors were labelled with ID XX_ and were used by different combinations for *Aspergillus oryzae* transformations (Table S6). The features of all plasmids are depicted by cartoon maps correspondingly (Figure S2).

Table S6 Plasmids used in this study

Construct ID	Plasmids	Features
XX01	pTYGS_arg-*hrtc*	HrTc under control of PamyB, *argB* as the selection marker
XX02	pTYGS_arg-*hrtc-hrl4-hrl5-hrl8*	PamyB induces HrTc, Padh induces HrL4, PgpdA induces HrL5, Peno induces HrL8; selection marker is *arg*
XX03	pTYGS_ade-*hrl3-hrl1-hrl7*	PamyB induces HrL3, Padh induces HrL1, PgpdA induces HrL7; selection marker is *ade*
XX04	pTYGS_arg-*hrtc-hrl4 -hrl8*	PamyB induces HrTc, Padh induces HrL4, Peno induces HrL8; selection marker is *arg*
XX05	pTYGS_arg-*hrtc-hrl5-hrl8*	PamyB induces HrTc, PgpdA induces HrL5, Peno induces HrL8; selection marker is *arg*
XX06	pTYGS_ade-*hrl1-hrl7*	Padh induces HrL1, PgpdA induces HrL7; selection marker is *ade*
XX07	pTYGS_ade-*hrl3-hrl7*	PamyB induces HrL3, PgpdA induces HrL7; selection marker is *ade*
XX08	pTYGS_arg-*hrtc-hrl8*	PamyB induces HrTc, Peno induces HrL8; selection marker is *arg*
XX09	pTYGS_ade-*prl3-prl4*	Padh induces PrL3, PgpdA induces PrL4; selection marker is *ade*
XX10	pTYGS_met-*prl7-prl9*	PgpdA induces PrL7, Peno induces PrL9; selection marker is met
XX11	pTYGS_ade-*prl3*	Padh induces PrL3; selection marker is *ade*
XX12	pTYGS_ade-*prl4*	PgpdA induces PrL4; selection marker is *ade*
XX13	pTYGS_met-*prl7*	PgpdA induces PrL7; selection marker is *met*
XX14	pTYGS_ade-*aol3-aol2*	Padh induces AoL3, PgpdA induces AoL2; selection marker is *ade*
XX15	pTYGS_ade-*xhr1*	PgpdA induces XhR1; selection marker is *ade*

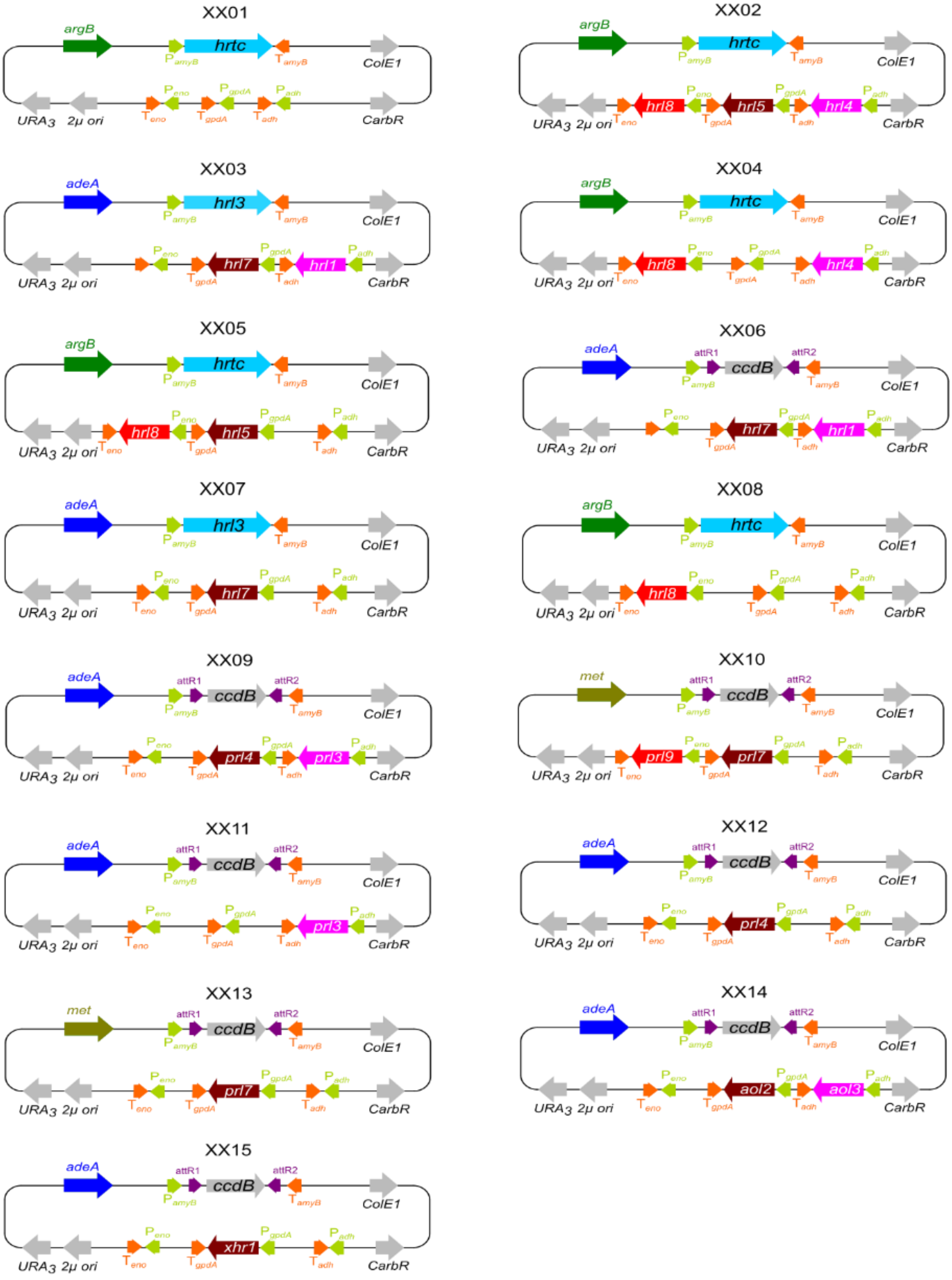

Figure S2 The maps of the plasmids in this study

3. Transformation and Selection of *A. oryzae*

3.1 Preparation of Protoplasts

A. oryzae NSAR1 was grown on a DPY plate for 5-7 days. Conidia were inoculated into 50 mL GN medium in a 250 mL flask, which was incubated overnight at 28 °C, 110 rpm. The grown mycelia were collected by a sterile Mira-cloth filter and were incubated in 25 mL 0.8 M NaCl solution containing 15 mg/mL lysing enzyme in a 50 mL-Falcon tube on a Stuart SB3 rotator at a speed of 6 for 4 hours, at room temperature. The protoplasts were released from hyphal strands by gentle pipetting with a wide–bore pipette. Afterwards, the supernatant was filtered through a sterile Mira-cloth filter and collected in a new 50 mL-Falcon tube and was centrifuged (5 min, 3000 x g) for collecting protoplasts.

3.2 Transformation and Selection

The resulting supernatant was discarded and the pellet, which is the protoplasts was suspended in 1 mL solution 1 and divided into 10 tubes by 15 ml-Falcon. The plasmids were added into the protoplast solution, for one plasmid, 1 μg of the plasmid was used for one tube; for two plasmids, 3 μg of each plasmid was used for one tube; for three plasmids, 6 μg of each plasmid was used for one tube. 10 µL water was added to one Falcon as a negative control. The empty plasmids were used as a positive control according to the type of selection media. The mixture of protoplast solution and plasmids was incubated on ice for 2 min. Then 1 mL solution 2 was added and the tube was turned upside down several times gently to mix the protoplasts, solutions and plasmids sufficiently. Subsquently, the tubes were incubated for 30 min at 28 °C. Pre–warmed 12 ml CZD/S soft agar was added to each tube and mix throughly, then overlaid onto two prepared CZD/S agar plates. Plates were incubated at 28 °C for 4–5 days until colonies appeared. The colonies that emerged were transferred to another CZD/S selection plate for one day. The procedure was repeated by streaking out single colonies on new CZD/S plates. The colonies were then cultivated for 5-7 days on DPY agar. The spores and mycelia were scratched into DPY medium for fermentation. Meanwhile the spores were transferred to make glycerol stocks.

DPY agar/ medium
2.00 % (w/v) Dextrin from potato starch (Sigma Aldrich) 1.00 % (w/v) Polypeptone (Roth) 0.50 % (w/v) Yeast extract (Duchefa Biochemie) 0.50 % (w/v) Monopotassium phosphate (Roth) 0.05 % (w/v) Magnesium sulfate hexahydrate (Sigma Aldrich).

GN medium
2.00 % (w/v) D (+)-Glucose Monohydrate (Roth); 1.00 % (w/v) Nutrient broth Nr. 2 from Oxoid (Fisher Scientific).

CZD/S Agar (soft agar was made by 0.80 % (w/v) Agar)
3.50 % (w/v) Czapek Dox broth (Duchefa Biochemie); 18.22 % (w/v) D-Sorbitol (= 1 M) (Roth); 0.10 % (w/v) Ammonium sulfate (Roth); 0.05 % (w/v) Adenine (Roth); 0.15 % (w/v) L-Methionine (Roth); 1.50 % (w/v) Agar (Duchefa Biochemie).

CZD/S1 Agar (soft agarwas made by 0.80 % (w/v) Agar)
3.50 % (w/v) Czapek Dox broth (Duchefa Biochemie); 18.22 % (w/v) D-Sorbitol (= 1 M); (Roth) 0.10 % (w/v) Ammonium sulfate (Roth); 0.15 % (w/v) L-Methionine (Roth); 1.50 % (w/v) Agar (Duchefa Biochemie).

CZD/S1 Agar w/o Methionine (soft agar was made by 0.80 % (w/v) Agar)
3.50 % (w/v) Czapek Dox broth (Duchefa Biochemie); 18.22 % (w/v) D-Sorbitol (= 1 M) (Roth); 0.10 % (w/v) Ammonium sulfate (Roth); 1.50 % (w/v) Agar (Duchefa Biochemie)

Solution 1: 0.8 M Sodium chloride, 10mM Calcium chloride, 50 mM Tris-HCl pH 7.5.

Solution 2: 60% (w/v) PEG3350, 0.8 M Sodium chloride, 10 mM Calcium chloride, 50 mM Tris-HCl pH 7.5

Table S7 Combinations of plasmids for each experimental group

EXP	*hrtc*	*hrl1*	*hrl3*	*hrl4*	*hrl5*	*hrl7*	*hrl8*	*prl3*	*prl4*	*prl7*	*prl9*	*aol2*	*aol3*	*xhr1*	Plasmids
1	✓														XX01
2	✓	✓	✓	✓	✓	✓	✓								XX02+XX03
3	✓	✓	✓	✓		✓	✓								XX03+XX04
4	✓	✓	✓		✓	✓	✓								XX03+XX05
5	✓	✓		✓	✓	✓	✓								XX02+XX06
6	✓		✓	✓	✓	✓	✓								XX02+XX07
7	✓	✓	✓			✓	✓								XX03+XX08
8	✓	✓				✓	✓								XX06+XX08
9	✓							✓	✓	✓	✓				XX01+XX9+ XX10
10	✓							✓							XX01+XX11
11	✓								✓						XX01+XX12
12	✓								✓	✓					XX01+XX12 +XX13
13	✓											✓	✓		XX01+XX14
14	✓													✓	XX01+XX15

4. Fermentation, Analysis and Compound Purification

4.1 Fermentation and Extraction Protocols

Transformants were isolated from DPY agar plates by scraping, and then 1 mL spore suspension was inoculated into 100 mL DPY-medium in a 500 mL baffled flask and incubated at 28 °C with 110 rpm shaking for 5-7 days. A hand blender was used to homogenise the whole culture, and then filtration was used to separate the mixture allowing the chemical to be released into the culture. The pH of the supernatant was adjusted to between 3 and 4 using 2 M hydrochloric acid, and then it was extracted twice with ethyl acetate. After the organic layers were separated, they were dried over $MgSO_4$, and the solvent was subsequently removed under decreased pressure. After dissolving the crude extract in methanol to a concentration of 10 mg/mL, the solution was filtered through a glass wool before being tested by LCMS. The concentration of the crude extract employed for the purification process was 50 mg/mL, after transformants were cultivated on a large scale (about 2 litres) for preparative LCMS.

4.2 Analytical and Preparative LC-MS

The collection of analytical LCMS data was conducted using a Waters LCMS system, which consisted of a Waters 2767 autosampler, a Waters 2545 pump, a Phenomenex Kinetex column (2.6 μm, C_{18}, 100 Å, 4.6 x 100 mm) equipped with a Phenomenex Security Guard precolumn (Luna, C_5, 300 Å), and with a solvent flow of 1.0 mL·min^{-1}. For detection purposes, two instruments were utilised: a Waters ZQ mass detector capable of operating in both ES^+ and ES^- modes within a mass range of 100 to 1000 *m/z*, and a 996 Diode Array detector with a wavelength range of 210 to 600 nm. In this study, two HPLC solvents were utilised: Acetonitrile (B) with a concentration of 0.045 % formic acid, and water (A) with an additional 0.05 % formic acid.

The purification of all compounds was carried out using a Waters mass-directed autopurification system consisting of a Waters 2767 autosampler, Binary Gradient Module 2545 with 515 HPLC pumps, and System Fluid Organiser. The purification process utilised a Phenomenex Kinetex Axia column (5 µm, C_{18}, 100 Å, 21.2 × 250 mm) equipped with a Security Guard pre-column (Luna C_5 300 Å). The elution of compounds occurred at a flow rate of 20 mL·min^{-1} at ambient temperature. The Waters Sample Manager 2767 instrument was employed to acquire fractions through either a mass-directed trigger or a time-dependent trigger. The fractions obtained from the mixture were subjected to vacuum evaporation as a preliminary step to remove the organic solvents. Subsequently, the resulting aqueous phases were dried using a Freeze Dryers and/or rotary evaportaor. The dried samples were weighed, dissolved, and subjected to analysis using HPLC prior to being submitted for nuclear magnetic resonance (NMR) analysis.

4.3 HRMS and NMR

The HR-ESI-MS studies utilised the Agilent 1200 Infinity Series High Resolution Electrospray Ionization-Mass Spectrometry (MS) instrument. The mass spectrometer utilised in this study was the maXis ESI-TOF instrument from Bruker Daltonics, Agilent Technologies. In the stationary phase, a Waters Acquity UPLC BEH C_{18} column (Milford, USA; 2.1 x 50 mm, 1.7 μm) was employed. The ultraviolet/visible spectra were recorded within the wavelength range of 200 to 600 nm. The NMR data were obtained by utilising three different apparatus: the Bruker Ascend 400, the Bruker Ultrashield 500, and the Bruker Ascend 600. The selection of apparatus was based on the weight of the compounds. Each apparatus was equipped with a cryo-cooled probe that operated at frequencies of 400/500/600 MHz (^1H) and 100/125/150 MHz (^{13}C), respectively.

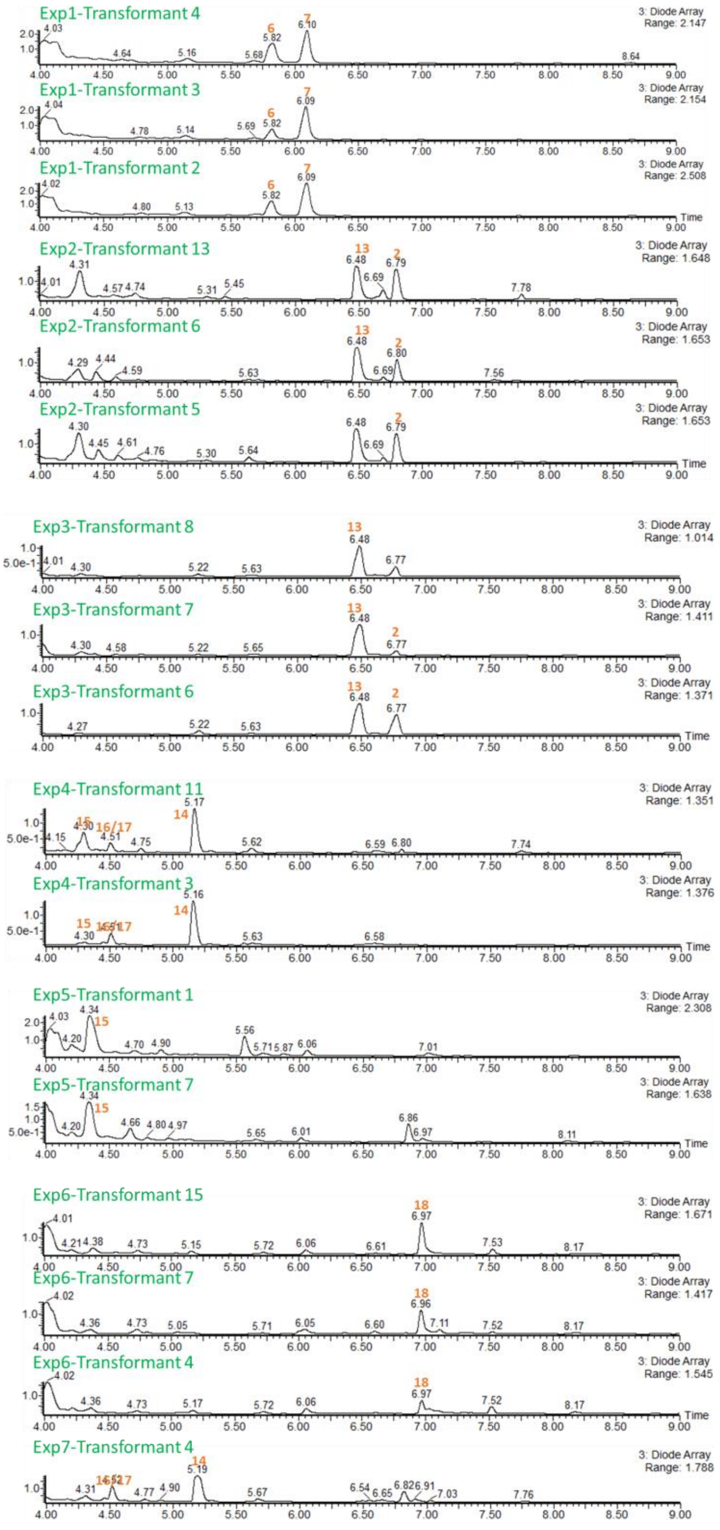

Figure S3 Chromatograms (DAD) of some transformants for each experiment.

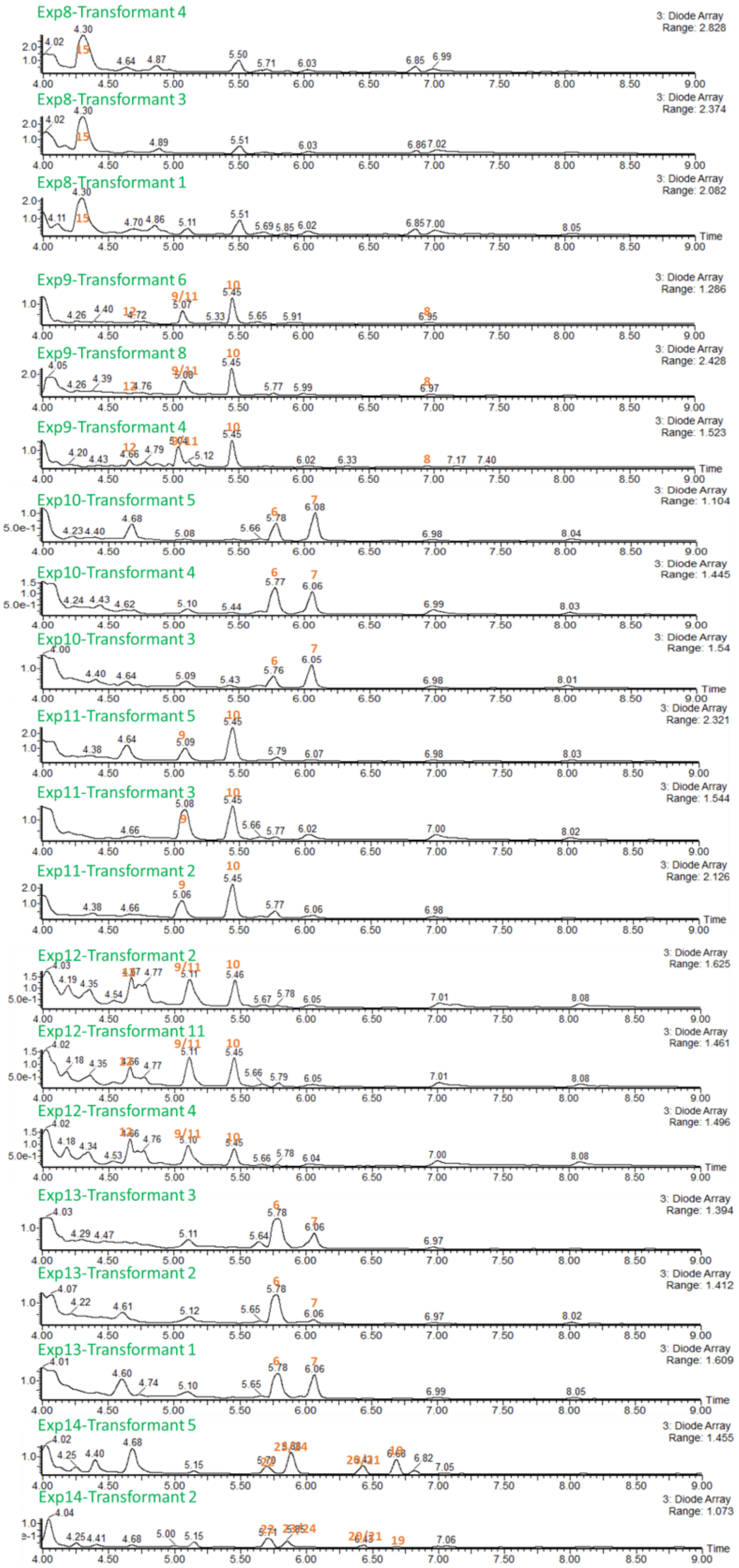

Figure S4 Chromatograms (DAD) of some transformants for each experiment.

5. Compound Characterization

Compounds	Titres (mg/L)	$[\alpha]^{20}_{D}$
**2**	8.5	+18.5 (*c* = 0.200, CH_3OH);<br>$[\alpha]^{25}_{D}$ +42 (*c* = 1.6, CH_3OH) from the literature [3]
**6**	5.0	-26.4 (*c* = 0.140, CH_3OH)
**7**	7.2	+48.3 (*c* = 0.180, CH_3OH);<br>$[\alpha]^{25}_{D}$ +120 (*c* = 0.2, CH_2Cl_2) from the literature [4]
**8**	4.2	+29.0 (*c* = 0.380, CH_3OH)
**9**	6.0	-27.0 (*c* = 0.270, CH_3OH)
**10**	4.7	+60.0 (*c* = 0.240, CH_3OH);<br>$[\alpha]^{20}_{D}$ +68 (*c* = 0.10, MeOH) from the literature [4]
**11**	6.0	+63.2 (*c* = 0.750, CH_3OH)
**12**	3.5	+150.6 (*c* = 0.180, CH_3OH)
**13**	15.0	+30.8 (*c* = 0.400, CH_3OH)
**14**	4.0	+21.8 (*c* = 0.220, CH_3OH)
**15**	5.0	+23.6 (*c* = 0.250, CH_3OH)
**16/17**	3.0	-
**18**	5.6	+43.3 (*c* = 0.150, CH_3OH)
**19**	4.0	+50.0 (*c* = 0.150, CH_3OH)
**20/21**	3.0	-
**22**	4.0	+14.0 (*c* = 0.050, CH_3OH)
**23/24**	10.0	-

Table S8 The titres (mg/L) and the $[\alpha]^{20}_{D}$ of all the isolated compounds, *c* reprecents the concentration (g/100ml), all the measurements were conductd at 20 °C.

Compound 2

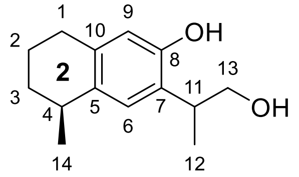

Chemical Formula: $C_{14}H_{20}O_2$
Exact Mass: 220.1463

Compound 2						
**Pos.**	**δ_C / ppm**	**δ_H / ppm (*J*/Hz)**	**^{1}H-^{1}H COSY**	**HMBC (H-C)**	**δ_C / ppm literature** [3]	**δ_H / ppm (*J*/Hz) literature** [3]
**1**	29.8	2.68, 2H, m	2	2, 3, 5, 9, 10	29.7	2.67, 2H, m
**2**	20.6	1.67, 1H, m;<br>1.85, 1H, m	2, 1, 3<br>2, 1, 3	1, 3, 4, 10<br>1, 3, 4, 10	20.5	1.67, 1H, m;<br>1.82, 1H, m
**3**	31.9	1.5, 1H, m;<br>1.88, 1H, m	3, 2, 4<br>3, 2, 4	1, 2, 4, 5<br>1, 2, 4, 5	31.8	1.49, 1H, m;<br>1.87, 1H, m
**4**	32.0	2.82, 1H, m	3, 14	2, 3, 5, 10, 14	31.9	2.83, 1H, m
**5**	134.4				134.3	
**6**	127.6	6.93, 1H, s		4, 8, 10, 11	127.3	6.92, 1H, s
**7**	128.3				128.2	
**8**	152.4				152.4	
**9**	117	6.6, 1H, s		1, 5, 7, 8	116.9	6.59, 1H, s
**10**	136.7				136.6	
**11**	37.1	3.19, 1H, pd (7.4, 7.4, 7.3, 7.3, 3.7)	12, 13	7, 8, 12, 13	36.8	3.19, 1H, dqd (7.8, 7.3, 3.7)
**12**	16.0	1.32, 3H, d (7.3)	11	7, 11, 13	15.8	1.30, 3H, d (7.3)
**13**	69.6	3.73, 1H, dd (9.8, 7.6);<br>3.93, 1H, dd (9.8, 3.8)	11, 13<br>11, 13	7, 11, 12<br>7, 11, 12	69.7	3.72, 1H, dd (9.8,7.8);<br>3.92, 1H, dd (9.8,3.7)
**14**	23.2	1.26, 3H, d (7.0)	4	3, 4, 5	23.1	1.24, 3H, d (7.9)

Table S9 Summarized NMR signals for ^{13}C, ^{1}H, ^{1}H-^{1}H COSY, HMBC for **2** recorded in $CDCl_3$. Literature [3] data was measured in $CDCl_3$

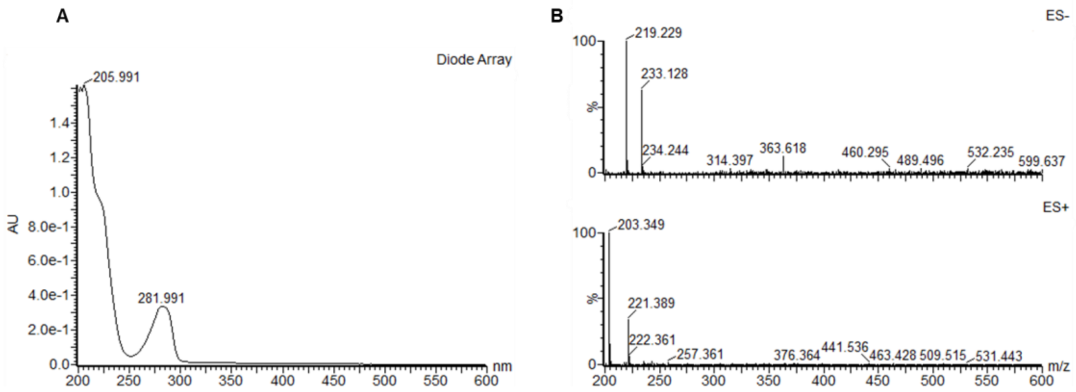

Figure S5 UV-absorption (**A**) and fragmentation pattern (**B**) of **2** in ES^- (top) and ES^+ TIC (bottom) by LR-LCMS

Elemental Composition Report **Page 1**

Single Mass Analysis
Tolerance = 20.0 PPM / DBE: min = -1.5, max = 50.0
Element prediction: Off
Number of isotope peaks used for i-FIT = 3

Monoisotopic Mass, Odd and Even Electron Ions
272 formula(e) evaluated with 5 results within limits (up to 30 closest results for each mass)
Elements Used:
C: 0-80 H: 0-110 N: 0-16 O: 0-10
Sun QTof Premier HAB321
YS 027, neg 745 (7.625) AM (Cen,3, 50.00, Ht,10000.0,554.26,0.70,LS 10) 1: TOF MS ES- 1.14e+002

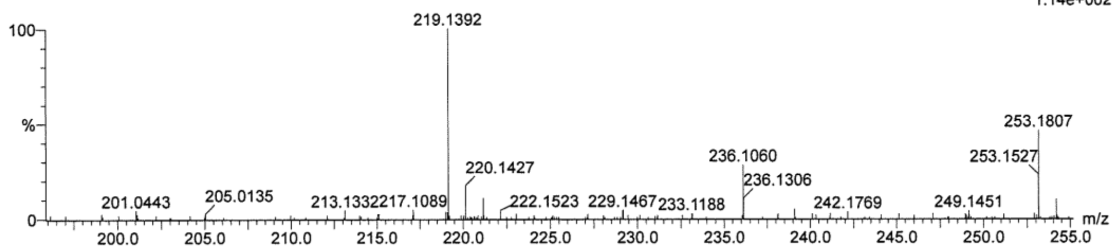

Mass	Calc. Mass	mDa	PPM	DBE	i-FIT	i-FIT (Norm)	Formula
Minimum:				-1.5			
Maximum:		5.0	20.0	50.0			
219.1392	219.1385	0.7	3.2	5.5	35.1	0.5	C14 H19 O2
	219.1372	2.0	9.1	6.0	35.9	1.4	C12 H17 N3 O
	219.1417	-2.5	-11.4	3.0	38.8	4.2	C2 H13 N13
	219.1358	3.4	15.5	6.5	37.0	2.4	C10 H15 N6
	219.1430	-3.8	-17.3	2.5	37.7	3.1	C4 H15 N10 O

Figure S6 HRMS data for **2**; *m/z* $(M-H)^-$ calc. mass is 219.1385, 219.1392 was found.

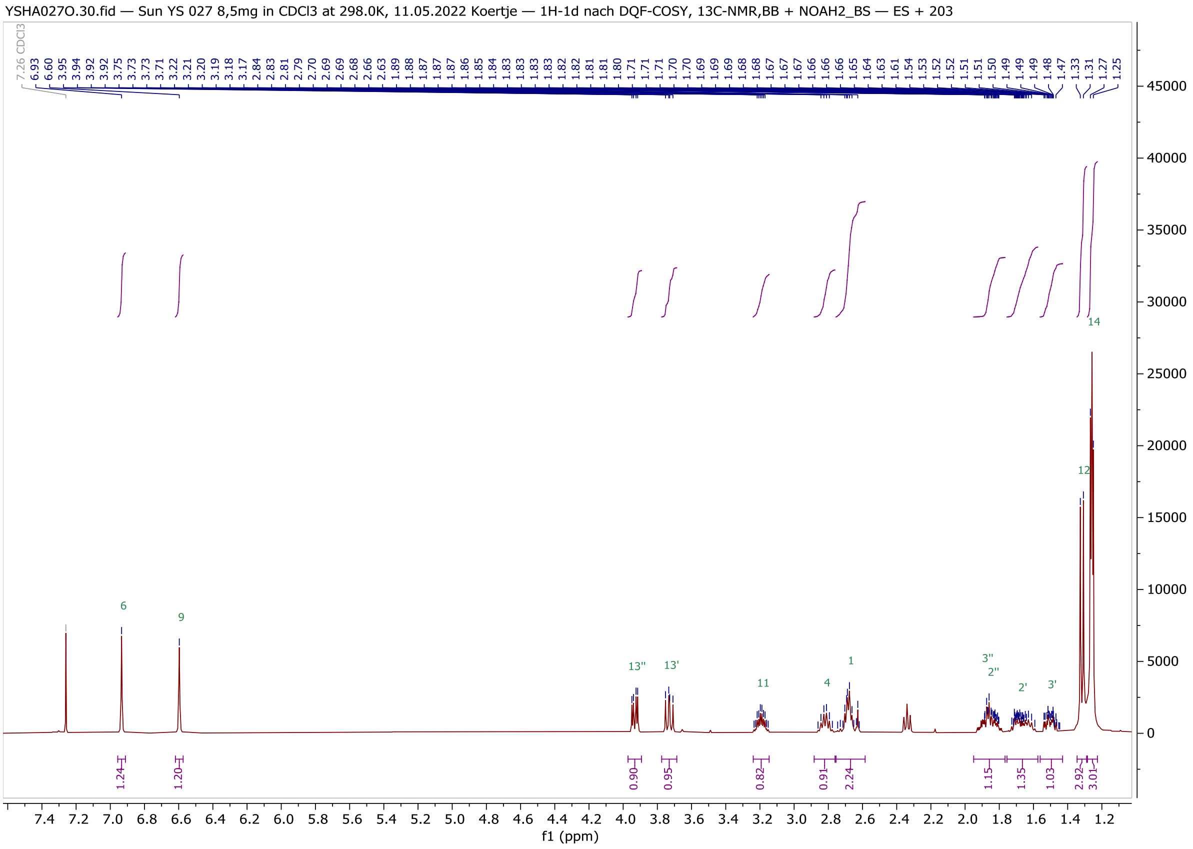

Figure S7 ^{1}H-NMR of **2** recorded at 400 MHz in $CDCl_3$

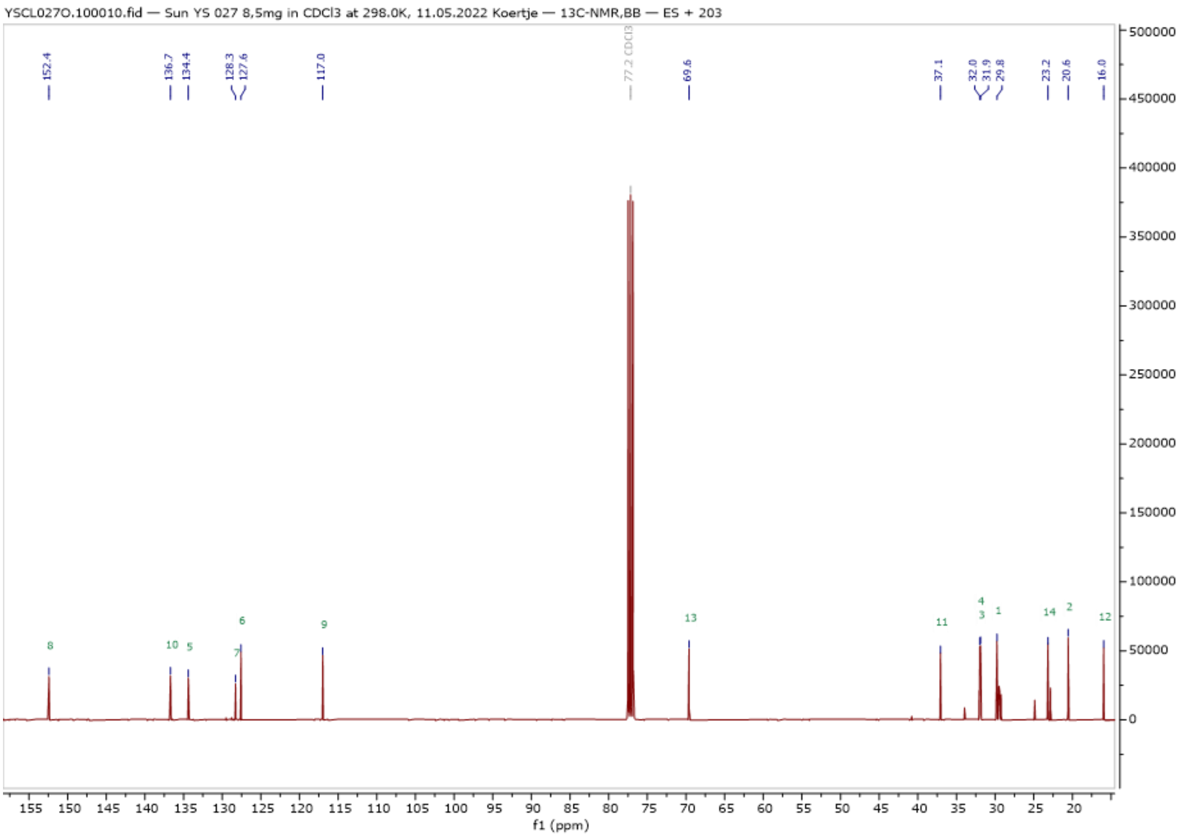

Figure S8 ^{13}C-NMR of **2** recorded at 100 MHz in $CDCl_3$

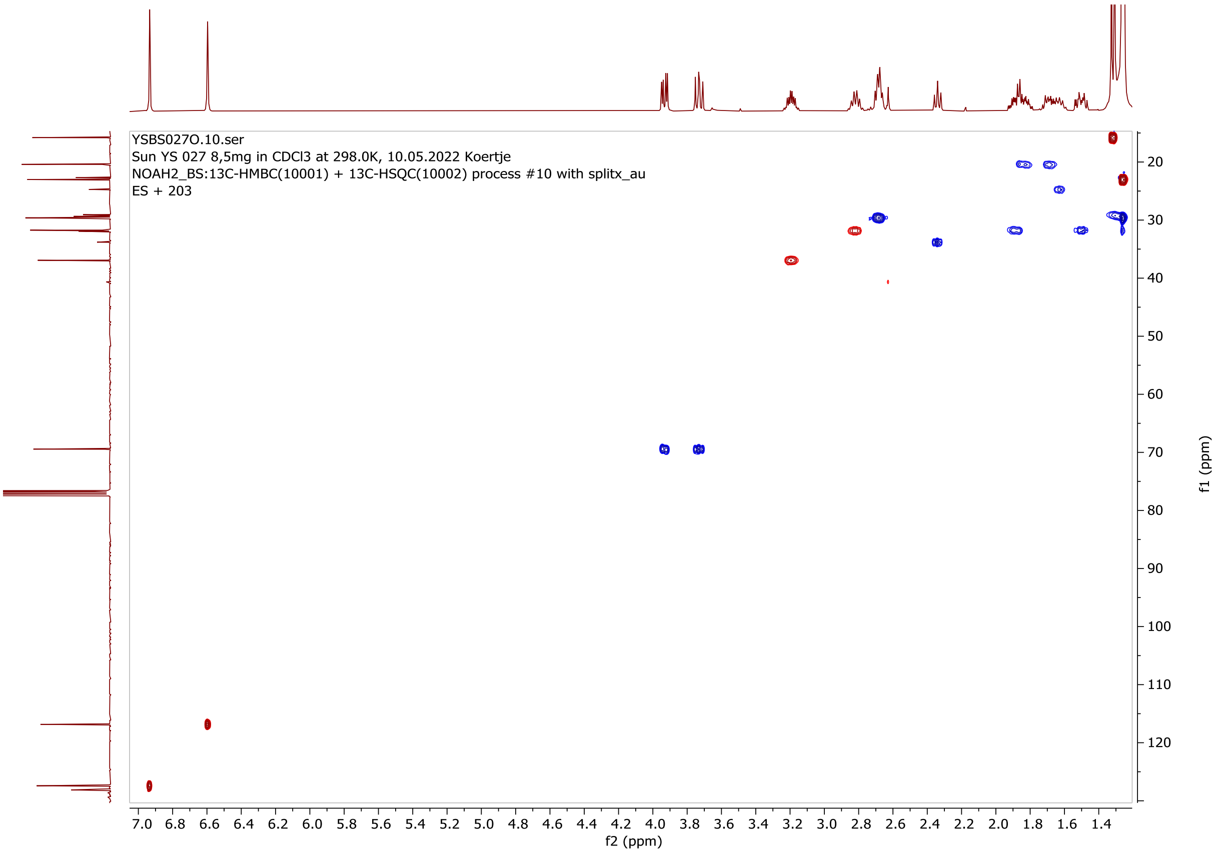

Figure S9 HSQC-spectrum of **2** recorded at 400, 100 MHz in $CDCl_3$

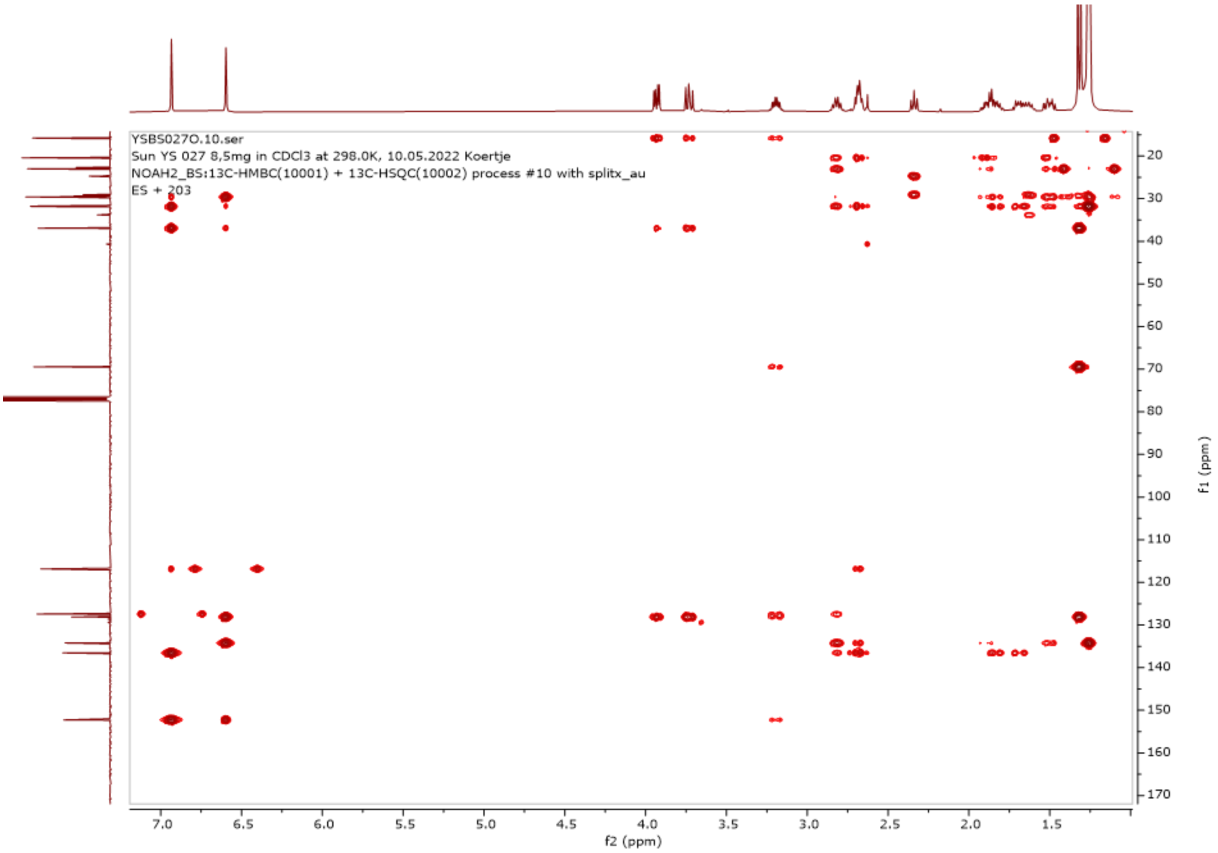

Figure S10 HMBC-spectrum of **2** recorded at 400, 100 MHz in $CDCl_3$

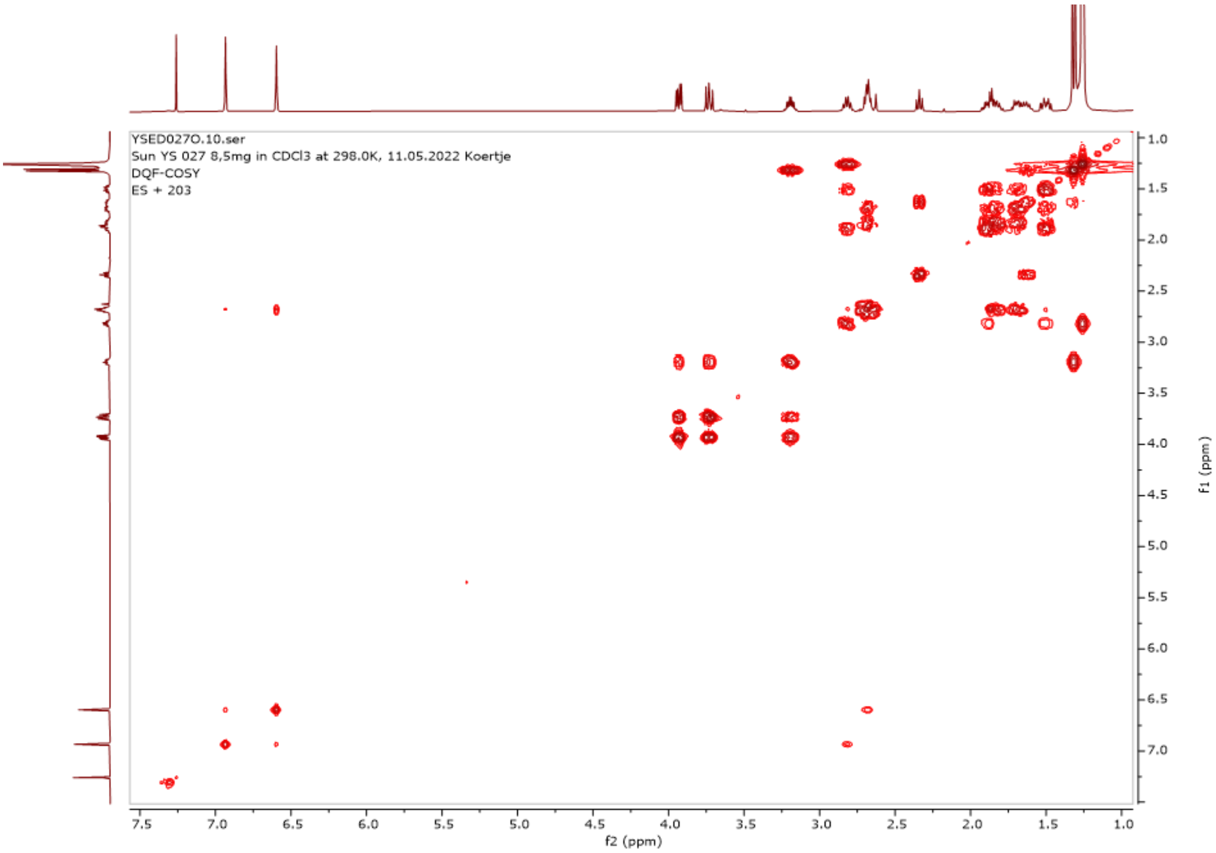

Figure S11 ^{1}H, ^{1}H-COSY-spectrum of **2** recorded at 400 MHz in $CDCl_3$

Compound 5

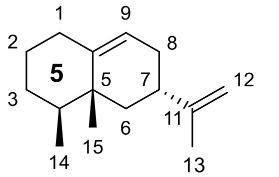

Chemical Formula: $C_{15}H_{24}$
Exact Mass: 204.1878

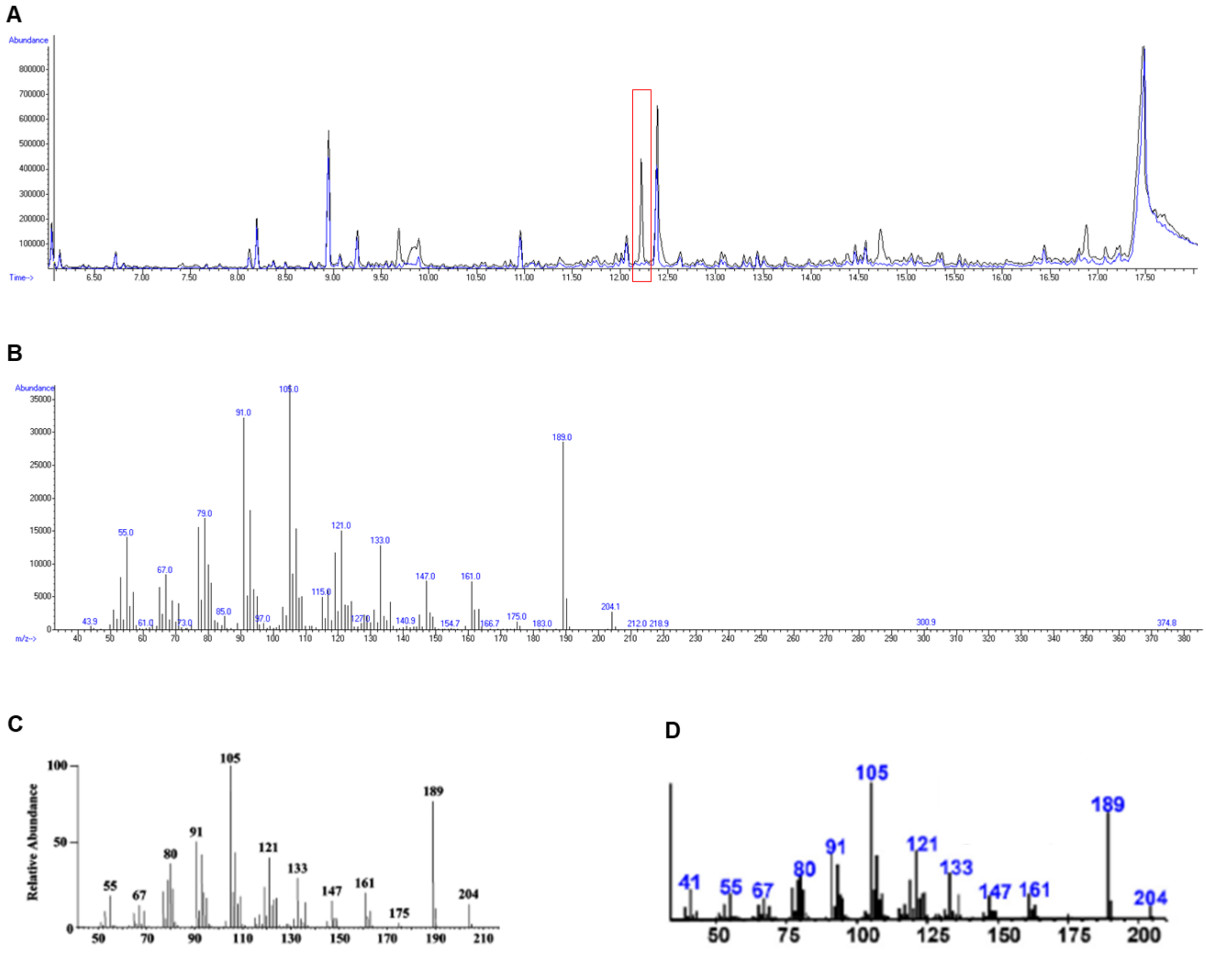

Figure S12. Detection of (+)-aristolochene **5** by GC-MS. **(A)** The chromatogram overlay of the control *A. oryzae* NSAR1 (in blue) and the transformant *A. oryzae* NSAR1+ HrTc (in black); the peak of **5** was labelled in a red box. **(B)** Mass information of compound **5. (C) (D)** Standard spectrums of (+)-aristolochene from literature. [5, 6]

Compound 6

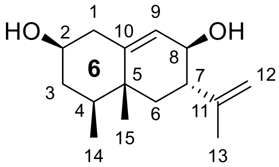

Chemical Formula: $C_{15}H_{24}O_2$
Exact Mass: 236.1776

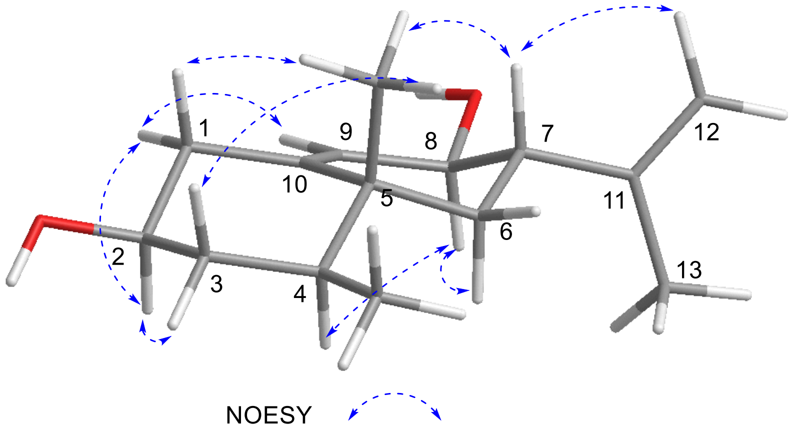

Compound 6				
**Pos.**	**δ_C / ppm**	**δ_H / ppm (*J*/Hz)**	**^1H-^1H COSY**	**HMBC (H-C)**
**1**	41.7	2.15, 1H, dddd (13.2, 11.4, 2.2, 2.2);<br>2.37, 1H, ddd (12.8, 5.0, 2.3)	1, 2, 8, 9<br>1, 2, 3	2, 9, 10<br>2, 3, 5, 9, 10
**2**	70.9	3.6, 1H, dddd (11.3, 11.3, 4.8, 4.8)	1, 3	
**3**	40.1	1.39, 1H, ddd (12.5, 12.3, 10.8);<br>1.77, 1H, m	3, 2, 4<br>3, 2, 1	2, 4, 5, 14<br>4
**4**	41.4	1.31, 1H, m	14	3, 5
**5**	38.1			
**6**	41.0	1.26, 1H, m;<br>1.67, 1H, dd (13.2, 2.6)	6, 7<br>6, 7	7, 8, 15<br>7, 8, 10, 15
**7**	47.7	2.22, 1H, ddd (12.6, 9.5, 2.6)	6, 8	6, 8, 11, 12, 13
**8**	69.1	4.07, 1H, ddd (9.5, 2.2, 2.2)	7, 9, 1	9, 10, 11
**9**	125.1	5.42, 1H, dd (1.9, 1.9)	1, 8	1, 5, 7
**10**	143.6			
**11**	146.5			
**12**	112.7	4.89, 1H, m;<br>4.91, 1H, m	12, 13<br>12, 13	7, 13<br>7, 13
**13**	19.5	1.73, 3H, dd (1.1, 1.1)	12	7, 11,12
**14**	15.1	0.87, 3H, d (6.7)	4	3, 5
**15**	17.6	1.02, 3H, s		4, 5, 10

Table S10 Summarized NMR signals for ^{13}C, ^1H, ^1H-^1H COSY, HMBC for **6** recorded in $CDCl_3$

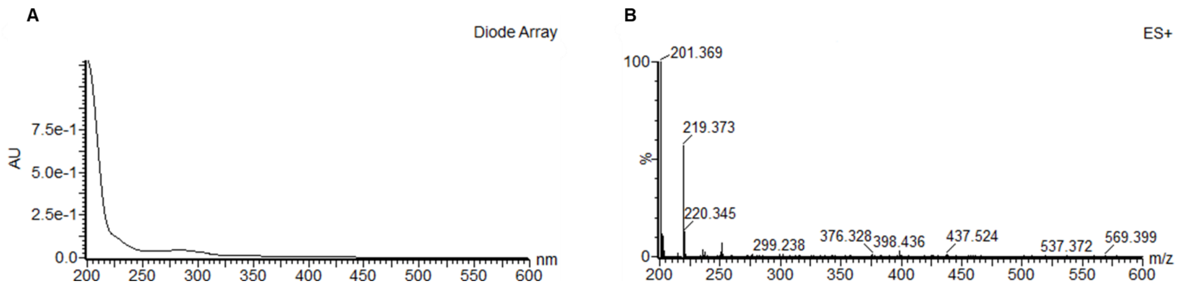

Figure S13 UV-absorption **(A)** and fragmentation pattern **(B)** of **6** in ES$^+$ TIC by LR-LCMS

Elemental Composition Report **Page 1**

Single Mass Analysis
Tolerance = 40.0 PPM / DBE: min = -1.5, max = 50.0
Element prediction: Off

Monoisotopic Mass, Odd and Even Electron Ions
26 formula(e) evaluated with 1 results within limits (up to 50 closest results for each mass)
Elements Used:
C: 0-25 H: 0-35 O: 0-4 Br: 0-1

Sun
YS 021 2810 (11.715) AM (Cen,5, 60.00, Ar,5000.0,190.00,1.00); Cm (2807:2822)

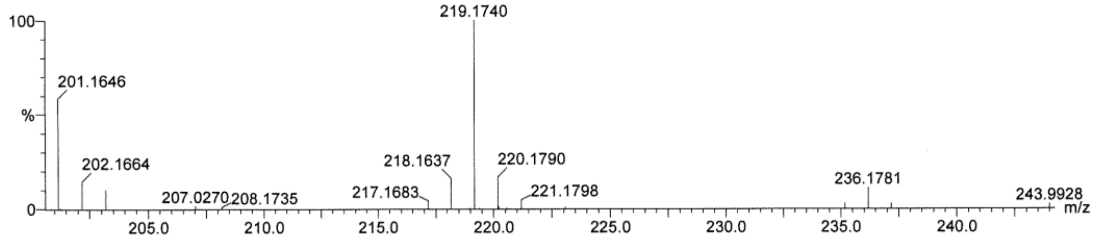

Minimum:				-1.5		
Maximum:		5.0	40.0	50.0		
Mass	Calc. Mass	mDa	PPM	DBE	i-FIT	Formula
236.1781	236.1776	0.5	2.1	4.0	2773022.3	C15 H24 O2

Figure S14 HRMS data for **6**. M calc. mass is 236.1776, 236.1781 was found by HR-GCMS

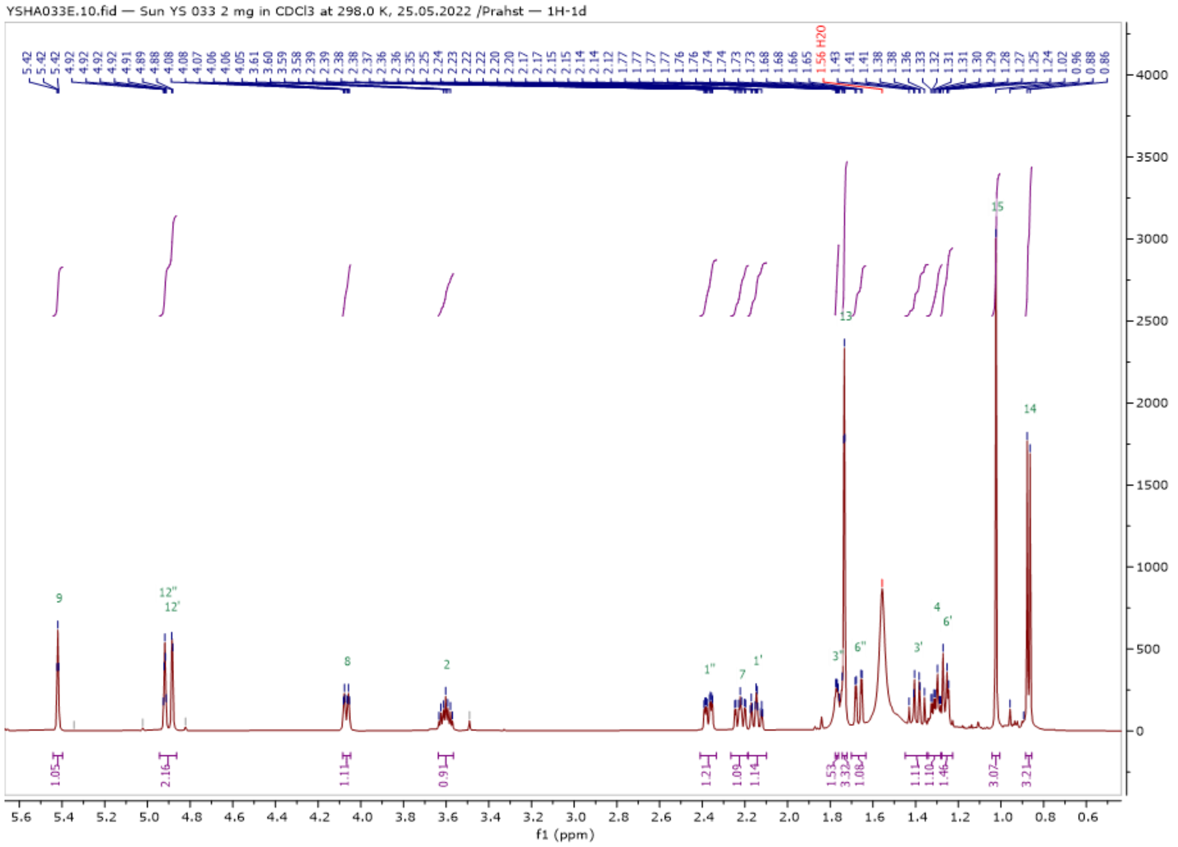

Figure S15 ^{1}H-NMR of **6** recorded at 500 MHz in $CDCl_3$

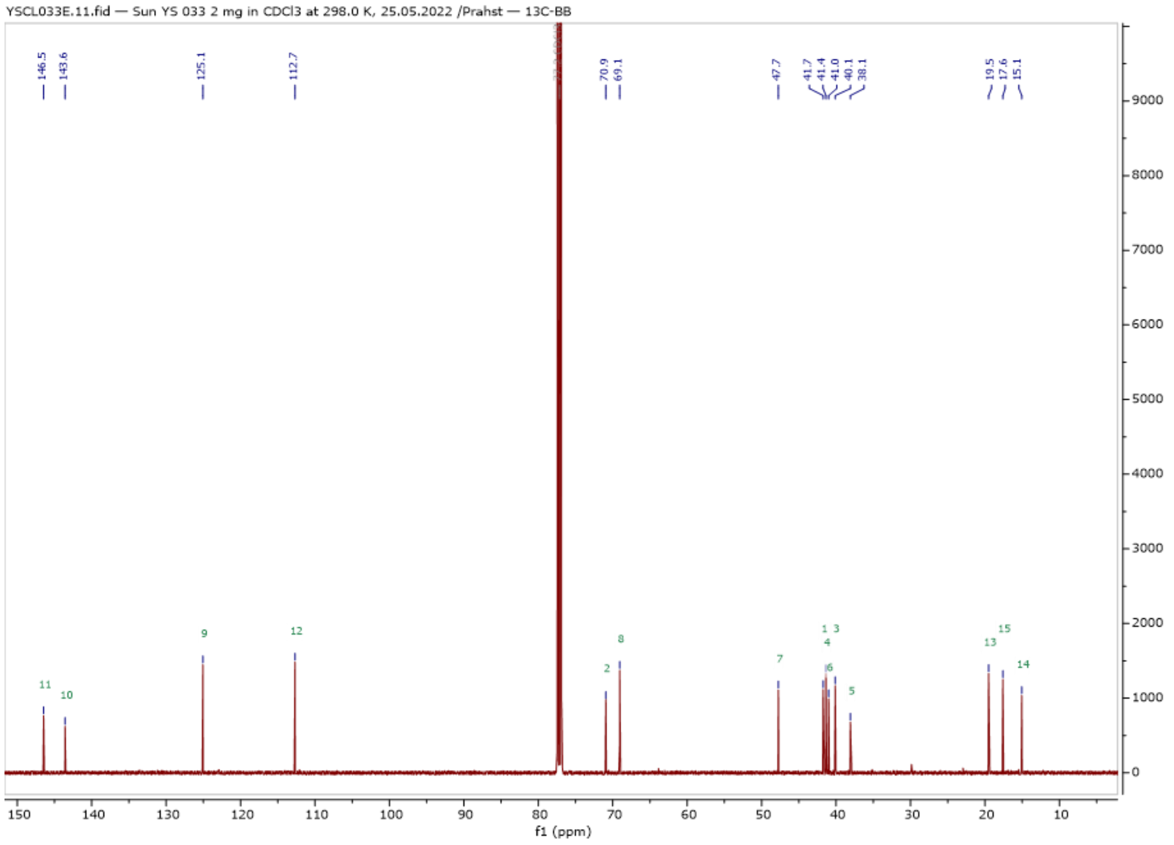

Figure S16 ^{13}C-NMR of **6** recorded at 125 MHz in $CDCl_3$

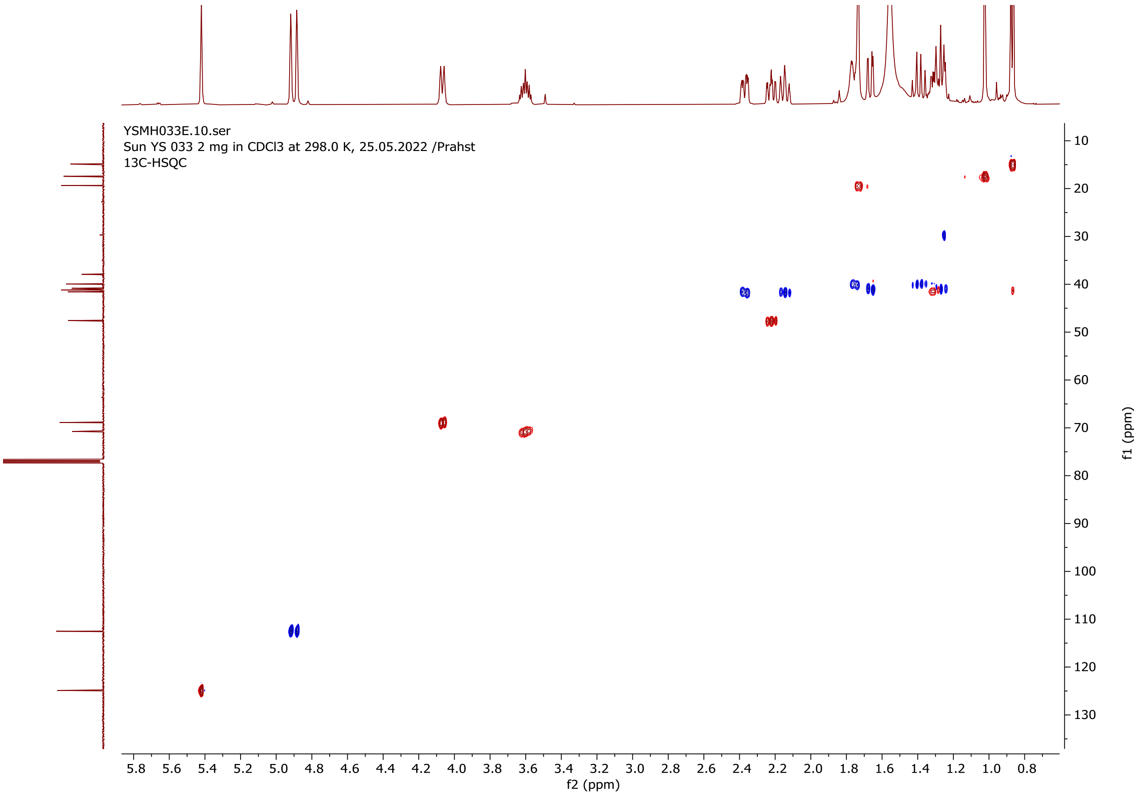

Figure S17 HSQC-spectrum of **6** recorded at 500, 125 MHz in $CDCl_3$

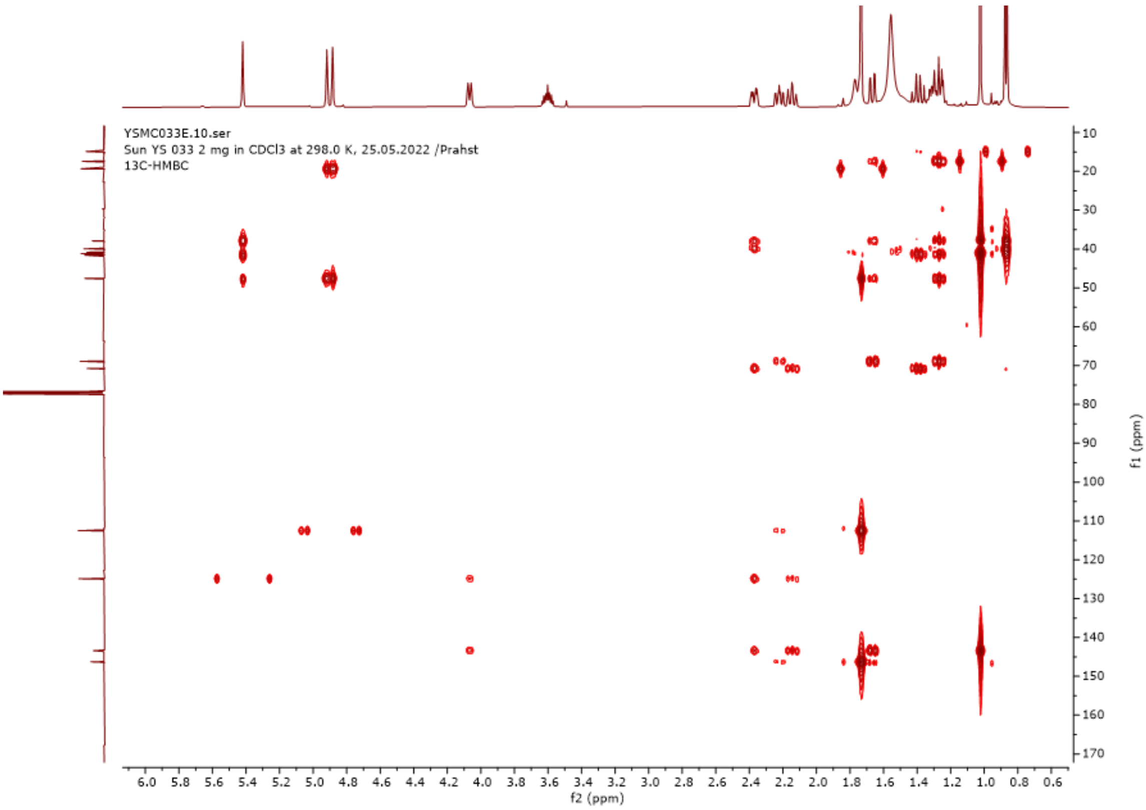

Figure S18 HMBC-spectrum of **6** recorded at 500, 125 MHz in $CDCl_3$

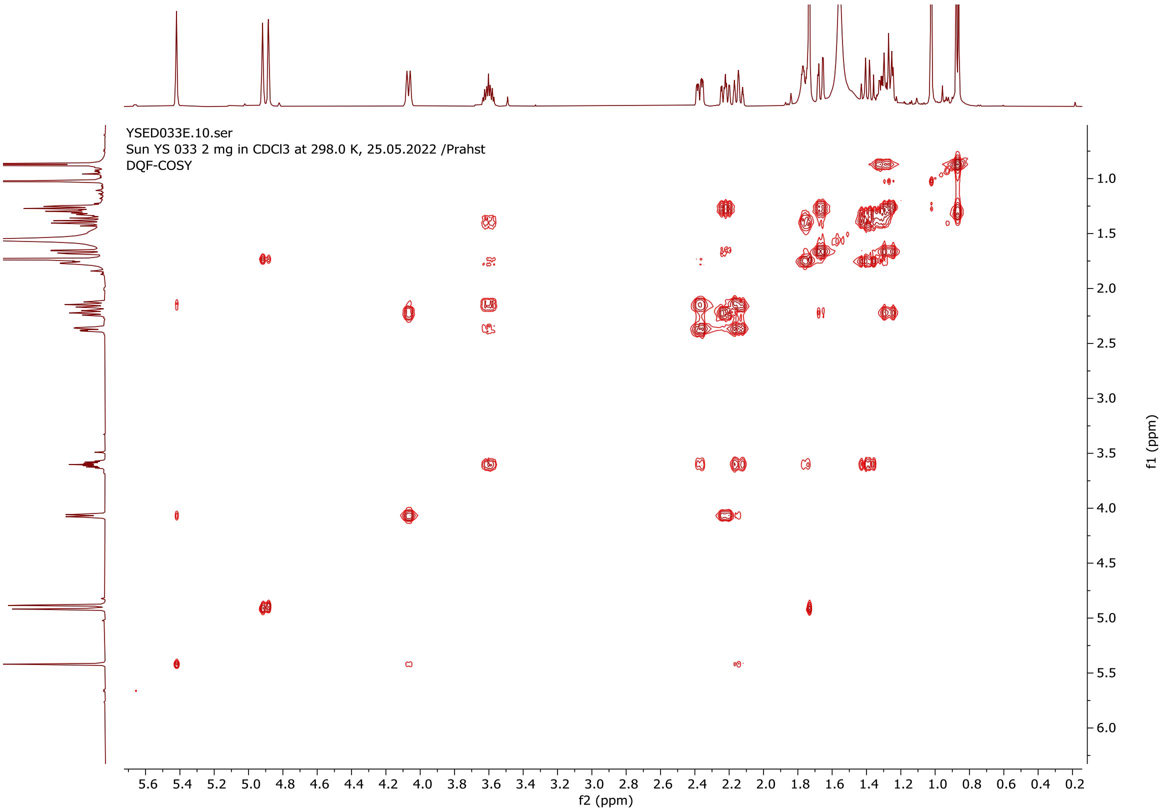

Figure S19 ^{1}H, ^{1}H-COSY-spectrum of **6** recorded at 500 MHz in $CDCl_3$

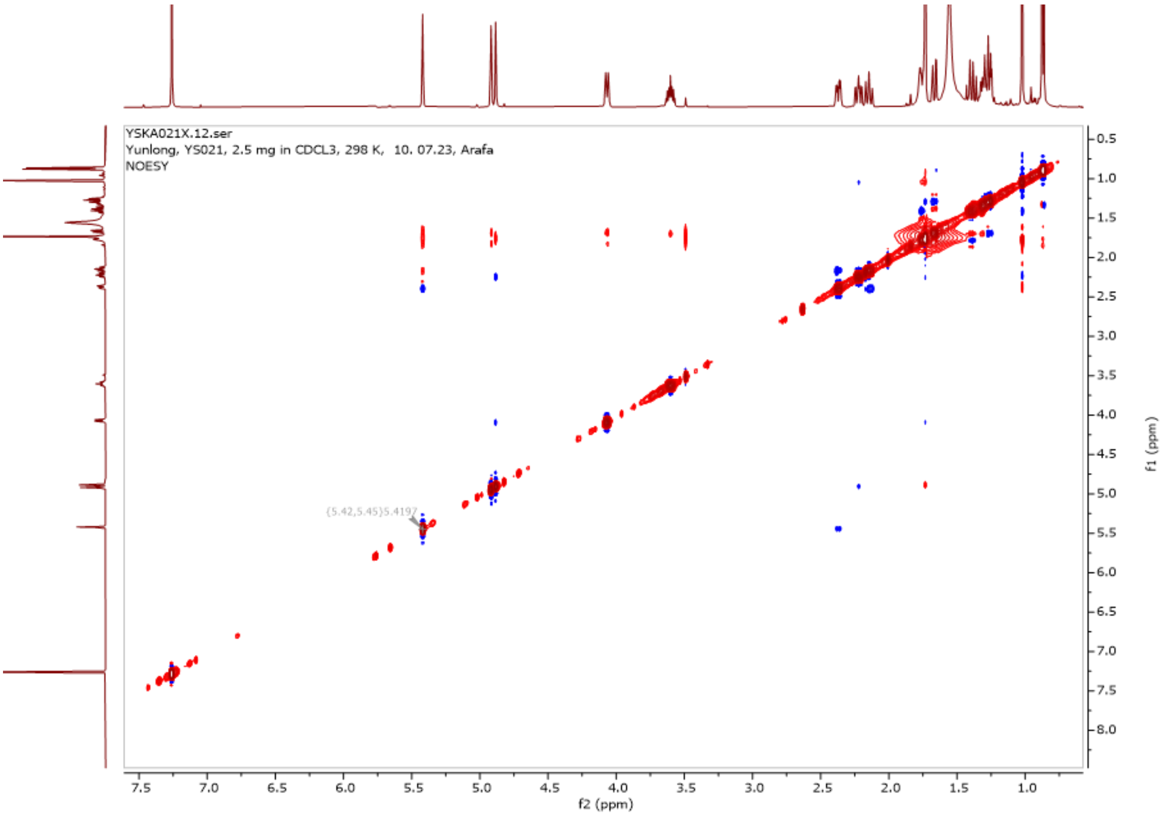

Figure S20 NOESY-spectrum of **6** recorded at 600 MHz in $CDCl_3$

Compound 7

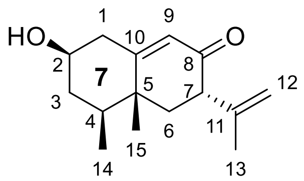

Chemical Formula: $C_{15}H_{22}O_2$
Exact Mass: 234.1620

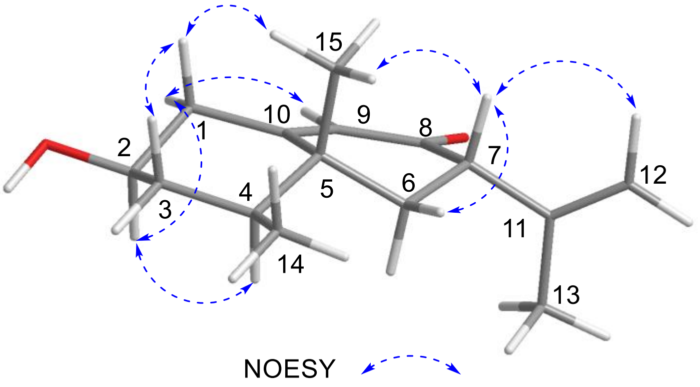

Compound 7						
**Pos.**	**δ_C / ppm**	**δ_H / ppm (*J*/Hz)**	**^{1}H-^{1}H COSY**	**HMBC (H-C)**	**δ_C / ppm literature [4]**	**δ_H / ppm (*J*/Hz) literature [4]**
**1**	42.2	2.33, 1H, ddd (13.6, 11.5, 2.0);<br>2.56, 1H, ddd (13.7, 4.9, 2.4)	1, 2, 9<br>1, 2, 3	2, 3, 9, 10<br>2, 3, 5, 9, 10	42.0	2.32, 1H, t (12.0);<br>2.57, 1H, dd (4.8, 12.0)
**2**	69.6	3.75, 1H, dddd (11.2,11.2, 4.7, 4.7)	1, 3		69.3	3.75, 1H, dddd (4.8, 6.0, 10.0, 12.0)
**3**	39.7	1.5, 1H, m;<br>1.85, 1H, m	3, 2, 4,<br>3, 2, 4, 1	4<br>4	39.5	1.56, 1H, dt (10.0, 12.0);<br>2.05, 1H, ddd (4.0, 6.0, 12.0)
**4**	41.4	1.5, 1H, m	14	6	40.6	1.50, 1H, ddq (4.0, 7.0, 12.0)
**5**	38.7				38.5	
**6**	40.8	1.8, 1H, m;<br>2.01, dd (13.1, 4.5)	6, 7<br>6, 7	5, 7, 8, 15<br>5, 7, 8, 10, 15	41.2	1.83, 1H, dd (12.0, 14.0);<br>2.00, 1H, dd (4.4, 12.0)
**7**	51.0	3.14, 1H, dd (14.5, 4.5)	6	6, 8, 11, 12, 13	50.8	3.15, 1H, dd (4.4, 14.0)
**8**	198.9				198.9	
**9**	125.9	5.79, 1H, d (2.0)	1	1, 5, 7	125.6	5.77, s
**10**	166.6				166.7	
**11**	143.6				143.4	
**12**	114.5	4.82, 1H, m;<br>4.98, 1H, m	12, 13<br>12, 13	7, 8, 11, 13<br>7, 8, 11, 13	114.2	4.80, 1H, s<br>4.97, 1H, s
**13**	20.2	1.74, 3H, m	12		20.0	1.72, 3H, s
**14**	15.0	0.96, 3H, d (6.2)	4	7, 11, 12	14.7	0.95, 3H, d (7.0)
**15**	16.1	1.16, 3H, s		4, 5, 6, 10	15.9	1.16, 3H, s

Table S11 Summarized NMR signals for ^{13}C, ^{1}H, H-^{1}H COSY, HMBC for **7** recorded in $CDCl_3$. Literature [4] data was measured in $CDCl_3$

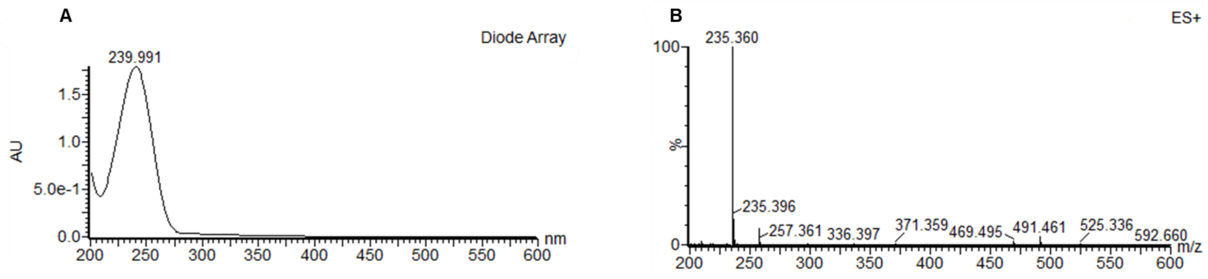

Figure S21 UV-absorption **(A)** and fragmentation pattern **(B)** of **7** in ES$^+$ TIC by LR-LCMS

Elemental Composition Report

Page 1

Single Mass Analysis

Tolerance = 20.0 PPM / DBE: min = -1.5, max = 50.0
Element prediction: Off
Number of isotope peaks used for i-FIT = 3

Monoisotopic Mass, Odd and Even Electron Ions
327 formula(e) evaluated with 5 results within limits (up to 30 closest results for each mass)
Elements Used:
C: 0-80 H: 0-110 N: 0-16 O: 0-12

Sun QTof Premier HAB321
YS 022 925 (9.447) AM (Cen,3, 70.00, Ht,10000.0,556.28,0.70,LS 10) 1: TOF MS ES+ 1.57e+002

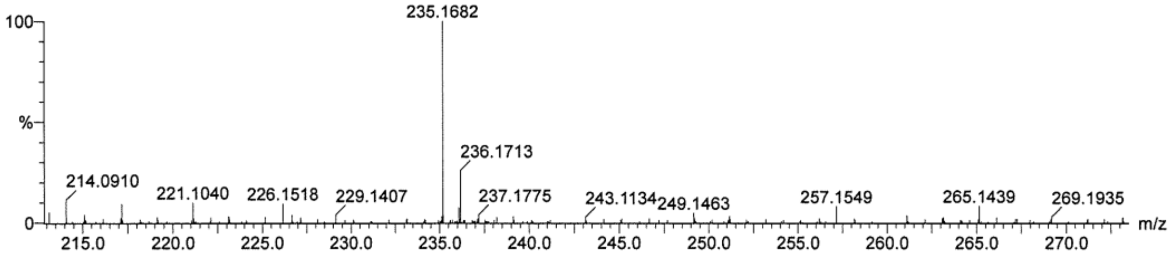

Minimum:				-1.5			
Maximum:		5.0	20.0	50.0			
Mass	Calc. Mass	mDa	PPM	DBE	i-FIT	i-FIT (Norm)	Formula
235.1682	235.1685	-0.3	-1.3	5.0	53.5	1.0	C13 H21 N3 O
	235.1671	1.1	4.7	5.5	54.4	1.9	C11 H19 N6
	235.1698	-1.6	-6.8	4.5	53.4	0.9	C15 H23 O2
	235.1658	2.4	10.2	0.5	55.5	2.9	C10 H23 N2 O4
	235.1644	3.8	16.2	1.0	57.1	4.6	C8 H21 N5 O3

Figure S22 HRMS data for **7**. *m/z* $(M+H)^+$ calc. mass is 235.1698, 235.1682 was found.

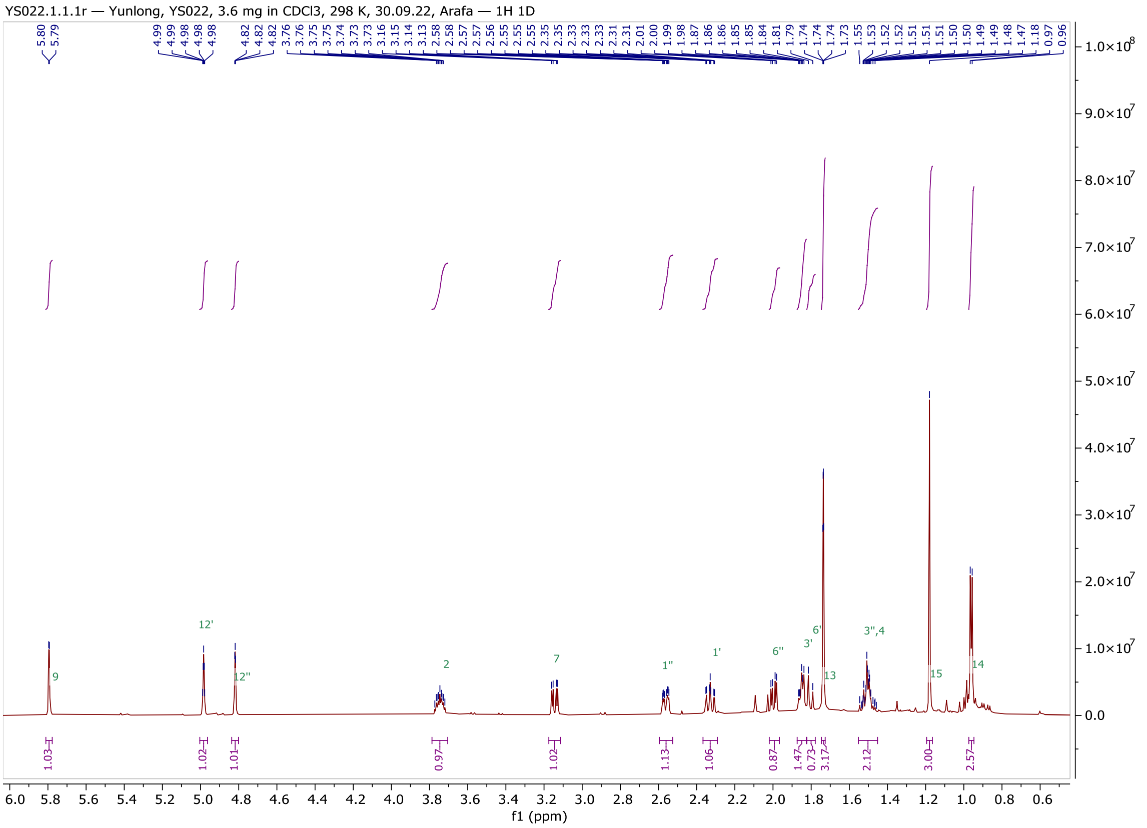

Figure S23 ^{1}H-NMR of **7** recorded at 600 MHz in $CDCl_3$

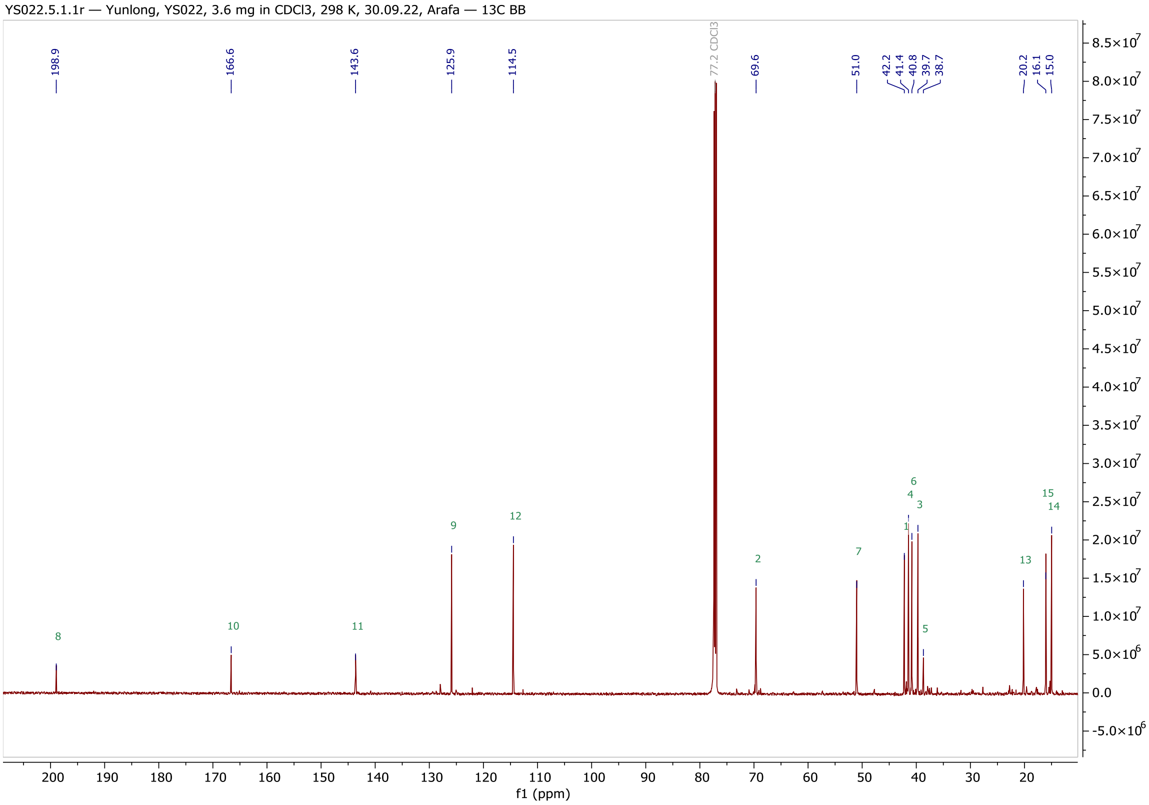

Figure S24 ^{13}C-NMR of **7** recorded at 150 MHz in $CDCl_3$

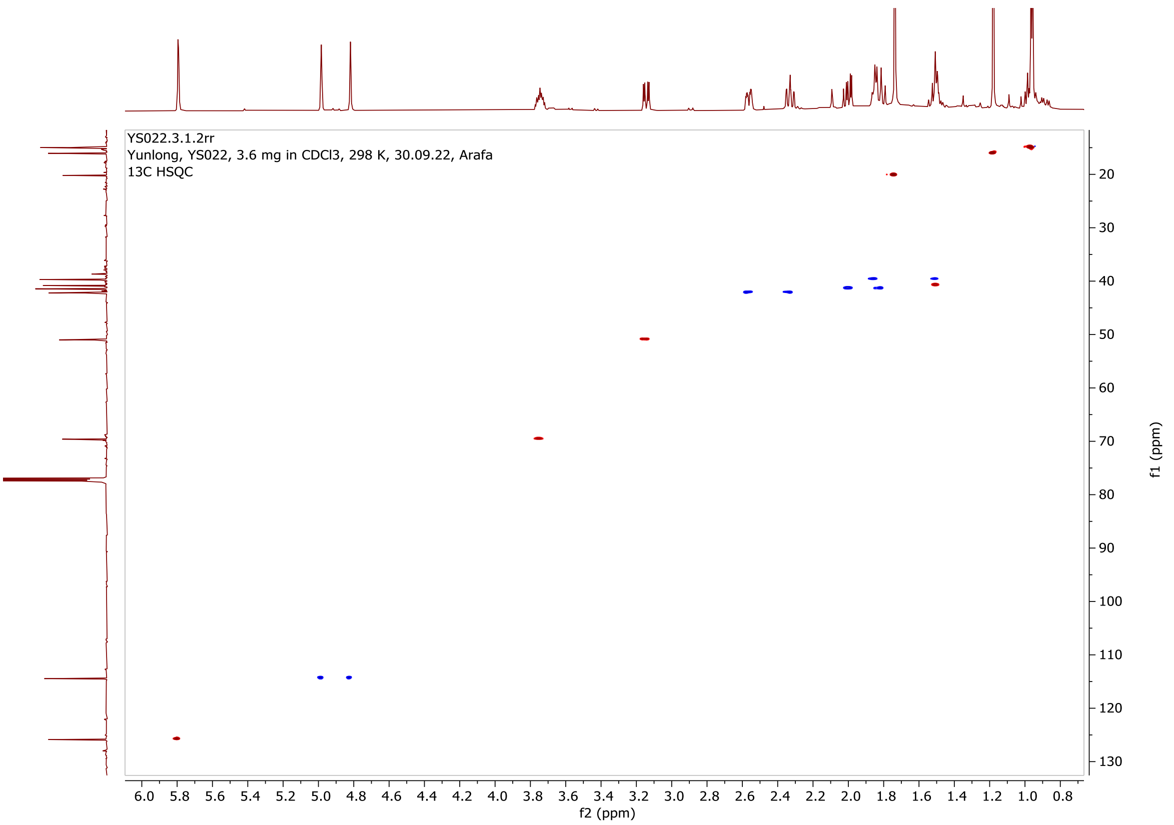

Figure S25 HSQC-spectrum of **7** recorded at 600, 150 MHz in $CDCl_3$

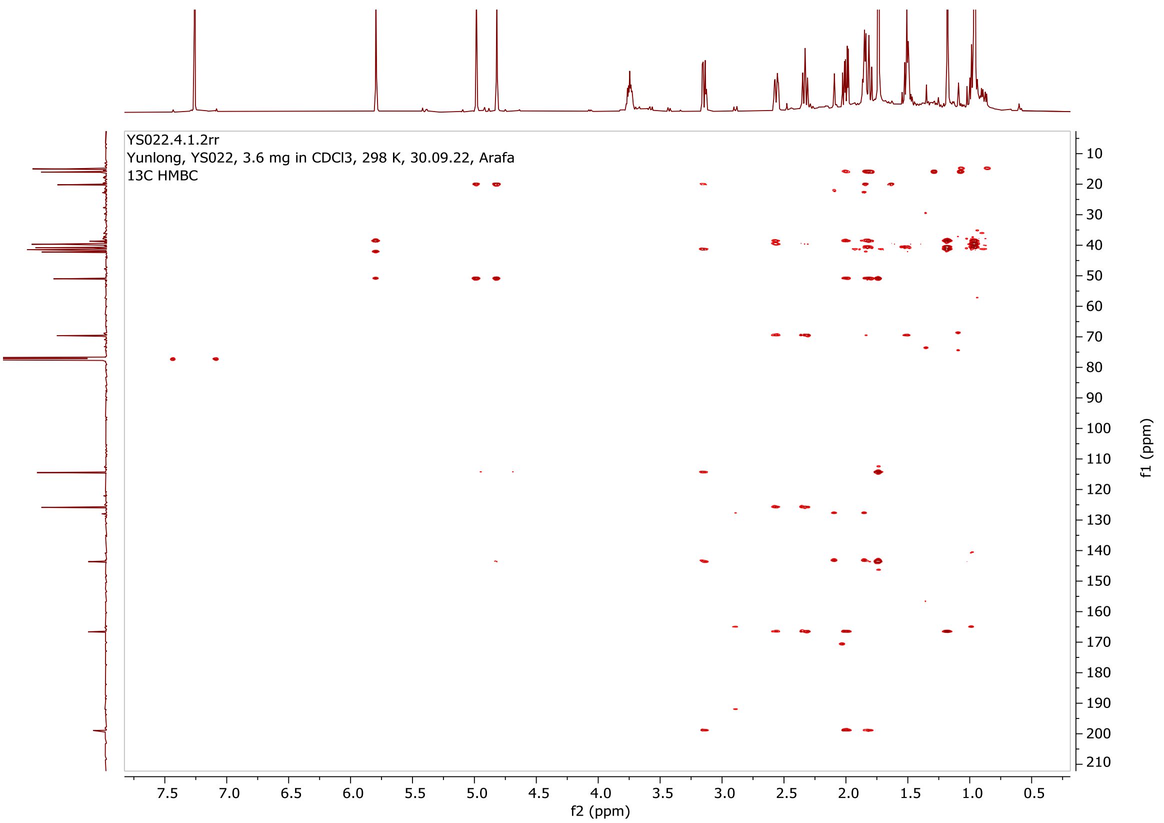

Figure S26 HMBC-spectrum of **7** recorded at 600, 150 MHz in $CDCl_3$

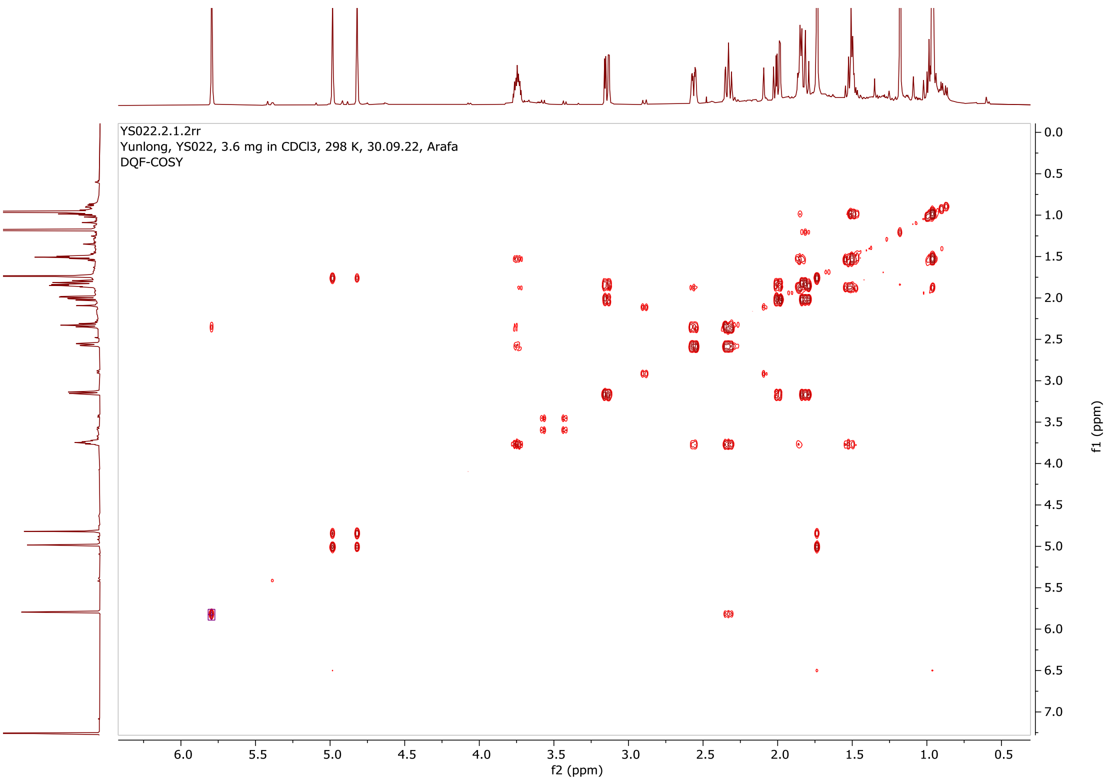

Figure S27 ^{1}H, ^{1}H-COSY -spectrum of **7** recorded at 600 MHz in $CDCl_3$

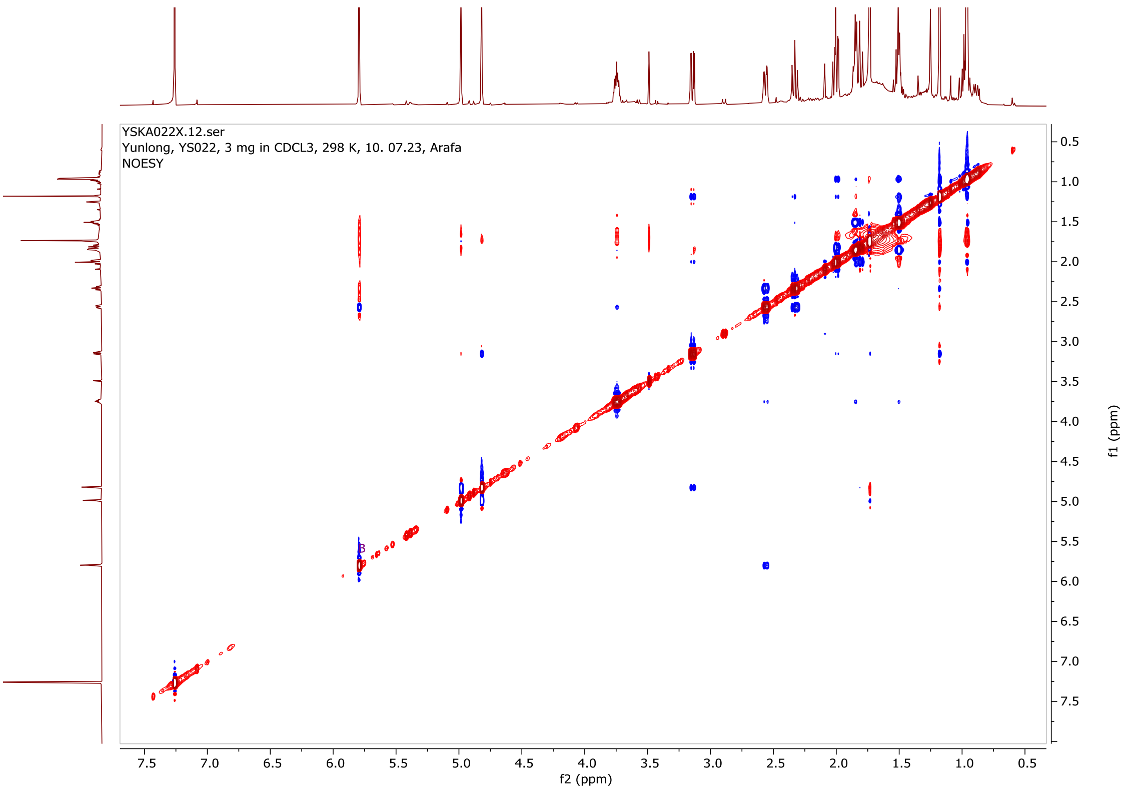

Figure S28 NOESY-spectrum of **7** recorded at 600 MHz in $CDCl_3$

Compound 8

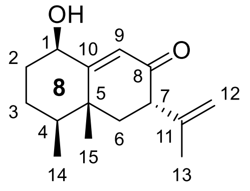

Chemical Formula: $C_{15}H_{22}O_2$
Exact Mass: 234.1620

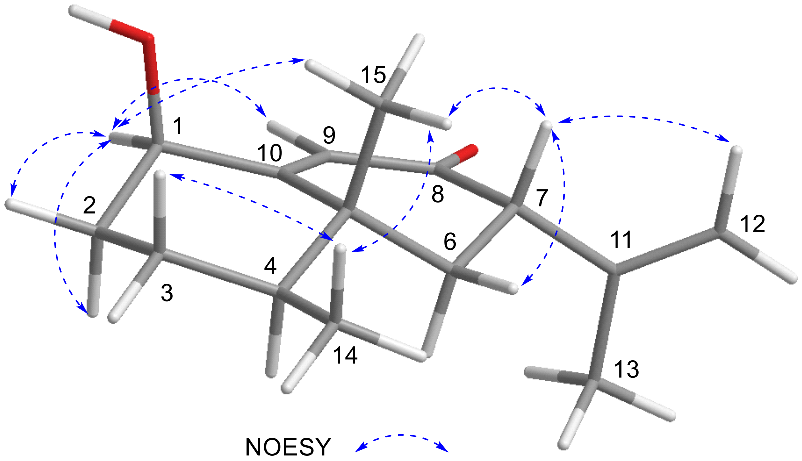

Compound 8				
**Pos.**	***δ*** δ_C **/ ppm**	δ_H **/ ppm (*J*/Hz)**	**^{1}H-^{1}H COSY**	**HMBC (H-C)**
**1**	73.2	4.33, 1H, dd (3.0, 3.0)	2	3, 5, 9
**2**	33.0	1.69, 1H, m;<br>2.01, 1H, m	2, 1, 3<br>2, 1, 3	3, 4<br>1, 3, 4, 10
**3**	24.9	1.41, 1H, m;<br>1.88, 1H, m	3, 2, 4<br>3, 2, 4	2, 4, 5<br>2, 4, 5
**4**	43.5	1.46, 1H, m	3, 14	14
**5**	39.1			
**6**	43.1	1.89, 1H, m;<br>1.95, 1H, m	6, 7<br>6, 7	5, 7, 8, 10<br>5, 7, 8, 10
**7**	51.6	3.24, 1H, dd (14.3, 4.6)	6	6, 8, 11, 12, 13
**8**	200.0			
**9**	126.7	5.84, 1H, s		1, 5, 7
**10**	167.5			
**11**	143.6			
**12**	114.5	4.82, 1H, m;<br>4.98, 1H, m	12, 13<br>12, 13	7, 13<br>7, 13
**13**	20.1	1.72, 3H, dd (1.5, 0.8)	12	7, 11, 12
**14**	15.3	0.95, 3H, d (6.8)	4	3, 4 , 5
**15**	18.4	1.37, 3H, s		4, 5, 6, 10

Table S12 Summarized NMR signals for ^{13}C, ^{1}H, ^{1}H-^{1}H COSY, HMBC for **8** recorded in $CDCl_3$

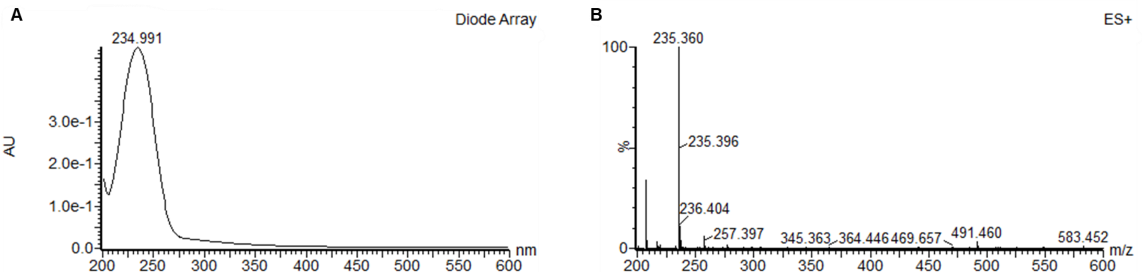

Figure S29 UV-absorption **(A)** and fragmentation pattern **(B)** of **8** in ES^+ TIC by LR-LCMS

Elemental Composition Report **Page 1**

Single Mass Analysis

Tolerance = 20.0 PPM / DBE: min = -1.5, max = 50.0
Element prediction: Off
Number of isotope peaks used for i-FIT = 3

Monoisotopic Mass, Odd and Even Electron Ions
210 formula(e) evaluated with 3 results within limits (all results (up to 1000) for each mass)
Elements Used:
C: 0-40 H: 0-60 N: 0-5 O: 0-5 Na: 0-1
Sun QTof Premier HAB321
YS 055 754 (7.712) AM (Cen,4, 70.00, Ht,10000.0,556.28,0.70,LS 10) 1: TOF MS ES+
1.72e+002

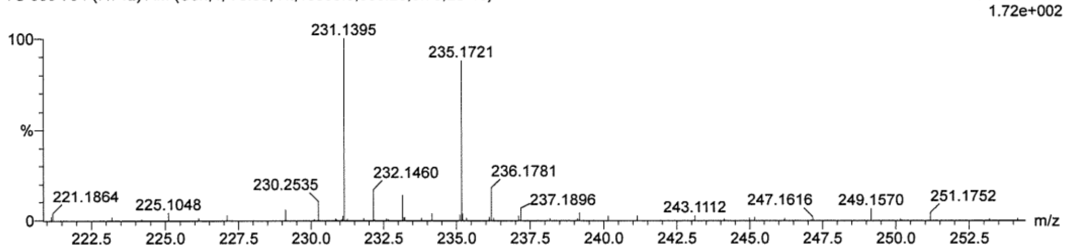

Minimum:				-1.5			
Maximum:		5.0	20.0	50.0			
Mass	Calc. Mass	mDa	PPM	DBE	i-FIT	i-FIT (Norm)	Formula
235.1721	235.1698	2.3	9.8	4.5	23.3	0.5	C15 H23 O2
	235.1685	3.6	15.3	5.0	24.4	1.5	C13 H21 N3 O
	235.1674	4.7	20.0	1.5	24.8	1.9	C13 H24 O2 Na

Figure S30 HRMS data for **8**; *m/z* $(M+H)^+$ calc. mass is 235.1698, 235.1721 was found

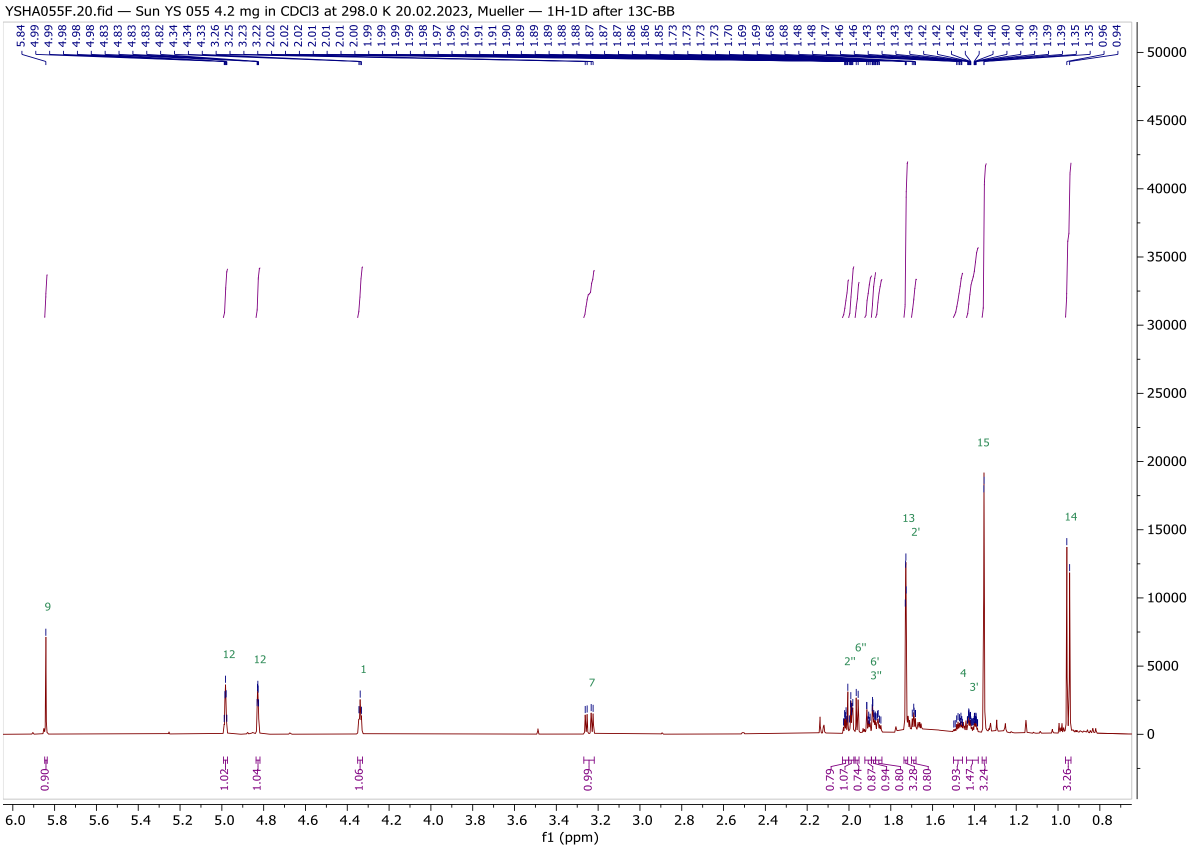

Figure S31 ^{1}H-NMR of **8** recorded at 500 MHz in $CDCl_3$

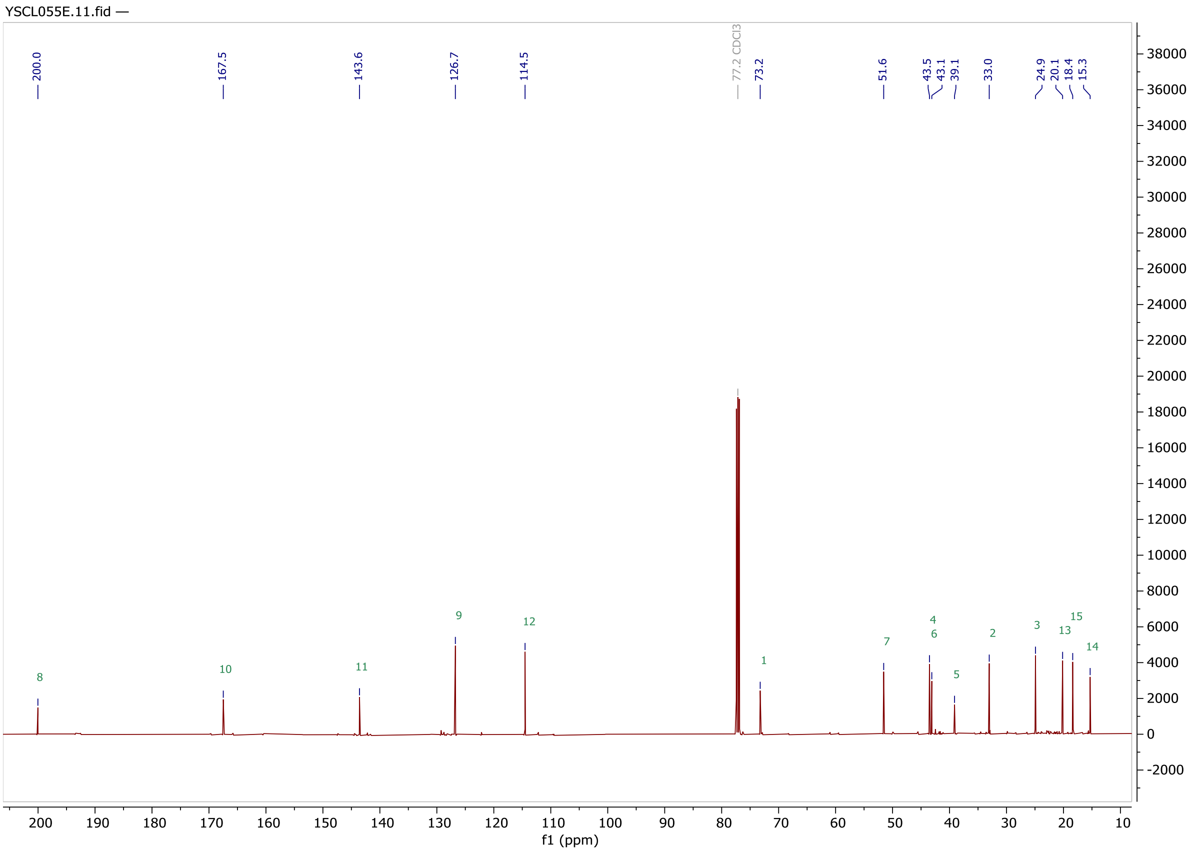

Figure S32 ^{13}C-NMR of **8** recorded at 125 MHz in $CDCl_3$

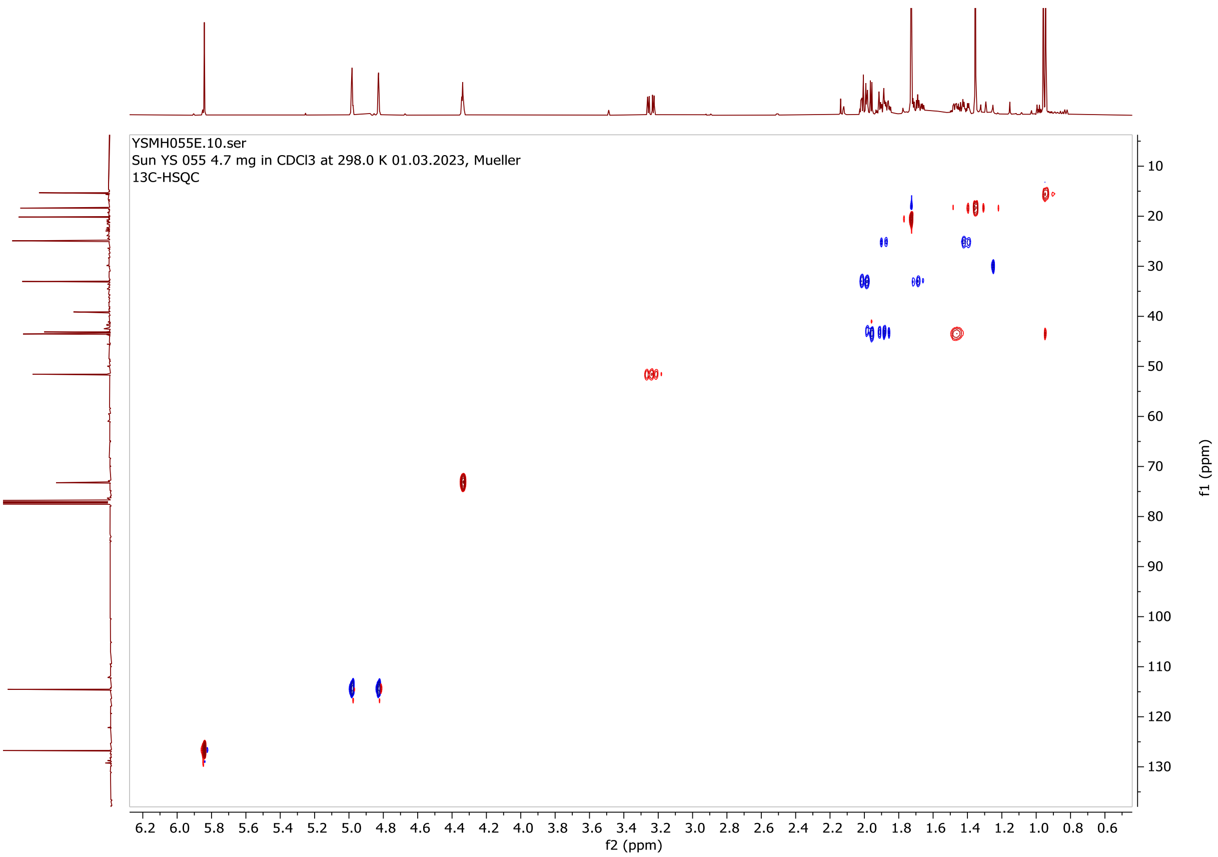

Figure S33 HSQC-spectrum of **8** recorded at 500, 125 MHz in $CDCl_3$

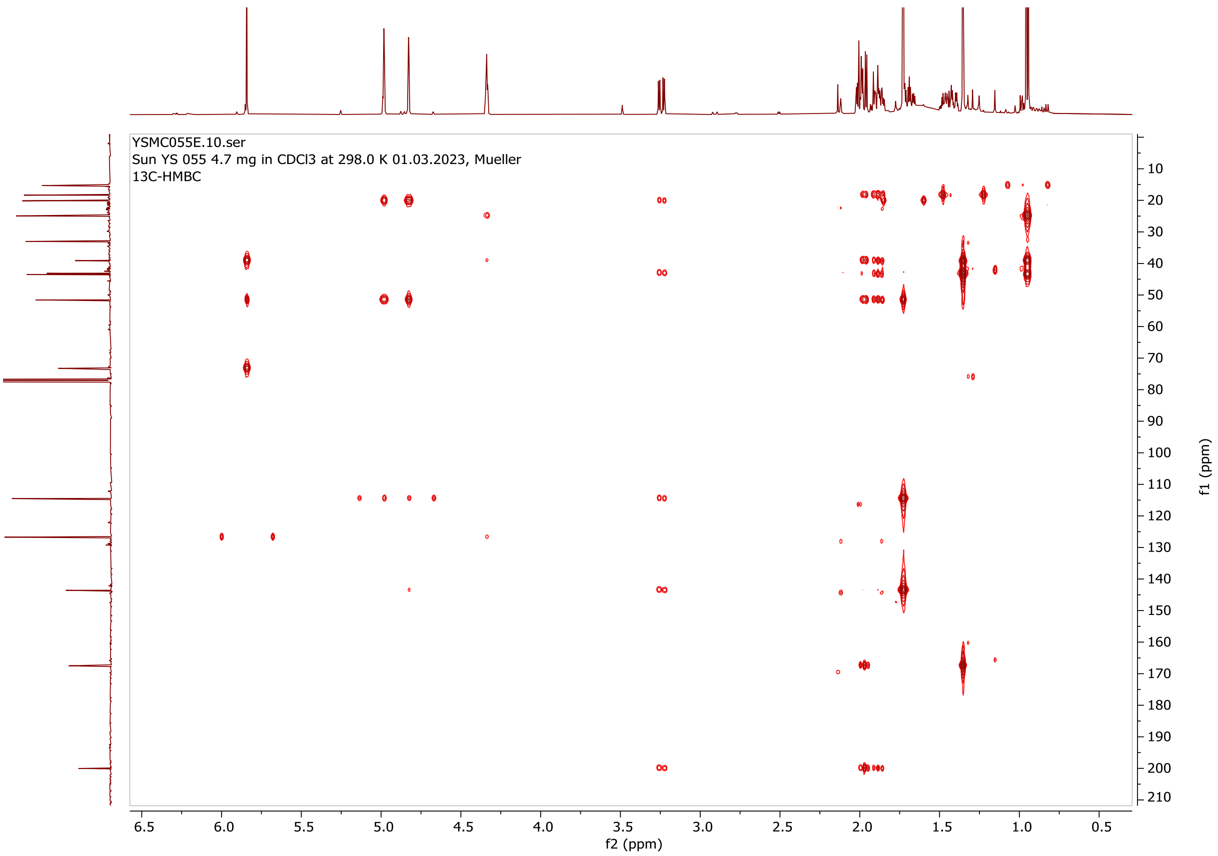

Figure S34 HMBC-spectrum of **8** recorded at 500, 125 MHz in $CDCl_3$

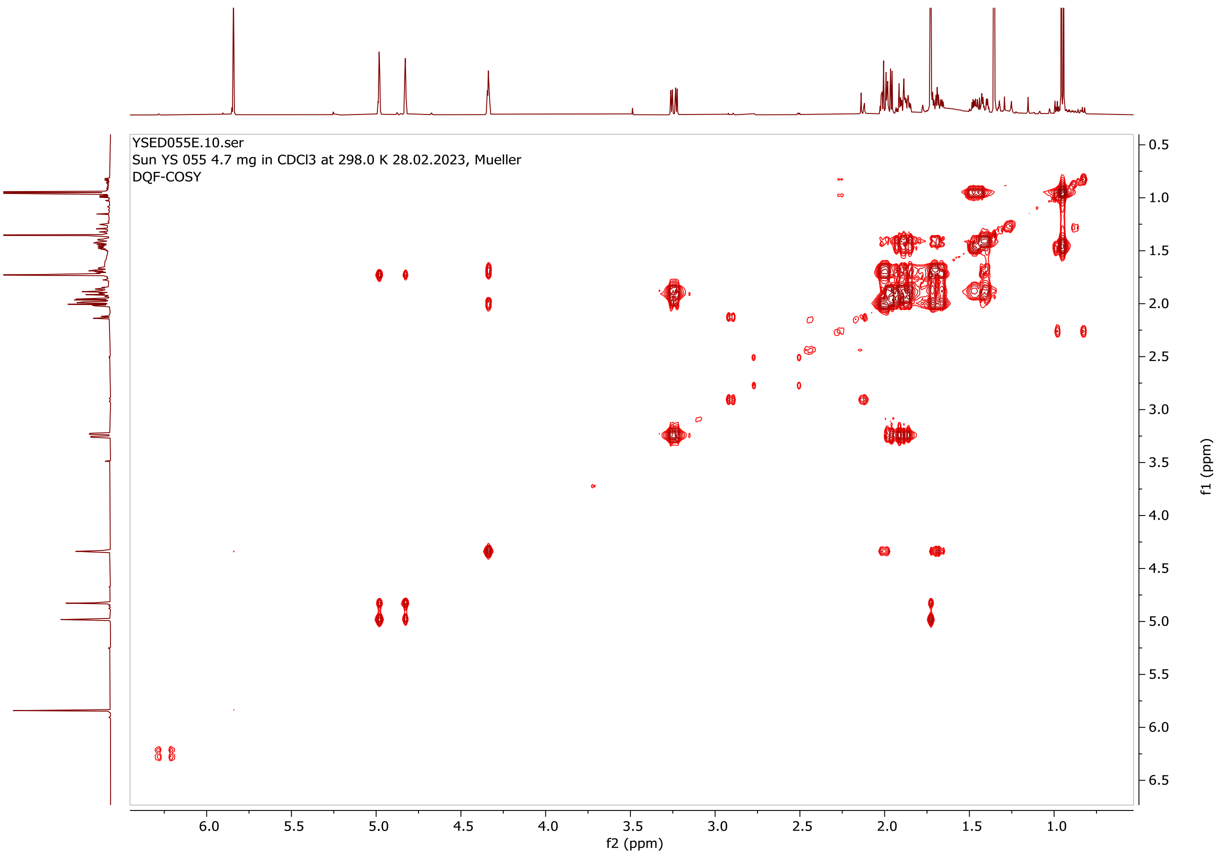

Figure S35 ^{1}H, ^{1}H-COSY-spectrum of **8** recorded at 500 MHz in $CDCl_3$

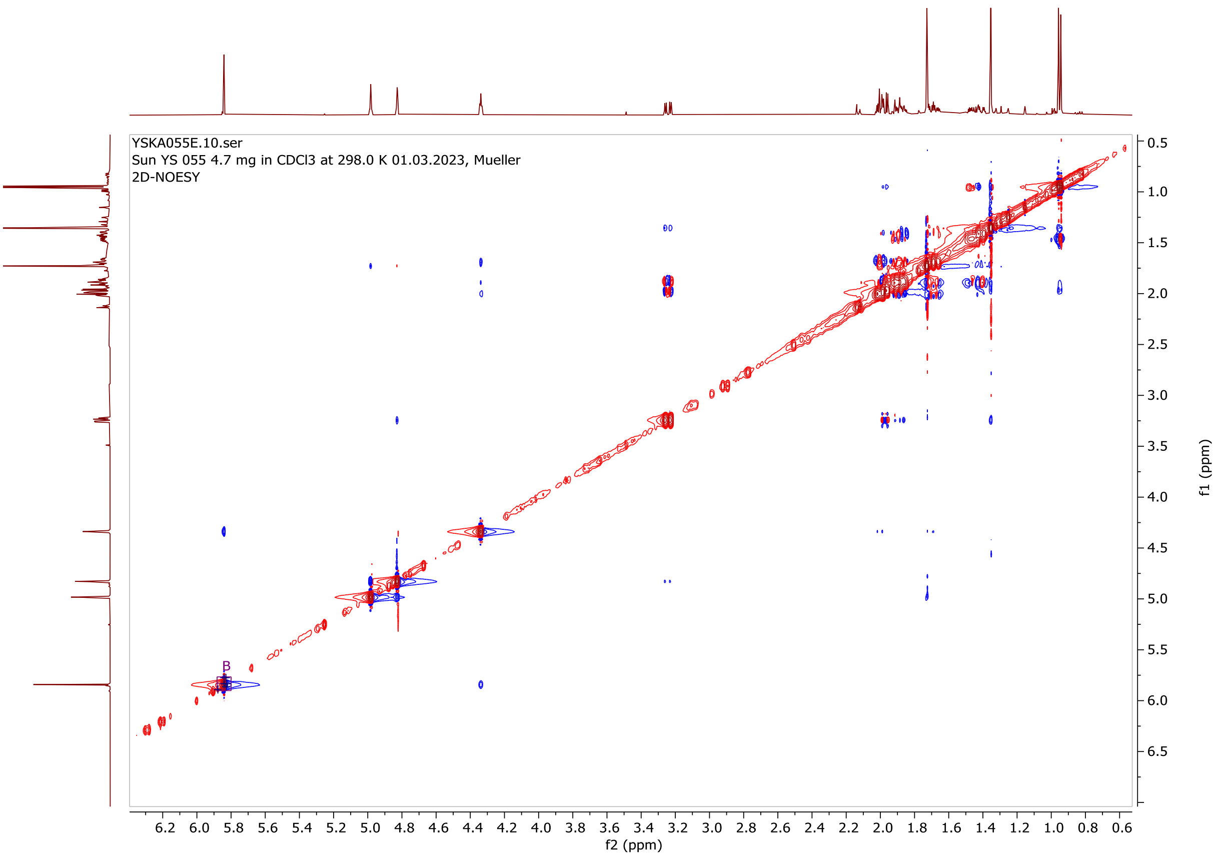

Figure S36 NOESY-spectrum of **8** recorded at 500 MHz in $CDCl_3$

Compound 9

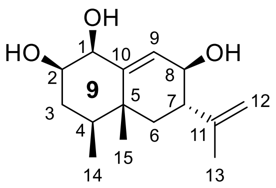

Chemical Formula: $C_{15}H_{24}O_3$
Exact Mass: 252.1725

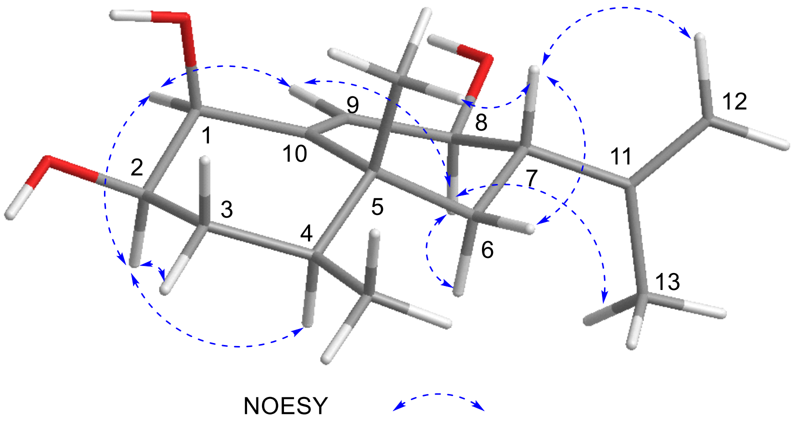

Compound 9				
**Pos.**	**δ_C / ppm**	**δ_H / ppm (J/Hz)**	**^{1}H-^{1}H COSY**	**HMBC (H-C)**
**1**	77.7	4.08, 1H, dd (3.4, 1.2)	2, 3	2, 3, 5, 9
**2**	73.2	3.51, 1H, ddd (11.8, 4.5, 3.4)	1, 3	1, 3
**3**	34.7	1.46, 1H, dddd (12.8, 4.5, 3.2, 1.2);<br>1.81, 1H, ddd (12.7, 12.7, 12.7)	3, 4, 2, 1<br>3, 4, 2	1, 2, 4, 5, 14<br>1, 2, 4, 5, 14
**4**	42.8	1.3, 1H, m	3, 14	3, 5, 14
**5**	38.6			
**6**	44.9	1.33, 1H, m;<br>1.59, 1H, dd (13.0, 2.6)	6, 7<br>6, 7	5, 7, 8, 10, 15<br>5, 7, 8, 10, 15
**7**	47.9	2.34, 1H, ddd (12.7, 9.7, 2.6)	6, 8	6, 8, 11, 12, 13
**8**	70.6	4.04, 1H, dd (9.7, 1.9)	7, 9	7, 9, 10, 11, 13
**9**	132.0	5.58, 1H, d (1.9)	8	1, 5, 7, 10, 11
**10**	146.2			
**11**	148.3			
**12**	111.9	4.83, 2H, m	13	7, 11, 13
**13**	20.1	1.74, 3H, m	12	7, 11, 12
**14**	15.2	0.85, 3H, d (6.9)	4	3, 4, 5
**15**	20.5	1.18, 3H, s		4, 5, 6, 10

Table S13 Summarized NMR signals for ^{13}C, ^{1}H, ^{1}H-^{1}H COSY, HMBC for **9** recorded in CD_3OD

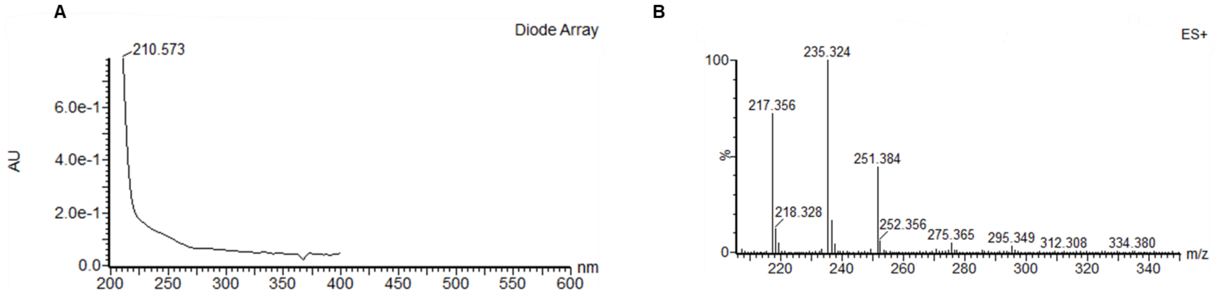

Figure S37 UV-absorption **(A)** and fragmentation pattern **(B)** of **9** in ES$^+$ TIC by LR-LCMS

Elemental Composition Report **Page 1**

Single Mass Analysis

Tolerance = 20.0 PPM / DBE: min = -1.5, max = 50.0
Element prediction: Off
Number of isotope peaks used for i-FIT = 3

Monoisotopic Mass, Even Electron Ions
28 formula(e) evaluated with 1 results within limits (all results (up to 1000) for each mass)
Elements Used:
C: 0-90 H: 0-50 O: 0-8

Sun QTof Premier HAB321
YS 056 447 (4.562) AM (Cen,4, 70.00, Ht,10000.0,556.28,0.70,LS 10) 1: TOF MS ES+ 1.34e+002

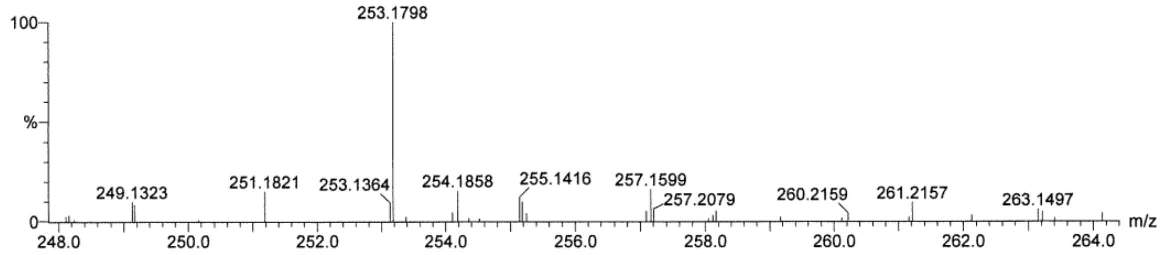

Minimum:				-1.5			
Maximum:		5.0	20.0	50.0			
Mass	Calc. Mass	mDa	PPM	DBE	i-FIT	i-FIT (Norm)	Formula
253.1798	253.1804	-0.6	-2.4	3.5	27.2	0.0	C15 H25 O3

Figure S38 HRMS data for **9**; *m/z* (M+H)$^+$ calc. mass is 253.1804, 253.1798 was found

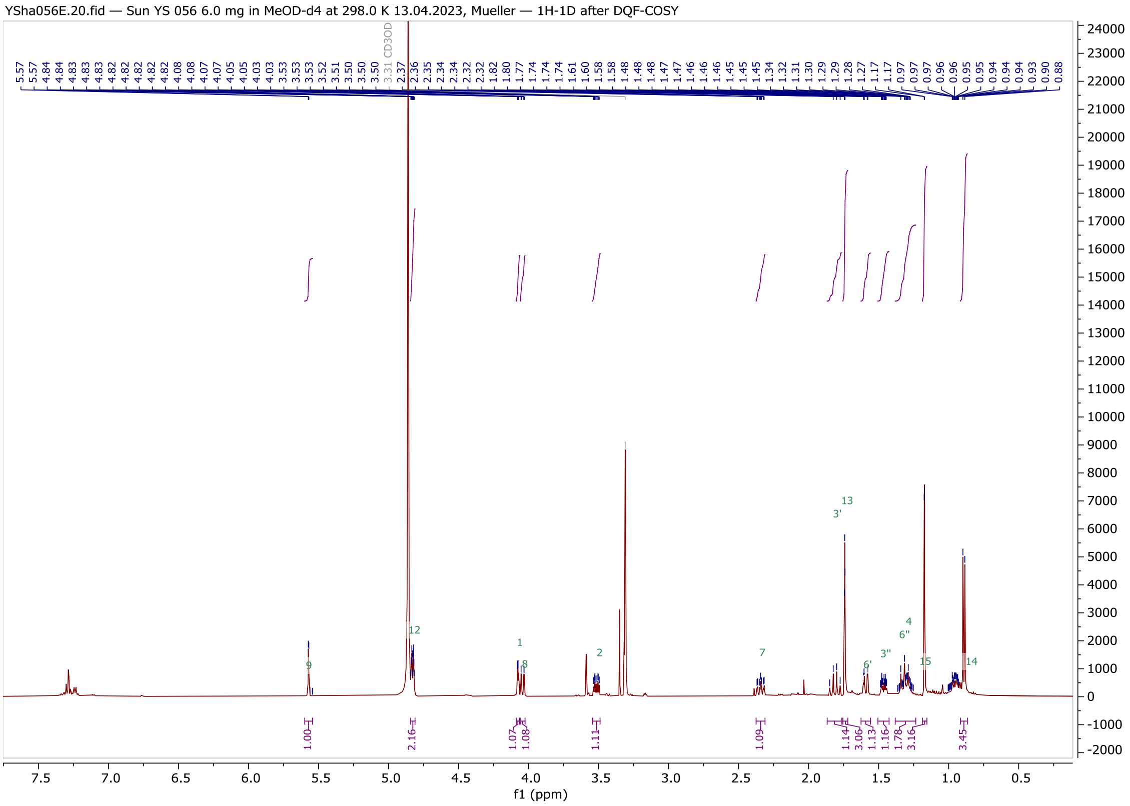

Figure S39 ^{1}H-NMR of **9** recorded at 500 MHz in CD_3OD

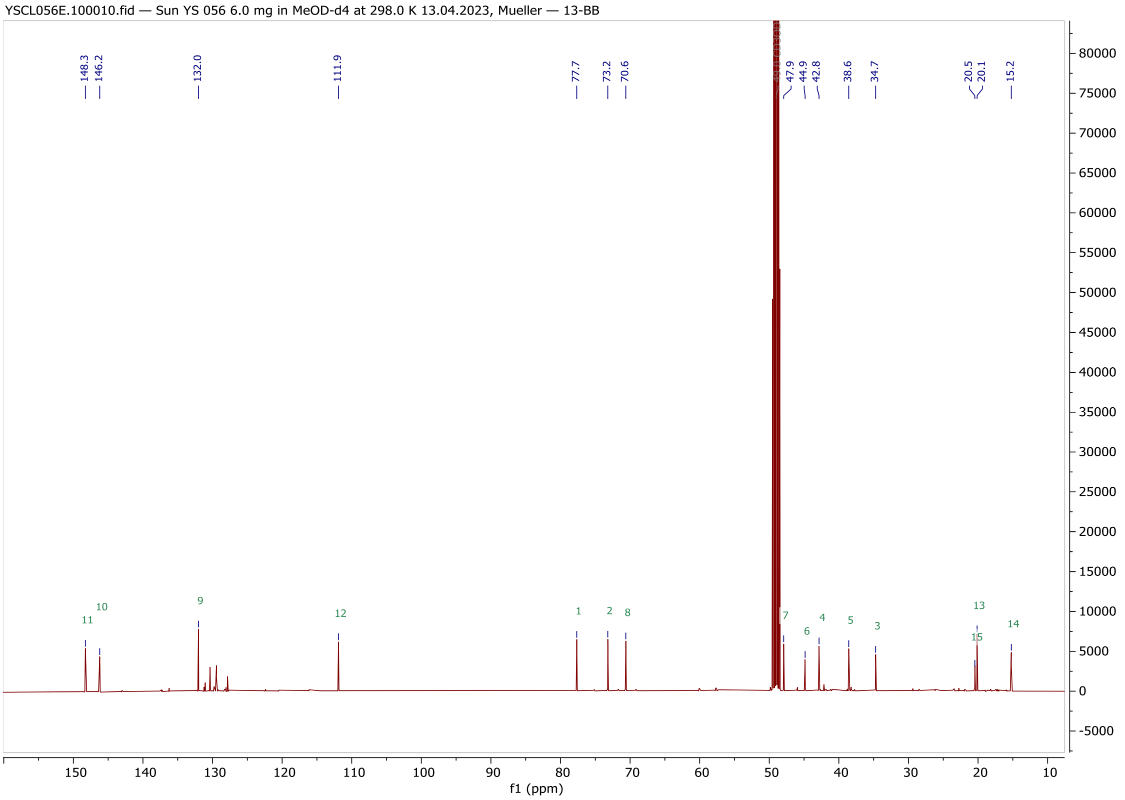

Figure S40 ^{13}C-NMR of **9** recorded at 125 MHz in CD_3OD

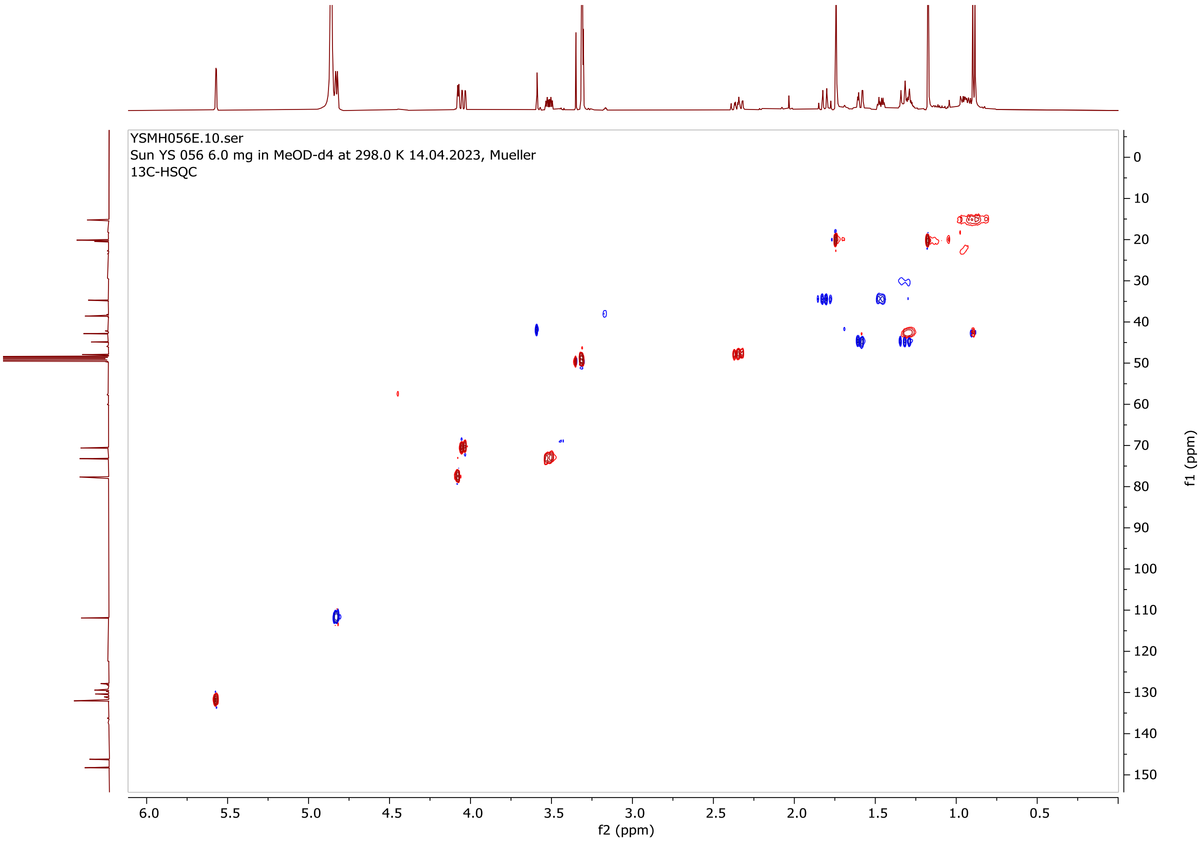

Figure S41 HSQC-spectrum of **9** recorded at 500, 125 MHz in CD_3OD

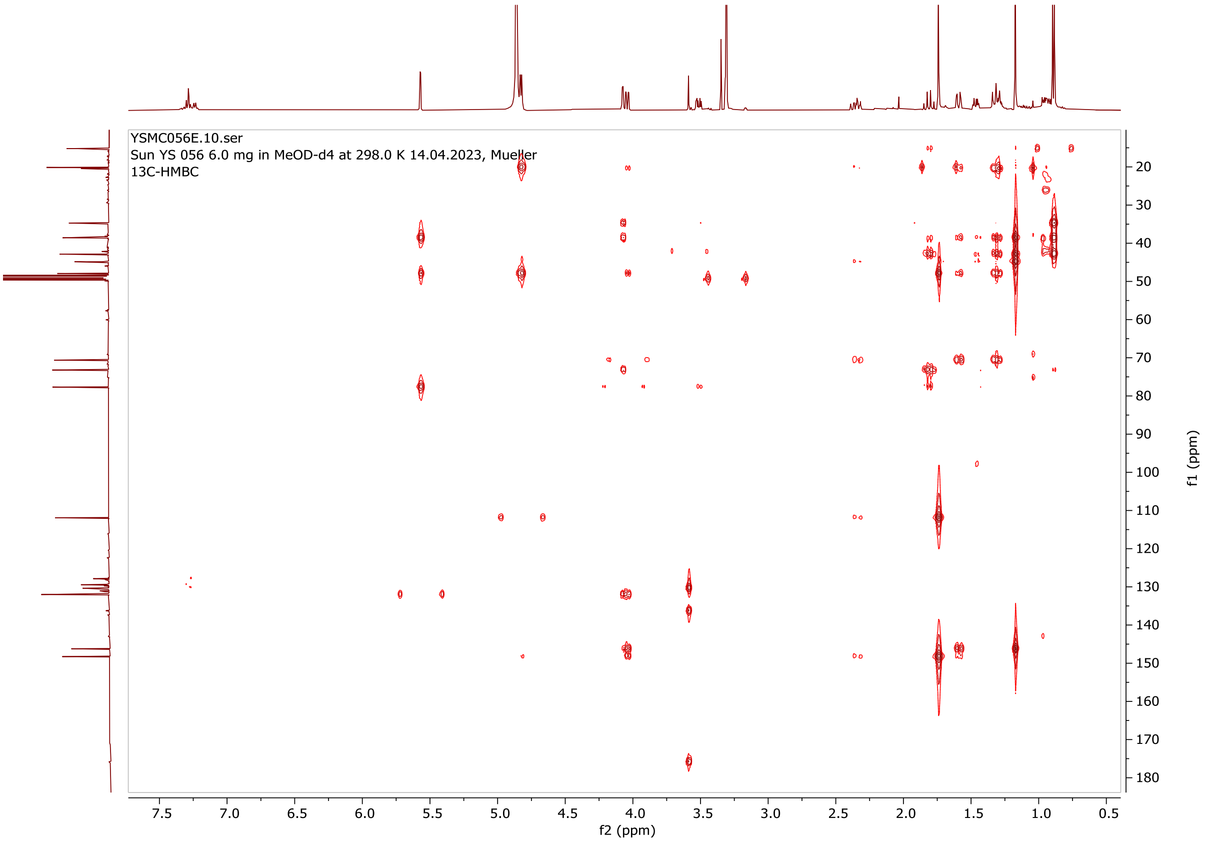

Figure S42 HMBC-spectrum of **9** recorded at 500, 125 MHz in CD_3OD

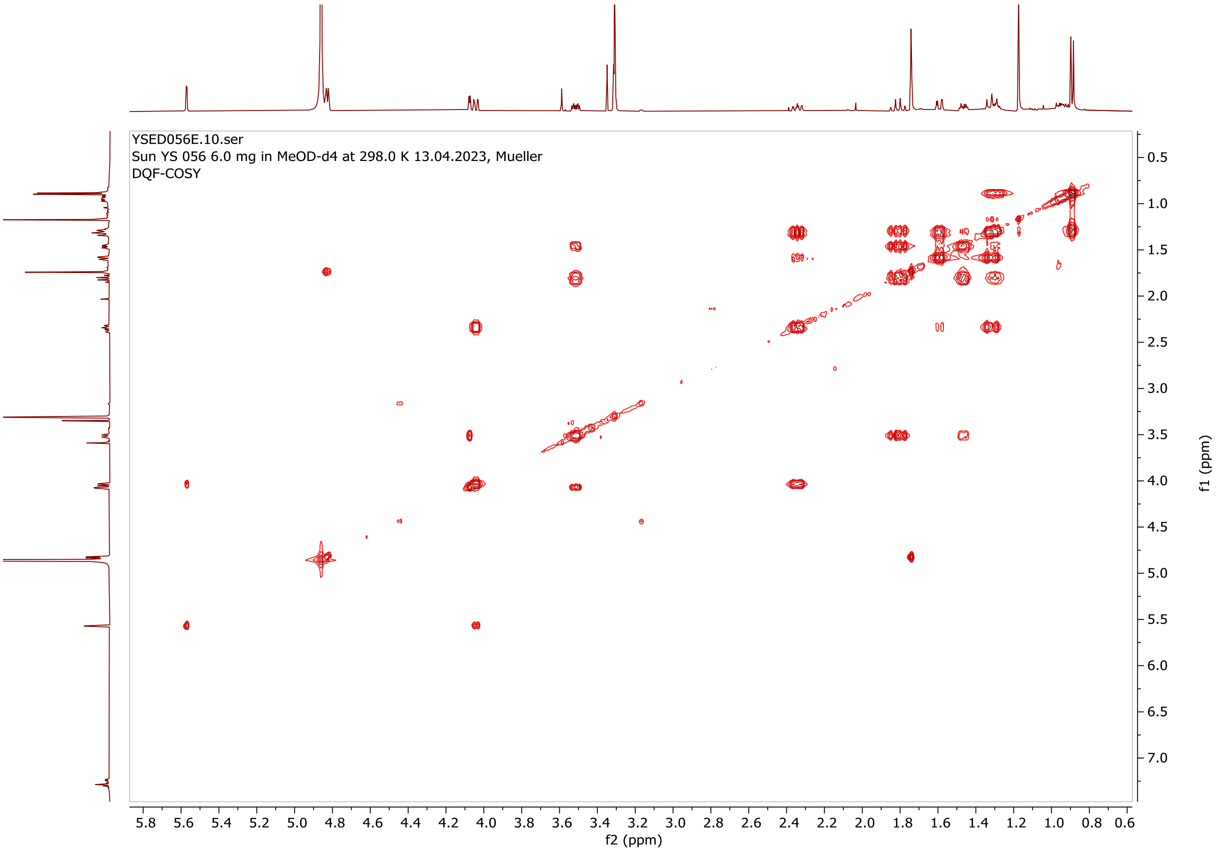

Figure S43 ^{1}H, ^{1}H-COSY-spectrum of **9** recorded at 500 MHz in CD_3OD

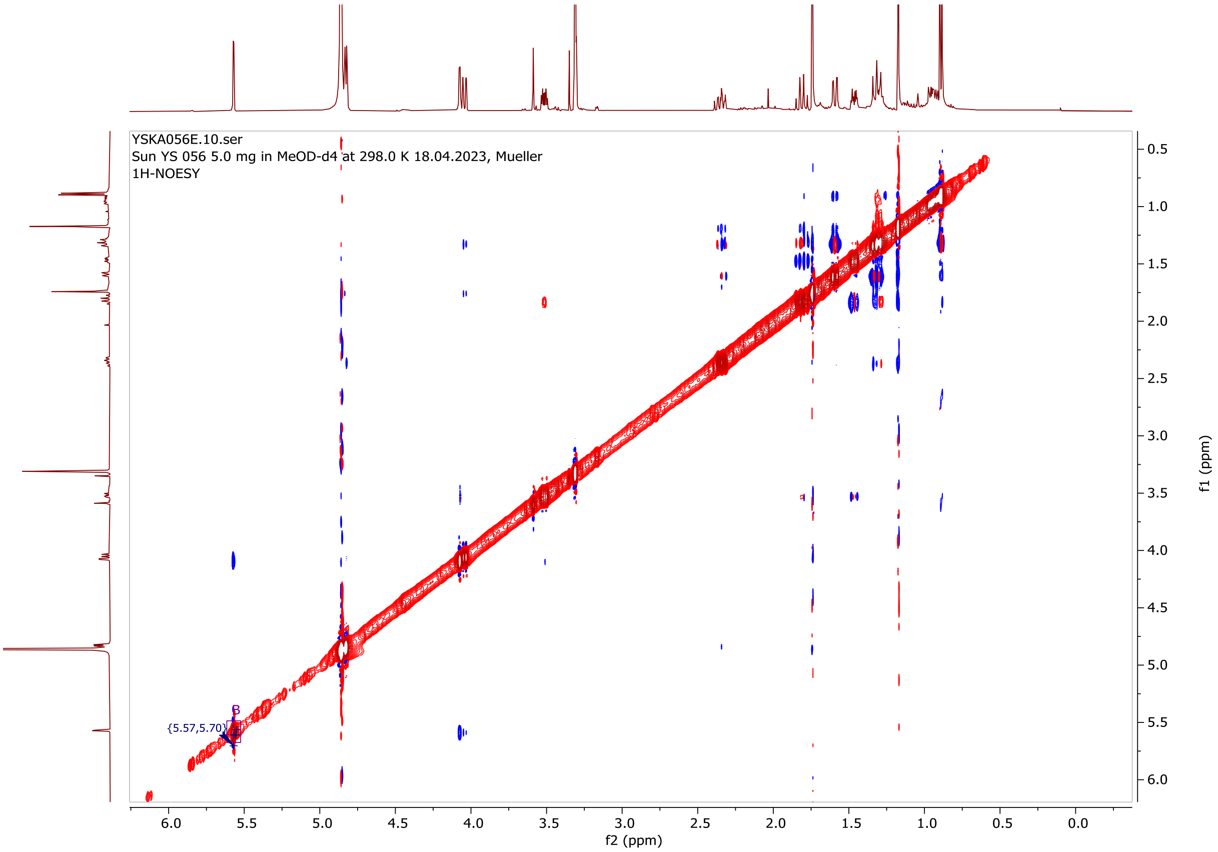

Figure S44 NOESY -spectrum of **9** recorded at 500 MHz in CD_3OD

Compound 10

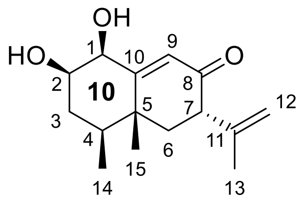

Chemical Formula: $C_{15}H_{22}O_3$
Exact Mass: 250.1569

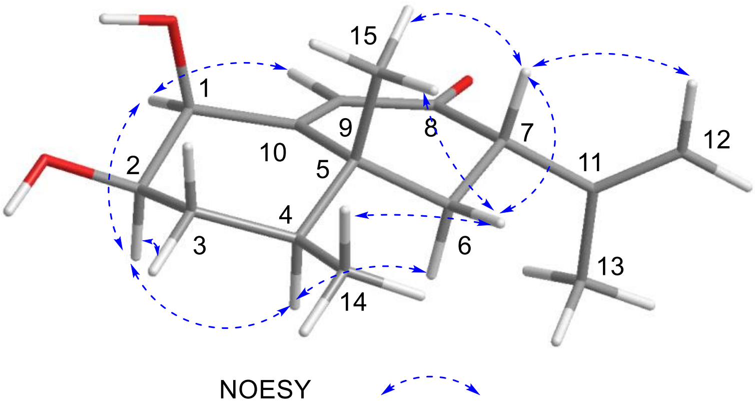

Compound 10				
**Pos.**	**δ_C / ppm**	**δ_H / ppm (*J*/Hz)**	**^{1}H-^{1}H COSY**	**HMBC (H-C)**
**1**	75.8	4.28, 1H, d (3.2)	2, 3	2, 3, 5, 9, 10
**2**	71.0	3.73, 1H, ddd (11.7, 4.7, 3.5)	3, 1	1, 3, 4
**3**	33.6	1.62, 1H, dddd (12.9, 4.6, 3.2, 1.2);<br>1.85, 1H, m	3, 4, 2, 1<br>3, 4, 2	1, 2, 4, 5<br>1, 2, 4, 5
**4**	40.6	1.48, 1H, m	3, 14	3, 4, 5
**5**	38.2			
**6**	43.0	1.85, 1H, m;<br>1.97, 1H, dd (13.0, 4.4)	6, 7<br>6, 7	5, 7, 8, 10, 15<br>5, 7, 8, 10, 15
**7**	51.4	3.24, 1H, dd (14.4, 4.4)	6	5, 6, 8, 11, 12, 13
**8**	199.6			
**9**	128.9	5.92, 1H, s		1, 5, 7, 8, 10
**10**	165.0			
**11**	143.3			
**12**	114.6	4.82, 1H, m;<br>4.98, 1H, m	12, 13<br>12, 13	7, 11, 13<br>7, 11, 13
**13**	20.2	1.7, 3H, m	12	7, 11, 12
**14**	14.9	0.96, 3H, d (6.9)	4	3, 4, 5
**15**	18.1	1.32, 3H, s		4, 5, 6

Table S14 Summarized NMR signals for $_{13}$C, ^{1}H, ^{1}H-^{1}H COSY, HMBC for **10** recorded in CDCl$_3$

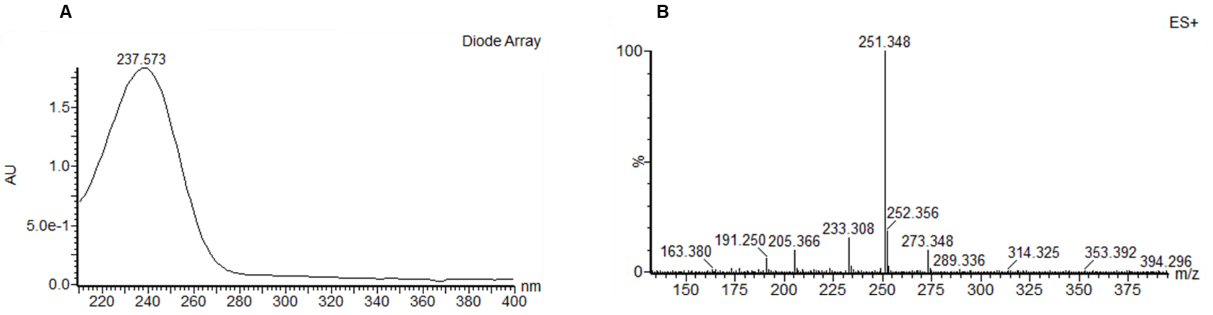

Figure S45 UV-absorption **(A)** and fragmentation pattern **(B)** of **10** in ES^+ TIC by LR-LCMS

Elemental Composition Report **Page 1**

Single Mass Analysis
Tolerance = 20.0 PPM / DBE: min = -1.5, max = 50.0
Element prediction: Off
Number of isotope peaks used for i-FIT = 3

Monoisotopic Mass, Odd and Even Electron Ions
118 formula(e) evaluated with 5 results within limits (all results (up to 1000) for each mass)
Elements Used:
C: 0-40 H: 0-60 N: 0-5 O: 0-5

Sun QTof Premier HAB321
YS 054, neg 585 (5.987) AM (Cen,4, 70.00, Ht,10000.0,554.26,0.70,LS 10) 1: TOF MS ES-
1.60e+002

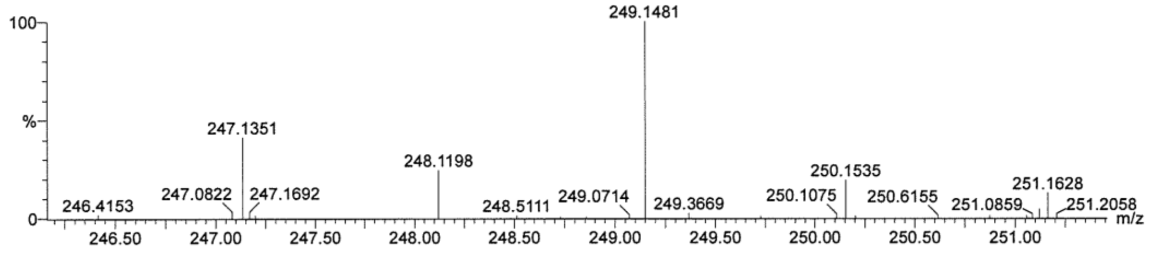

Mass	Calc. Mass	mDa	PPM	DBE	i-FIT	i-FIT (Norm)	Formula
Minimum:				-1.5			
Maximum:		5.0	20.0	50.0			
249.1481	249.1477	0.4	1.6	6.0	36.5	1.6	C13 H19 N3 O2
	249.1491	-1.0	-4.0	5.5	35.8	0.9	C15 H21 O3
	249.1450	3.1	12.4	1.5	37.7	2.8	C10 H21 N2 O5
	249.1517	-3.6	-14.4	10.0	36.1	1.2	C18 H19 N
	249.1437	4.4	17.7	2.0	38.6	3.7	C8 H19 N5 O4

Figure S46 HRMS data for **10**; *m/z* $(M-H)^-$ calc. mass is 249.1491, 249.1481 was found

YSHA054E.50.fid — Sun YS 054 4.7 mg in CDCl3 at 298.0 K 23.02.2023, Mueller — 1H-1D after all

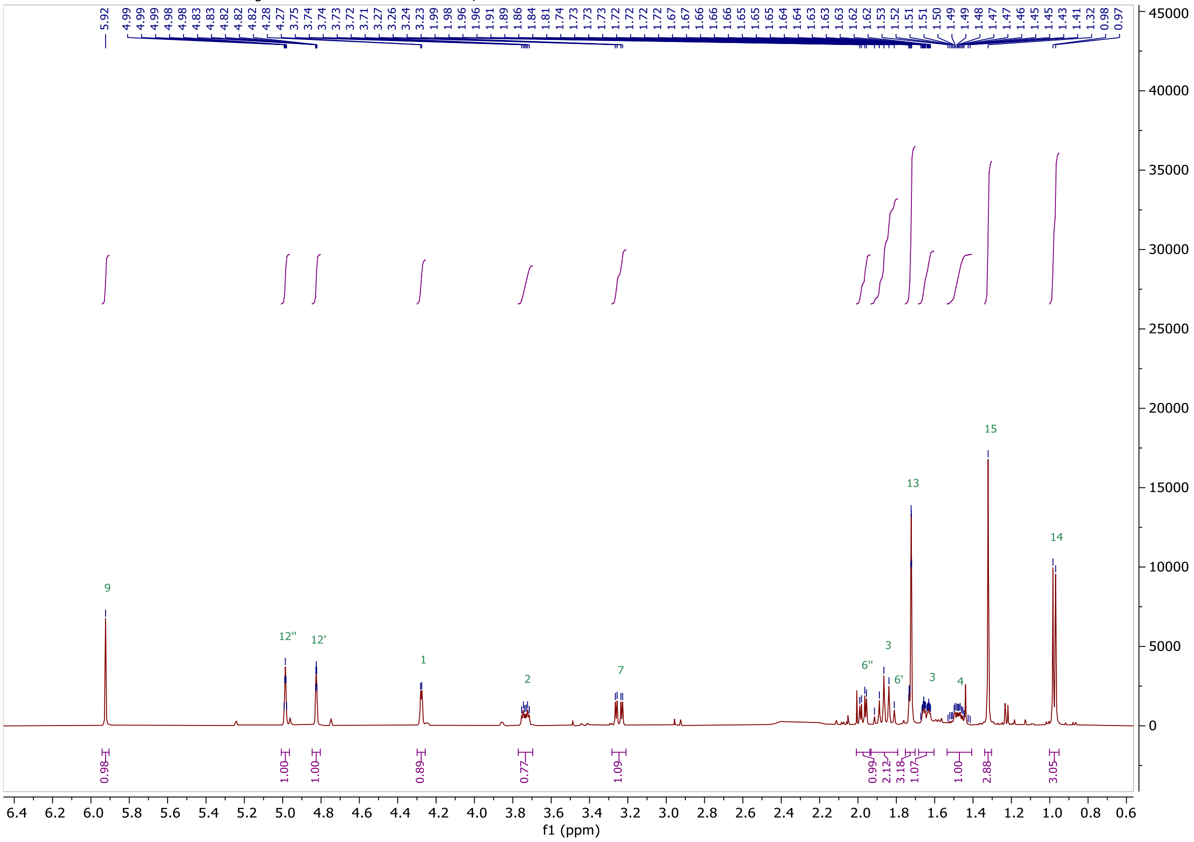

Figure S47 ^{1}H-NMR of **10** recorded at 500 MHz in CD_3OD

YSCL054E.10.fid — Sun YS 054 4.7 mg in CDCl3 at 298.0 K 22.02.2023, Mueller — 13C-BB

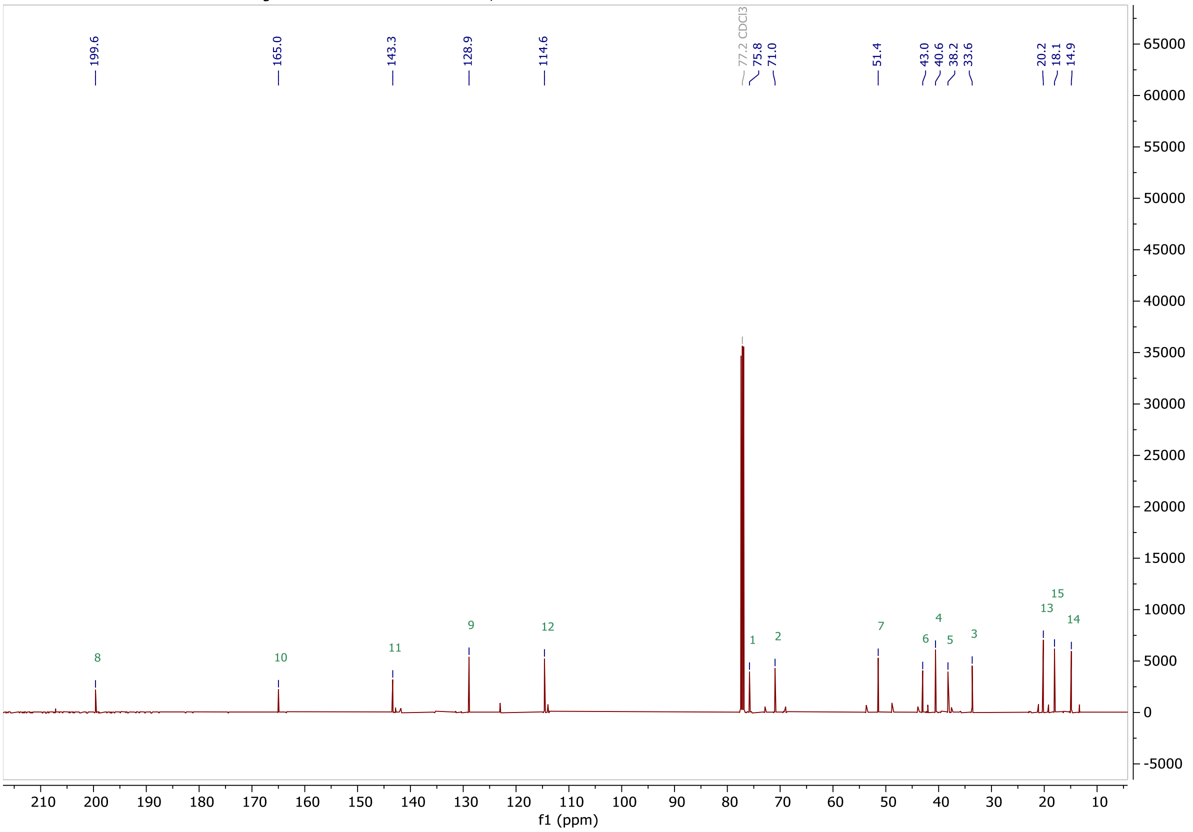

Figure S48 ^{13}C-NMR of **10** recorded at 125 MHz in $CDCl_3$

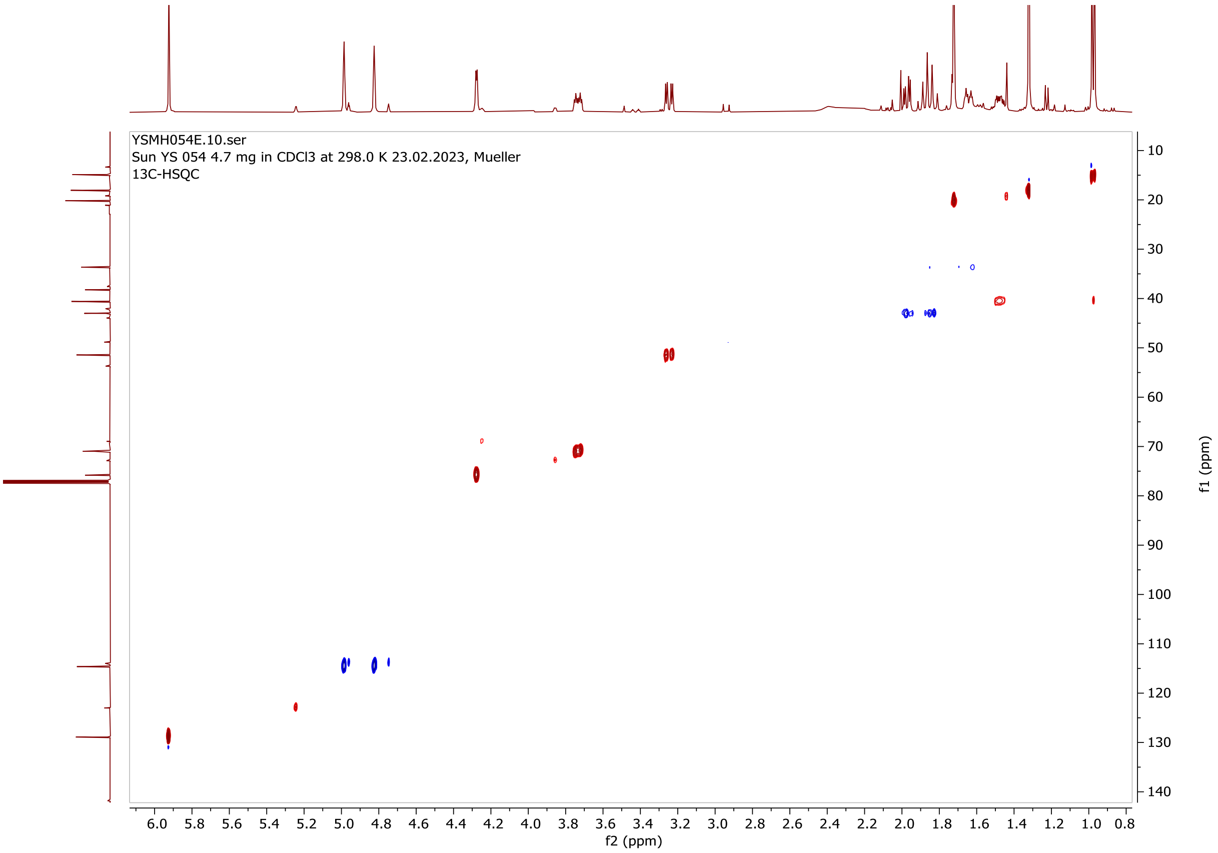

Figure S49 HSQC-spectrum of **10** recorded at 500, 125 MHz in $CDCl_3$

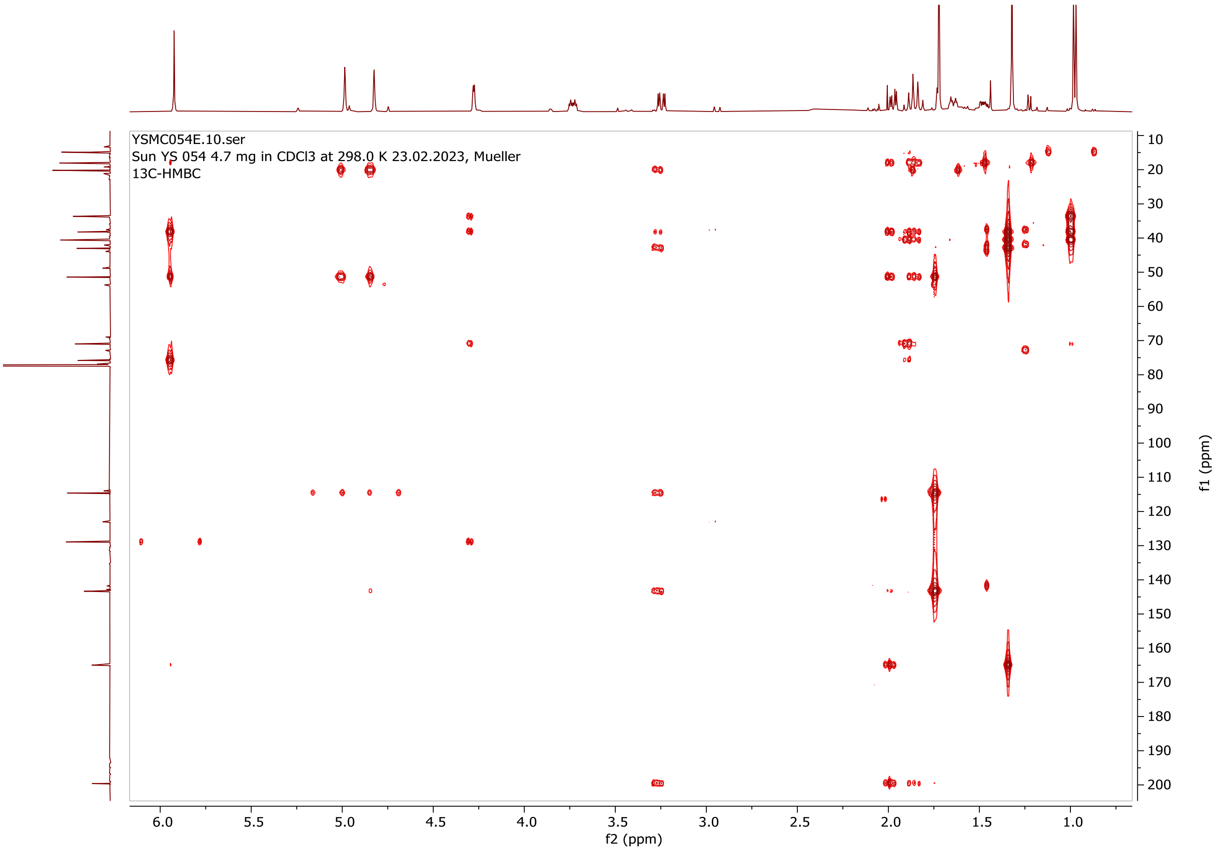

Figure S50 HMBC-spectrum of **10** recorded at 500, 125 MHz in $CDCl_3$

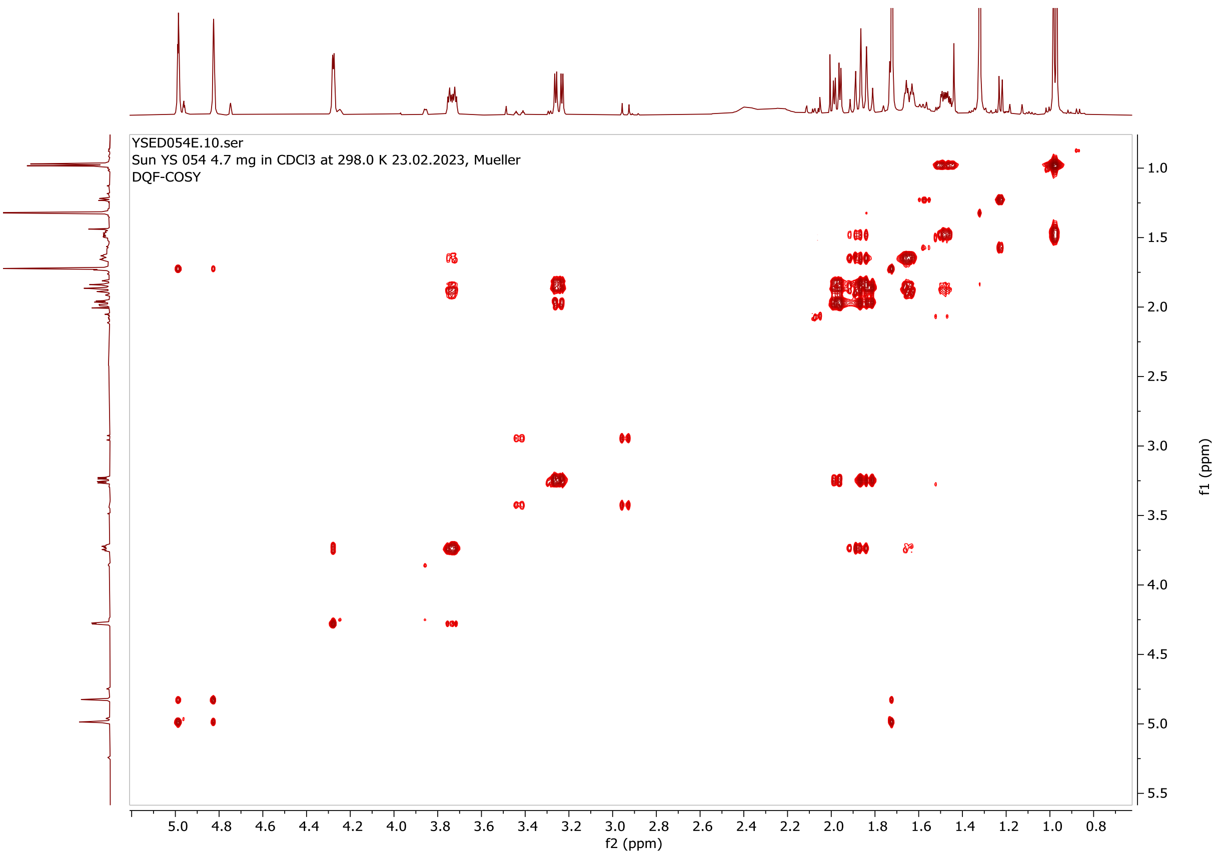

Figure S51 ^{1}H, ^{1}H-COSY -spectrum of **10** recorded at 500 MHz in $CDCl_3$

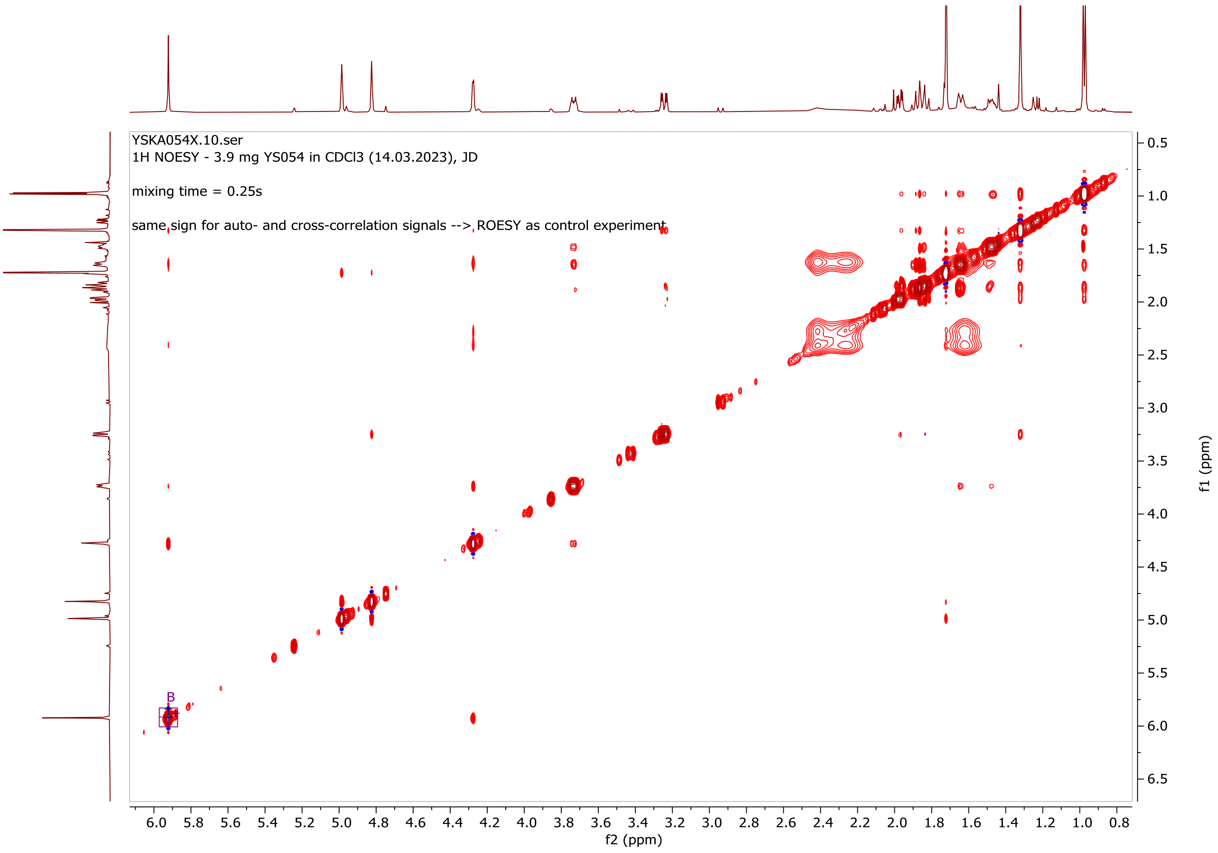

Figure S52 NOESY-spectrum of **10** recorded at 600 MHz in $CDCl_3$

Compound 11

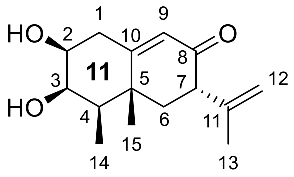

Chemical Formula: $C_{15}H_{22}O_3$
Exact Mass: 250.1569

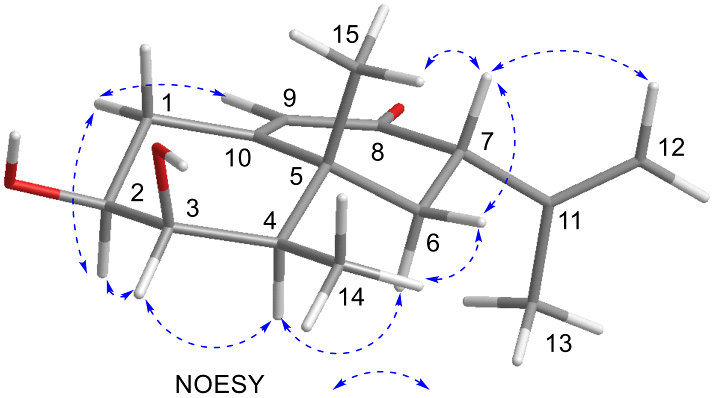

Compound 11				
**Pos.**	***δ*** δ_C **/ ppm**	δ_H **/ ppm (J/Hz)**	**^{1}H-^{1}H COSY**	**HMBC (H-C)**
**1**	36.2	2.34, 1H, dd (13.1, 4.7);<br>2.79, 1H, ddd (13.1, 12.5, 2.1)	1, 2<br>1, 2, 9	2, 3, 9, 10<br>2, 3, 9, 10
**2**	71.4	3.73, 1H, ddd (12.2, 4.8, 3.1)	1, 3	
**3**	74.5	3.86, 1H, dd (3.3, 3.3)	2, 4	2, 4, 5, 14
**4**	44.5	1.47, 1H, dddd (7.1, 7.1, 7.1, 2.6)	14, 3	5, 6, 14, 15
**5**	38.6			
**6**	42.2	1.78, 1H, dd (13.7, 13.7);<br>1.98, 1H, dd (13.0, 4.5)	6, 7<br>6, 7	4, 5, 7, 8, 15<br>5, 7, 8, 10, 15
**7**	50.6	3.15, 1H, dd (14.4, 4.5)	6	5, 6, 8, 11, 12, 13
**8**	199.1			
**9**	125.7	5.82, 1H, d (1.9)	1	1, 5, 7
**10**	167.1			
**11**	143.5			
**12**	114.6	4.81, 1H, m;<br>4.97, 1H, m	12, 13<br>12, 13	7, 11, 13<br>7, 11, 13
**13**	20.2	1.72, 3H, m	12	7, 11, 12
**14**	11.8	1.16, 3H, d (7.1)	4	3, 4, 5
**15**	18.8	1.36, 3H, s		4, 5, 6, 10

Table S15 Summarized NMR signals for ^{13}C, ^{1}H, ^{1}H-^{1}H COSY, HMBC for **11** recorded in $CDCl_3$

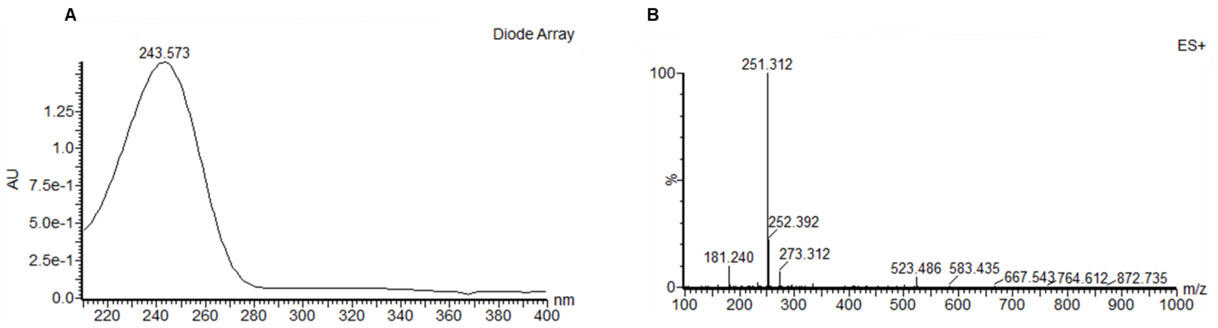

Figure S53 UV-absorption **(A)** and fragmentation pattern **(B)** of **11** in ES^{+} TIC by LR-LCMS

Elemental Composition Report **Page 1**

Single Mass Analysis
Tolerance = 20.0 PPM / DBE: min = -1.5, max = 50.0
Element prediction: Off
Number of isotope peaks used for i-FIT = 3

Monoisotopic Mass, Odd and Even Electron Ions
225 formula(e) evaluated with 6 results within limits (all results (up to 1000) for each mass)
Elements Used:
C: 0-40 H: 0-60 N: 0-5 O: 0-5 Na: 0-1
Sun QTof Premier HAB321
YS 053 680 (6.935) AM (Cen,4, 70.00, Ht,10000.0,556.28,0.70,LS 10) 1: TOF MS ES+ 1.71e+002

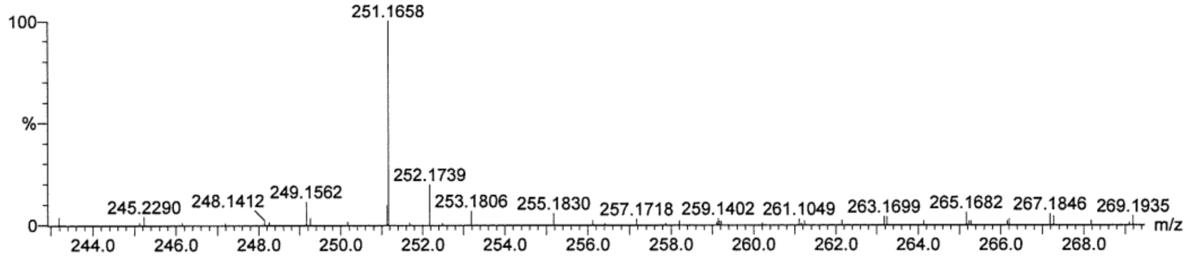

Minimum:				-1.5			
Maximum:		5.0	20.0	50.0			
Mass	Calc. Mass	mDa	PPM	DBE	i-FIT	i-FIT (Norm)	Formula
251.1658	251.1650	0.8	3.2	6.0	12.1	1.5	C16 H22 N Na
	251.1647	1.1	4.4	4.5	12.1	1.5	C15 H23 O3
	251.1674	-1.6	-6.4	9.0	11.5	0.9	C18 H21 N
	251.1634	2.4	9.6	5.0	13.1	2.5	C13 H21 N3 O2
	251.1623	3.5	13.9	1.5	13.5	2.9	C13 H24 O3 Na
	251.1610	4.8	19.1	2.0	14.6	4.0	C11 H22 N3 O2 Na

Figure S54 HRMS data for **11**; *m/z* (M + H)$^{+}$ calc. mass is 251.1647, 251.1658 was found

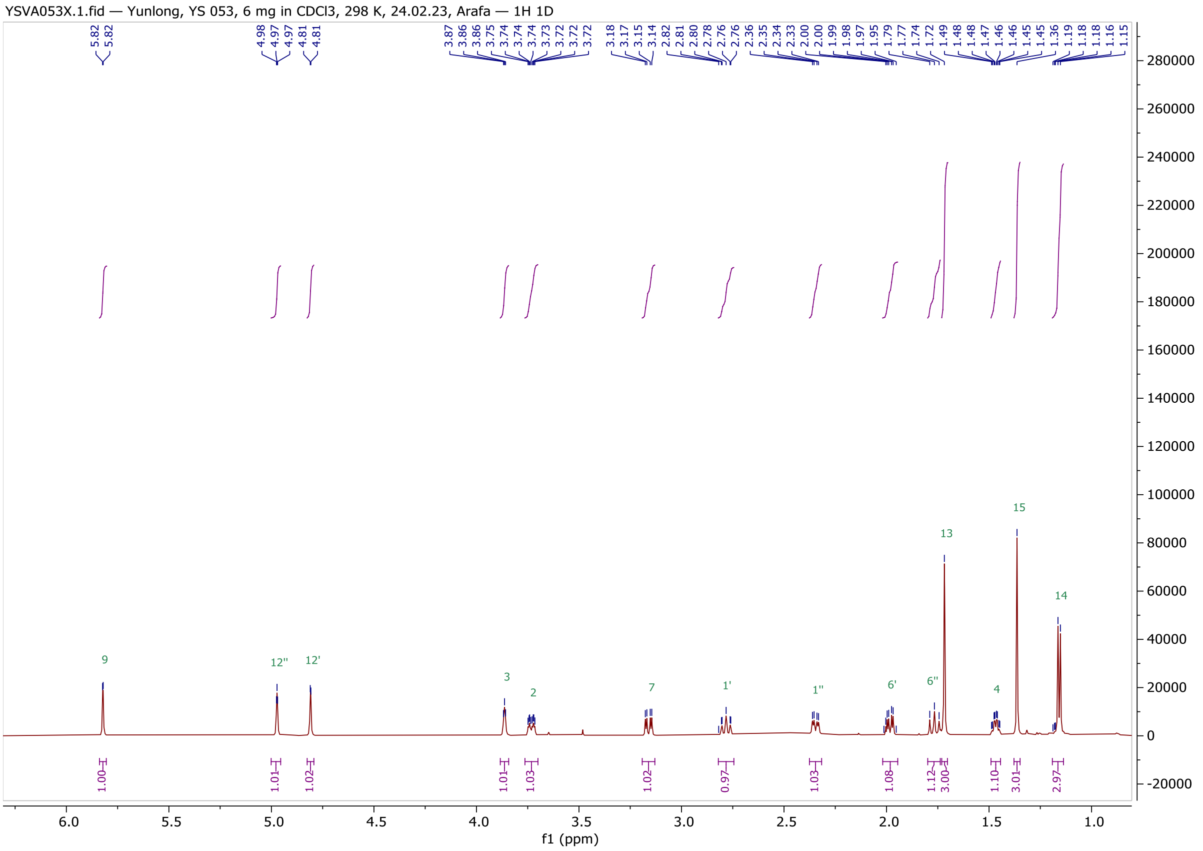

Figure S55 ^{1}H-NMR of **11** recorded at 600 MHz in $CDCl_3$

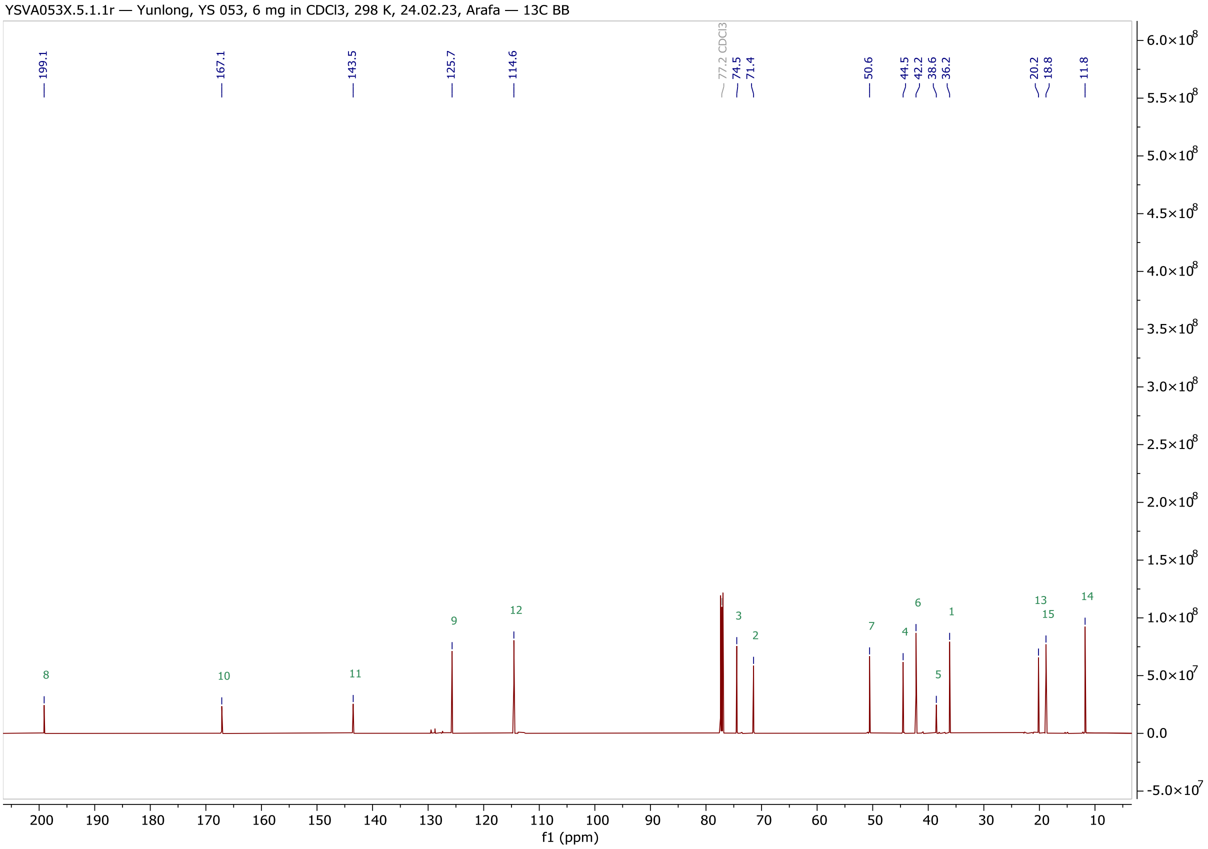

Figure S56 ^{13}C-NMR of **11** recorded at 150 MHz in $CDCl_3$

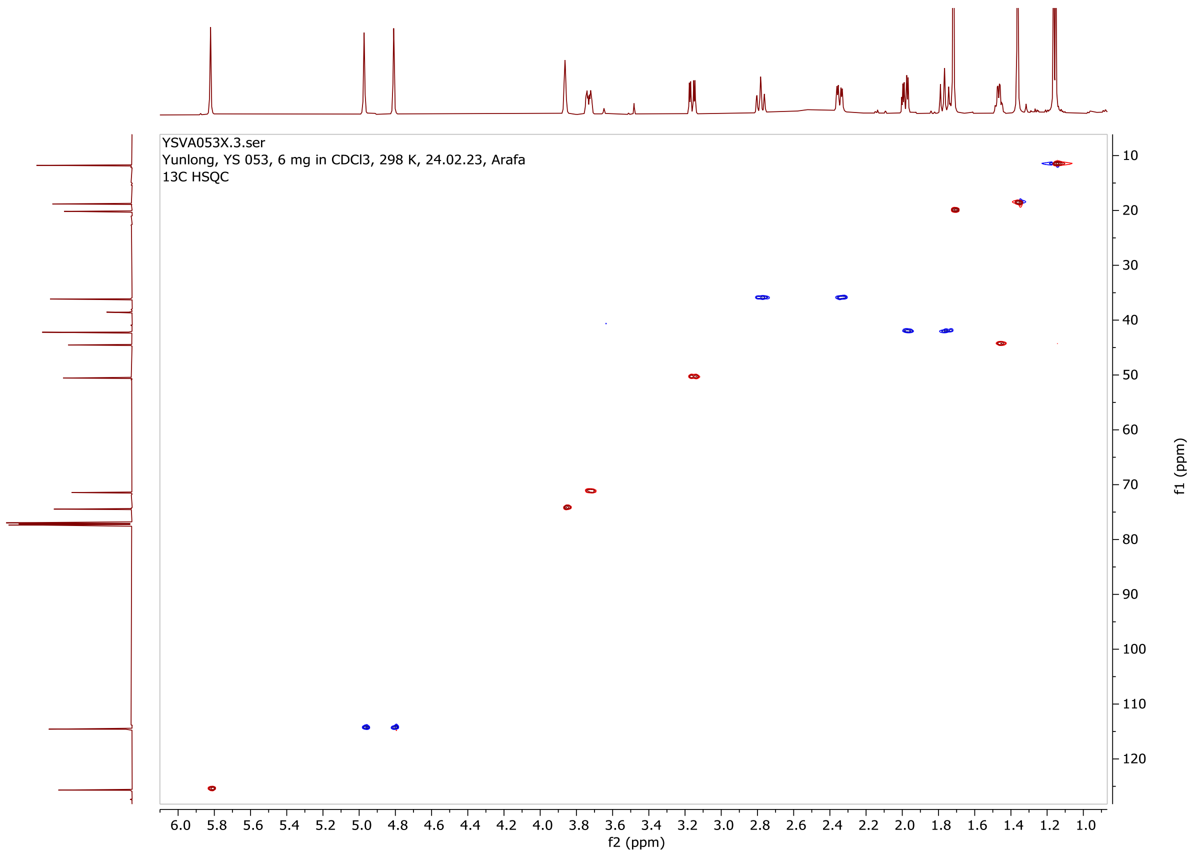

Figure S57 HSQC-spectrum of **11** recorded at 600, 150 MHz in $CDCl_3$

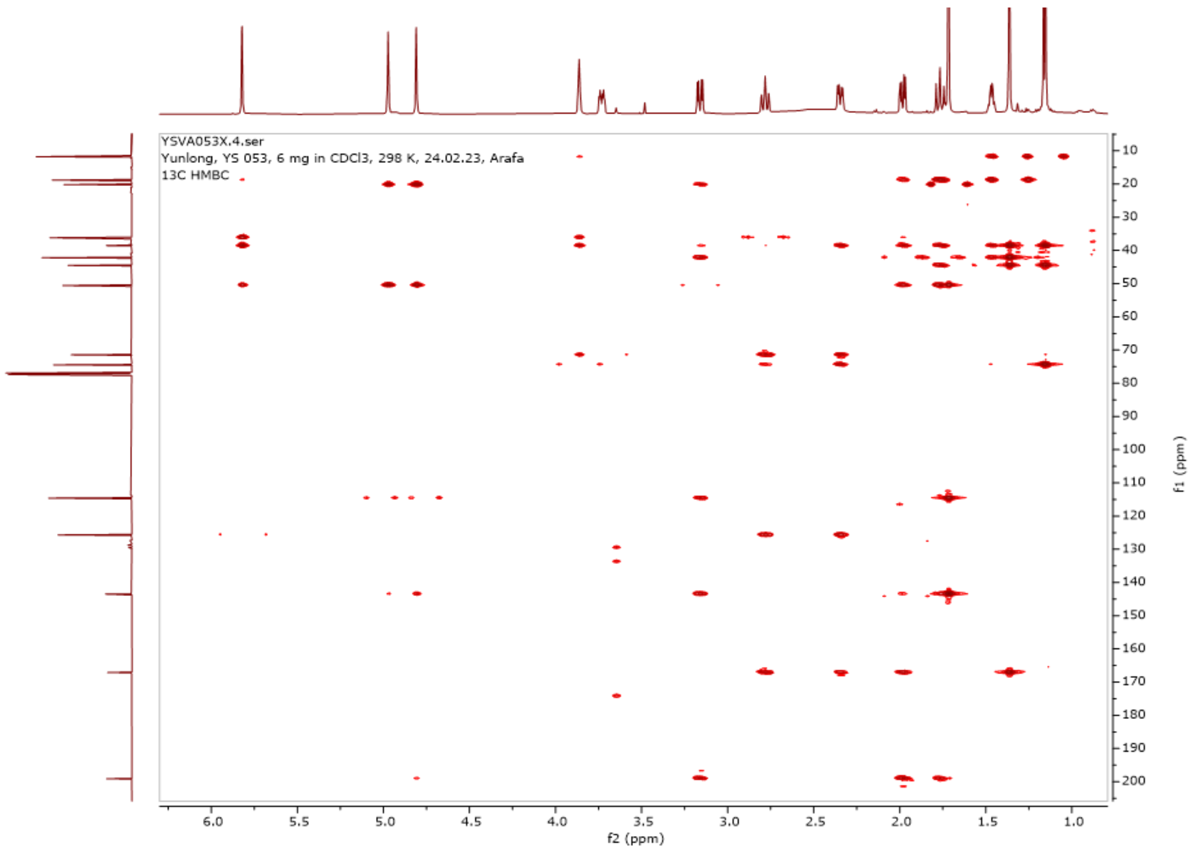

Figure S58 HMBC-spectrum of **11** recorded at 600, 150 MHz in $CDCl_3$

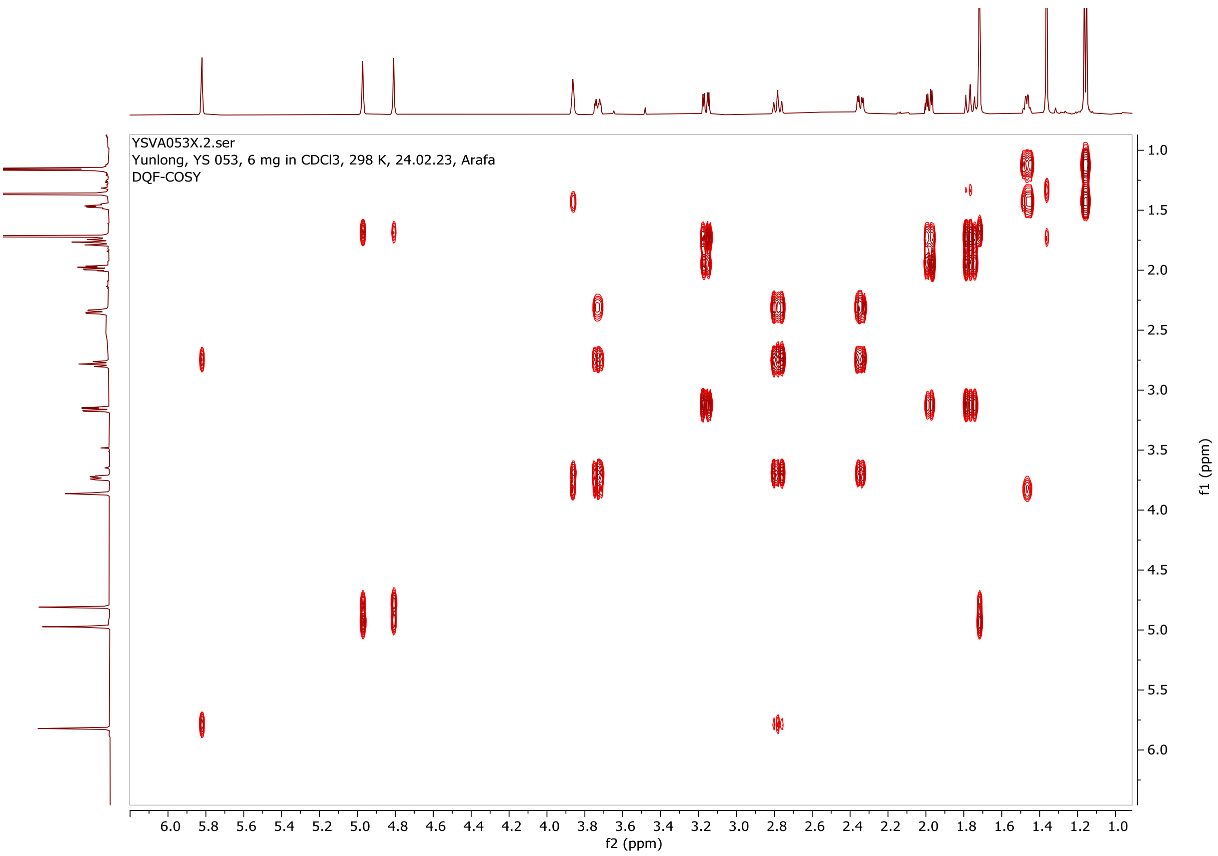

Figure S59 ^{1}H, ^{1}H-COSY-spectrum of **11** recorded at 600 MHz in $CDCl_3$

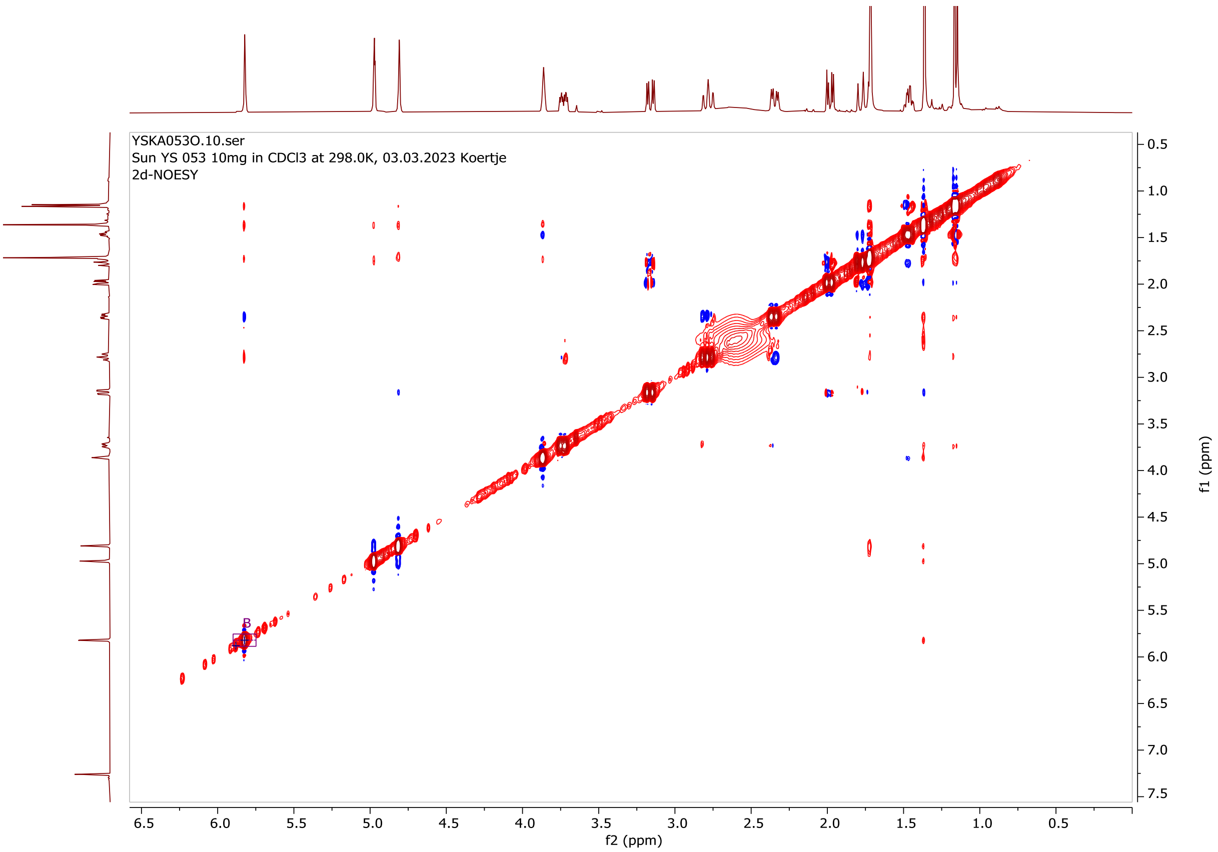

Figure S60 NOESY-spectrum of **11** recorded at 600 MHz in $CDCl_3$

Compound 12

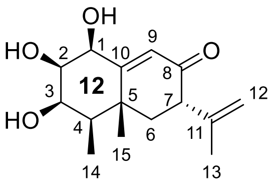

Chemical Formula: $C_{15}H_{22}O_4$
Exact Mass: 266.1518

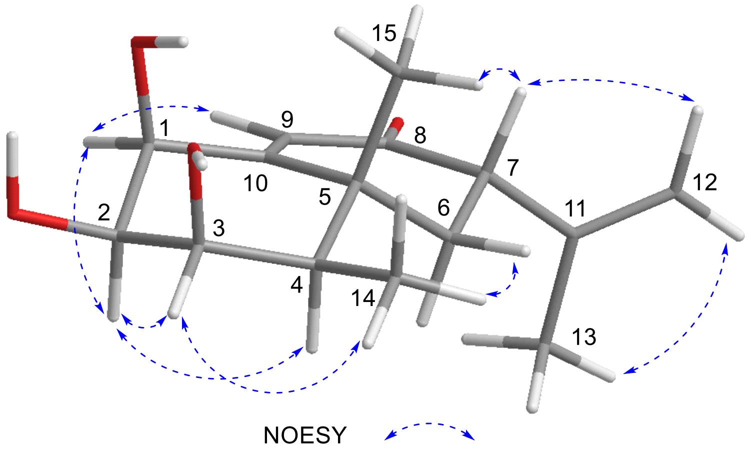

Compound 12				
**Pos.**	**δ_C / ppm**	**δ_H / ppm (*J*/Hz)**	**^{1}H-^{1}H COSY**	**HMBC (H-C)**
**1**	79.2	4.32, 1H, dd (3.4, 1.5)	2, 3	2, 3, 5, 9
**2**	72.1	3.6, 1H, dd (3.4, 3.4)	1, 3	1
**3**	77.8	3.88, 1H, ddd (3.0, 3.0, 1.5)	2, 4, 1	1, 2, 4, 5, 14
**4**	45.9	1.56, 1H, m	3, 14	5, 6, 14, 15
**5**	38.9			
**6**	45.6	1.9, 1H, m;	6, 7	4, 5, 7, 15
		2.02, 1H, dd (12.8, 4.5)	6, 7	5, 7, 10, 15
**7**	52.1	3.35, 1H, m	6	6, 8, 11, 12, 13
**8**	202.0			
**9**	128.3	5.87, 1H, s		1, 5, 7
**10**	168.8			
**11**	144.8			
**12**	114.8	4.82, 1H, m;	12, 13	7, 13
		4.93, 1H, m	12, 13	7, 13
**13**	20.2	1.69, 3H, m	12	7, 11, 12
**14**	12.1	1.19, 3H, d (7.1)	4	3, 4, 5
**15**	21.4	1.53, 3H, s		4, 5, 10

Table S16 Summarized NMR signals for ^{13}C, ^{1}H, ^{1}H-^{1}H COSY, HMBC for **12** recorded in CD_3OD

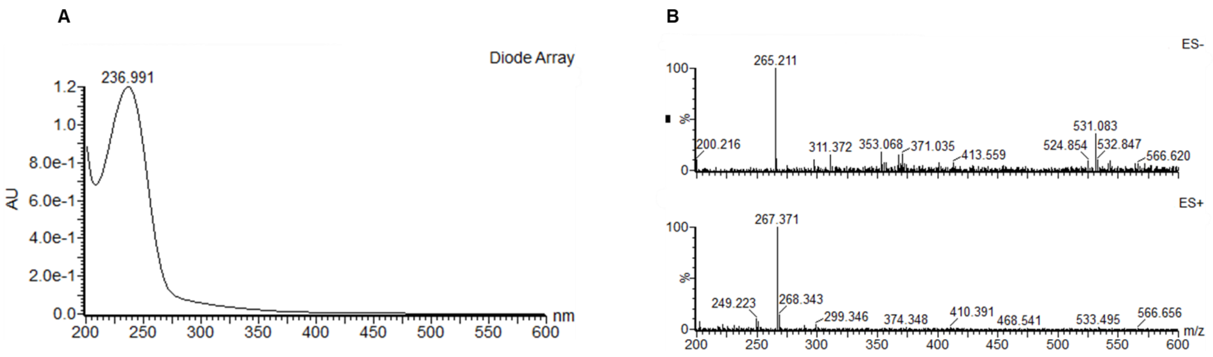

Figure S61 UV-absorption **(A)** and fragmentation pattern **(B)** of **12** in ES^+ TIC (bottom) and ES^- TIC (top) by LR-LCMS

Elemental Composition Report

Page 1

Single Mass Analysis

Tolerance = 20.0 PPM / DBE: min = -1.5, max = 50.0
Element prediction: Off
Number of isotope peaks used for i-FIT = 3

Monoisotopic Mass, Even Electron Ions
37 formula(e) evaluated with 2 results within limits (all results (up to 1000) for each mass)
Elements Used:
C: 0-90 H: 0-50 O: 0-4 Na: 0-1

Sun QTof Premier HAB321
YS 057 503 (5.134) AM (Cen,4, 90.00, Ht,10000.0,556.28,0.70,LS 10) 1: TOF MS ES+ 1.97e+002

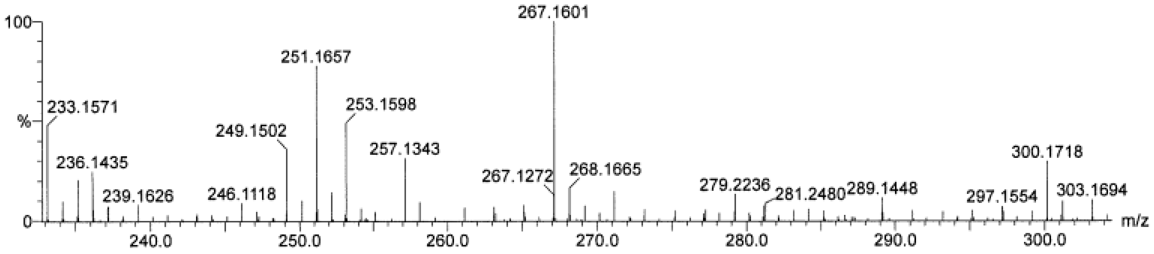

Minimum:				-1.5			
Maximum:		5.0	20.0	50.0			
Mass	Calc. Mass	mDa	PPM	DBE	i-FIT	i-FIT (Norm)	Formula
267.1601	267.1596	0.5	1.9	4.5	24.0	0.2	C15 H23 O4
	267.1572	2.9	10.9	1.5	25.3	1.6	C13 H24 O4 Na

Figure S62 HRMS data for **12**; *m/z* $(M+H)^+$ calc. mass is 267.1596, 267.1601 was found

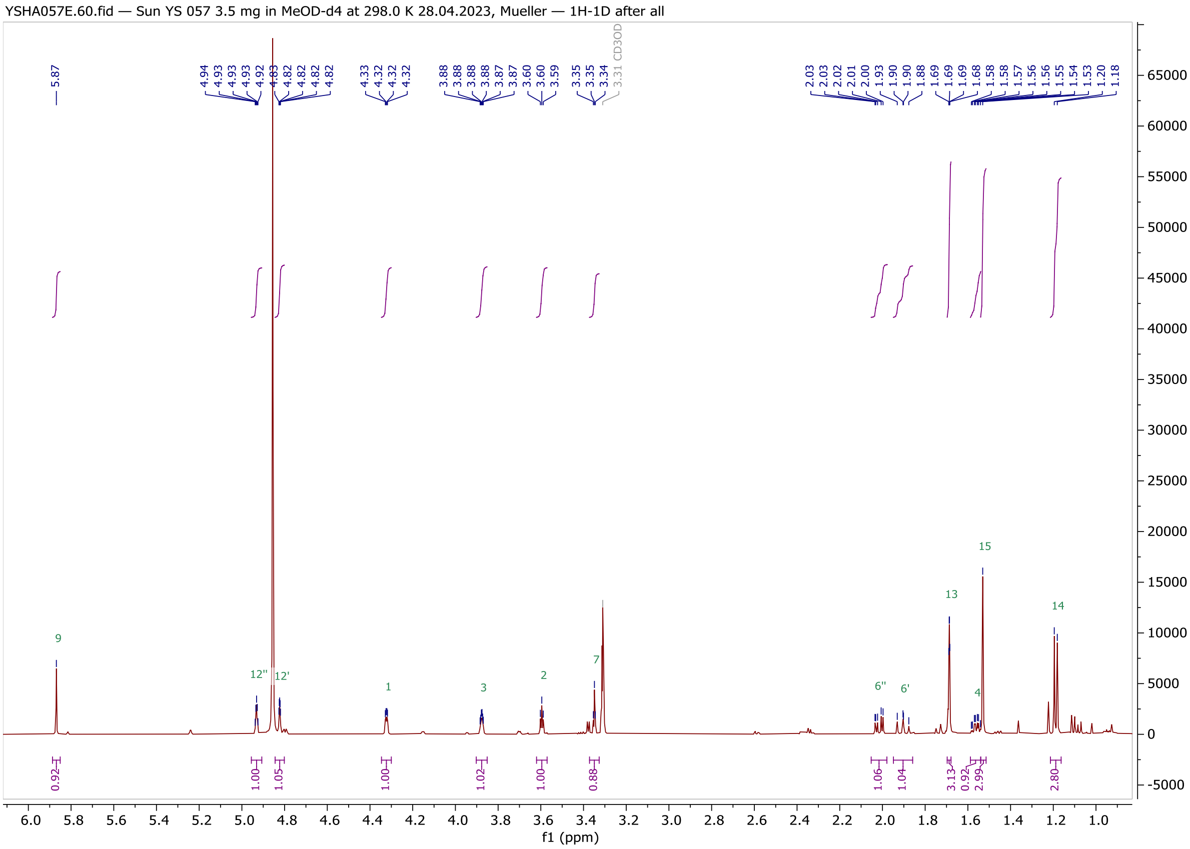

Figure S63 ^{1}H-NMR of **12** recorded at 500 MHz in CD_3OD

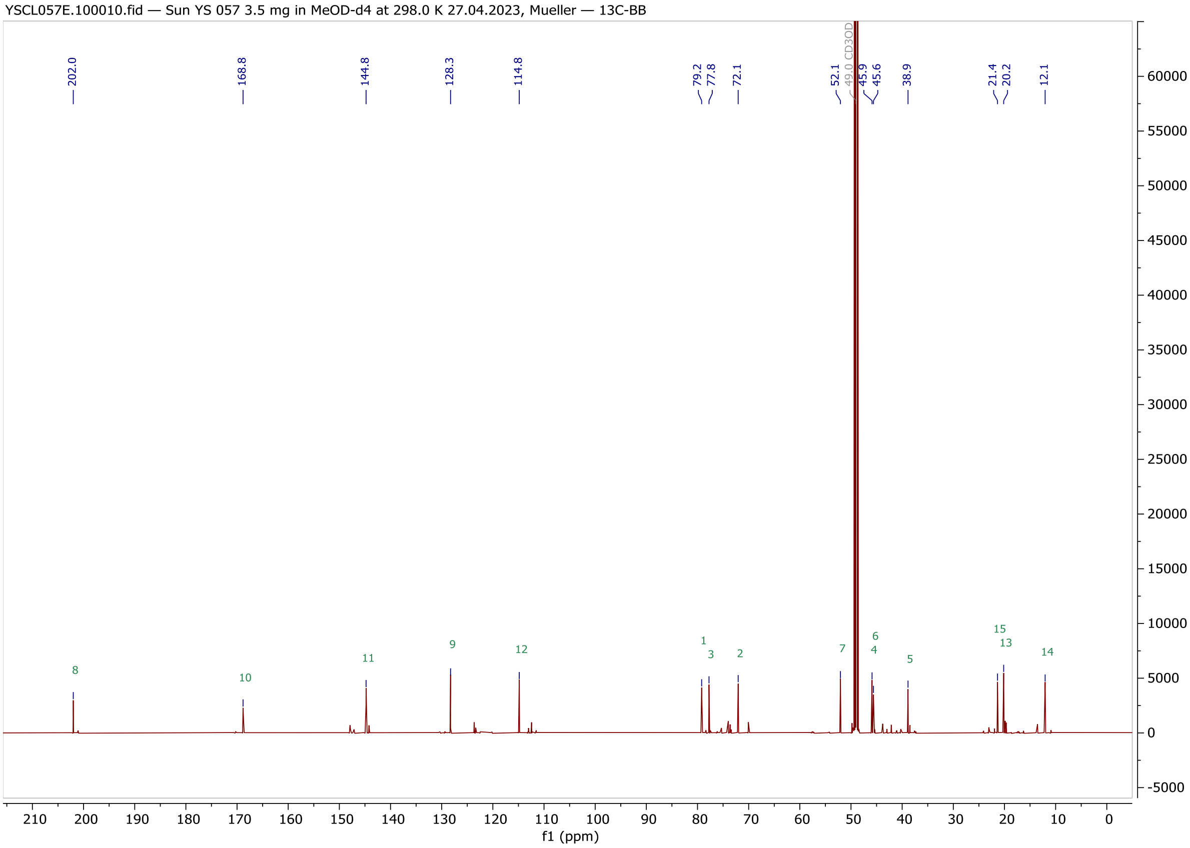

Figure S64 ^{13}C-NMR of **12** recorded at 125 MHz in CD_3OD

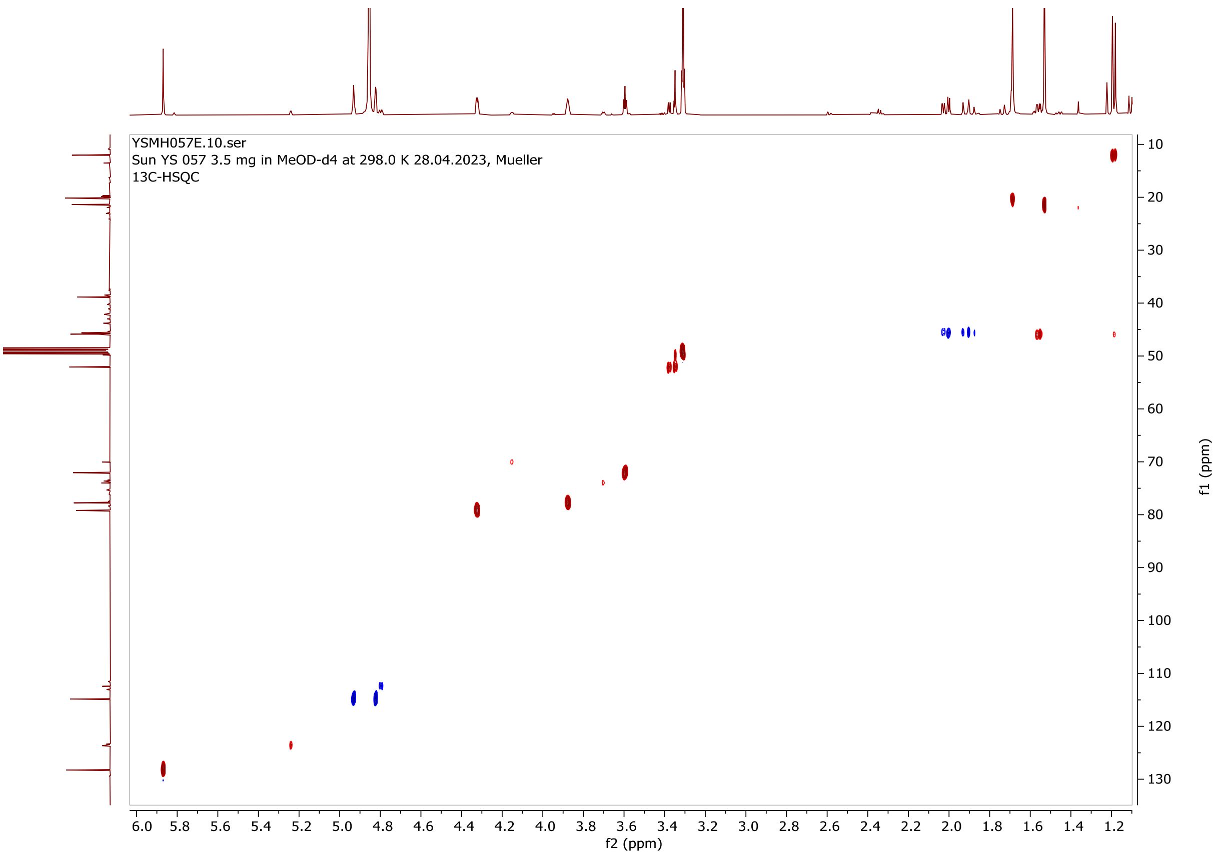

Figure S65 HSQC-spectrum of **12** recorded at 500, 125 MHz in CD_3OD

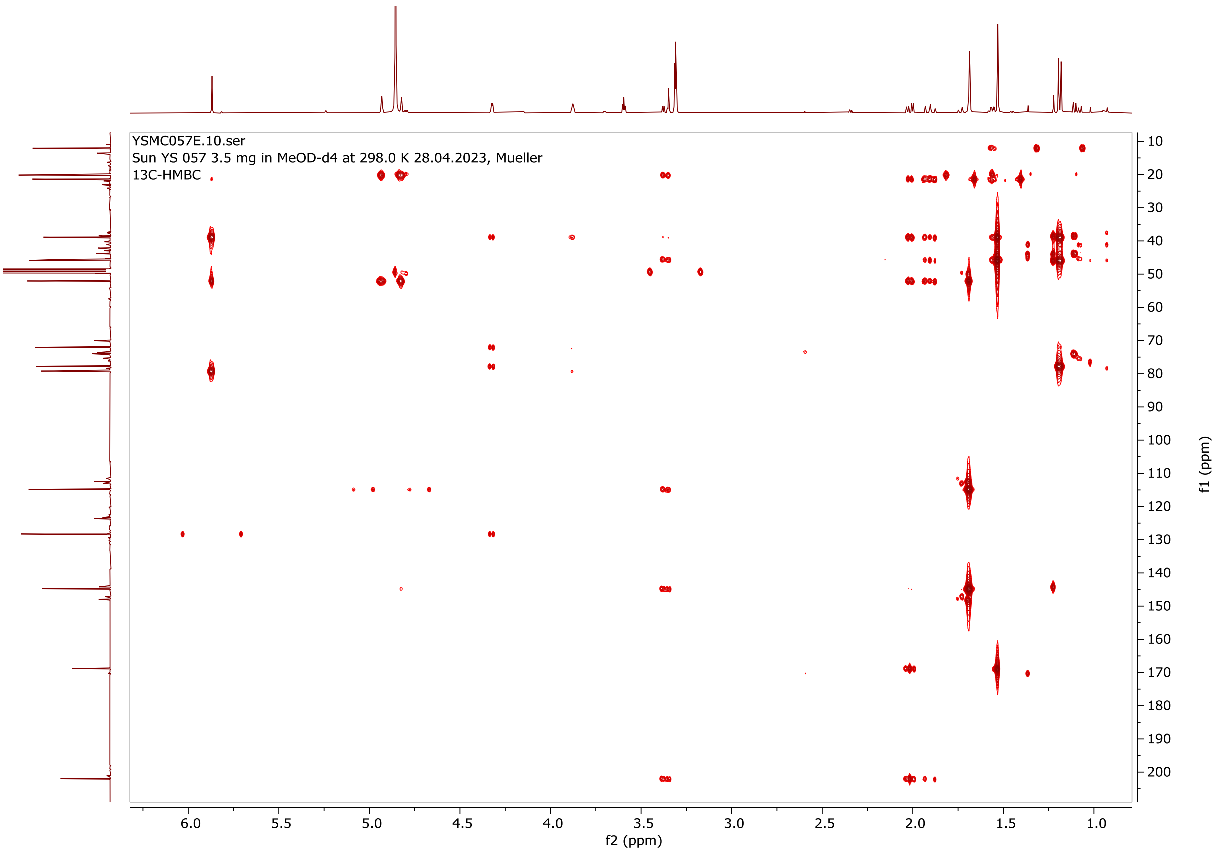

Figure S66 HMBC-spectrum of **12** recorded at 500, 125 MHz in CD_3OD

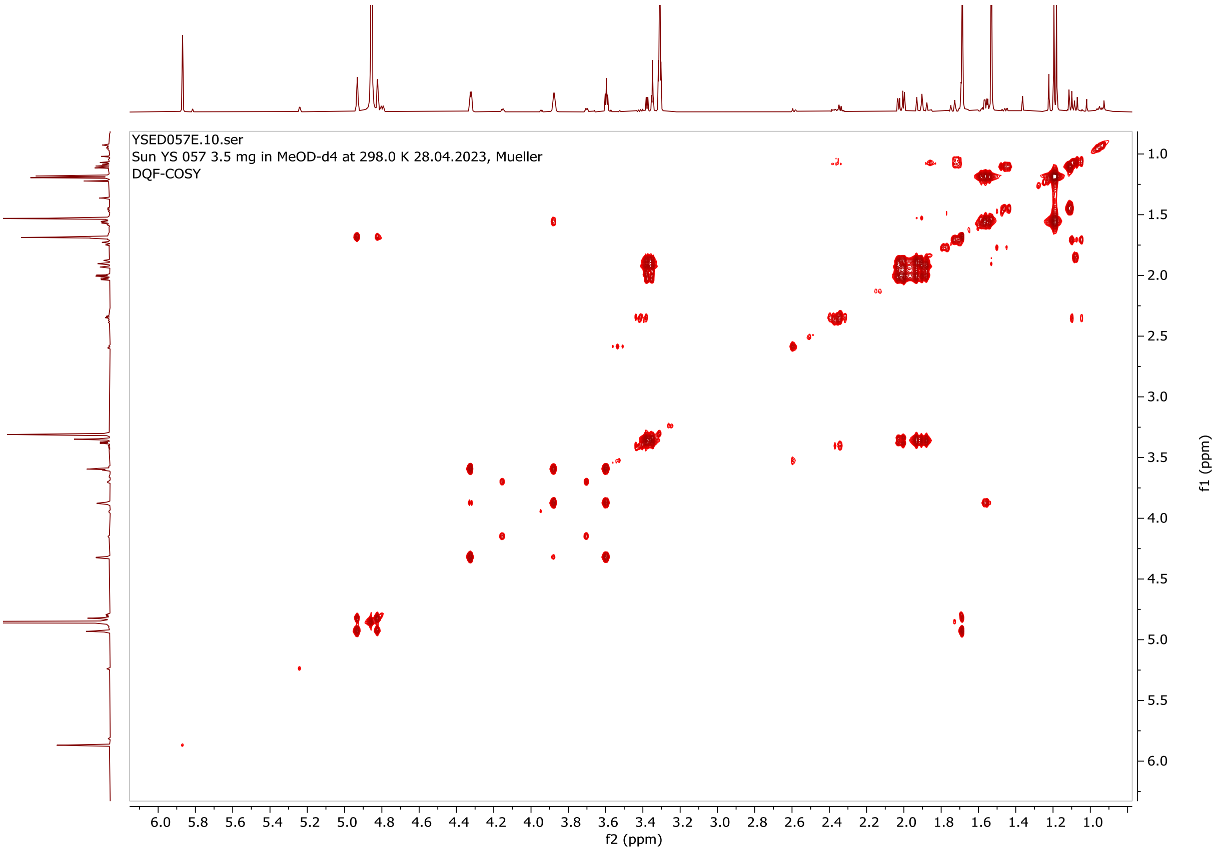

Figure S67 ^{1}H, ^{1}H-COSY-spectrum of **12** recorded at 500 MHz in CD_3OD

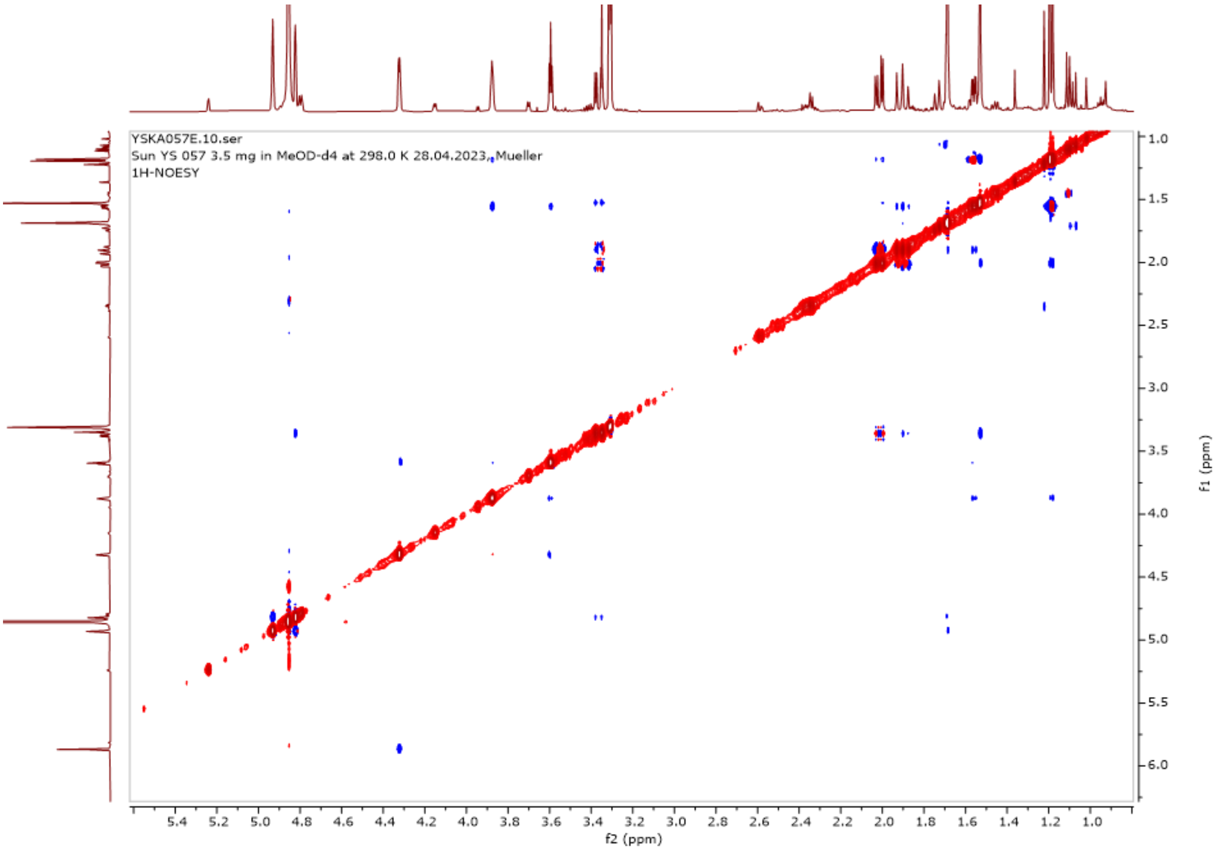

Figure S68 NOESY-spectrum of **12** recorded at 500 MHz in CD_3OD

Compound 13

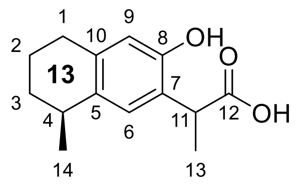

Chemical Formula: $C_{14}H_{18}O_3$
Exact Mass: 234.1256

Compound 13				
**Pos.**	***δ***			

**Pos.**	δ_C / ppm	δ_H / ppm (*J*/Hz)	^{1}H-^{1}H COSY	HMBC (H-C)
**1**	29.8	2.67, 2H, m	1, 2	2, 3, 5, 9, 10
**2**	20.5	1.68, 1H, m;	2, 1, 3	1, 3, 10
		1.84, 1H, m	2, 1, 3	1, 3, 10
**3**	31.7	1.49, 1H, m;	3, 2, 4	1, 2, 4, 14
		1.86, 1H, m	3, 2, 4	1, 2, 4, 14
**4**	31.9	2.82, 1H, m	3, 14	2, 3, 5, 6, 14
**5**	135.0			
**6**	128.4	6.97, 1H, s		8, 10, 11
**7**	123.5			
**8**	151.7			
**9**	117.2	6.58, 1H, s		1, 8, 10
**10**	137.8			
**11**	41.4	3.88, 1H, ddd (7.2, 7.2, 7.2)	13	7, 8, 12, 13
**12**	181.4			
**13**	16.2	1.54, 3H, d (7.3)	11	7, 11, 12
**14**	23.1	1.24, 3H, d (7.0)	4	3, 4, 5

Table S17 Summarized NMR signals for ^{13}C, ^{1}H, ^{1}H-^{1}H COSY, HMBC for **13** recorded in $CDCl_3$

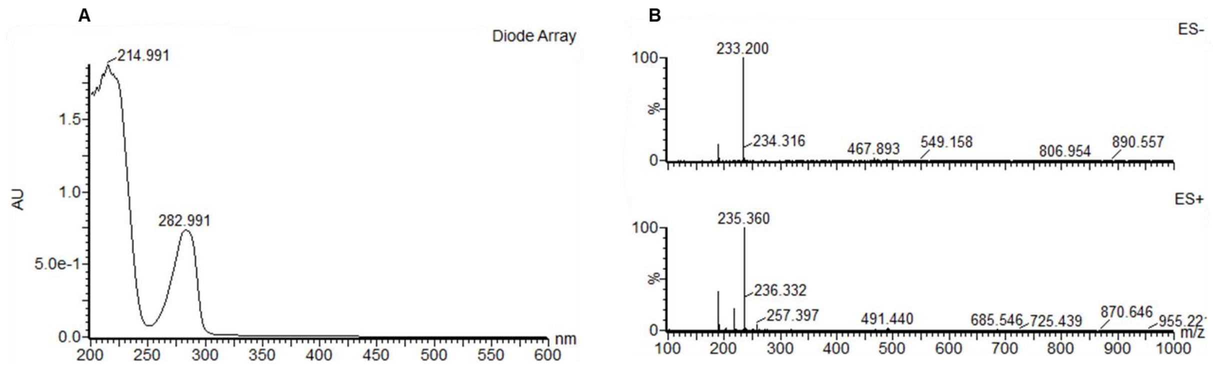

Figure S69 UV-absorption **(A)** and fragmentation pattern **(B)** of **13** in ES$^+$ TIC (bottom) and ES$^-$ TIC (top) by LR-LCMS

Elemental Composition Report

Page 1

Single Mass Analysis

Tolerance = 20.0 PPM / DBE: min = -1.5, max = 50.0
Element prediction: Off
Number of isotope peaks used for i-FIT = 3

Monoisotopic Mass, Odd and Even Electron Ions
319 formula(e) evaluated with 7 results within limits (up to 30 closest results for each mass)
Elements Used:
C: 0-80 H: 0-110 N: 0-16 O: 0-10
Sun QTof Premier HAB321
YS 026 710 (7.259) AM (Cen,3, 50.00, Ht,10000.0,556.28,0.70,LS 10) 1: TOF MS ES+ 1.57e+002

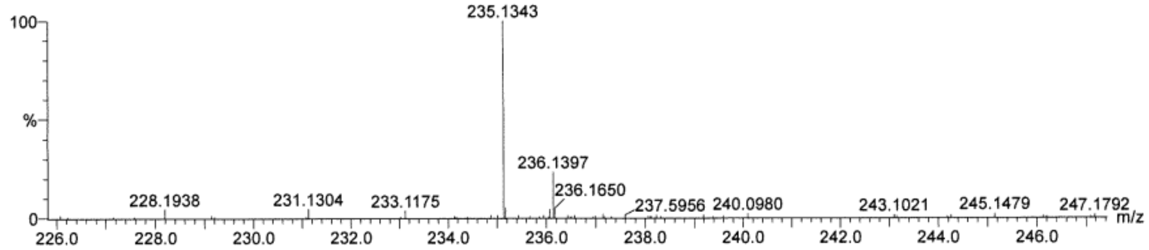

Mass	Calc. Mass	mDa	PPM	DBE	i-FIT	i-FIT (Norm)	Formula
Minimum:				-1.5			
Maximum:		5.0	20.0	50.0			
235.1343	235.1334	0.9	3.8	5.5	42.0	1.2	C14 H19 O3
	235.1353	-1.0	-4.3	3.5	48.7	7.9	H11 N16
	235.1361	-1.8	-7.7	10.0	41.3	0.5	C17 H17 N
	235.1321	2.2	9.4	6.0	43.4	2.6	C12 H17 N3 O2
	235.1366	-2.3	-9.8	3.0	47.1	6.3	C2 H13 N13 O
	235.1307	3.6	15.3	6.5	45.0	4.2	C10 H15 N6 O
	235.1379	-3.6	-15.3	2.5	46.5	5.6	C4 H15 N10 O2

Figure S70 HRMS data for **13**; *m/z* (M+H)$^+$ calc. mass is 235.1334, 235.1343 was found

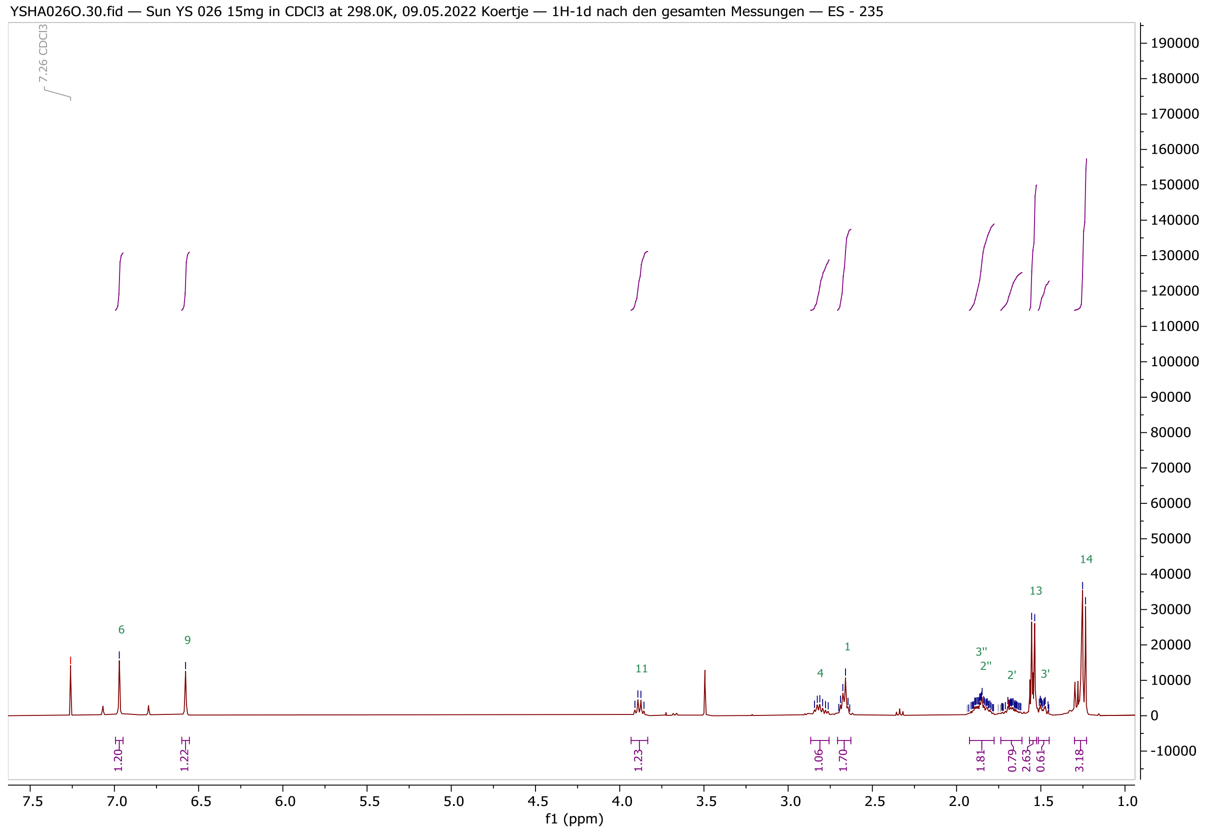

Figure S71 ^{1}H-NMR of **13** recorded at 400 MHz in $CDCl_3$

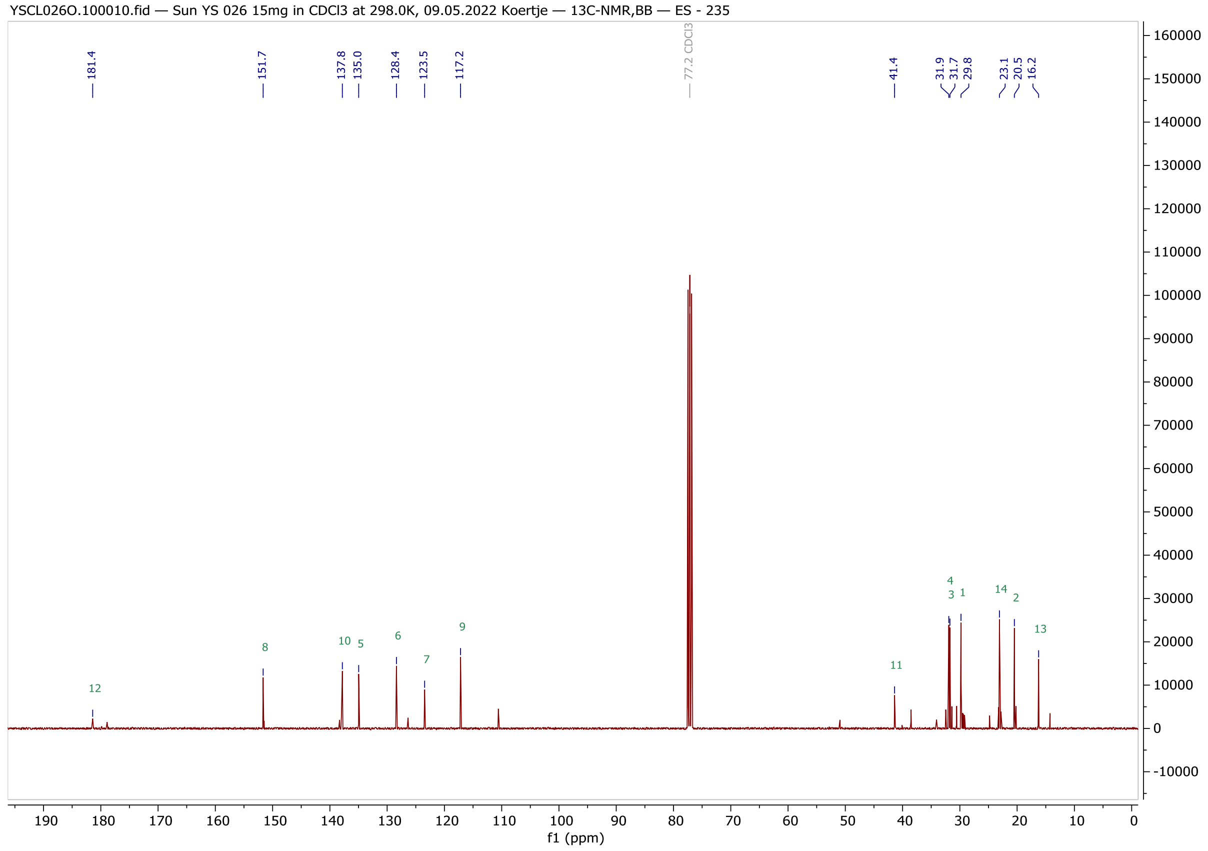

Figure S72 ^{13}C-NMR of **13** recorded at 100 MHz in $CDCl_3$

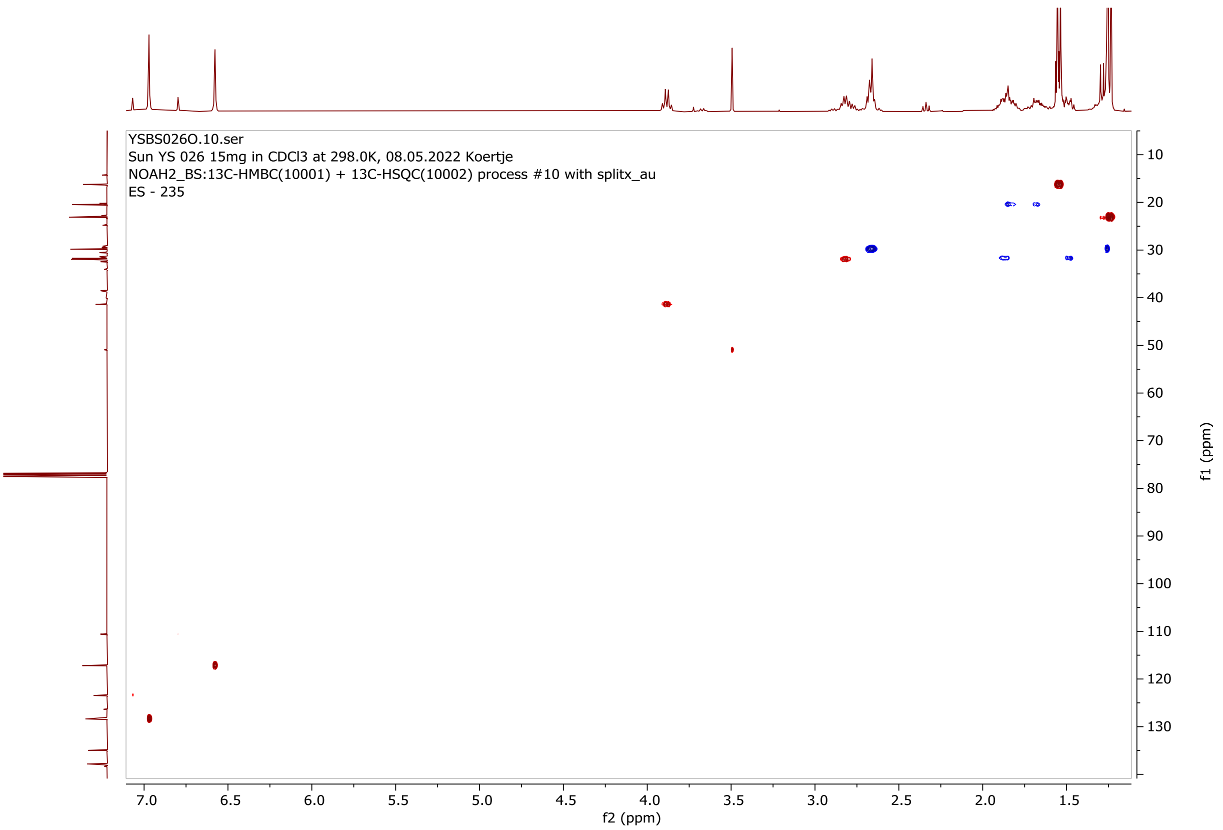

Figure S73 HSQC-spectrum of **13** recorded at 400, 100 MHz in $CDCl_3$

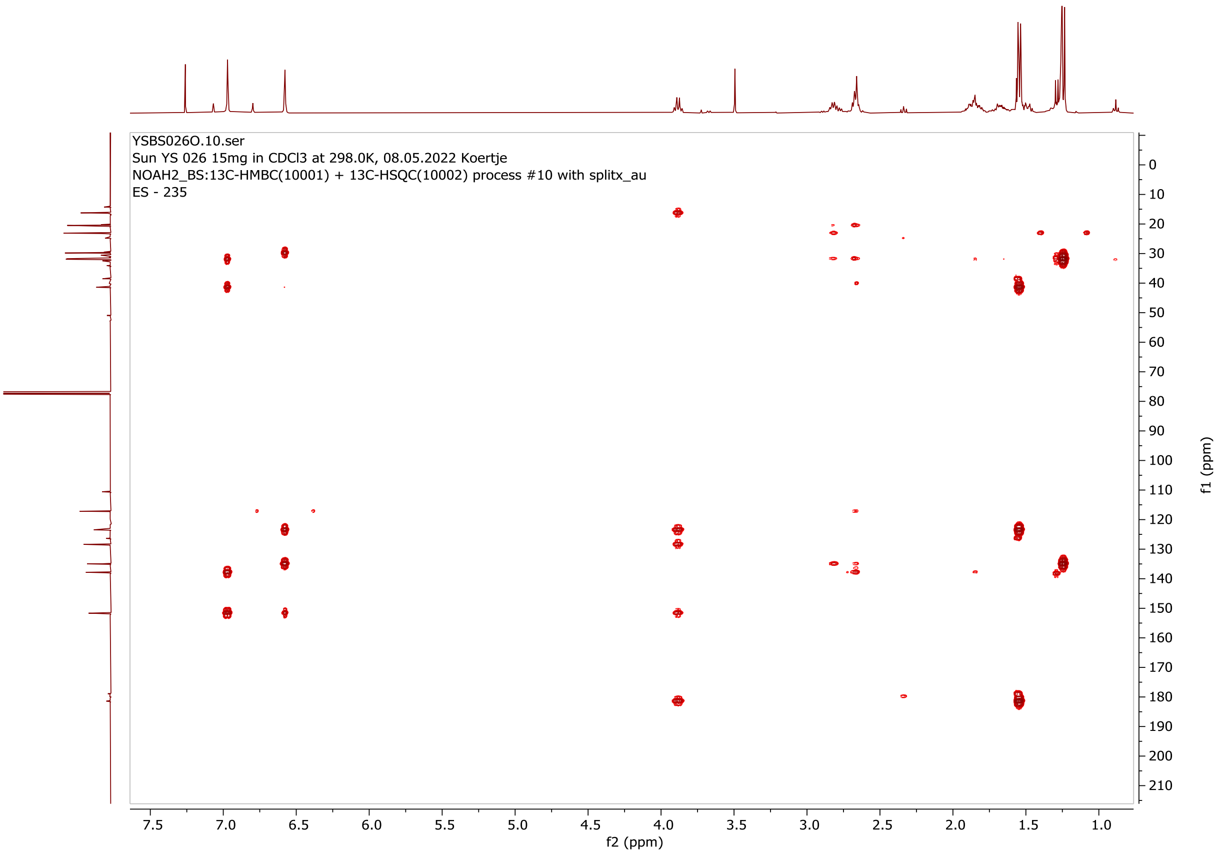

Figure S74 HMBC-spectrum of **13** recorded at 400, 100 MHz in $CDCl_3$

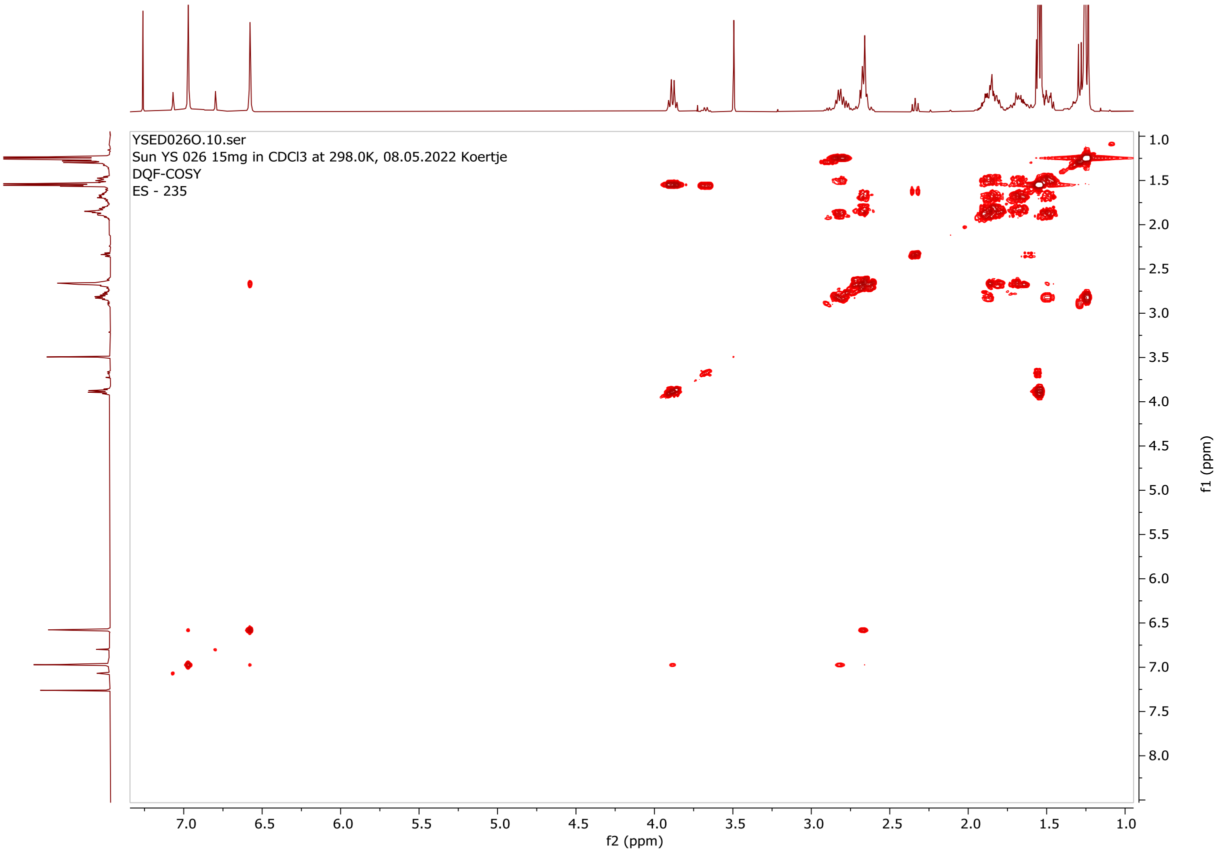

Figure S75 ^{1}H, ^{1}H-COSY-spectrum of **13** recorded at 400 MHz in $CDCl_3$

Compound 14

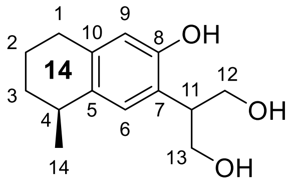

Chemical Formula: $C_{14}H_{20}O_3$
Exact Mass: 236.1412

Compound 14				
**Pos.**	**δ_C / ppm**	**δ_H / ppm (*J*/Hz)**	**^{1}H-^{1}H COSY**	**HMBC (H-C)**
**1**	30.8	2.62, 2H, m	1, 2	2, 3, 5, 9, 10
**2**	21.6	1.66, 1H, m;<br>1.82, 1H, m	2, 1, 3<br>2, 1, 3	1, 3, 10<br>1, 3, 10
**3**	33.1	1.47, 1H, m;<br>1.87, 1H, m	3, 2, 4<br>3, 2, 4	1, 2, 4, 5, 14<br>1, 2, 4, 5, 14
**4**	33.1	2.78, 1H, m	3, 14	2, 3, 5, 6, 10, 14
**5**	134.1			
**6**	129.7	6.91, 1H, s		1, 4, 8, 9, 10, 11
**7**	125.6			
**8**	154.1			
**9**	116.2	6.45, 1H, s		1, 5, 7, 8, 11
**10**	136.9			
**11**	46.4	3.22, 1H, dddd (6.4, 6.4, 6.4, 6.4)	12, 13	6, 7, 8, 12, 13
**12**	64.1	3.86, 2H, m;	12, 11	7, 11, 13
**13**	64.0	3.86, 2H, m	13, 11	7, 11, 12
**14**	23.5	1.23, 3H, d (7.0)	4	3, 4, 5

Table S18 Summarized NMR signals for ^{13}C, ^{1}H, ^{1}H-^{1}H COSY, HMBC for **14** recorded in CD_3OD

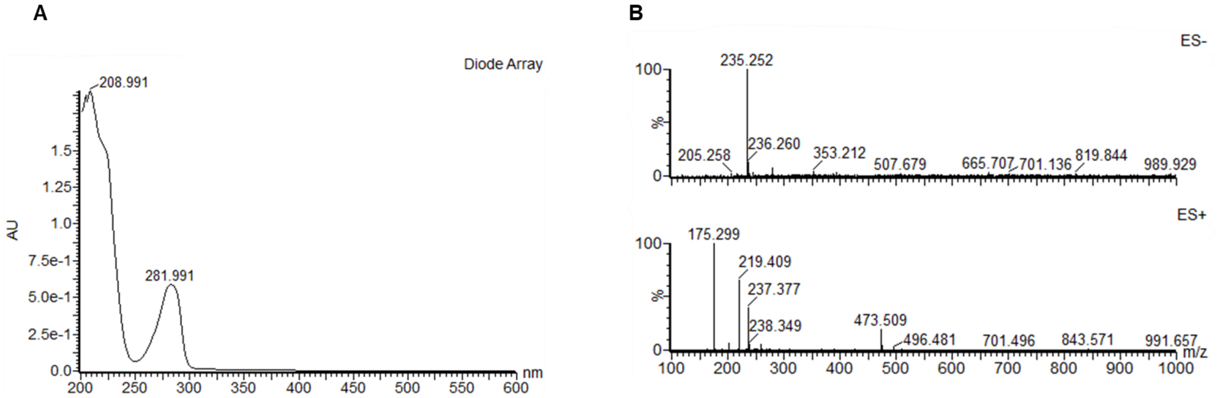

Figure S76 UV-absorption **(A)** and fragmentation pattern **(B)** of **14** in ES^{+} TIC (bottom) and ES^{-} TIC (top) by LR-LCMS

Elemental Composition Report

Page 1

Single Mass Analysis

Tolerance = 20.0 PPM / DBE: min = -1.5, max = 50.0
Element prediction: Off
Number of isotope peaks used for i-FIT = 3

Monoisotopic Mass, Odd and Even Electron Ions
769 formula(e) evaluated with 12 results within limits (all results (up to 1000) for each mass)
Elements Used:
C: 0-95 H: 0-132 N: 0-9 O: 0-24 S: 0-4

Sun QTof Premier HAB321
YS 051 624 (6.361) AM (Cen,4, 70.00, Ht,10000.0,554.26,0.70,LS 10)

1: TOF MS ES-
1.73e+002

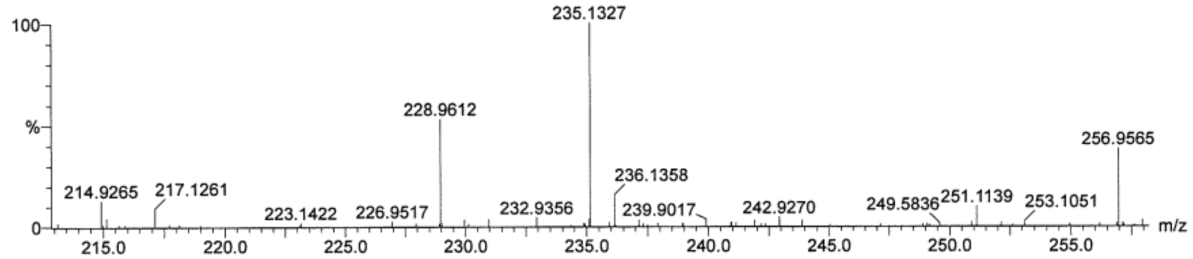

Mass	Calc. Mass	mDa	PPM	DBE	i-FIT	i-FIT (Norm)	Formula
Minimum:				-1.5			
Maximum:		5.0	20.0	50.0			
235.1327	235.1328	-0.1	-0.4	2.0	14.9	4.2	C5 H17 N9 S
	235.1321	0.6	2.6	6.0	11.8	1.1	C12 H17 N3 O2
	235.1334	-0.7	-3.0	5.5	11.6	0.9	C14 H19 O3
	235.1341	-1.4	-6.0	1.5	14.6	3.9	C7 H19 N6 O S
	235.1307	2.0	8.5	6.5	12.9	2.2	C10 H15 N6 O
	235.1303	2.4	10.2	0.5	18.0	7.3	C10 H23 N2 S2
	235.1354	-2.7	-11.5	1.0	15.0	4.2	C9 H21 N3 O2 S

Figure S77 HRMS data for **14**; *m/z* (M-H)$^{-}$ calc. mass is 235.1334, 235.1327 was found

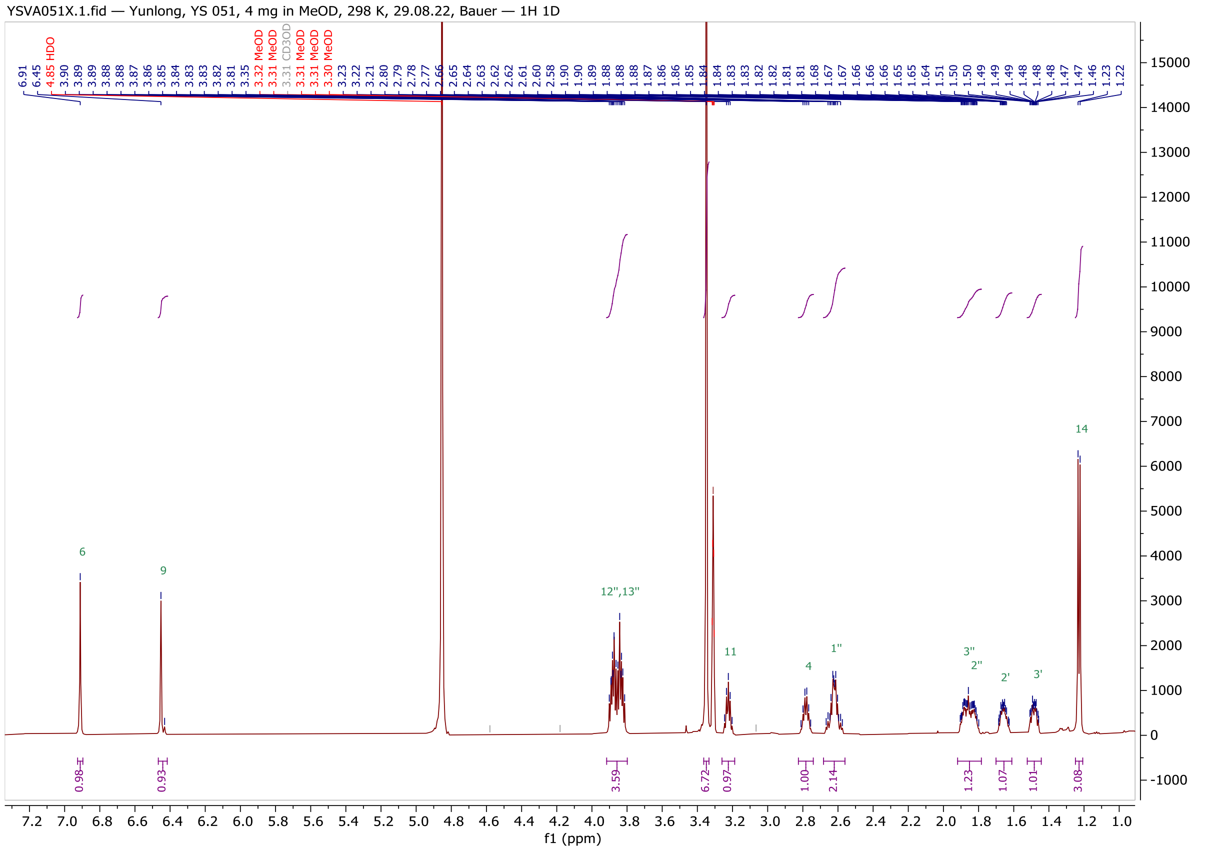

Figure S78 ^{1}H-NMR of **14** recorded at 600 MHz in CD_3OD

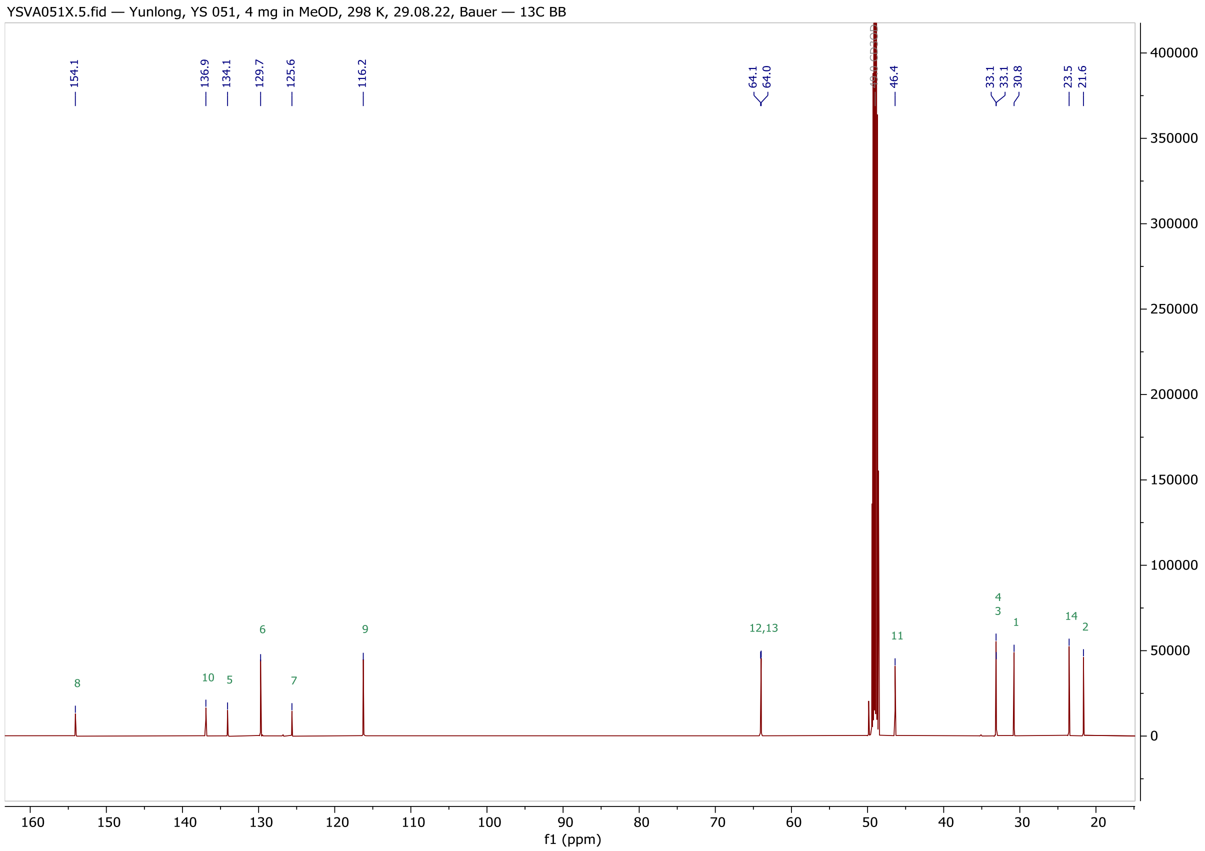

Figure S79 ^{13}C-NMR of **14** recorded at 150 MHz in CD_3OD

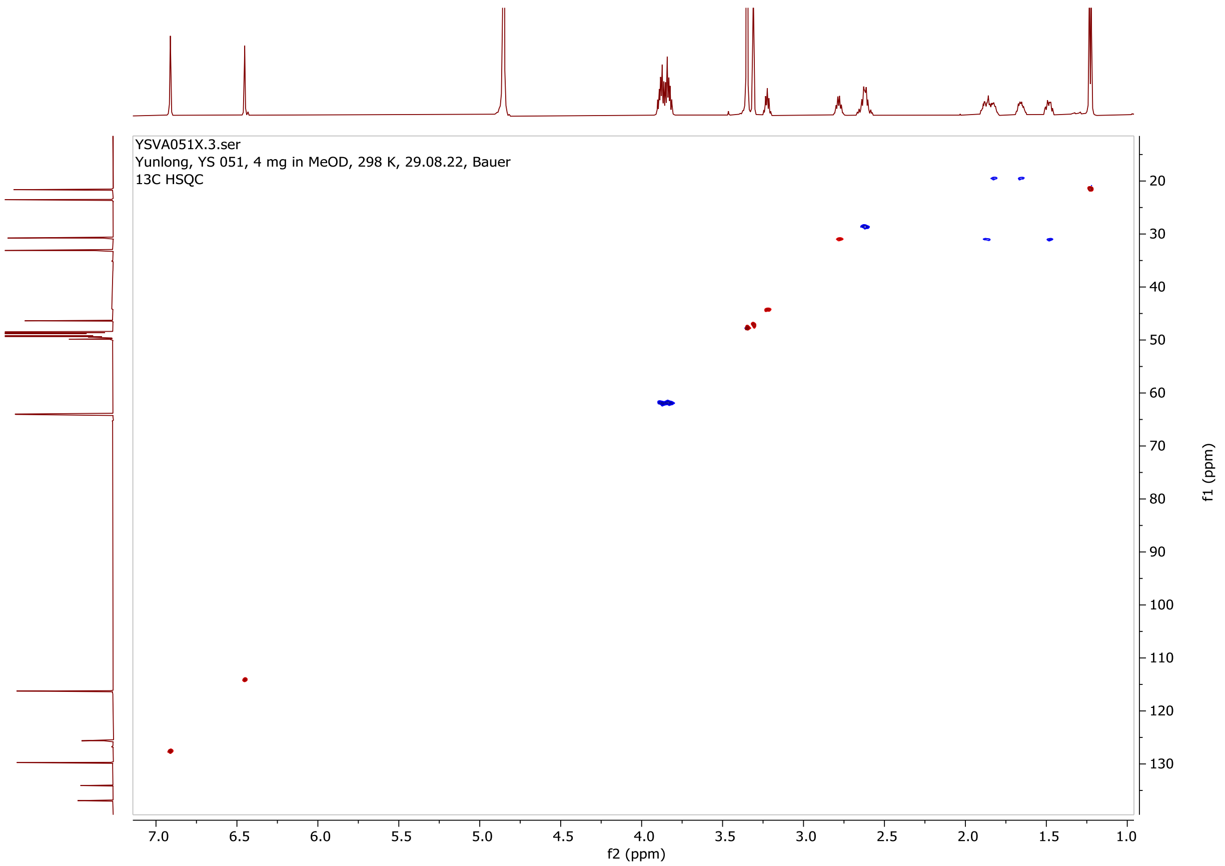

Figure S80 HSQC-spectrum of **14** recorded at 600, 150 MHz in CD_3OD

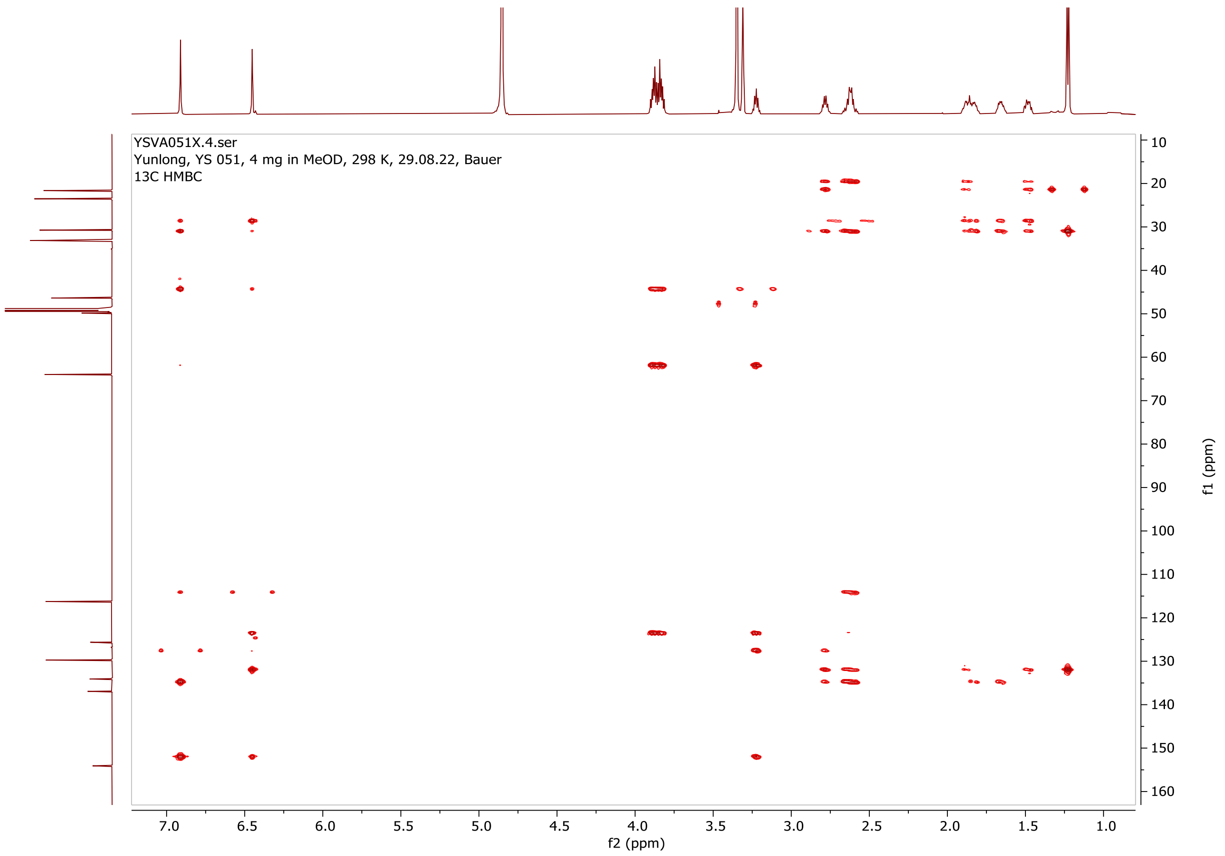

Figure S81 HMBC-spectrum of **14** recorded at 600, 150 MHz in CD_3OD

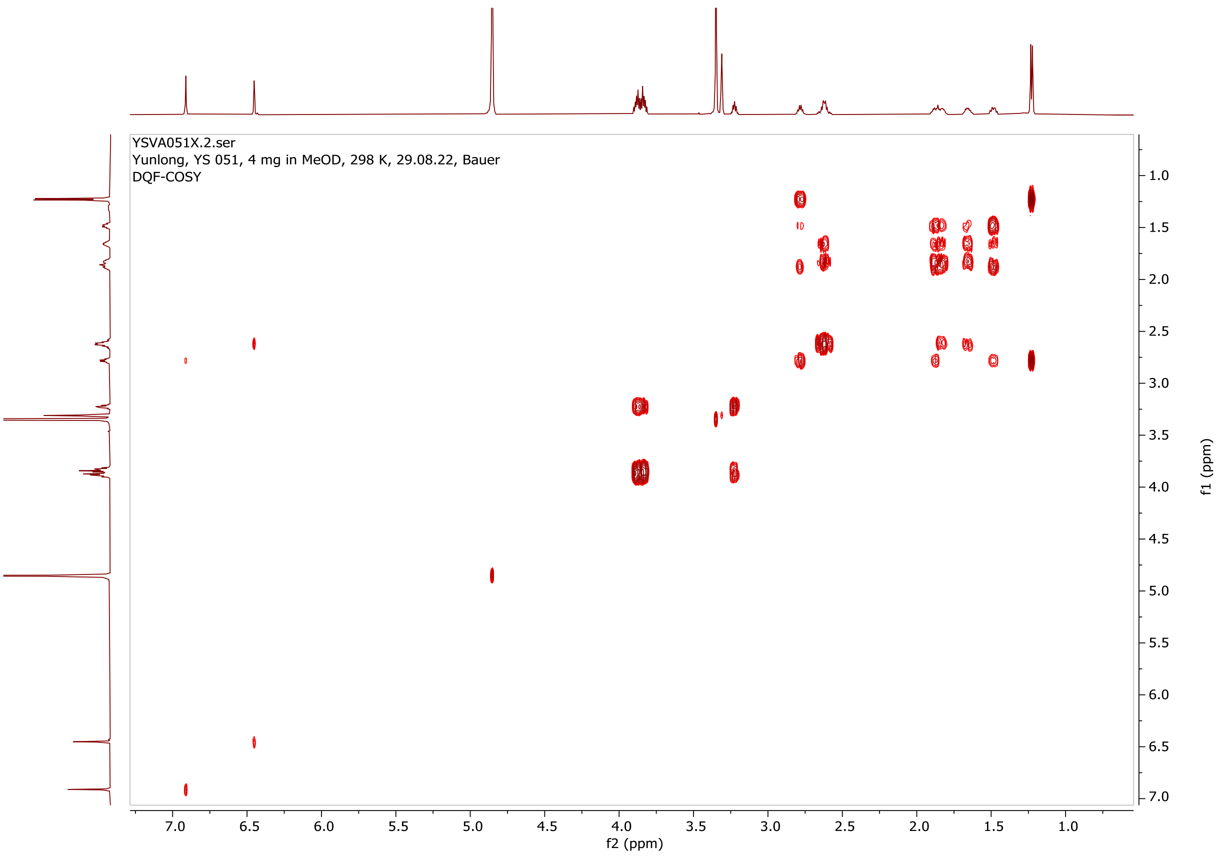

Figure S82 ^{1}H, ^{1}H-COSY-spectrum of **14** recorded at 600 MHz in CD_3OD

Compound 15

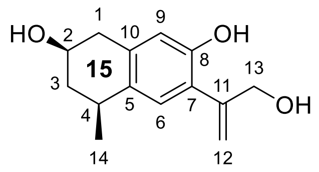

Chemical Formula: $C_{14}H_{18}O_3$
Exact Mass: 234.1256

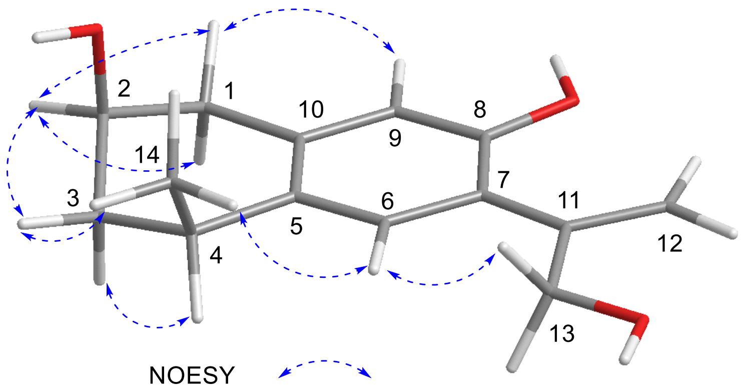

Compound 15				
**Pos.**	**δ_C / ppm**	**δ_H / ppm (*J*/Hz)**	**^{1}H-^{1}H COSY**	**HMBC (H-C)**
**1**	40.4	2.59, 1H, dd (15.5, 10.3);<br>2.9, 1H, ddd (15.6, 5.3, 2.4)	1, 2<br>1, 2, 3	2, 3, 5, 9, 10<br>2, 3, 5, 9, 10
**2**	68.3	3.9, 1H, m	1, 3	
**3**	43.5	1.33, 1H, m;<br>2.14, 1H, dddd (11.9, 5.8, 3.5, 2.4)	3, 2, 4<br>3, 2, 4, 1	1, 2, 4, 5, 14<br>1, 2, 4, 5
**4**	33.3	2.86, 1H, m	3, 14	3, 5, 14
**5**	132.7			
**6**	129.2	7.02, 1H, s		4, 8, 10, 11
**7**	127.2			
**8**	153.5			
**9**	116.5	6.48, 1H, s		1, 5, 7, 8
**10**	136.6			
**11**	149.6			
**12**	114.4	5.15, 1H, m;<br>5.35, 1H, m	12, 13<br>12, 13	7, 11, 13<br>7, 11, 13
**13**	65.9	4.35, 2H, m	12	7, 11, 12
**14**	22.4	1.3, 3H, d (6.8)	4	3, 4, 5

Table S19 Summarized NMR signals for ^{13}C, ^{1}H, ^{1}H-^{1}H COSY, HMBC for **15** recorded in CD_3OD

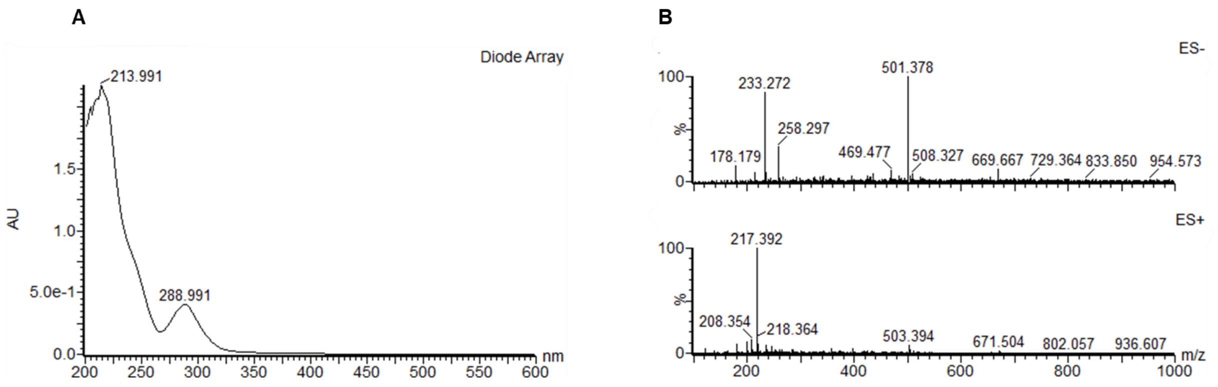

Figure S83 UV-absorption **(A)** and fragmentation pattern **(B)** of **15** in ES$^+$ TIC (bottom) and ES$^-$ TIC (top) by LR-LCMS

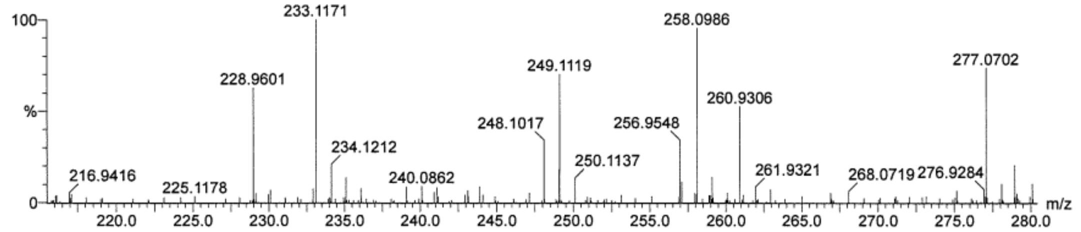

Elemental Composition Report **Page 1**

Single Mass Analysis
Tolerance = 20.0 PPM / DBE: min = -1.5, max = 50.0
Element prediction: Off
Number of isotope peaks used for i-FIT = 3

Monoisotopic Mass, Odd and Even Electron Ions
366 formula(e) evaluated with 7 results within limits (all results (up to 1000) for each mass)
Elements Used:
C: 0-55 H: 0-65 N: 0-4 O: 0-11 S: 0-2
Sun QTof Premier HAB321
YS 052 503 (5.144) AM (Cen,4, 70.00, Ht,10000.0,554.26,0.70,LS 10) 1: TOF MS ES-
1.50e+002

Minimum:				-1.5			
Maximum:		5.0	20.0	50.0			
Mass	Calc. Mass	mDa	PPM	DBE	i-FIT	i-FIT (Norm)	Formula
233.1171	233.1178	-0.7	-3.0	6.5	38.3	2.3	C14 H17 O3
	233.1164	0.7	3.0	7.0	38.6	2.6	C12 H15 N3 O2
	233.1146	2.5	10.7	1.5	36.6	0.6	C10 H21 N2 S2
	233.1198	-2.7	-11.6	2.0	38.6	2.6	C9 H19 N3 O2 S
	233.1204	-3.3	-14.2	11.0	38.1	2.1	C17 H15 N
	233.1137	3.4	14.6	2.5	40.3	4.2	C9 H17 N2 O5
	233.1211	-4.0	-17.2	1.5	38.6	2.6	C11 H21 O3 S

Figure S84 HRMS data for **15**; *m/z* (M-H)$^-$ calc. mass is 233.1178, 233.1171 was found

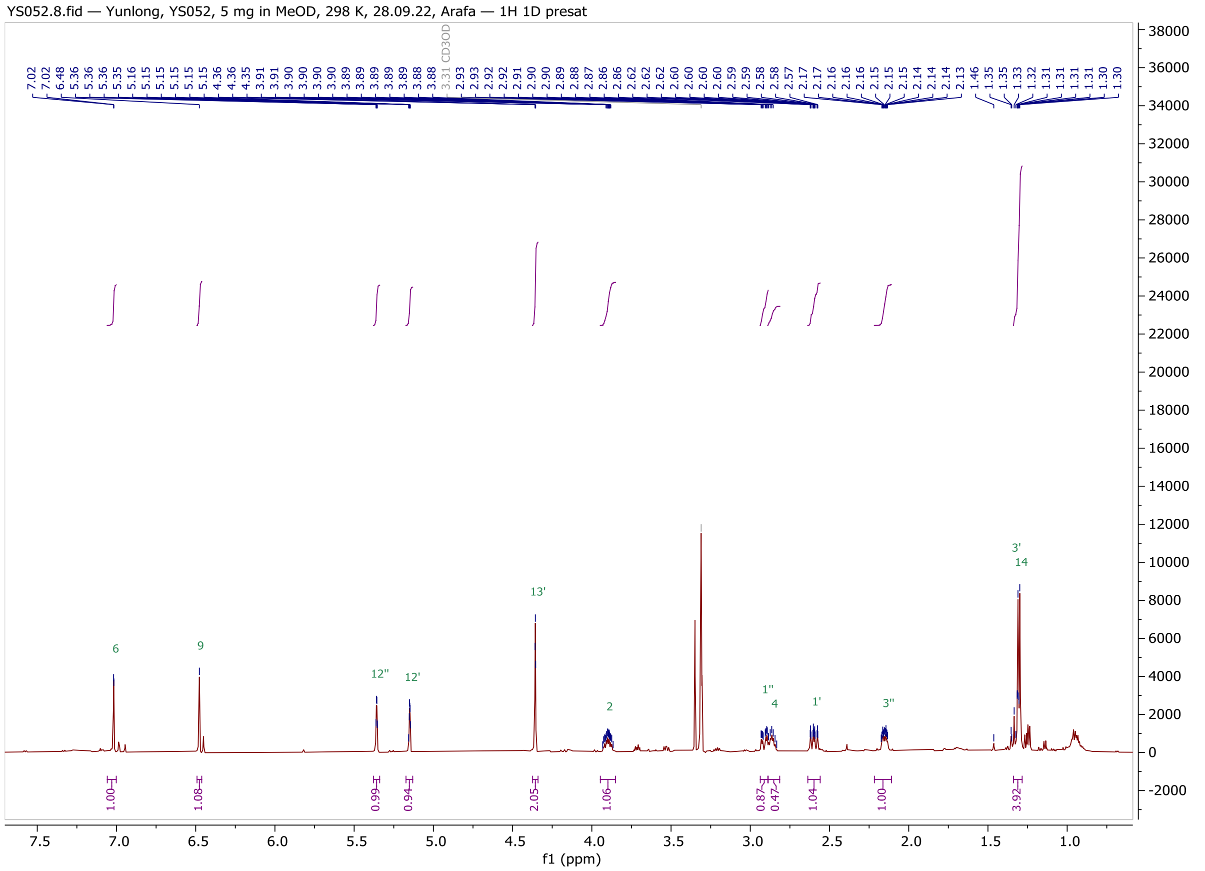

Figure S85 ^{1}H-NMR of **15** recorded at 600 MHz in CD_3OD

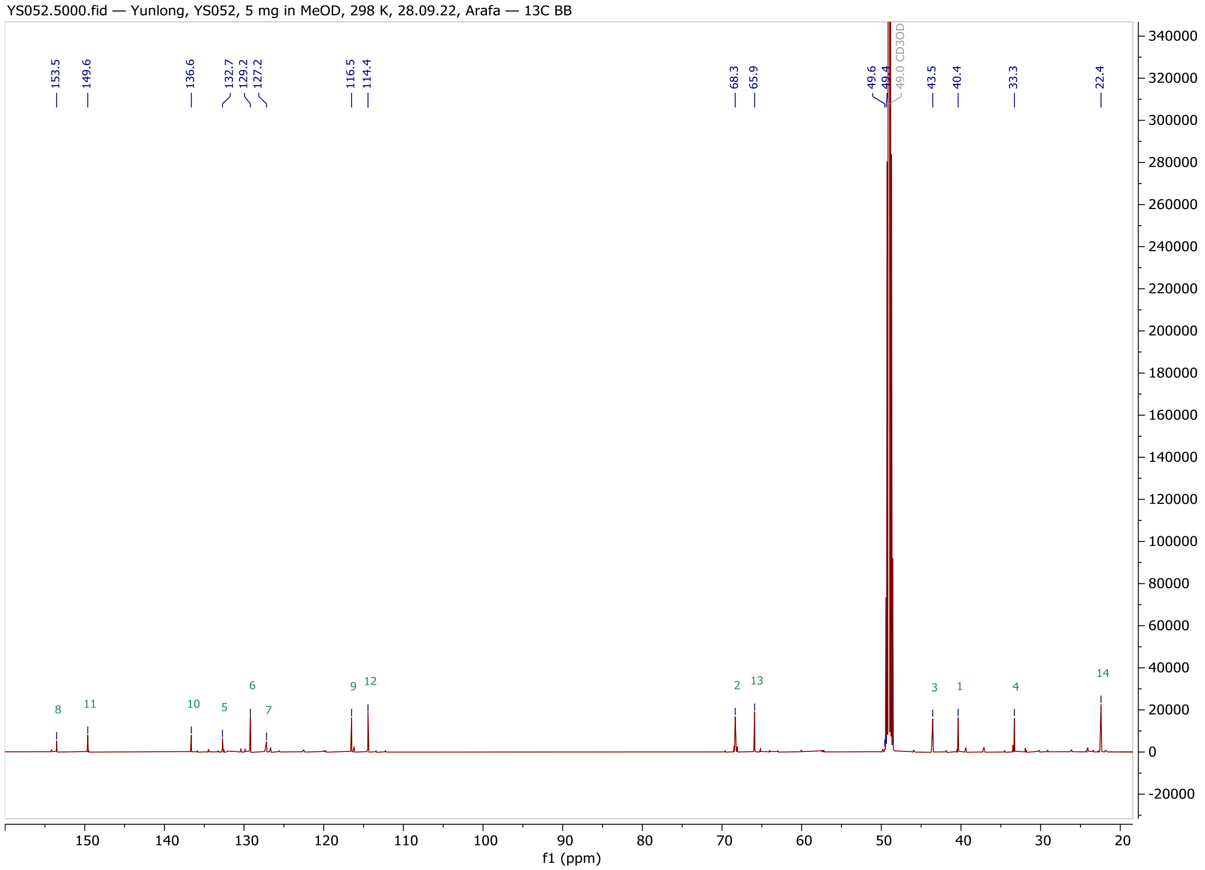

Figure S86 ^{13}C-NMR of **15** recorded at 150 MHz in CD_3OD

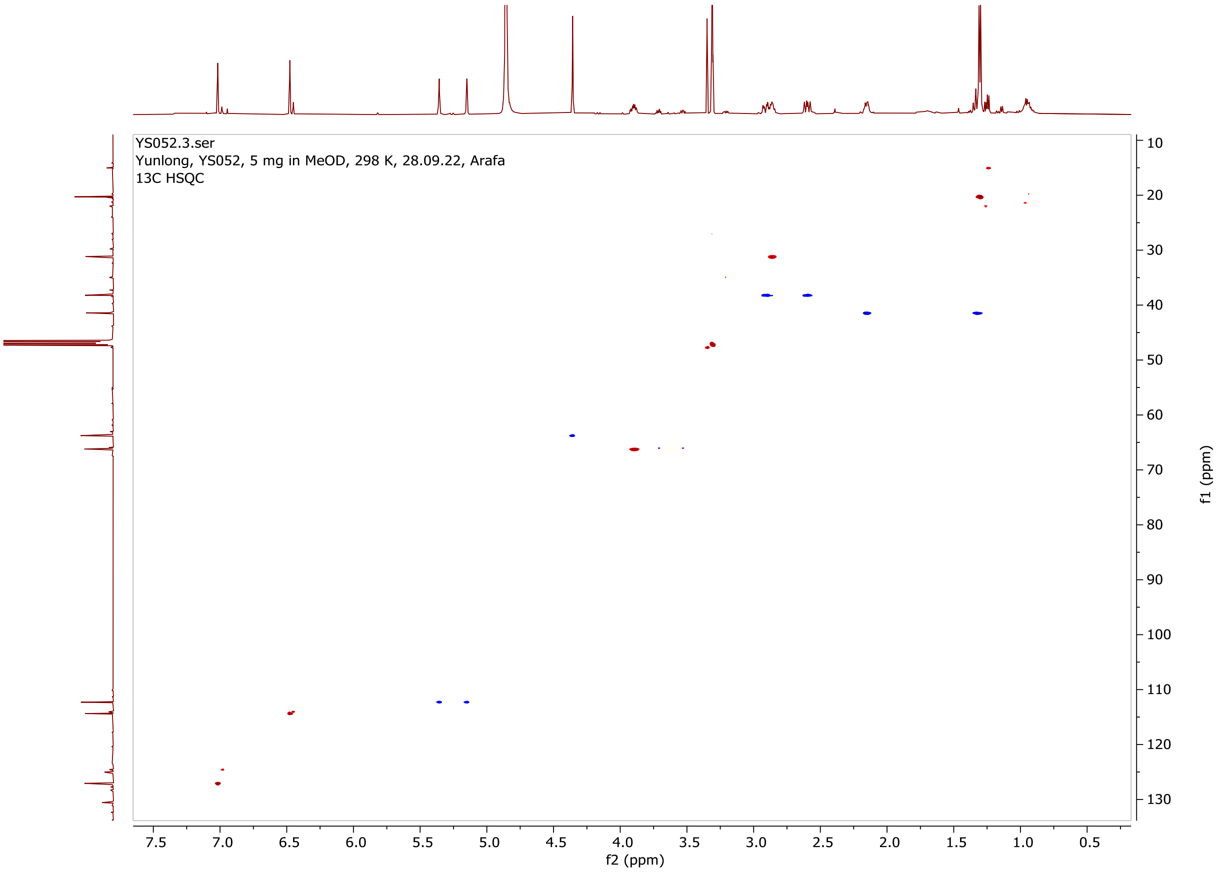

Figure S87 HSQC-spectrum of **15** recorded at 600, 150 MHz in CD_3OD

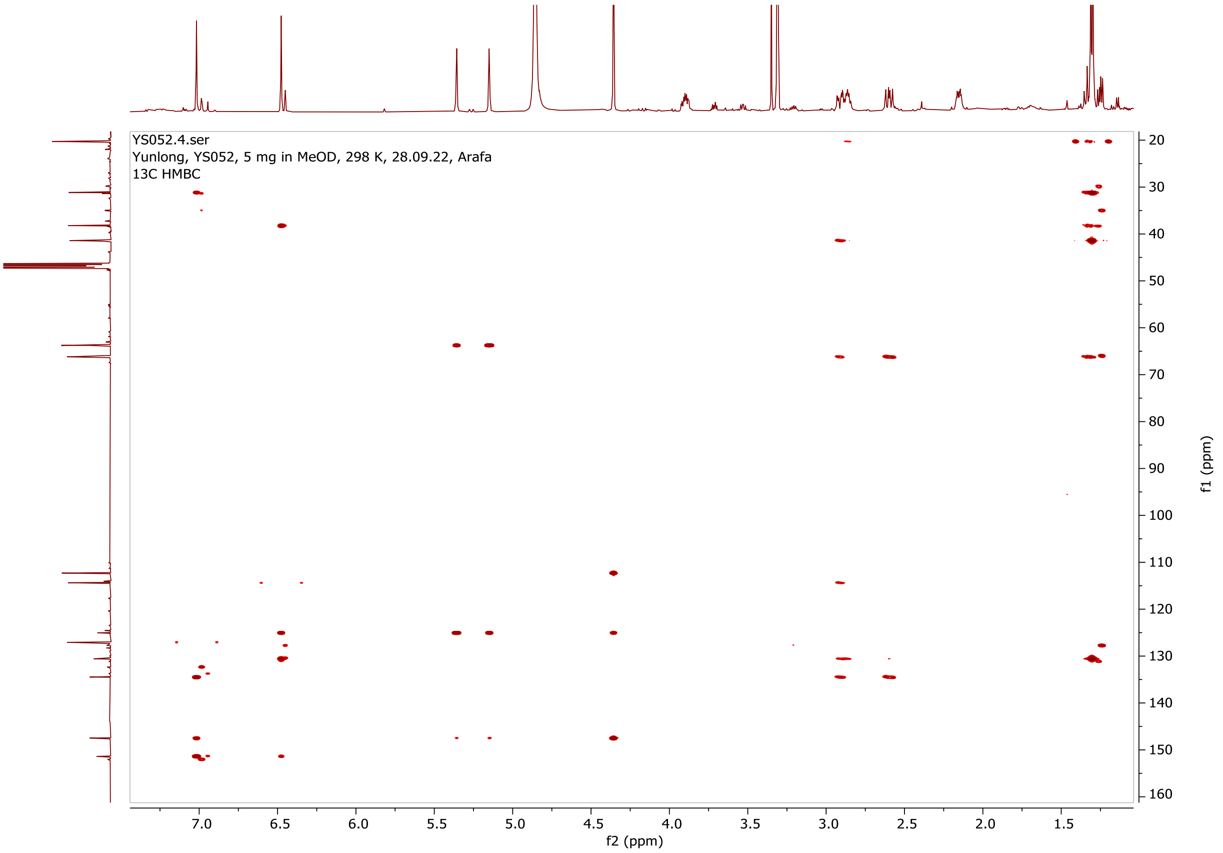

Figure S88 HMBC-spectrum of **15** recorded at 600, 150 MHz in CD_3OD

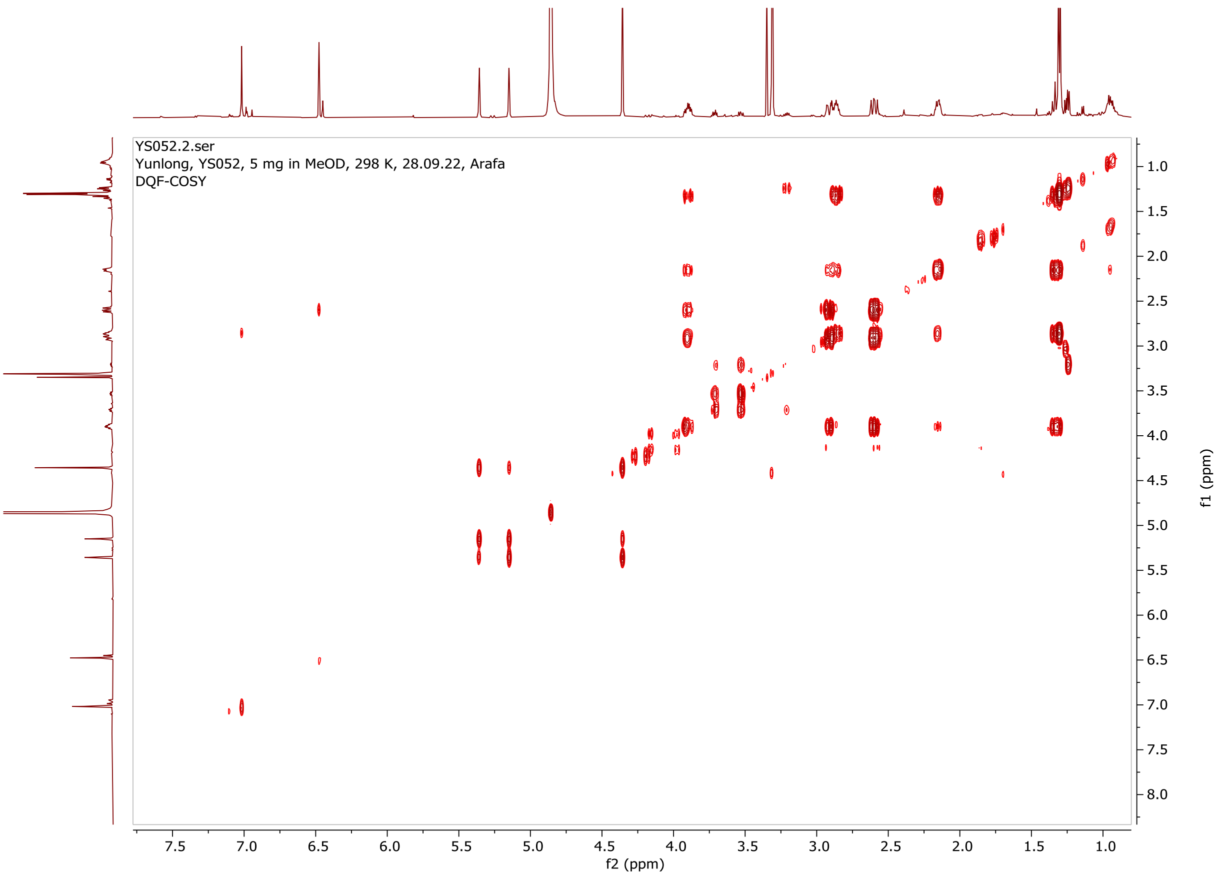

Figure S89 ^{1}H, ^{1}H-COSY-spectrum of **15** recorded at 600 MHz in CD_3OD

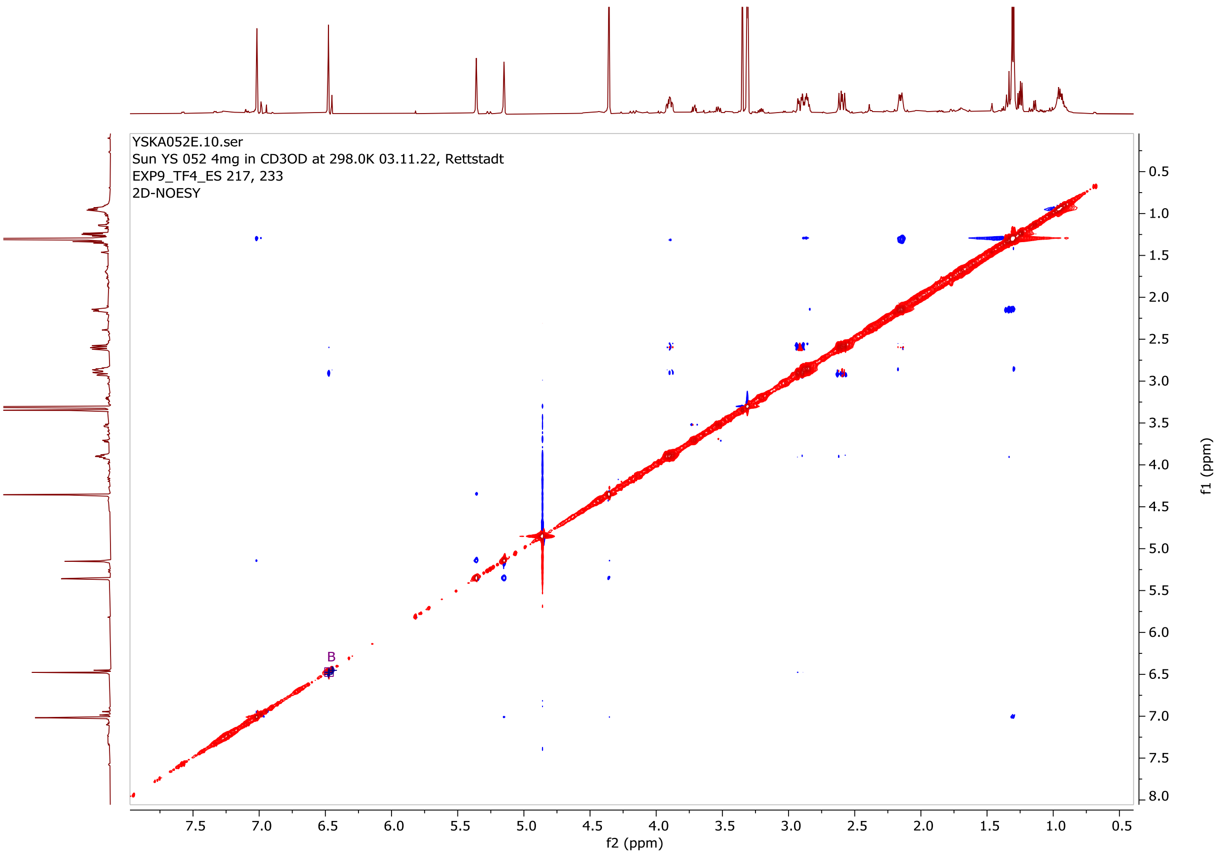

Figure S90 NOESY-spectrum of **15** recorded at 600 MHz in CD_3OD

Compound 16, 17

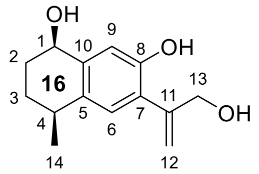

Chemical Formula: $C_{14}H_{18}O_3$
Exact Mass: 234.1256

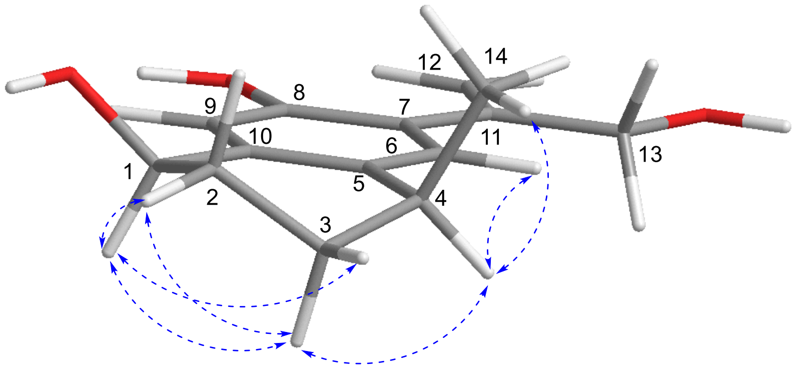

NOESY

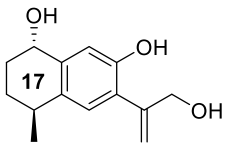

Chemical Formula: $C_{14}H_{18}O_3$
Exact Mass: 234.1256

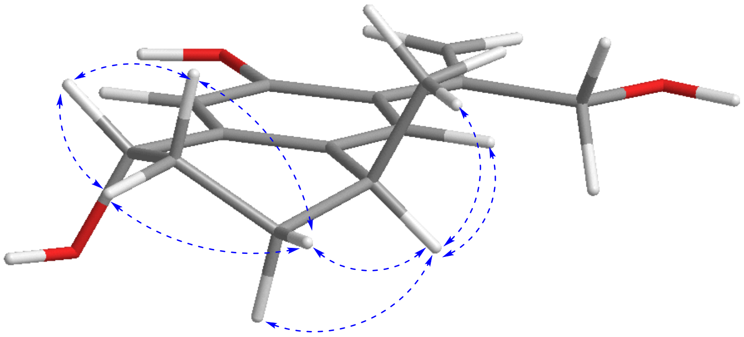

NOESY

Compounds **16** and **17** were purified and detected to be a single peak by LR-LCMS. Both of their UV (λ_{max}) were found to be 289 nm, ESI-MS m/z: $[M-H]^-$ =233, $[M-H_2O]^+$= 217. HR-ESI-MS m/z $[M-H]^-$ was found to be 233.1160, and the calculated $[M-H]^-$ was 234.1256-1.0078 = 233.1178.

The NMR showed that compounds **16** and **17** are a mixture. They were proposed to be a pair of epimers according to 1D and 2D NMR. **16** has a hydroxyl group at C-1 which is the (1*R*)-epimer, and **17** also has a hydroxyl group at C-1 which is the (1*S*)-epimer. Compared to ^{13}C-NMR of compound **15**, C-8, C-11, C-10, C-5, C-6, C-9, C-12, C-13, and C-14 are almost identical with the corresponding carbons of compounds **16, 17**. C-1, C-3 and C-4 shifted more, which was caused by the hindrance of the hydroxyl group. According to the HMBC, the proton of H-9 has coherence to C-1 at 69.1 ppm and 69.2 ppm which means the hydroxyl group is at C-1 in both epimers. According to the ^{1}H, ^{1}H-COSY, the proton of H-9 has a correlation with the proton of H-1 at 4.57 ppm. The proton of H-4 has no correlation with the proton of H-3 at 4.57 ppm. All of the above can prove that the hydroxyl group is at C-1, and there is no hydroxyl group at C-3 in each of the mixtures.

NOESY was used to classify the chemical shift of these two (1*R*) (1*S*) - epimers clearly. First, we built two structure models for those two compounds. We started from the proton of H-4 because two proton signals of H-4 are distinctly located in different chemical shifts (2.84 ppm, 2.75 ppm). By using the protons of H-4 signals in the NOESY, the position of the protons of H-3 was found. The difference in NOESY between the two compounds allows for the identification of their associated signals, which in turn allows for the distinction between the two compounds (Table S20, 21).

Compound 16				
**Pos.**	**δ_C / ppm**	**δ_H / ppm (*J*/Hz)**	**^{1}H-^{1}H COSY**	**HMBC (H-C)**
**1**	69.1	4.57, 1H, m	2, 9	3, 5, 10
**2**	31.2	1.71, 1H, m;	2, 3	1, 3, 4, 10
		2.07, 1H, m	1, 2, 3	1, 3, 4, 10
**3**	29.1	1.42, 1H, m;	2, 3, 4	1, 2, 4, 5
		2.11, 1H, m	3, 4	1, 2, 4, 5
**4**	33.1	2.84, 1H, m	3, 14	2, 3, 5, 14
**5**	134.0			
**6**	130.4	6.97, 1H, m		4, 8, 10, 11
**7**	128.5			
**8**	153.5			
**9**	115.9	6.85, 1H, d (0.8)	1	1, 5, 7, 8
**10**	140.6			
**11**	149.6			
**12**	114.5	5.16, 1H, m	12, 13	7, 11, 13
		5.37, 1H, m	12, 13	7, 11, 13
**13**	65.8	4.36, 2H, m	12	7, 11, 12
**14**	23.1	1.22, 3H, d (7.0)	4	3, 4, 5

Table S20 Summarized NMR signals for ^{13}C, ^{1}H, ^{1}H-^{1}H COSY, HMBC for **16** recorded in CD_3OD

Compound 17				
**Pos.**	**δ_C / ppm**	**δ_H / ppm (*J*/Hz)**	**^{1}H-^{1}H COSY**	**HMBC (H-C)**
**1**	69.2	4.57, 1H, m	2, 9	3, 5, 10
**2**	30.8	1.88, 2H, m	1, 3	1, 3, 4, 10
**3**	28.7	1.68, 1H, m;	2, 4	1, 2, 4, 5, 14
		1.84, 1H, m	2, 4	1, 2, 4, 14
**4**	33.0	2.75, 1H, m	3, 14	2, 3, 5, 14
**5**	134.2			
**6**	130.3	6.98, 1H, m		4, 8, 10, 11,
**7**	128.5			
**8**	153.5			
**9**	115.8	6.86, 1H, d (0.9)	1	1, 5, 7, 8
**10**	140.5			
**11**	149.6			
**12**	114.5	5.16, 1H, m;	12, 13	7, 11, 13
		5.37, 1H, m	12, 13	7, 11, 13
**13**	65.8	4.36, 2H, m		7, 11, 12
**14**	23.0	1.28, 3H, d (7.0)	4	3, 4, 5

Table S21 Summarized NMR signals for ^{13}C, ^{1}H, ^{1}H-^{1}H COSY, HMBC for **17** recorded in CD_3OD

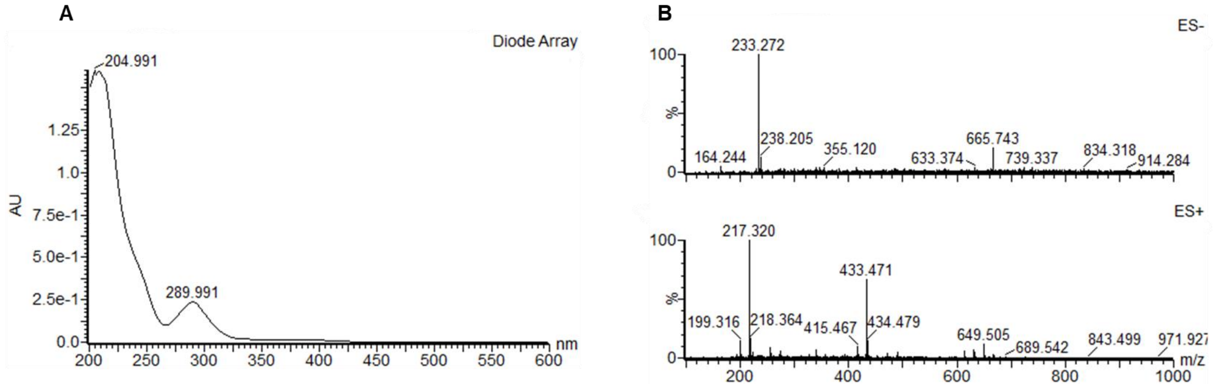

Figure S91 UV-absorption **(A)** and fragmentation pattern **(B)** of **16** and **17** in ES^+ TIC (bottom) and ES^- TIC (top) by LR-LCMS

Elemental Composition Report

Page 1

Single Mass Analysis

Tolerance = 20.0 PPM / DBE: min = -1.5, max = 50.0
Element prediction: Off
Number of isotope peaks used for i-FIT = 3

Monoisotopic Mass, Odd and Even Electron Ions
744 formula(e) evaluated with 13 results within limits (all results (up to 1000) for each mass)
Elements Used:
C: 0-95 H: 0-132 N: 0-9 O: 0-24 S: 0-4
Sun QTof Premier HAB321
YS 050 530 (5.411) AM (Cen,4, 70.00, Ht,10000.0,554.26,0.70,LS 10) 1: TOF MS ES- 2.19e+002

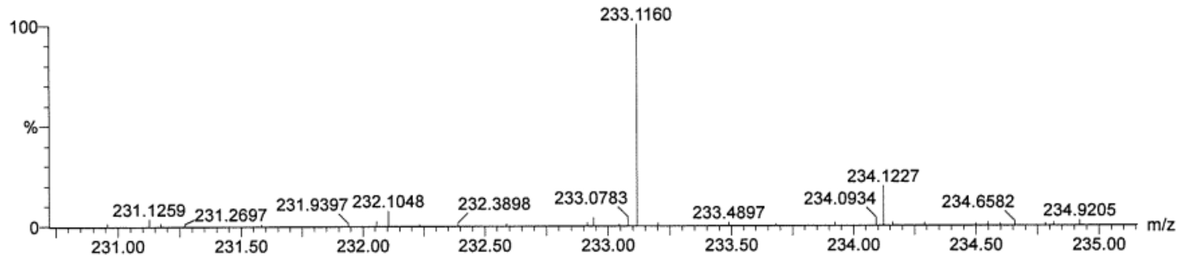

Mass	Calc. Mass	mDa	PPM	DBE	i-FIT	i-FIT (Norm)	Formula
Minimum:				-1.5			
Maximum:		5.0	20.0	50.0			
233.1160	233.1164	-0.4	-1.7	7.0	48.4	1.1	C12 H15 N3 O2
	233.1151	0.9	3.9	7.5	48.6	1.4	C10 H13 N6 O
	233.1171	-1.1	-4.7	3.0	54.3	7.1	C5 H15 N9 S
	233.1146	1.4	6.0	1.5	55.5	8.3	C10 H21 N2 S2
	233.1144	1.6	6.9	-1.5	55.5	8.3	C2 H17 N8 O3 S
	233.1178	-1.8	-7.7	6.5	48.9	1.7	C14 H17 O3

Figure S92 HRMS data for **16** and **17**; *m/z* $(M-H)^-$ calc. mass is 233.1178, 233.1160 was found

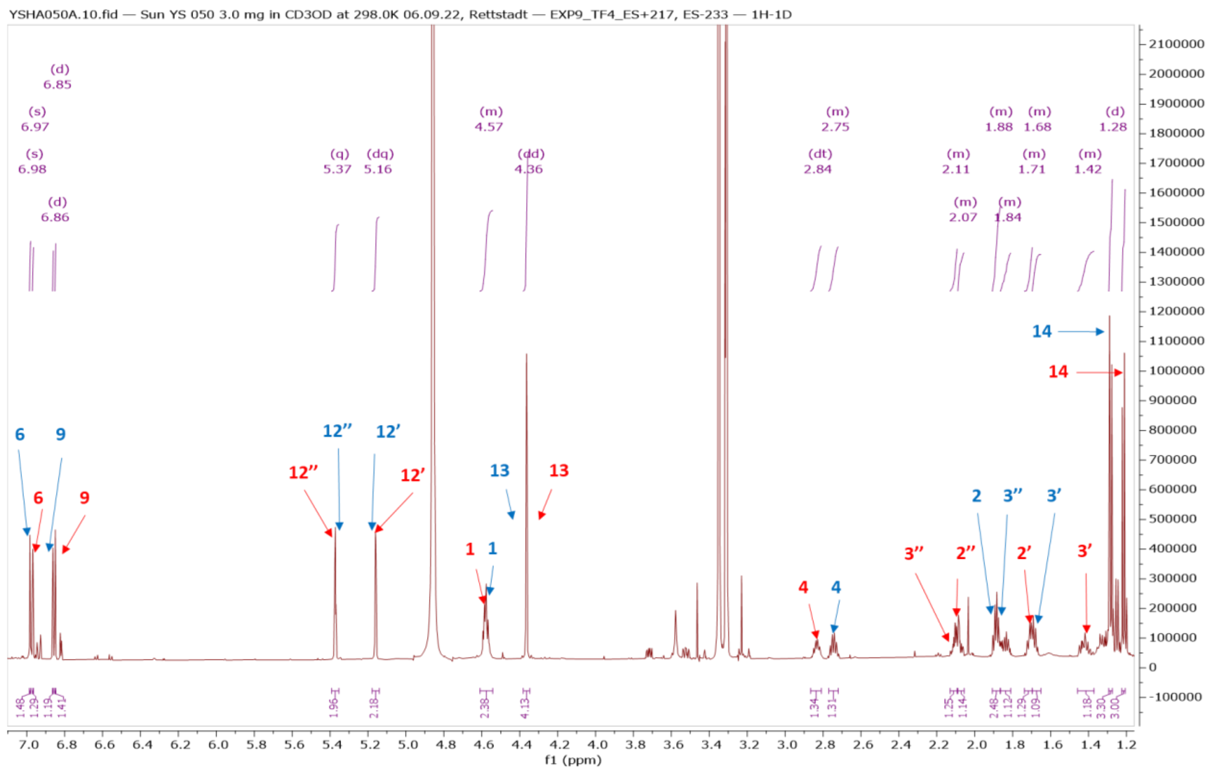

Figure S93 ^{1}H-NMR of **16** and **17** mixture recorded at 600 MHz in CD_3OD. The red numbers and arows represent the proton positions of **16**; The blue numbers and arrows represent the proton positions of **17.** The shifts labels are on the top of the integral curves; the type of the peaks are in the brackets.

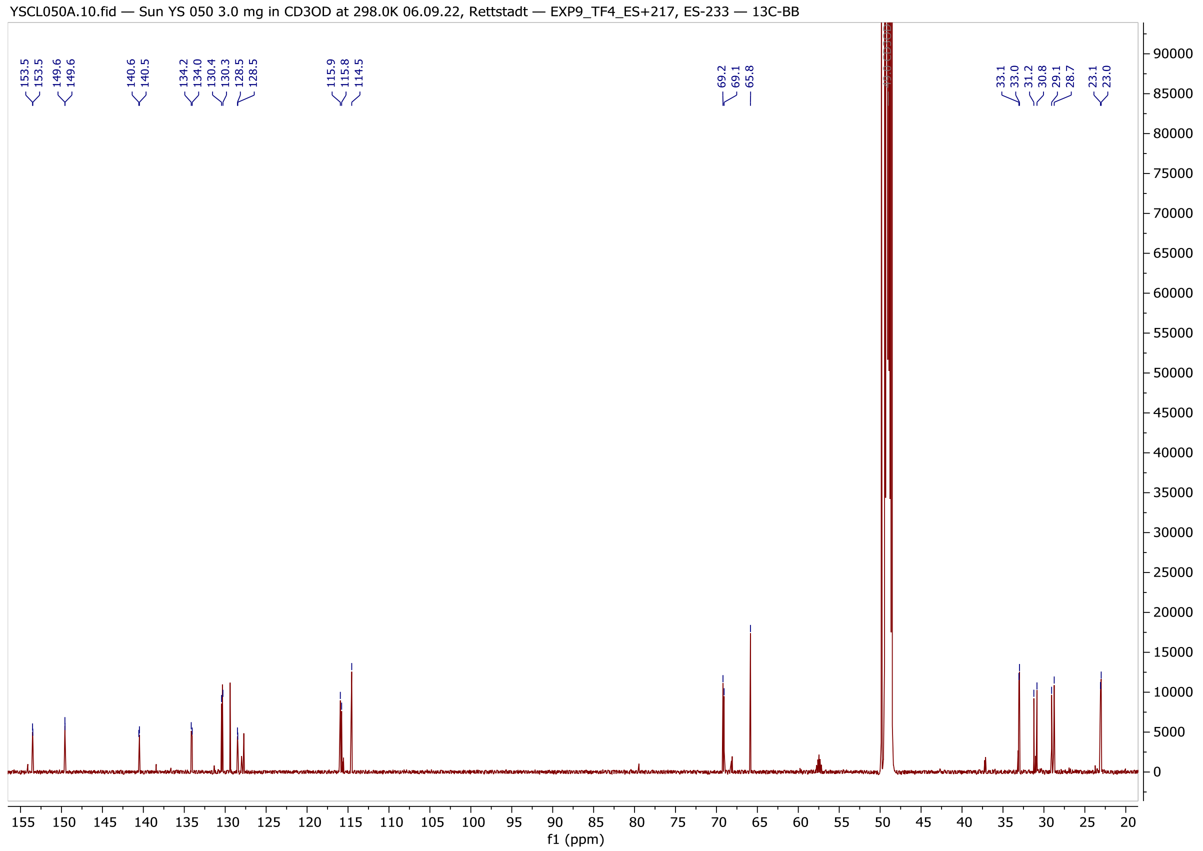

Figure S94 ^{13}C-NMR of **16** and **17** mixture recorded at 150 MHz in CD_3OD.

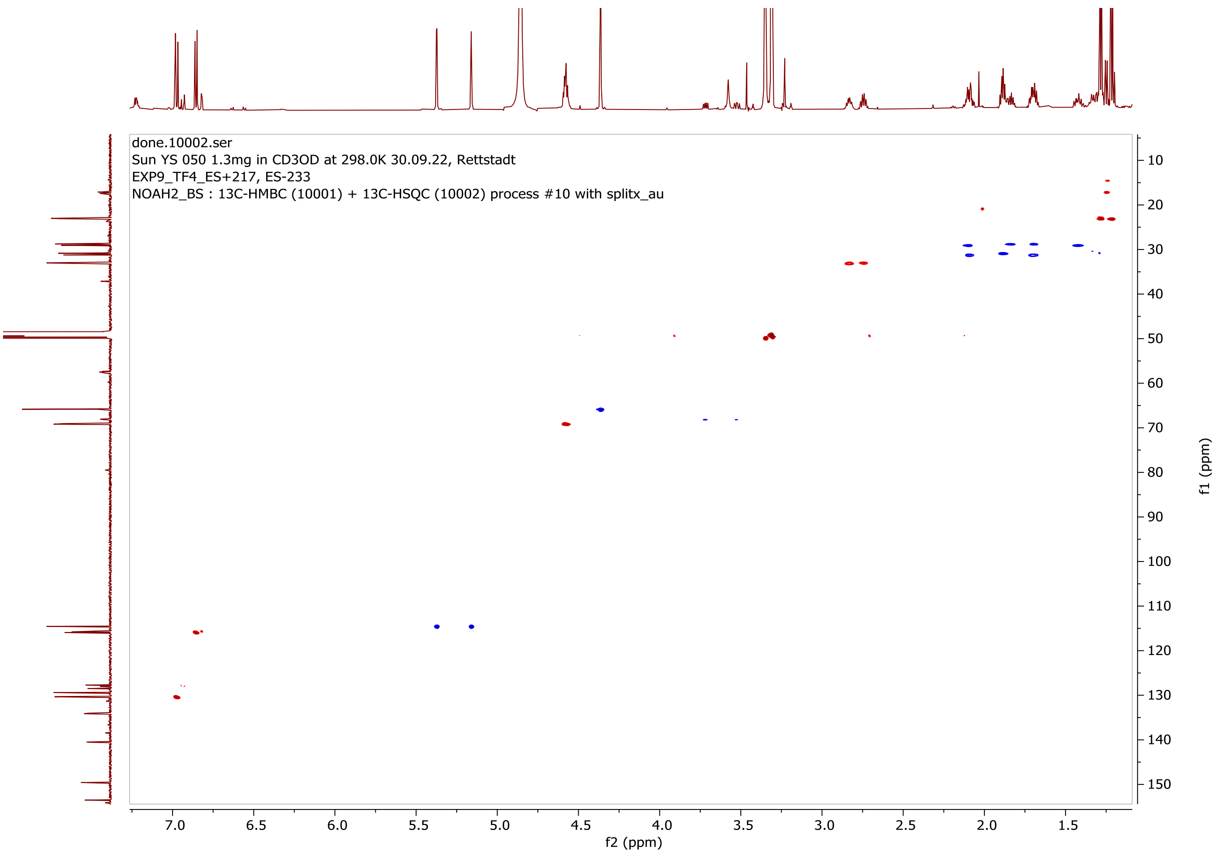

Figure S95 HSQC-spectrum of **16** and **17** mixture recorded at 600, 150 MHz in CD_3OD

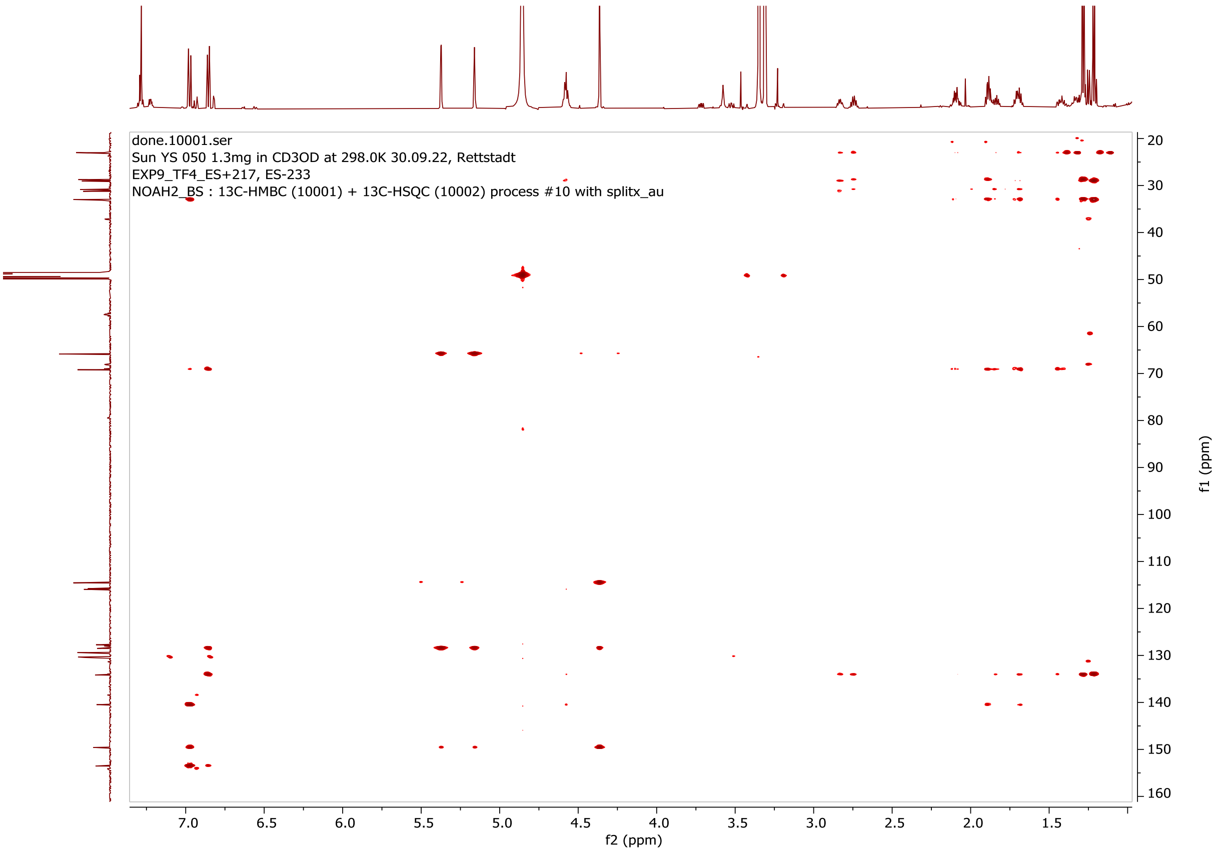

Figure S96 HMBC-spectrum of **16** and **17** mixture recorded at 600, 150 MHz in CD_3OD

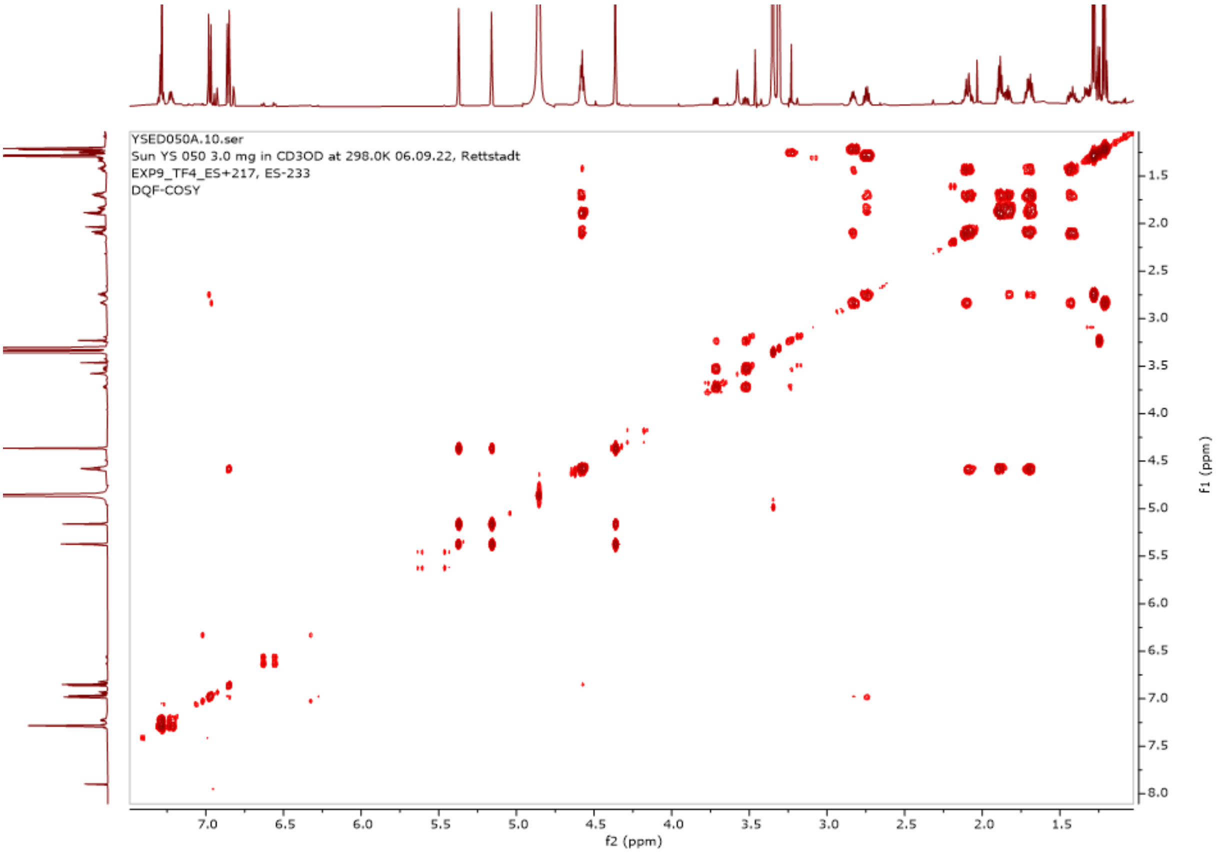

Figure S97 ^{1}H, ^{1}H-COSY-spectrum of **16** and **17** mixture recorded at 600 MHz in CD_3OD

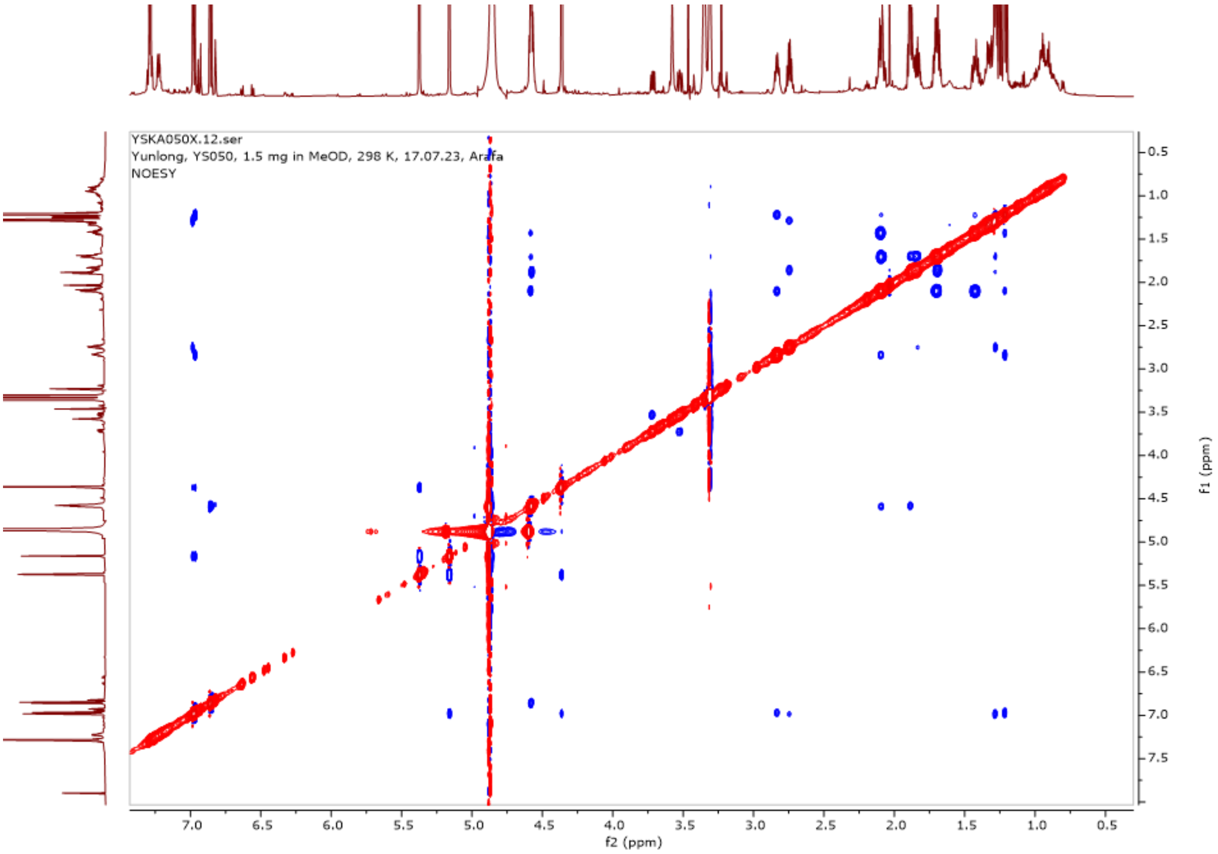

Figure S98 NOESY-spectrum of **16** and **17** mixture recorded at 600 MHz in CD_3OD

Compound 18

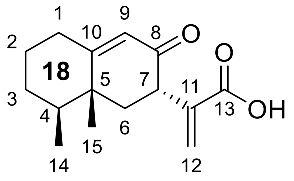

Chemical Formula: $C_{15}H_{20}O_3$
Exact Mass: 248.1412

Compound 18				
**Pos.**	***δC* / ppm**	***δH* / ppm (*J*/Hz)**	**^{1}H-^{1}H COSY**	**HMBC (H-C)**
**1**	33.2	2.32, 2H, m	1, 2, 9	2
**2**	26.4	1.46, 1H, m;	2, 1, 3	4
		1.87, 1H, m	2, 1, 3	4, 10
**3**	30.5	1.46, 1H, m;	3, 2, 4	2, 4, 5
		1.56, 1H, m	3, 2	1, 2, 4, 5
**4**	43.7	1.48, 1H, m	14	3, 5
**5**	39.9			
**6**	41.8	2.02, 2H, m	6, 7	5, 7, 8, 11, 13
**7**	45.9	3.56, 1H, dd (12.0, 6.9)	6	6, 8, 11, 12, 13
**8**	198.0			
**9**	123.7	5.78, 1H, d (1.7)	1	1, 5, 7
**10**	170.9			
**11**	139.0			
**12**	128.5	5.71, 1H, s;		7, 11, 13
		6.45, 1H, s		7, 11, 13
**13**	170.0			
**14**	15.3	0.92, 3H, d (5.7)	4	3, 4, 5
**15**	16.2	1.19, 3H, s		4, 5, 6, 10

Table S22 Summarized NMR signals for ^{13}C, ^{1}H, ^{1}H-^{1}H COSY, HMBC for **18** recorded in $CDCl_3$

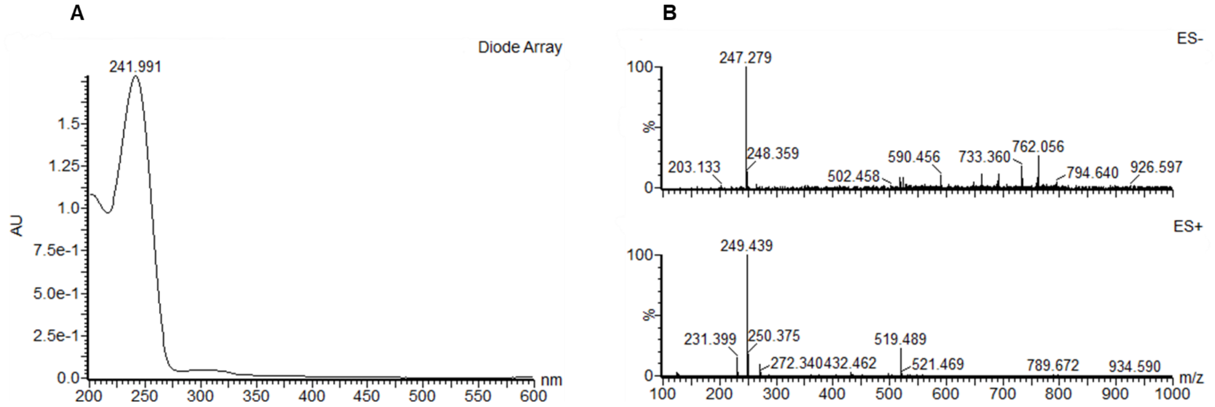

Figure S99 UV-absorption **(A)** and fragmentation pattern **(B)** of **18** in ES^{+} TIC (bottom) and ES^{-} TIC (top) by LR-LCMS

Elemental Composition Report

Page 1

Single Mass Analysis (displaying only valid results)

Tolerance = 10.0 PPM / DBE: min = -1.5, max = 50.0
Selected filters: None

Monoisotopic Mass, Odd and Even Electron Ions
114 formula(e) evaluated with 3 results within limits (up to 40 closest results for each mass)
Elements Used:
C: 0-40 H: 0-85 N: 0-6 O: 0-4

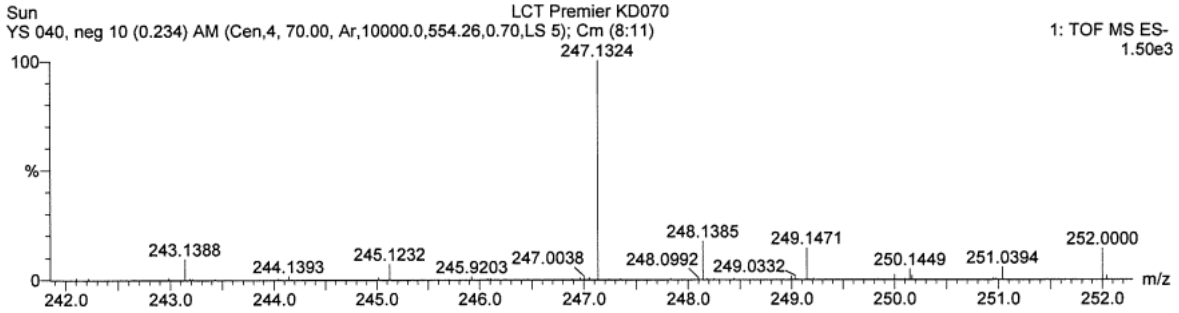

Minimum:				-1.5		
Maximum:		5.0	10.0	50.0		
Mass	Calc. Mass	mDa	PPM	DBE	i-FIT	Formula
247.1324	247.1321	0.3	1.2	7.0	84.7	C13 H17 N3 O2
	247.1334	-1.0	-4.0	6.5	78.8	C15 H19 O3
	247.1307	1.7	6.9	7.5	92.3	C11 H15 N6 O

Figure S100 HRMS data for **18**; *m/z* (M-H)$^{-}$ calc. mass is 247.1334, 247.1324 was found

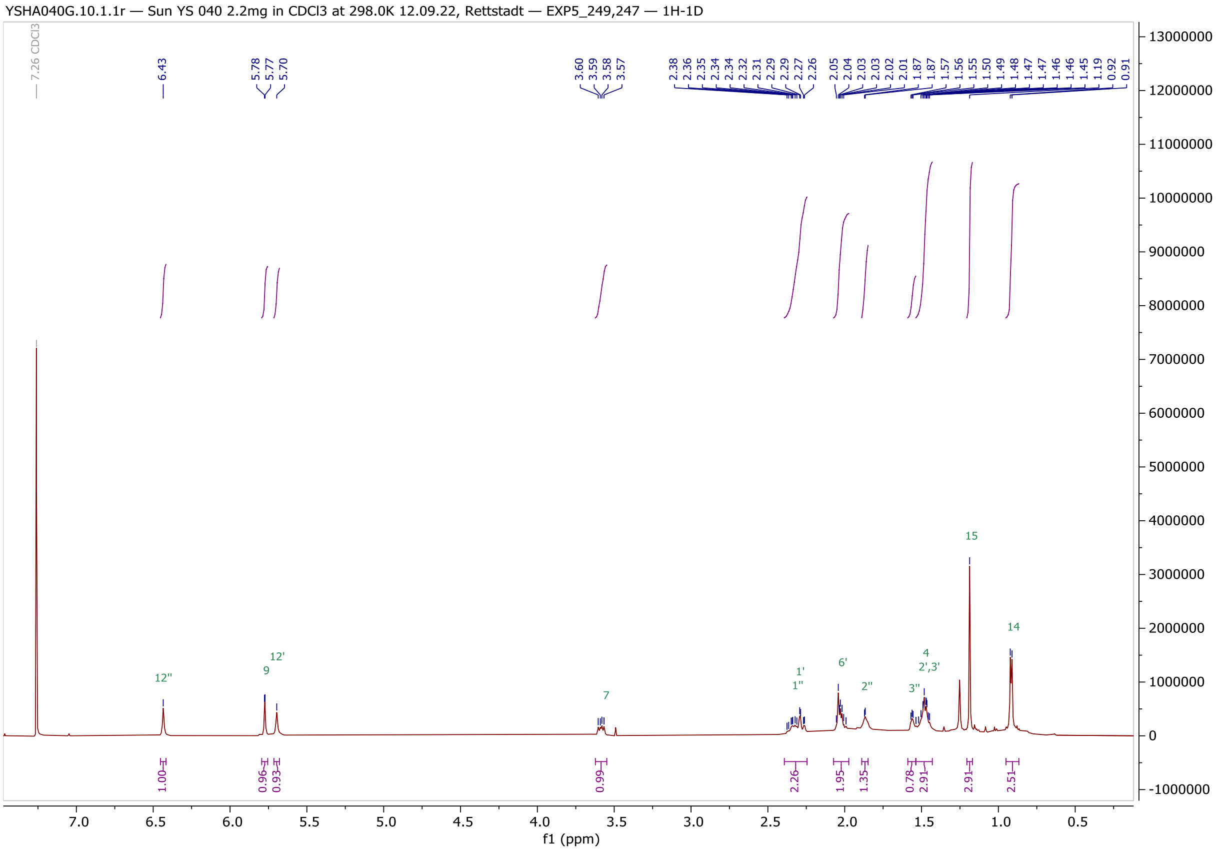

Figure S101 ^{1}H-NMR of **18** recorded at 500 MHz in $CDCl_3$

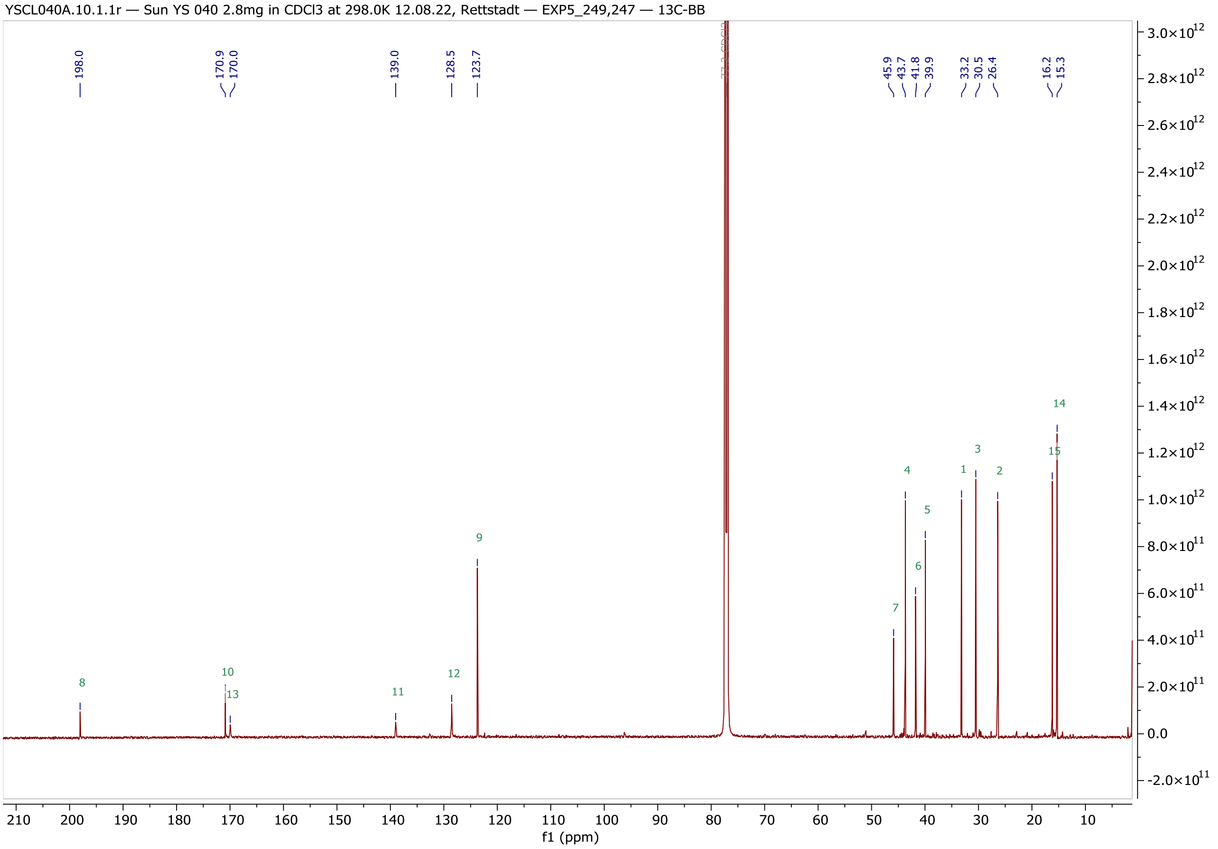

Figure S102 ^{13}C-NMR of **18** recorded at 150 MHz in $CDCl_3$

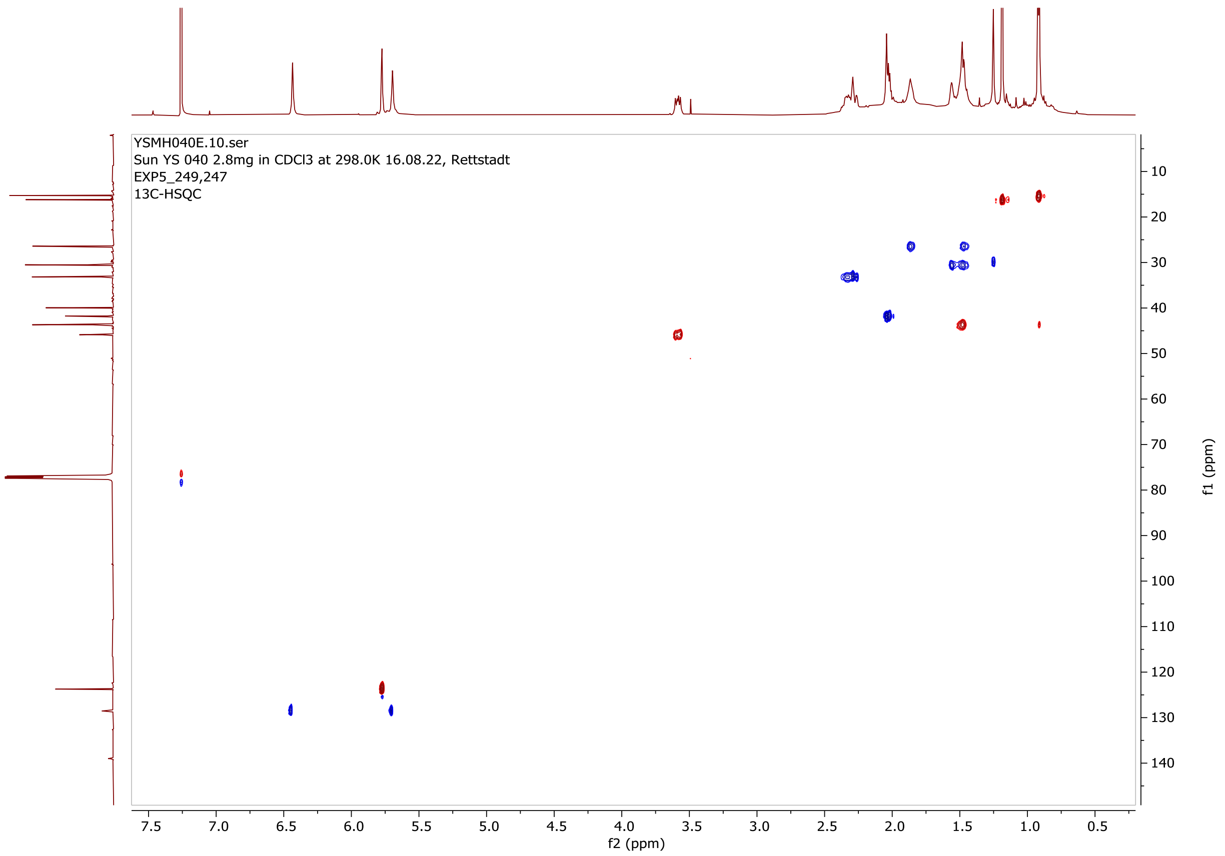

Figure S103 HSQC-spectrum of **18** recorded at 500, 125 MHz in $CDCl_3$

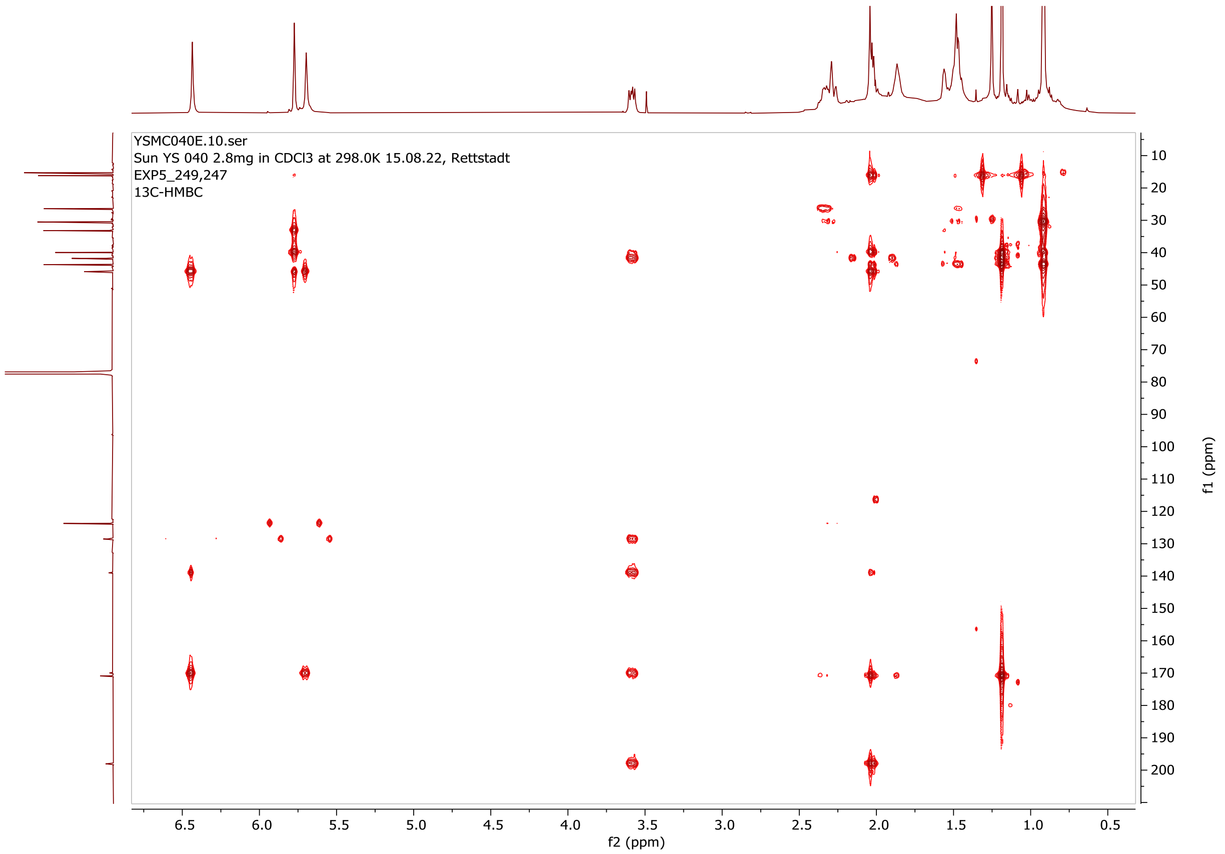

Figure S104 HMBC-spectrum of **18** recorded at 500, 125 MHz in $CDCl_3$

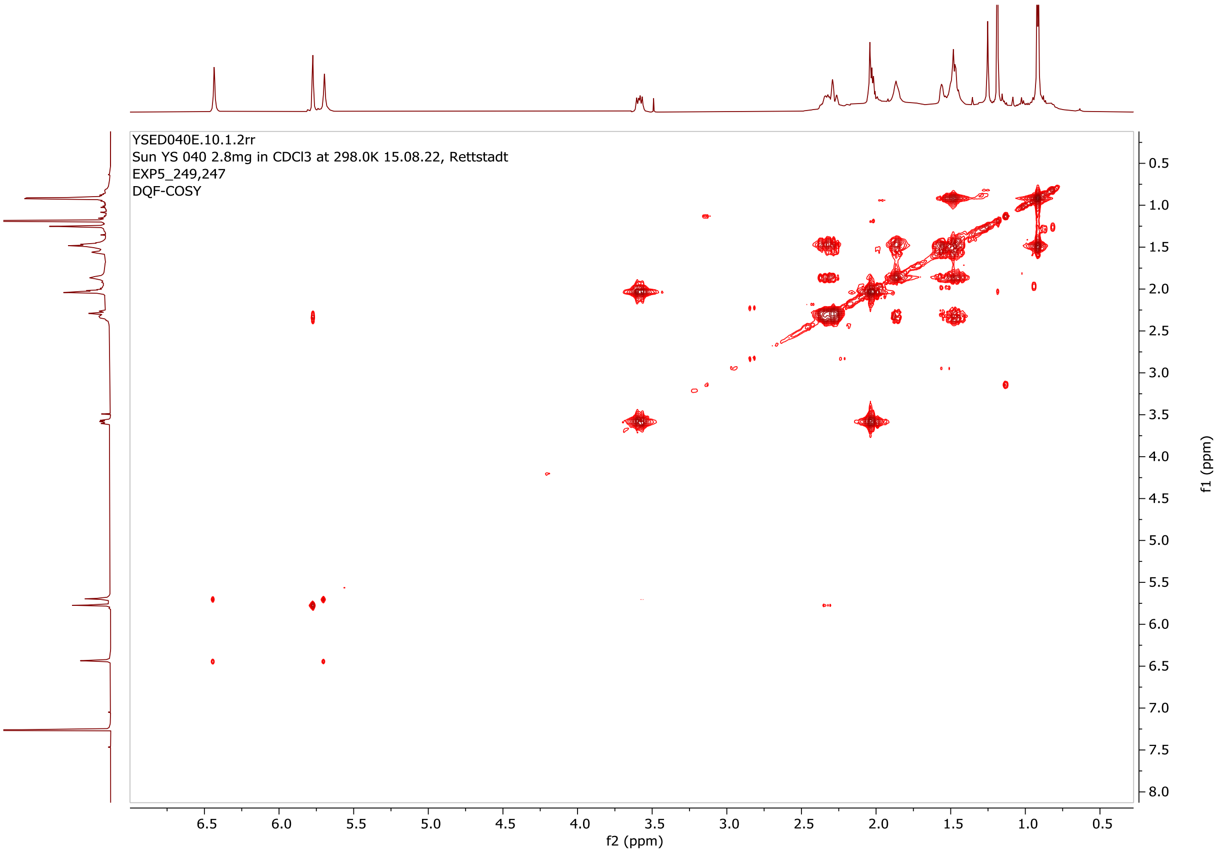

Figure S105 ^{1}H, ^{1}H-COSY-spectrum of **18** recorded at 500 MHz in $CDCl_3$

Compound 19

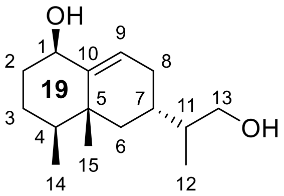

Chemical Formula: $C_{15}H_{26}O_2$
Exact Mass: 238.1933

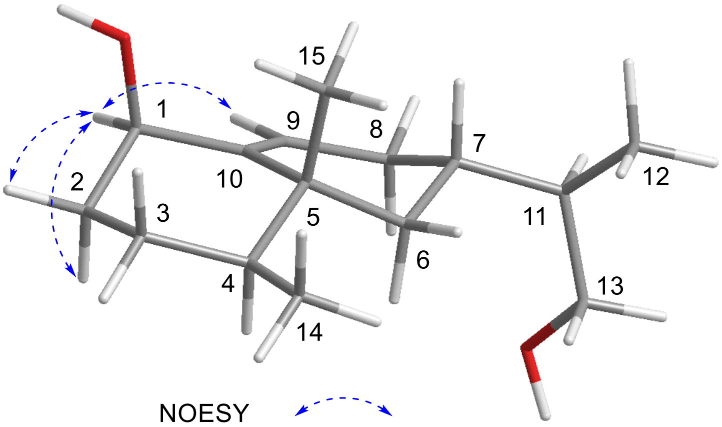

Compound 19				
**Pos.**	***δ*** δ_C **/ ppm**	δ_H **/ ppm (J/Hz)**	**^{1}H-^{1}H COSY**	**HMBC (H-C)**
**1**	74.9	4.21, 1H, dd (3.0, 3.0)	2	3, 9
**2**	33.7	1.56, 1H, m;	2, 3, 4	3, 4
		1.89, 1H, dddd (13.8, 2.8, 2.8, 2.7)	2, 3, 1	1, 2, 3
**3**	25.6	1.31, 1H, m;	2, 3	1, 2, 4, 5
		1.79, 1H, m	2, 3, 4	2, 4
**4**	43.9	1.31, 1H, m	3, 14	5, 15
**5**	38.0			
**6**	43.1	1.01, 1H, dd (12.2, 12.2);	6, 7	4, 5, 7, 8, 11, 15
		1.66, 1H, m	6	5, 7, 8, 10, 11, 15
**7**	31.3	1.8, 1H, m	6, 8, 11	6, 8, 11
**8**	28.4	1.76, 1H, m;	8, 7, 9	6, 7, 9, 10
		2.02, 1H, m	8, 7, 9	6, 7, 9, 10
**9**	125.9	5.6, 1H, dd (5.3, 2.1)	8	1, 5, 7, 8
**10**	145.3			
**11**	40.5	1.59, 1H, m	7, 12, 13	6, 7, 8, 12, 13
**12**	13.1	0.93, 3H, d (6.9)	11	7, 11, 13
**13**	66.6	3.51, 1H, dd (10.5, 6.7);	13, 11	7, 11, 12
		3.64, 1H, dd (10.5, 5.7)	13, 11	7, 11, 12
**14**	15.8	0.9, 3H, d (6.7)	4	3, 4, 5
**15**	21.0	1.13, 3H, s		4, 5, 6, 10

Table S23 Summarized NMR signals for ^{13}C, ^{1}H, ^{1}H-^{1}H COSY, HMBC for **19** recorded in $CDCl_3$

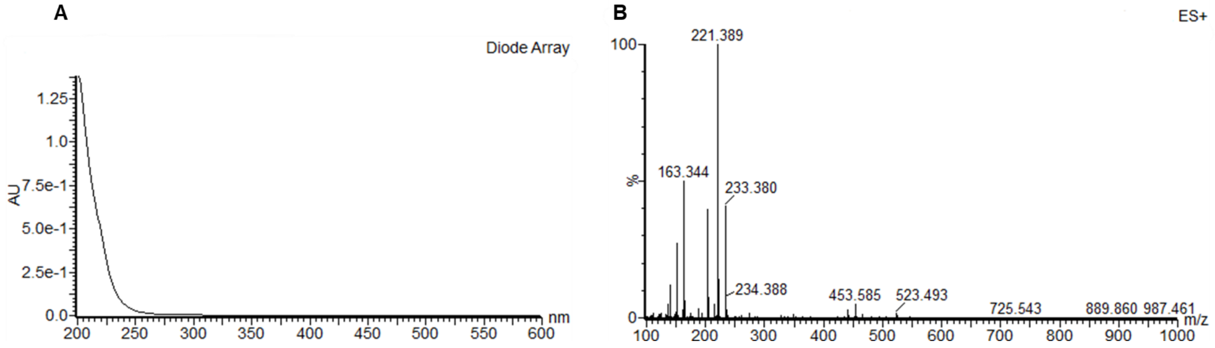

Figure S106 UV-absorption **(A)** and fragmentation pattern **(B)** of **19** in ES$^+$ TIC by LR-LCMS

Elemental Composition Report **Page 1**

Single Mass Analysis
Tolerance = 20.0 PPM / DBE: min = -1.5, max = 50.0
Element prediction: Off
Number of isotope peaks used for i-FIT = 3

Monoisotopic Mass, Even Electron Ions
28 formula(e) evaluated with 2 results within limits (all results (up to 1000) for each mass)
Elements Used:
C: 0-31 H: 0-45 O: 0-3 Na: 0-1
Sun QTof Premier HAB321
YS 062 745 (7.625) AM (Cen,4, 80.00, Ht,10000.0,556.28,0.70,LS 10) 1: TOF MS ES+ 1.30e+001

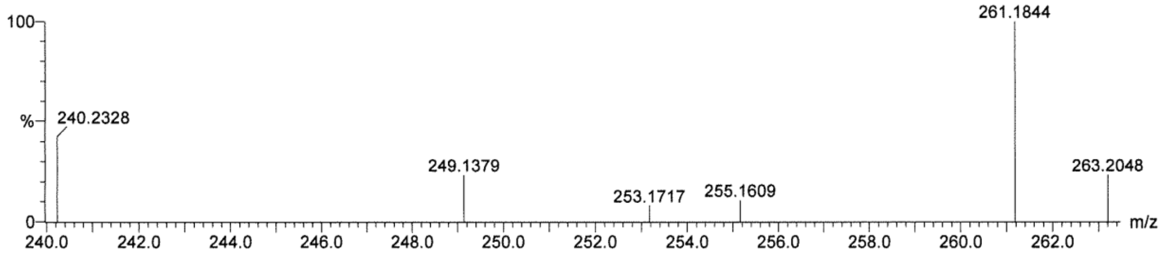

Minimum:				-1.5			
Maximum:		5.0	20.0	50.0			
Mass	Calc. Mass	mDa	PPM	DBE	i-FIT	i-FIT (Norm)	Formula
261.1844	261.1855	-1.1	-4.2	5.5	10.1	0.6	C17 H25 O2
	261.1831	1.3	5.0	2.5	10.2	0.8	C15 H26 O2 Na

Figure S107 HRMS data for **19**; *m/z* (M + Na)$^+$ calc. mass is 261.1855, 261.1844 was found

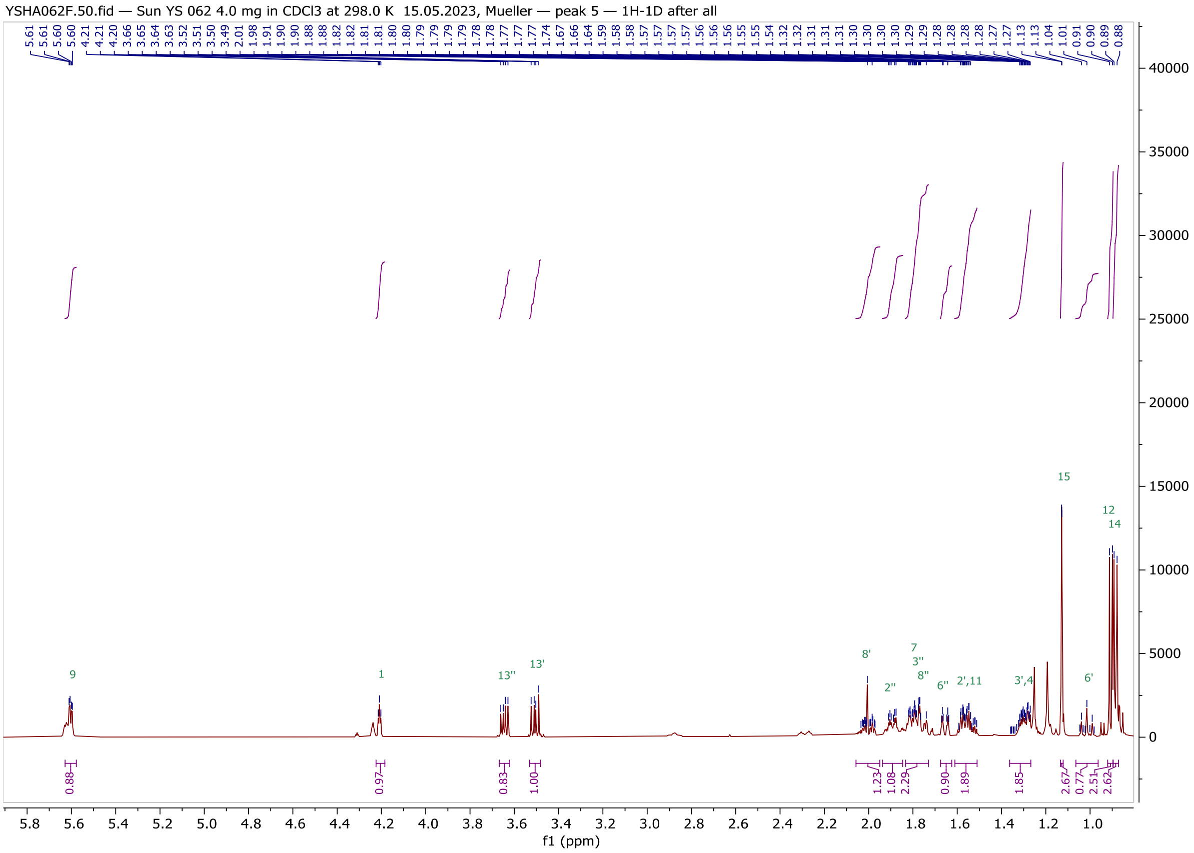

Figure S108 ^{1}H-NMR of **19** recorded at 500 MHz in $CDCl_3$

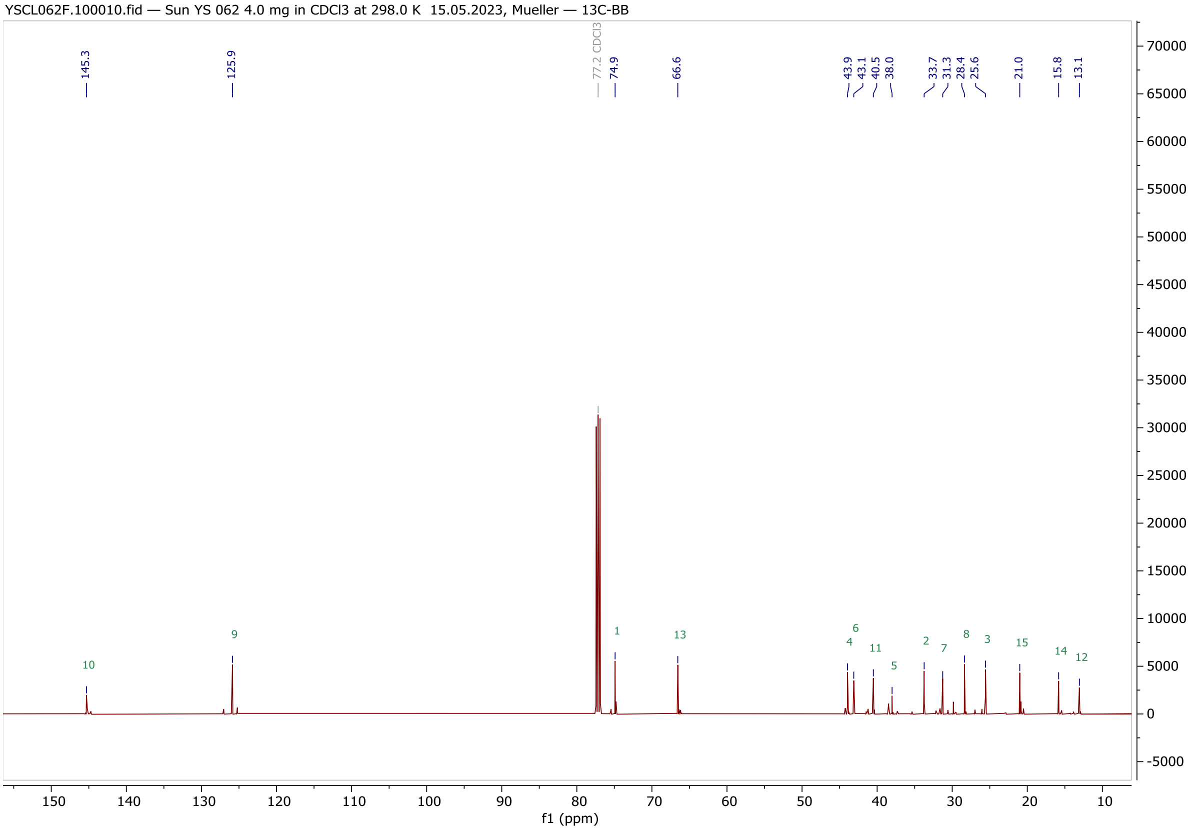

Figure S109 ^{13}C-NMR of **19** recorded at 125 MHz in $CDCl_3$

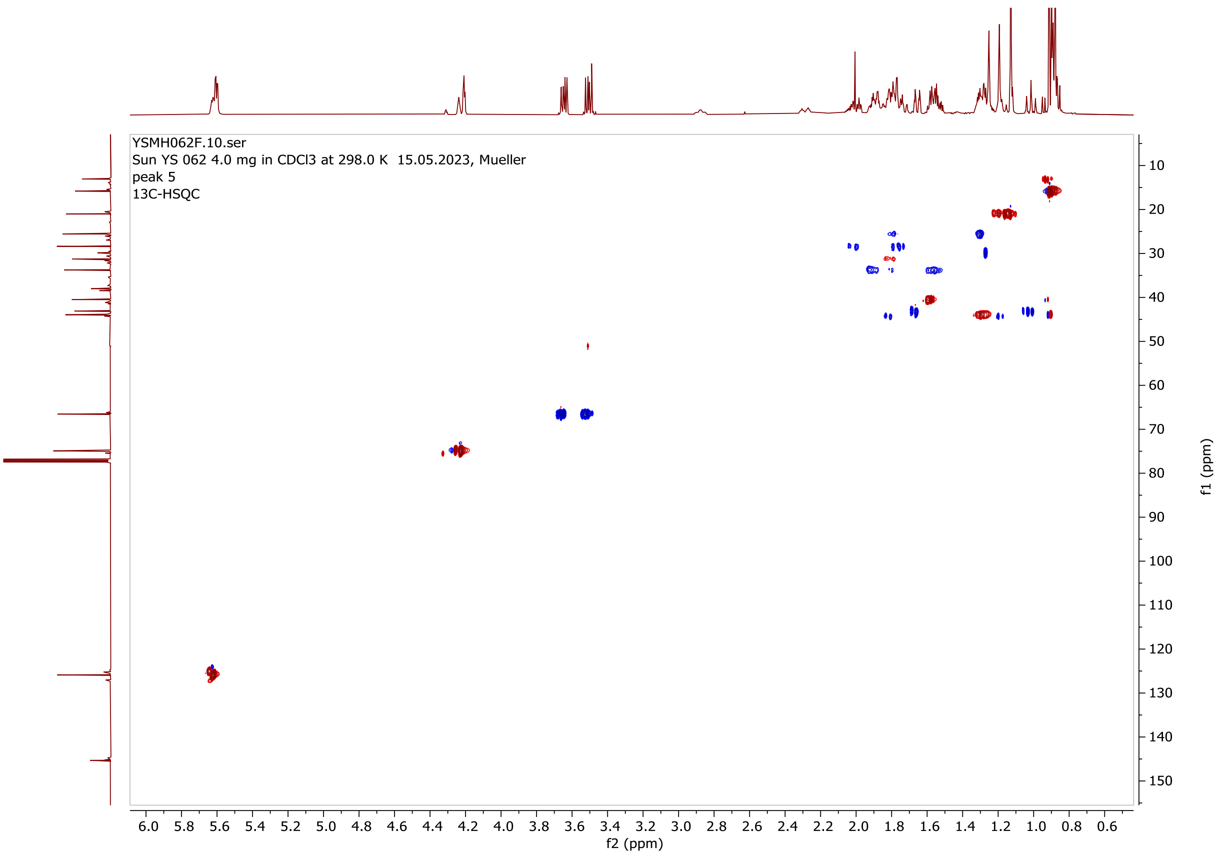

Figure S110 HSQC-spectrum of **19** recorded at 500, 125 MHz in $CDCl_3$

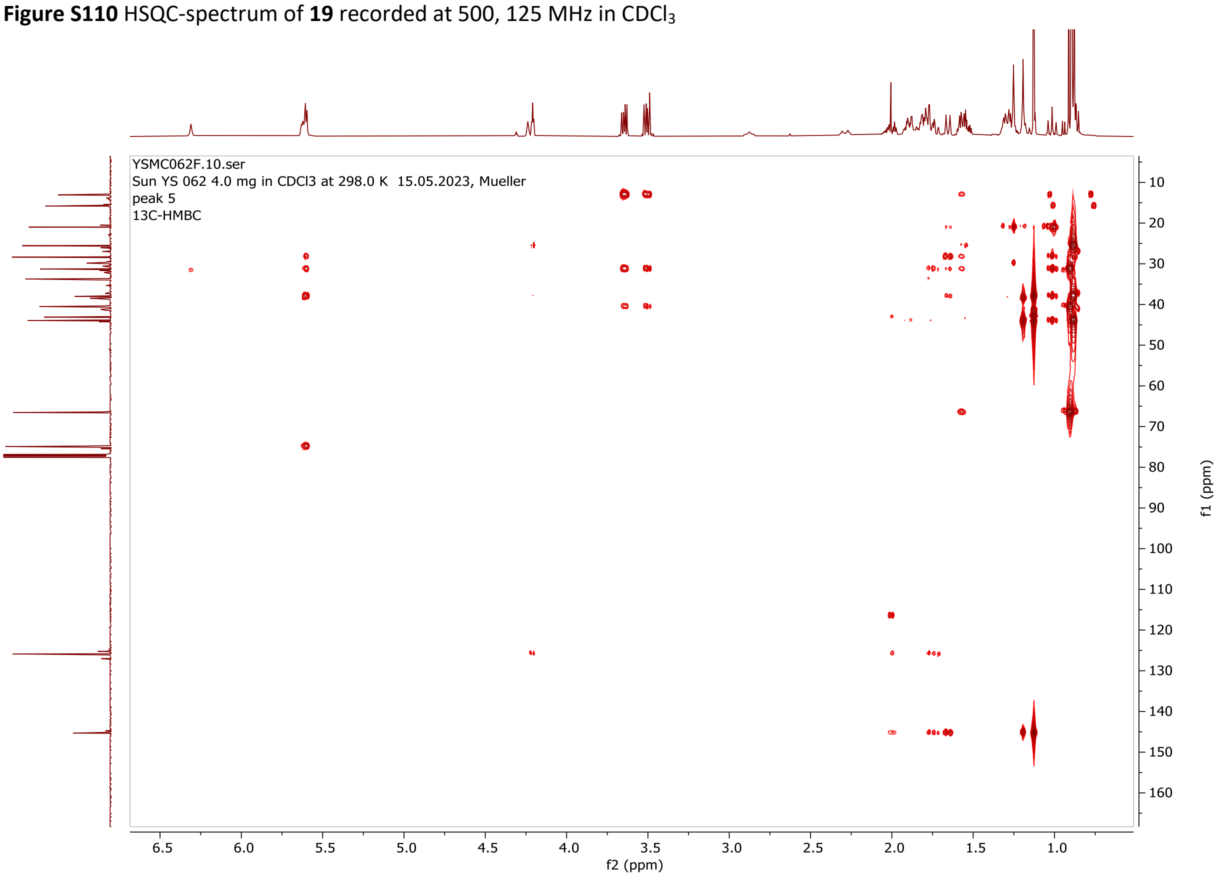

Figure S111 HMBC-spectrum of **19** recorded at 500, 125 MHz in $CDCl_3$

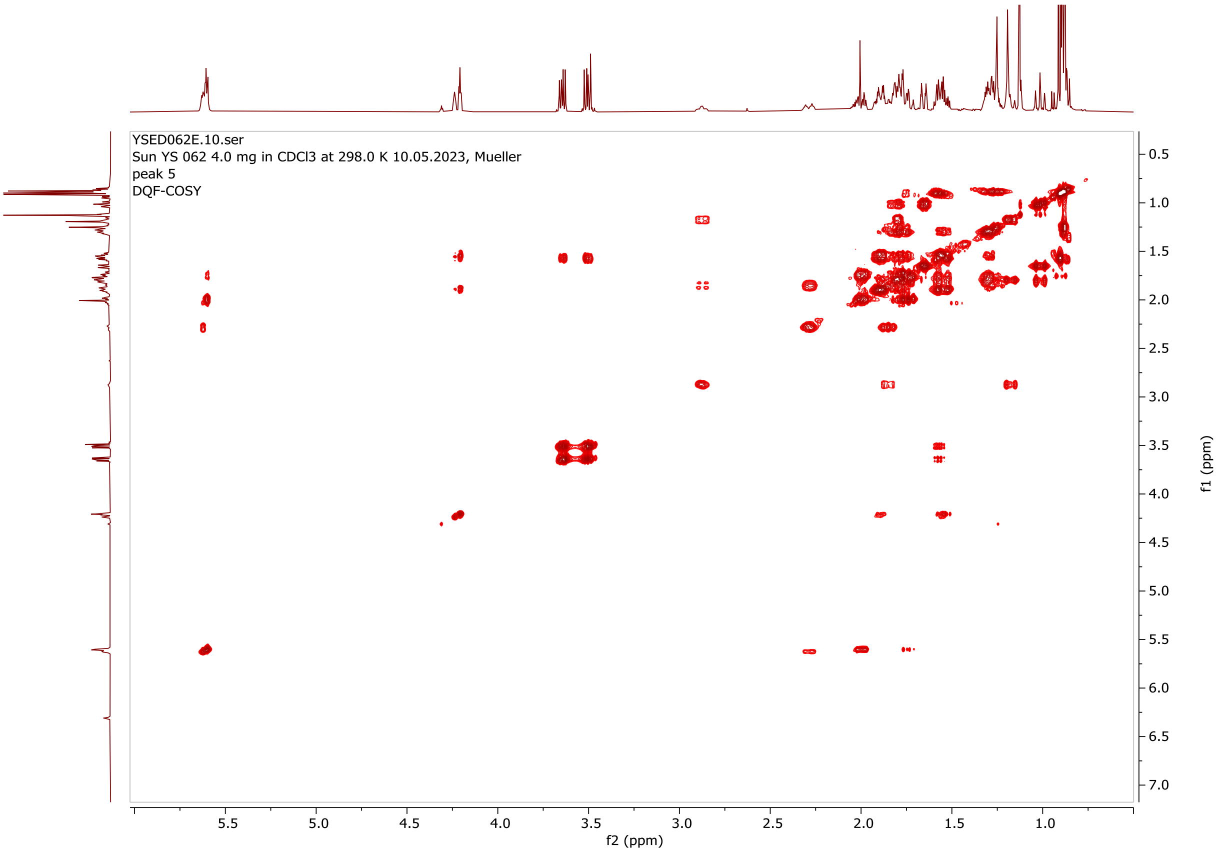

Figure S112 ^{1}H, ^{1}H-COSY-spectrum of **19** recorded at 500 MHz in $CDCl_3$

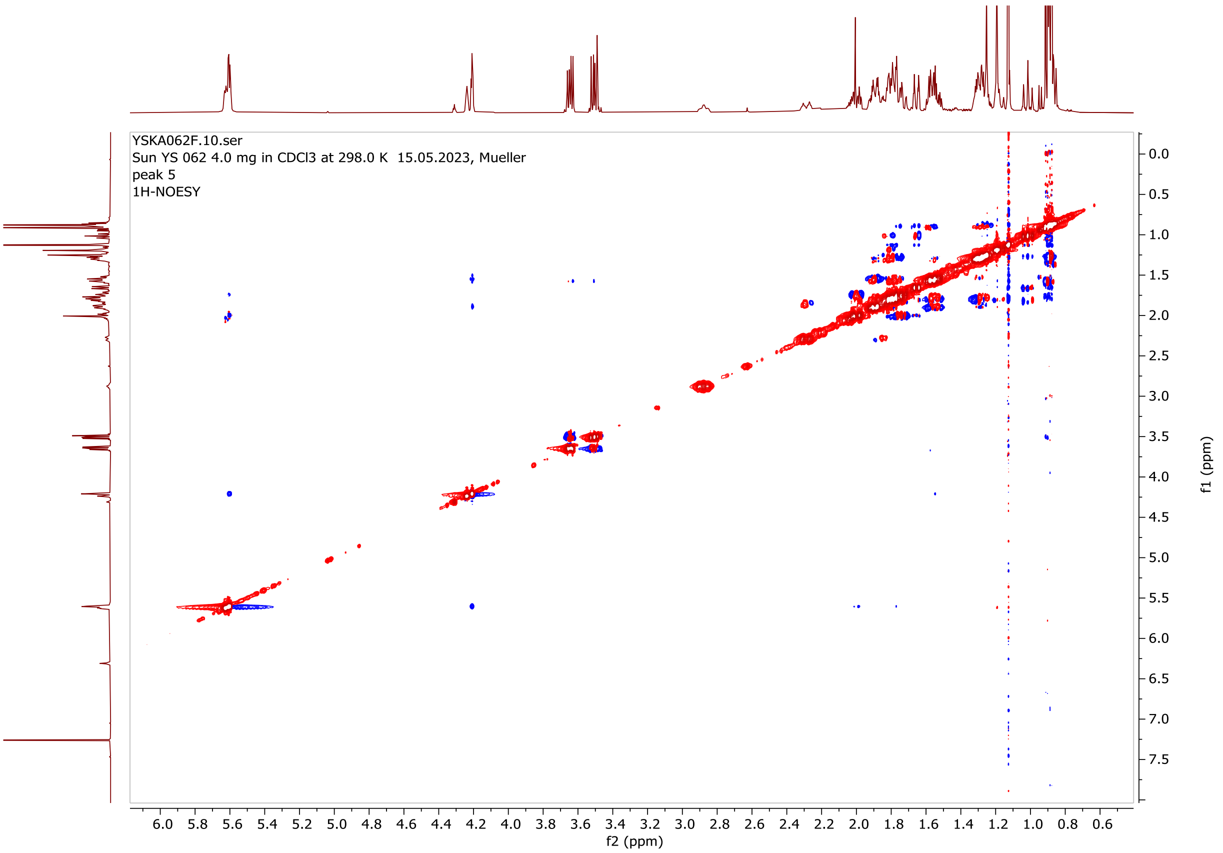

Figure S113 NOESY-spectrum of **19** recorded at 500 MHz in $CDCl_3$

Compound 20, 21

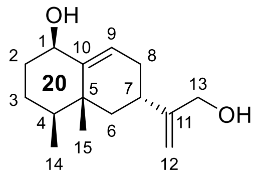

Chemical Formula: $C_{15}H_{24}O_2$
Exact Mass: 236.1776

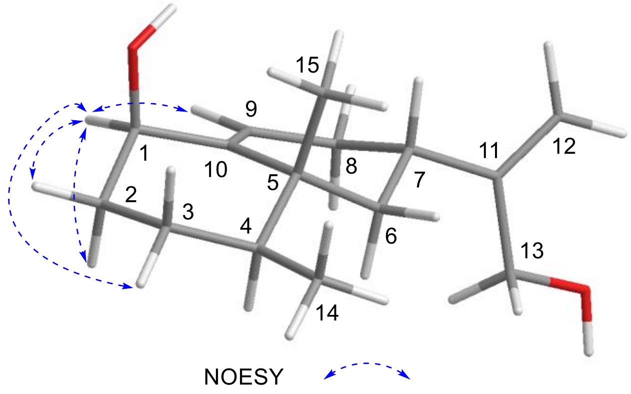

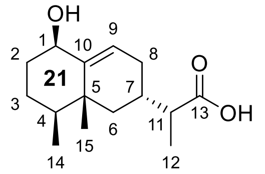

Chemical Formula: $C_{15}H_{24}O_3$
Exact Mass: 252.1725

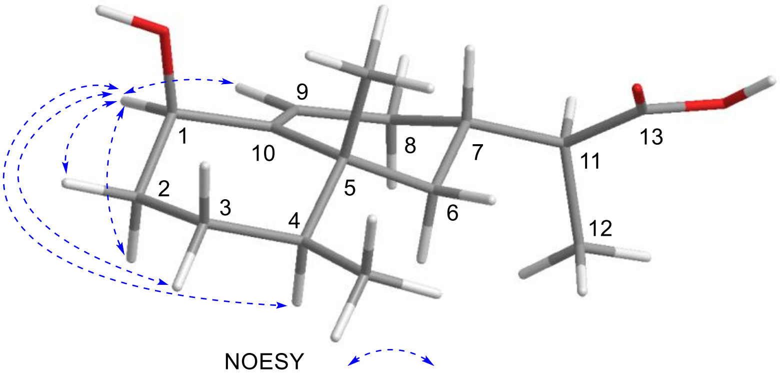

The NMR of compounds **20** and **21** were elucidated to be a mixture, where **20** has a hydroxyl group at C-13, **21** has a keto group at C-13. The ^{13}C NMR, ^{1}H NMR, and 2D NMR of **20** were assigned first (Table S24). According to protons of H-13 of **20** in HMBC, the ^{13}C shifts of C-11, C-12 and C-7 were deduced and also the proton shifts of H-7 and H-12 were found by HSQC. According to ^{1}H, ^{1}H-COSY of proton of H-7, the proton shifts of H-8 and H-6 were found, subsequently.The ^{13}C shifts of C-8 and C-6 were found by HSQC. The proton shift of H-9 was found according to the proton shift of H-8 by ^{1}H, ^{1}H-COSY. The proton shift of H-1 was found using ^{1}H, ^{1}H-COSY of the proton shift of H-9. The proton shifts of H-2, H-3 and H-4 were subsequently found. According to the HSQC, their ^{13}C shifts also were found.

The stereochemical configuration of the hydroxyl group at C-1 was deduced using their coupling constants. The coupling constants of the proton at C-1 of compounds **20** and **21** were all J = 3.0, 3.0 Hz, which means the dihedral angle of 2H'-1H-2H'' is about 60 °according to the Karplus relationship. It was deduced that both of those two compounds have an (R) configuration at C-1, which means the hydrogen is on the bottom of the plane. The information from NOESY was used to confirm this deduction (Figure S124).

Compound 20				
**Pos.**	**δ_C / ppm**	**δ_H / ppm (*J*/Hz)**	**^{1}H-^{1}H COSY**	**HMBC (H-C)**
**1**	74.9	4.23, 1H, dd (3.0, 3.0)	2	3, 5, 9
**2**	33.7	1.55, 1H, m;	2, 1, 3	3, 4
		1.90, 1H, m	2, 1, 3	3, 4, 10
**3**	25.6	1.29, 1H, m;	3, 2	1, 4, 5
		1.77, 1H, m	3, 2, 4	1, 4, 5
**4**	43.8	1.29, 1H, m	3, 14	6, 15
**5**	38.3			
**6**	43.9	1.21, 1H, m;	6, 7	4, 5, 15
		1.77, 1H, m	6, 7	4, 5, 15
**7**	33.5	2.40, 1H, m	6, 8	11, 12, 13
**8**	32.1	1.90, 1H, m;	8, 7, 9	6, 7, 9, 10
		2.19, 1H, m	8, 7, 9	6, 7, 9, 10
**9**	125.5	5.63, 1H, dd (5.2, 2.2)	8	1, 5, 7
**10**	145.2			
**11**	153.8			
**12**	108.3	4.92, 1H, m;	12, 13	7, 11, 13
		5.08, 1H, m	12, 13	7, 11, 13
**13**	65.4	4.15, 2H, m	12	7, 11, 12
**14**	15.8	0.89, 3H, d (6.9)	4	3, 4, 5
**15**	21.0	1.15, 3H, s		4, 5, 10

Table S24 Summarized NMR signals for ^{13}C, ^{1}H, ^{1}H-^{1}H COSY, HMBC for **20** recorded in $CDCl_3$

Compound 21				
**Pos.**	**δ_C / ppm**	**δ_H / ppm (*J*/Hz)**	**^{1}H-^{1}H COSY**	**HMBC (H-C)**
**1**	74.8	4.21, 1H, dd (3.0, 3.0)	2	3, 5, 9
**2**	33.8	1.55, 1H, m;	2, 1, 3	3, 4
		1.90, 1H, m	2, 1, 3	1, 3, 4, 10
**3**	25.5	1.29, 1H, m;	3, 2	1, 4, 5
		1.77, 1H, m	3, 2, 4	1, 4, 5
**4**	43.8	1.29, 1H, m	3, 14	2, 5, 6
**5**	38.0			
**6**	41.8	0.96, 1H, m;	6, 7	4, 5, 8, 10, 15
		1.77, 1H, m	6, 7	4, 5, 8, 10, 15
**7**	33.4	2.01, 1H, m	6, 8, 11	6, 11, 12
**8**	30.3	1.83, 1H, m;	8, 7, 9	7, 9, 10, 11
		2.11, 1H, m	8, 7, 9	6, 7, 9, 10
**9**	125.1	5.57, 1H, dd (5.3, 2.2)	8	1, 5, 7, 8
**10**	145.3			
**11**	44.3	2.32, 1H, dddd (6.9, 6.9, 6.9, 6.9)	7, 12	6, 7, 8, 12, 13
**12**	14.2	1.21, 3H, d (7.0)	11	7, 11, 13
**13**	180.2			
**14**	15.8	0.89, 3H, d (6.6)	4	3, 4, 5
**15**	21.0	1.15, 3H, s		4, 5, 10

Table S25 Summarized NMR signals for ^{13}C, ^{1}H, ^{1}H-^{1}H COSY, HMBC for **21** recorded in $CDCl_3$

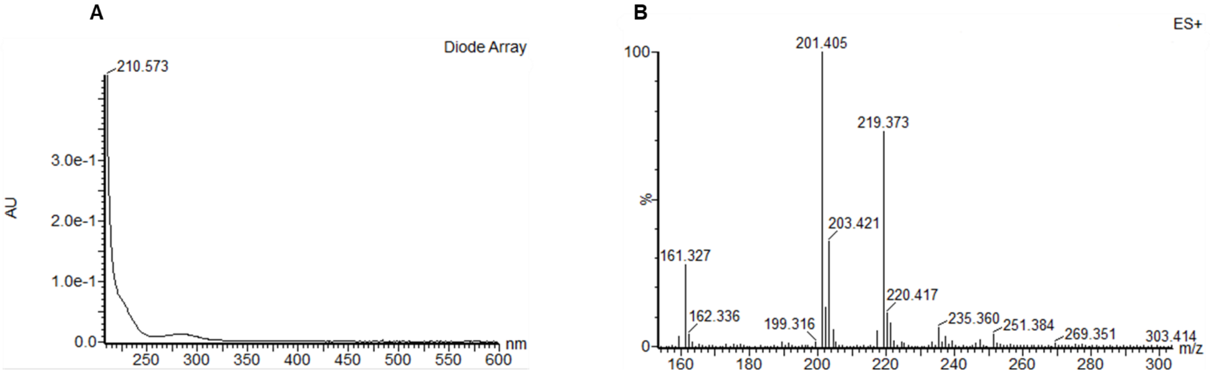

Figure S114 UV-absorption **(A)** and fragmentation pattern **(B)** of **20** in ES$^+$ TIC by LR-LCMS

Elemental Composition Report

Page 1

Single Mass Analysis

Tolerance = 20.0 PPM / DBE: min = -1.5, max = 50.0
Element prediction: Off
Number of isotope peaks used for i-FIT = 3

Monoisotopic Mass, Even Electron Ions
283 formula(e) evaluated with 2 results within limits (all results (up to 1000) for each mass)
Elements Used:
C: 0-31 H: 0-45 N: 0-4 O: 0-5 S: 0-3
Sun QTof Premier HAB321
YS 061b 672 (6.866) AM (Cen,4, 30.00, Ht,10000.0,556.28,0.70,LS 10)

1: TOF MS ES+
1.01e+002

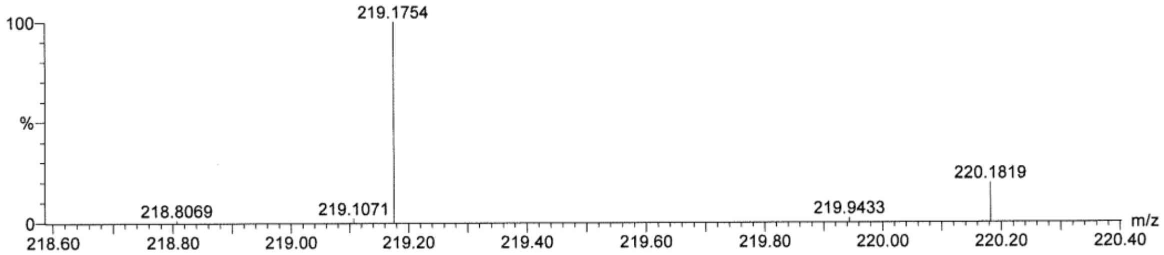

Minimum:				-1.5			
Maximum:		5.0	20.0	50.0			
Mass	Calc. Mass	mDa	PPM	DBE	i-FIT	i-FIT (Norm)	Formula
219.1754	219.1749	0.5	2.3	4.5	9.7	0.0	C15 H23 O
	219.1783	-2.9	-13.2	-0.5	14.0	4.3	C12 H27 O S

Figure S115 HRMS data for **20**; *m/z* (M-H_2O+H)$^+$ calc. mass is 219.1749, 219.1754 was found

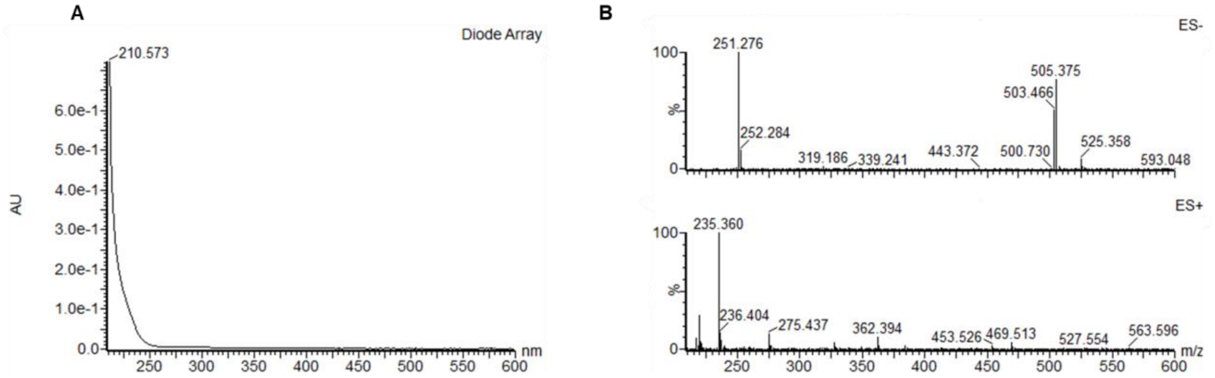

Figure S116 UV-absorption **(A)** and fragmentation pattern **(B)** of **21** in ES$^+$ TIC (bottom) and ES$^-$ TIC (top) by LR-LCMS

Elemental Composition Report **Page 1**

Single Mass Analysis (displaying only valid results)

Tolerance = 5.0 PPM / DBE: min = -1.5, max = 50.0

Selected filters: None

Monoisotopic Mass, Even Electron Ions

320 formula(e) evaluated with 1 results within limits (up to 40 closest results for each mass)

Elements Used:

C: 0-72 H: 0-50 N: 0-4 O: 0-4 Na: 0-1 Si: 0-1

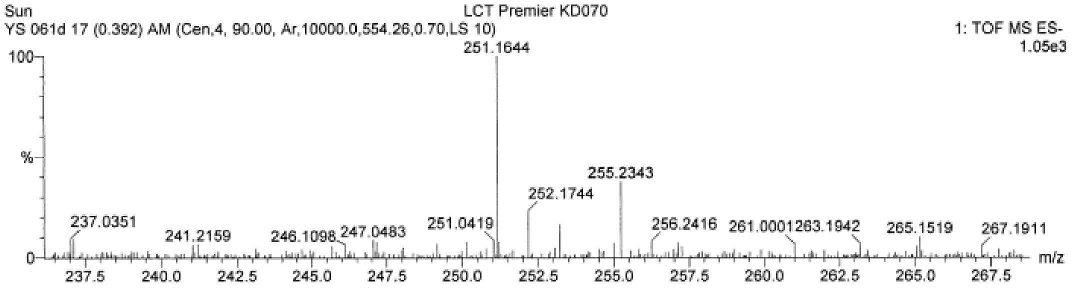

Minimum:				-1.5		
Maximum:		5.0	5.0	50.0		
Mass	Calc. Mass	mDa	PPM	DBE	i-FIT	Formula
251.1644	251.1647	-0.3	-1.2	4.5	77.0	C15 H23 O3

Figure S117 HRMS data for **21**; *m/z* (M- H)$^-$ calc. mass is 251.1647, 251.1644 was found

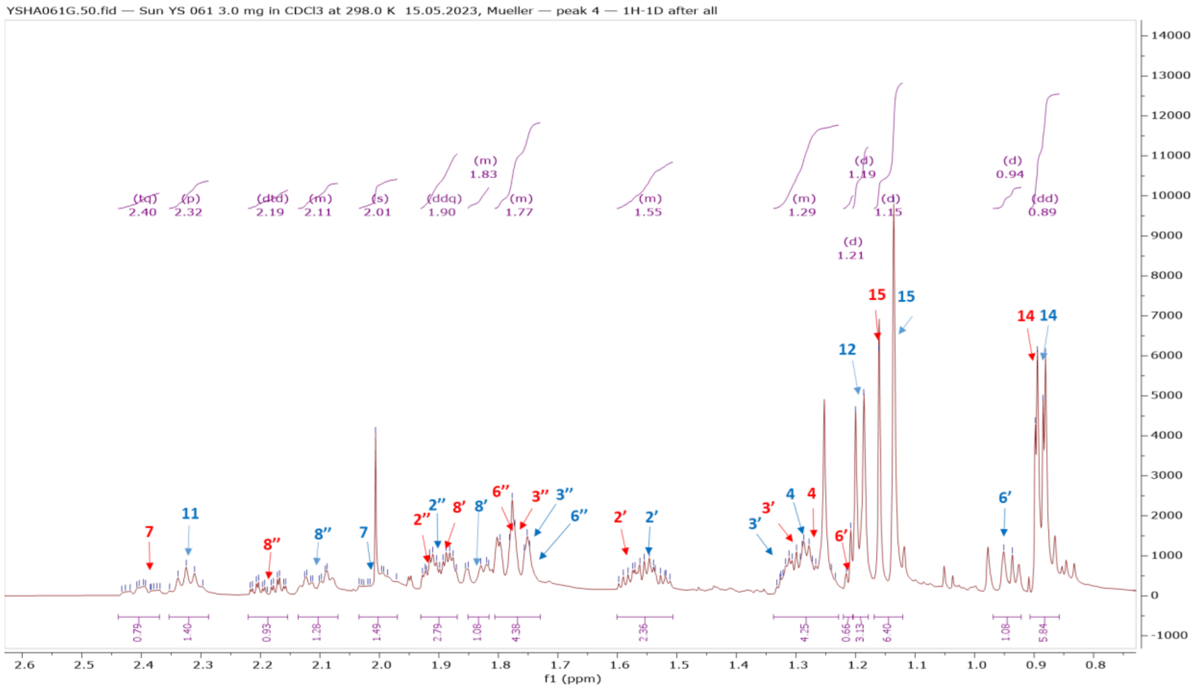

Figure S118 ^{1}H-NMR of **20** and **21** mixture recorded at 500 MHz in $CDCl_3$. The red numbers and arows represent the proton positions of **20**; The blue numbers and arrows represent the proton positions of **21.** The shifts labels are on the top of the integral curves; the type of the peaks are in the brackets (0.8 ppm – 2.6 ppm).

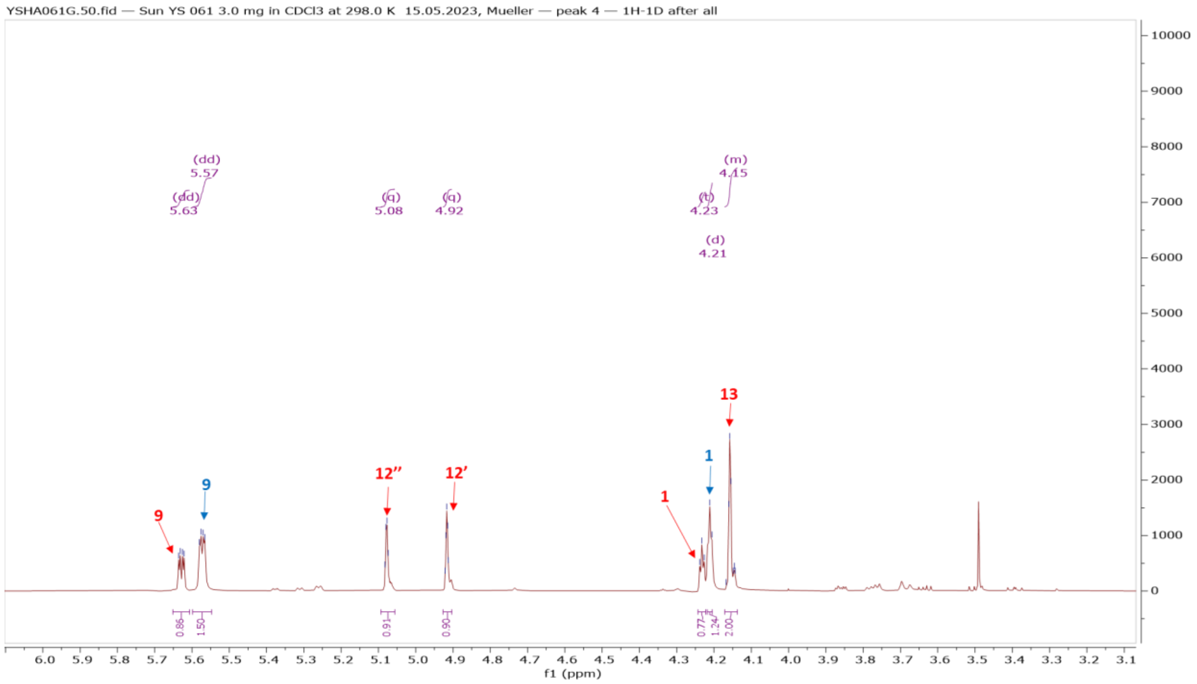

Figure S119 ^{1}H-NMR of **20** and **21** mixture recorded at 500 MHz in $CDCl_3$. The red numbers and arows represent the proton positions of **20**; The blue numbers and arrows represent the proton positions of **21.** The shifts labels are on the top of the integral curves; the type of the peaks are in the brackets (3.1 ppm – 6.0 ppm).

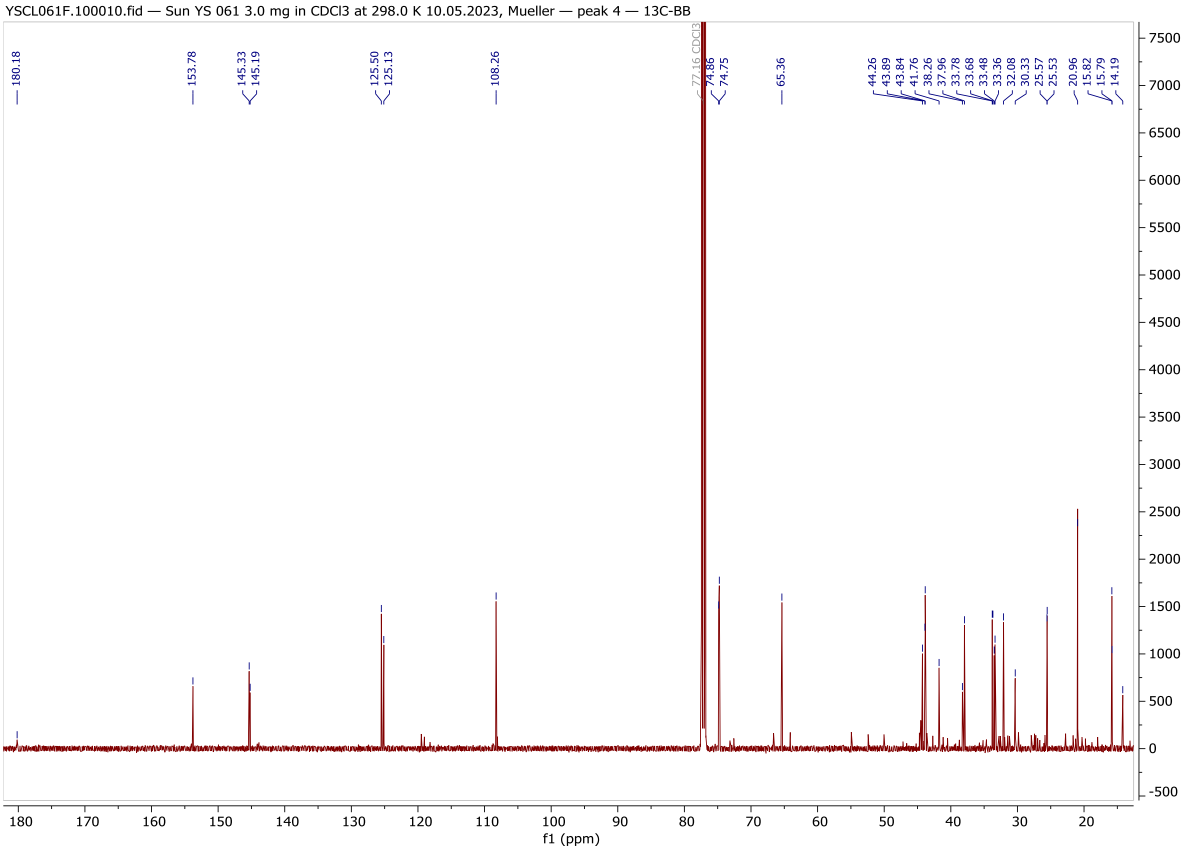

Figure S120 ^{13}C-NMR of **20** and **21** mixture recorded at 125 MHz in $CDCl_3$

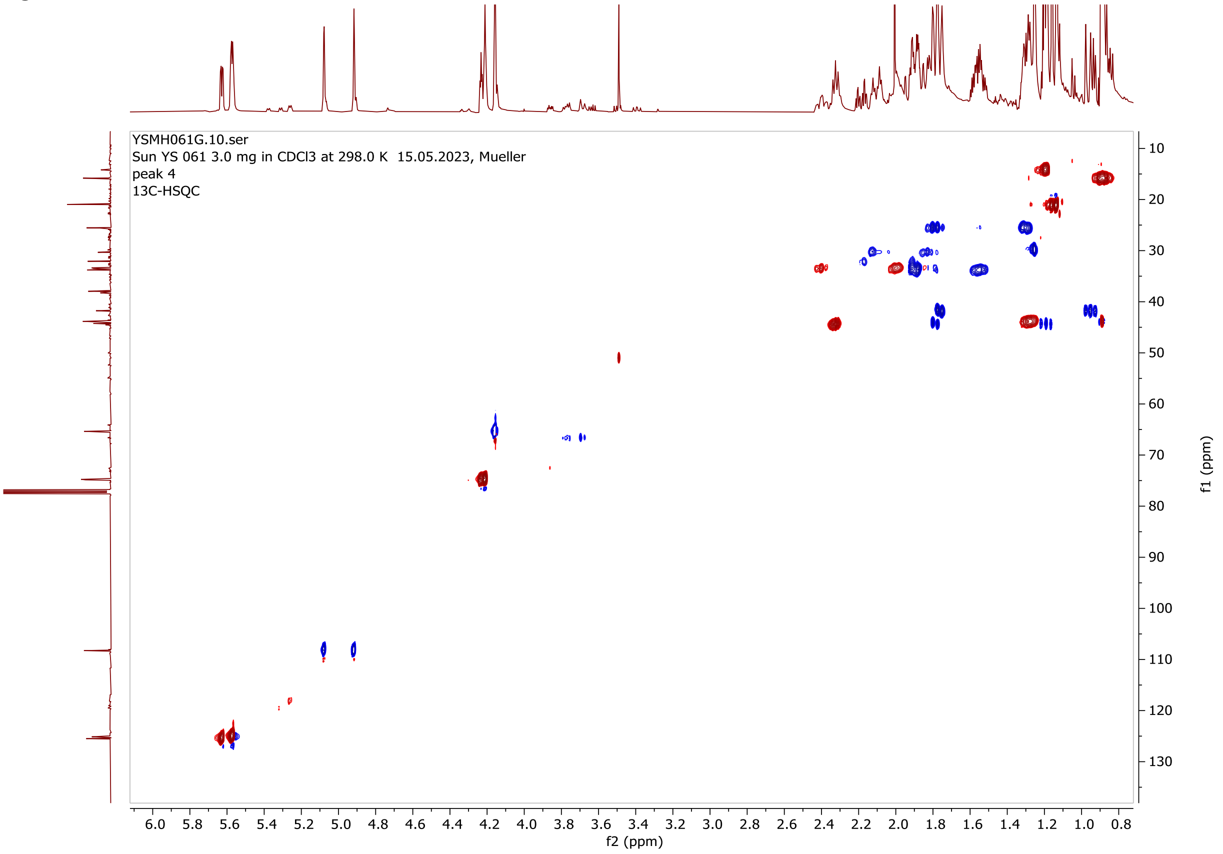

Figure S121 HSQC-spectrum of **20** and **21** mixture recorded at 500, 125 MHz in $CDCl_3$

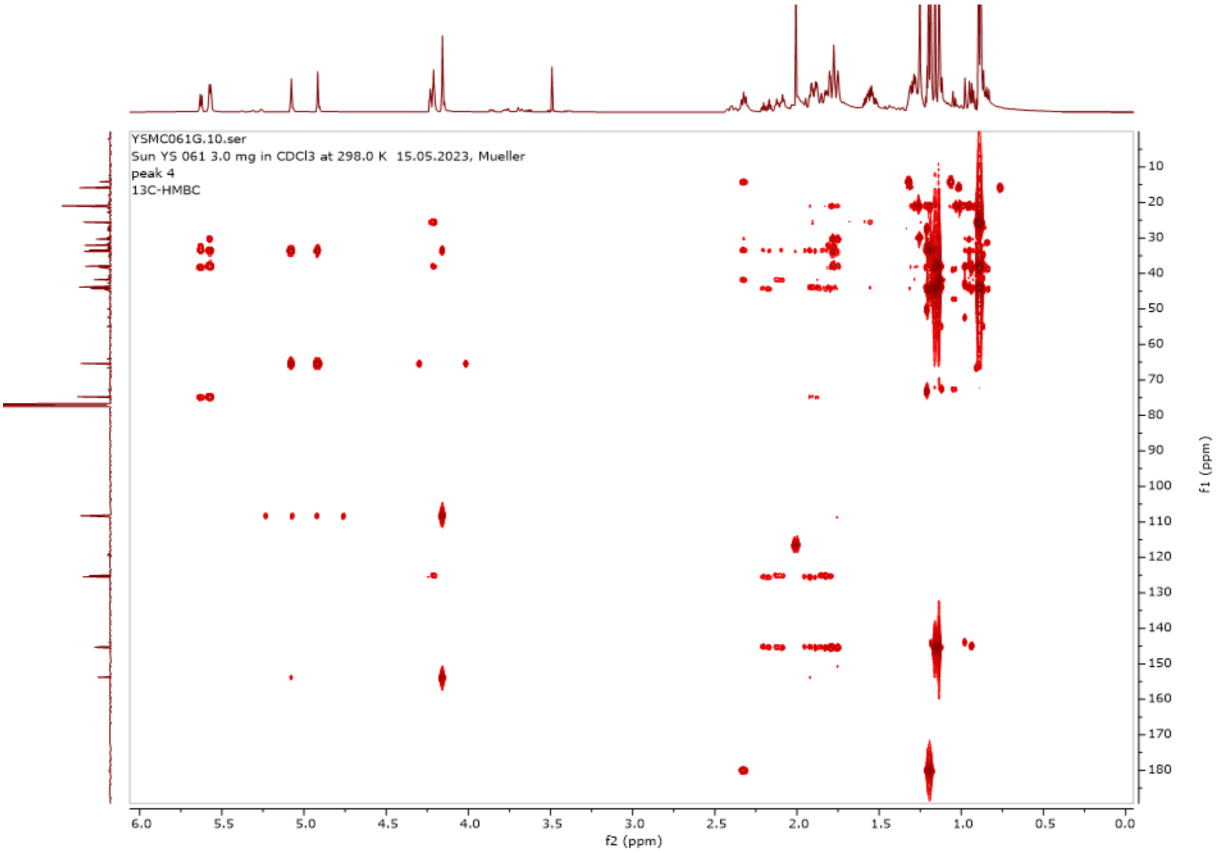

Figure S122 HMBC-spectrum of **20** and **21** mixture recorded at 500, 125 MHz in $CDCl_3$

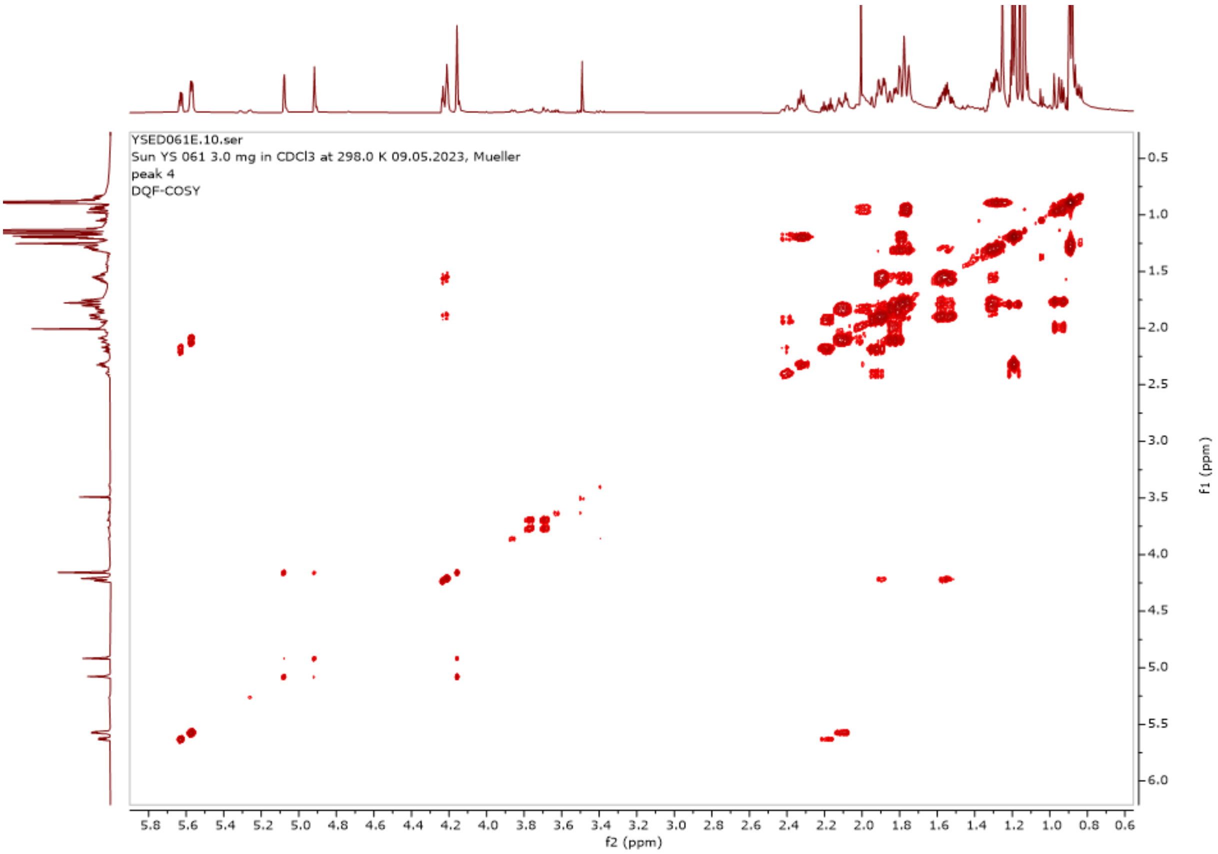

Figure S123 ^{1}H, ^{1}H-COSY-spectrum of **20** and **21** mixture recorded at 500 MHz in $CDCl_3$

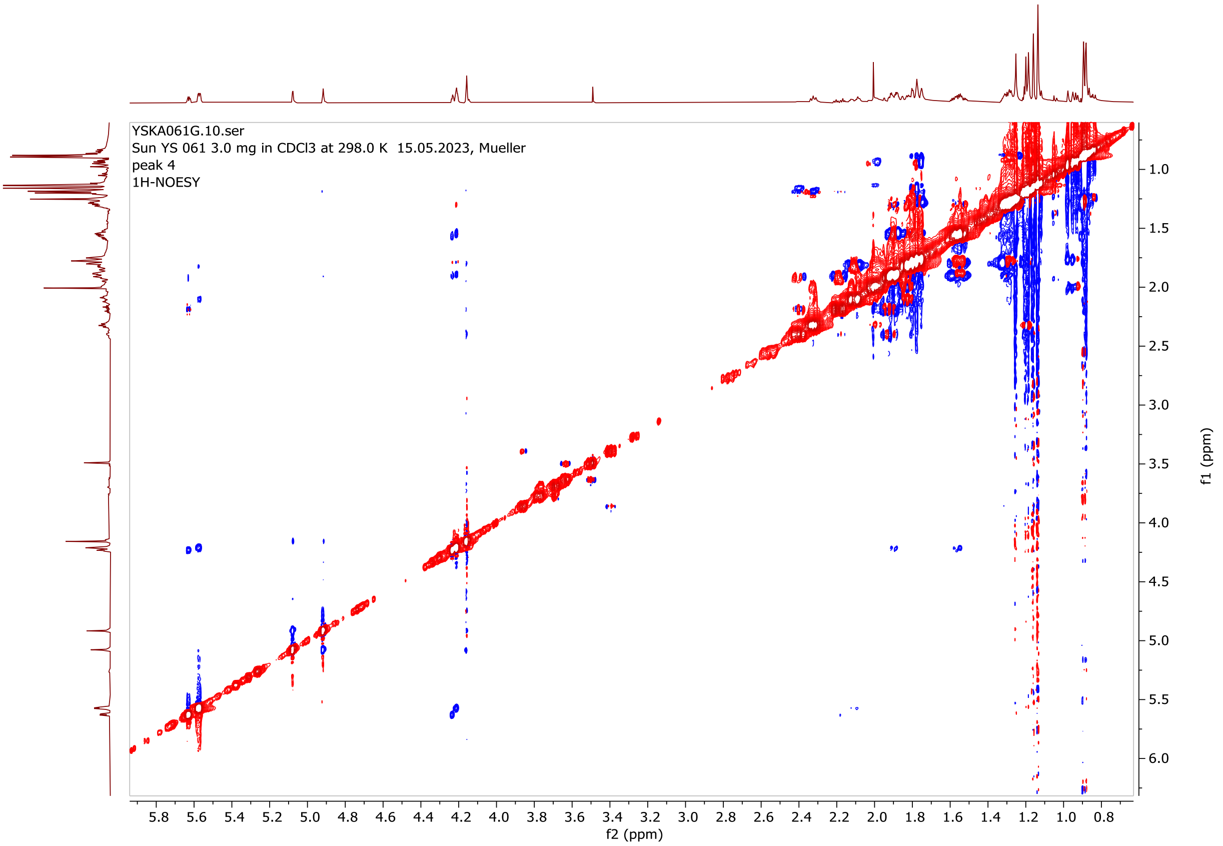

Figure S124 NOESY-spectrum of **20** and **21** mixture recorded at 500 MHz in $CDCl_3$

Compound 22

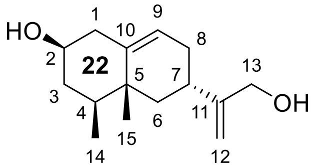

Chemical Formula: $C_{15}H_{24}O_2$
Exact Mass: 236.1776

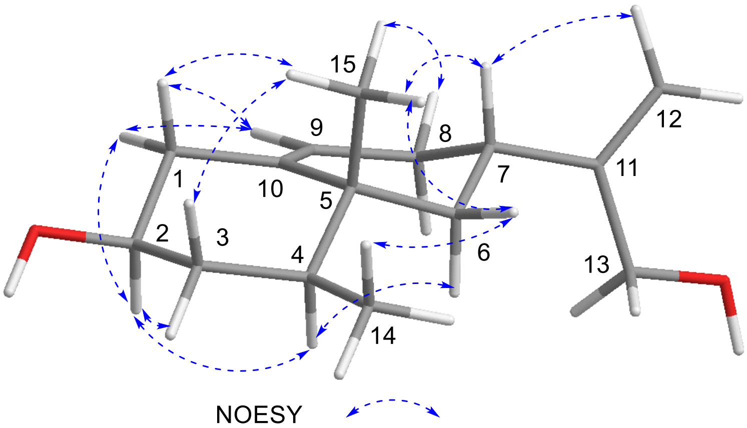

Compound 22				
**Pos.**	***δ***			

**Pos.**	δ_C / ppm	δ_H / ppm (*J*/Hz)	^{1}H-^{1}H COSY	HMBC (H-C)
**1**	42.1	2.11, 1H, m;<br>2.34, 1H, m	1, 2<br>1, 2, 3	2, 3, 5, 9, 10<br>2, 3, 5, 9, 10
**2**	71.1	3.57, 1H, dddd (11.0, 11.0, 4.8, 4.8)	1, 3	
**3**	40.3	1.37, 1H, m<br>1.73, 1H, dddd (11.7, 4.4, 2.4, 2.4)	3, 2, 4<br>3, 2, 4, 1	2, 4<br>1, 2, 4, 5
**4**	41.4	1.33, 1H, m	3, 14	
**5**	38.0			
**6**	43.3	1.16, 1H, m;<br>1.82, 1H, ddd (12.7, 2.3, 2.3)	6, 7<br>6, 7, 8	4, 5, 7, 8, 15<br>5, 7, 8, 10, 15
**7**	33.9	2.32, 1H, m	6, 8, 12	6, 8, 11, 12
**8**	32.1	1.9, 1H, dddd (17.2, 11.7, 3.7, 2.3);<br>2.11, 1H, m	8, 7, 9, 6<br>8, 7, 9	7, 9, 10<br>9, 10
**9**	121.6	5.4, 1H, m	8	7
**10**	141.5			
**11**	153.8			
**12**	108.2	4.91, 1H, m;<br>5.07, 1H, m	12, 13<br>12, 13	7, 11, 13<br>7, 11, 13
**13**	65.4	4.15, 2H, dd (1.3, 1.3)	12	7, 11, 12
**14**	15.5	0.88, 3H, d (6.6)	4	3, 4, 5
**15**	18.2	0.98, 3H, s		4, 5, 6, 10

Table S26 Summarized NMR signals for ^{13}C, ^{1}H, ^{1}H-^{1}H COSY, HMBC for **22** recorded in $CDCl_3$

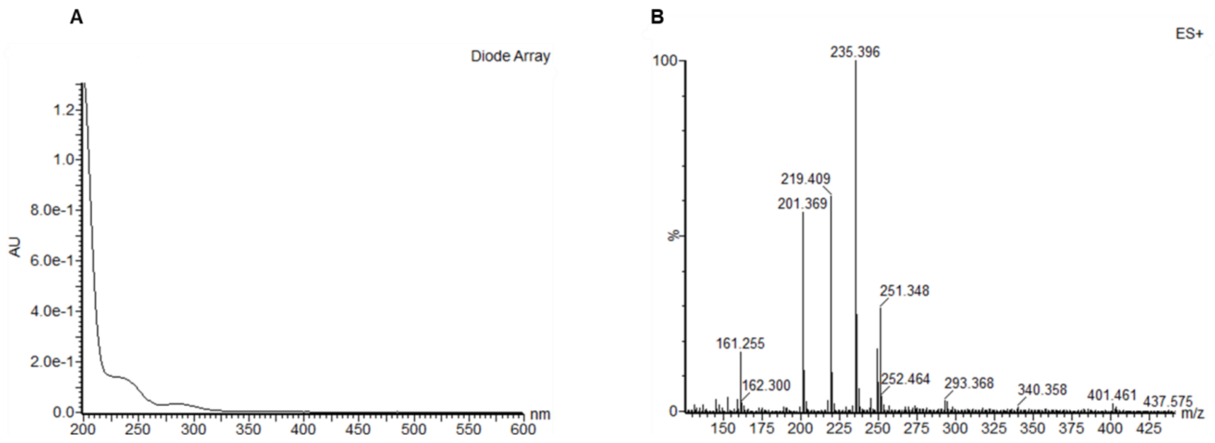

Figure S125 UV-absorption **(A)** and fragmentation pattern **(B)** of **22** in ES$^+$ TIC by LR-LCMS

Elemental Composition Report

Page 1

Single Mass Analysis

Tolerance = 40.0 PPM / DBE: min = -1.5, max = 50.0
Element prediction: Off

Monoisotopic Mass, Odd and Even Electron Ions
26 formula(e) evaluated with 1 results within limits (up to 50 closest results for each mass)
Elements Used:
C: 0-25 H: 0-35 O: 0-4 Br: 0-1

Sun
YS 058 2934 (12.229) AM (Cen,5, 70.00, Ar,5000.0,190.00,1.00); Cm (2930:2941)

TOF MS CI+
3.88e+002

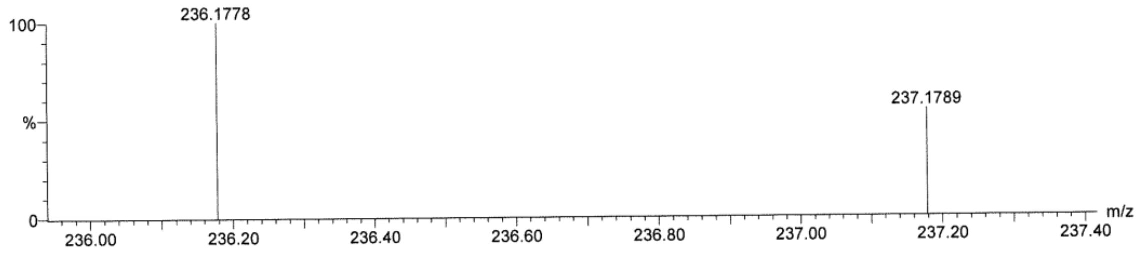

Minimum:				-1.5		
Maximum:		5.0	40.0	50.0		
Mass	Calc. Mass	mDa	PPM	DBE	i-FIT	Formula
236.1778	236.1776	0.2	0.8	4.0	2773078.8	C15 H24 O2

Figure S126 HRMS data for **22**; M calc. mass is 236.1776, 236.1778 was found by HR-GCMS

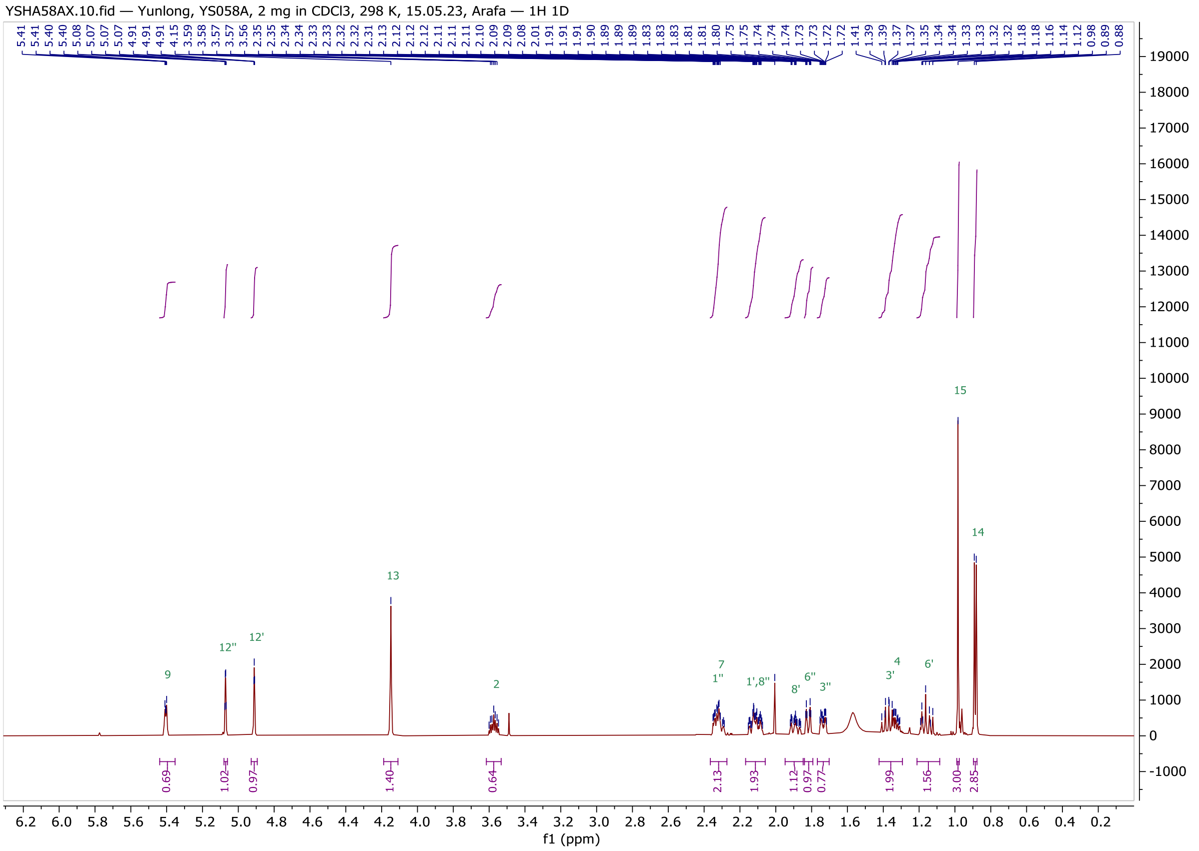

Figure S127 ^{1}H-NMR of **22** recorded at 600 MHz in $CDCl_3$

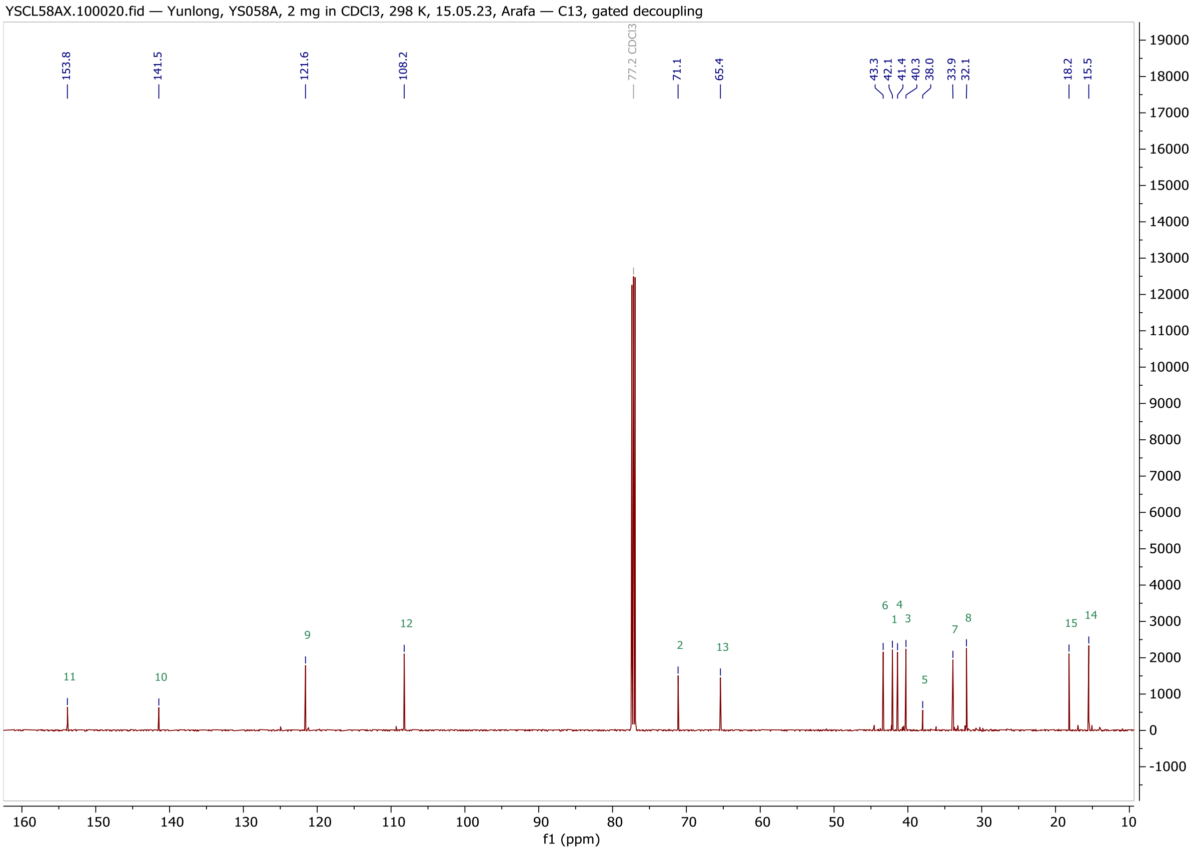

Figure S128 ^{13}C-NMR of **22** recorded at 150 MHz in $CDCl_3$

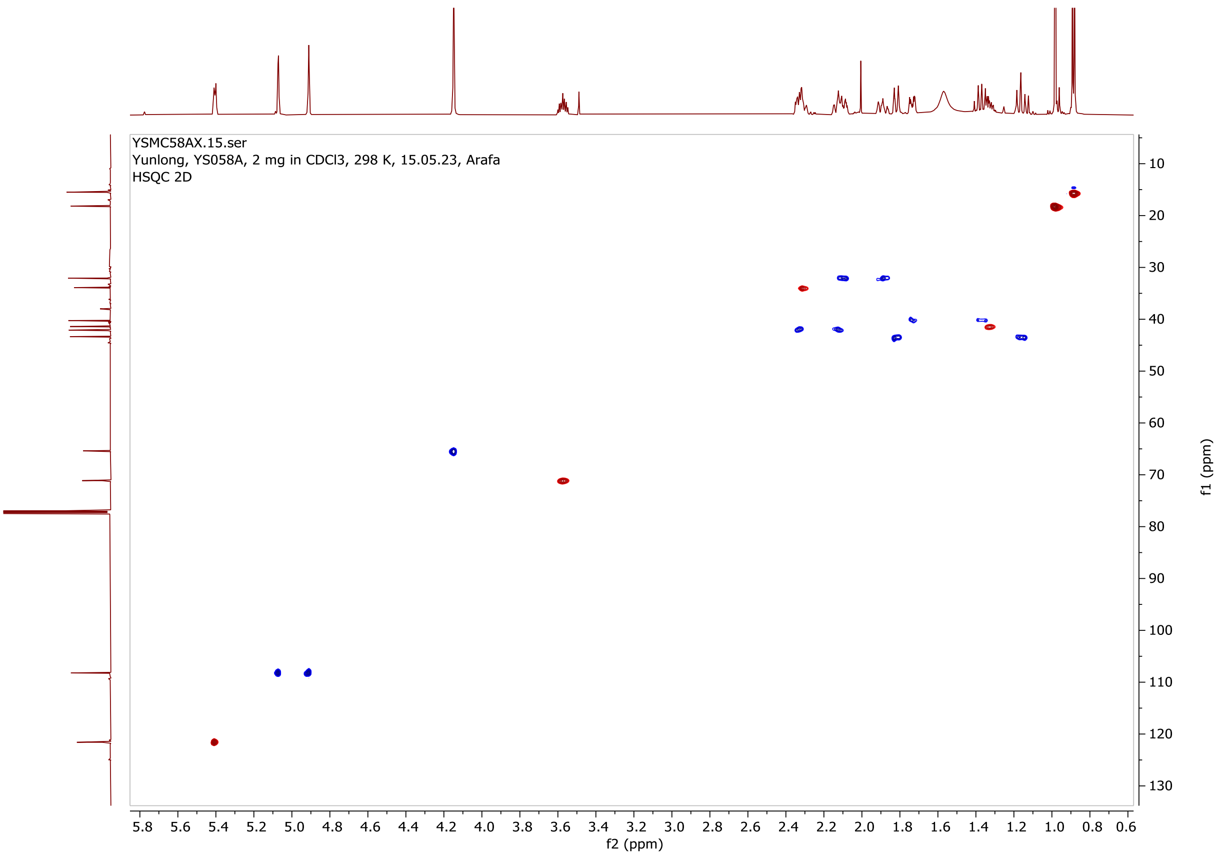

Figure S129 HSQC-spectrum of **22** recorded at 600, 150 MHz in $CDCl_3$

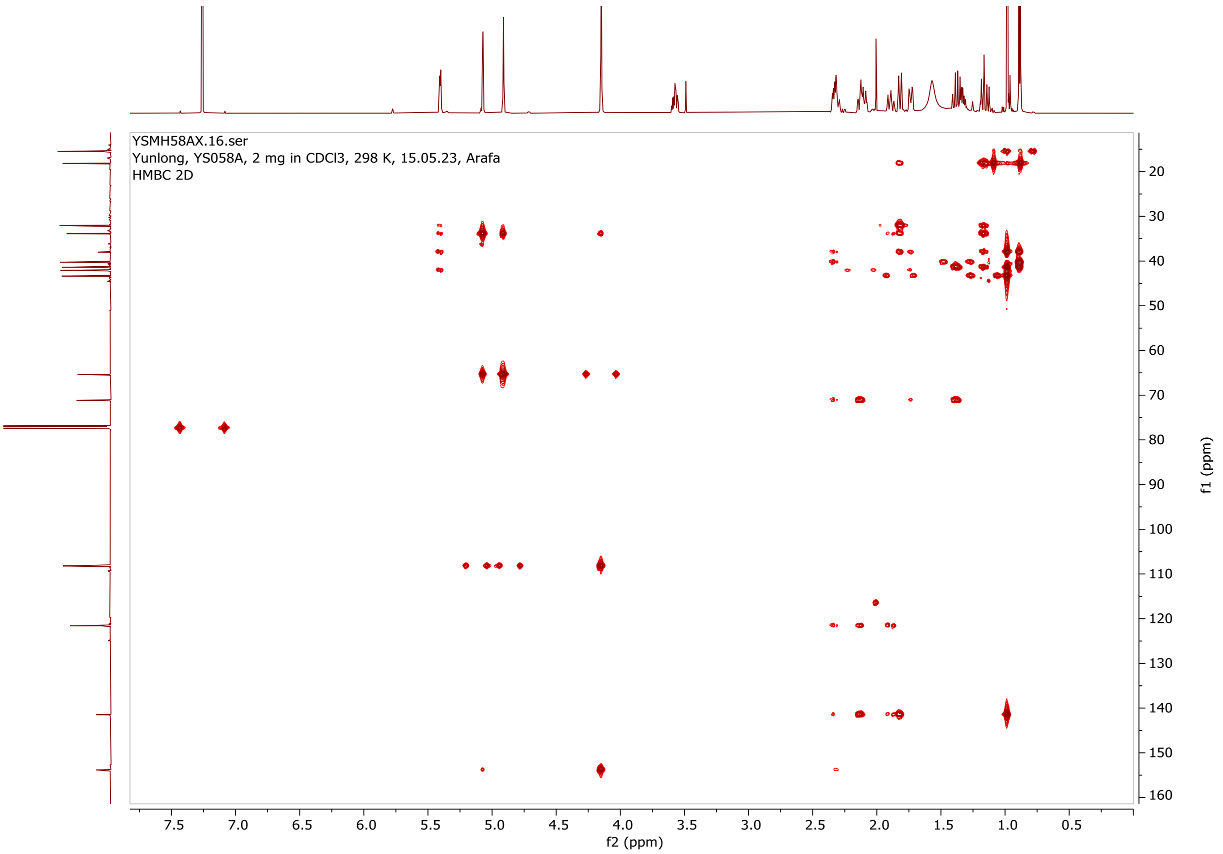

Figure S130 HMBC-spectrum of **22** recorded at 600, 150 MHz in $CDCl_3$

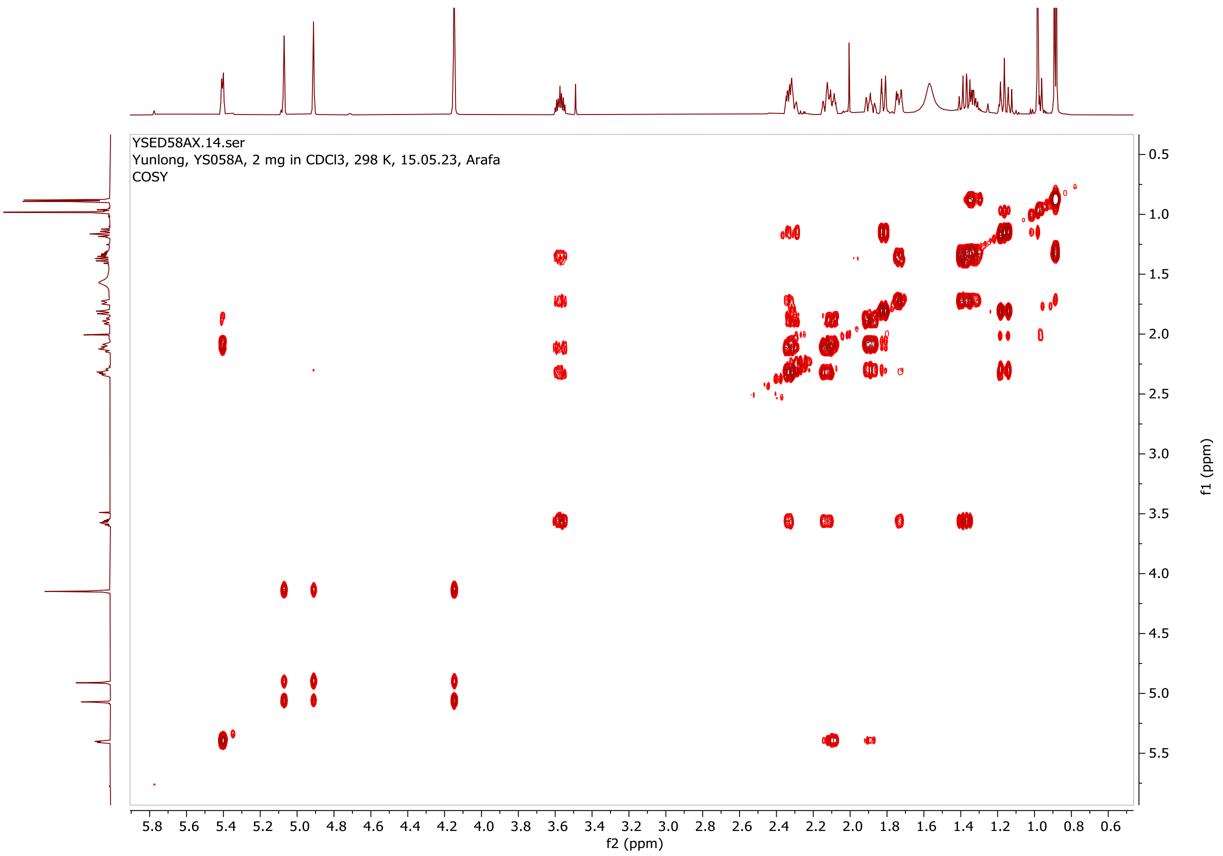

Figure S131 ^{1}H, ^{1}H-COSY-spectrum of **22** recorded at 600 MHz in $CDCl_3$

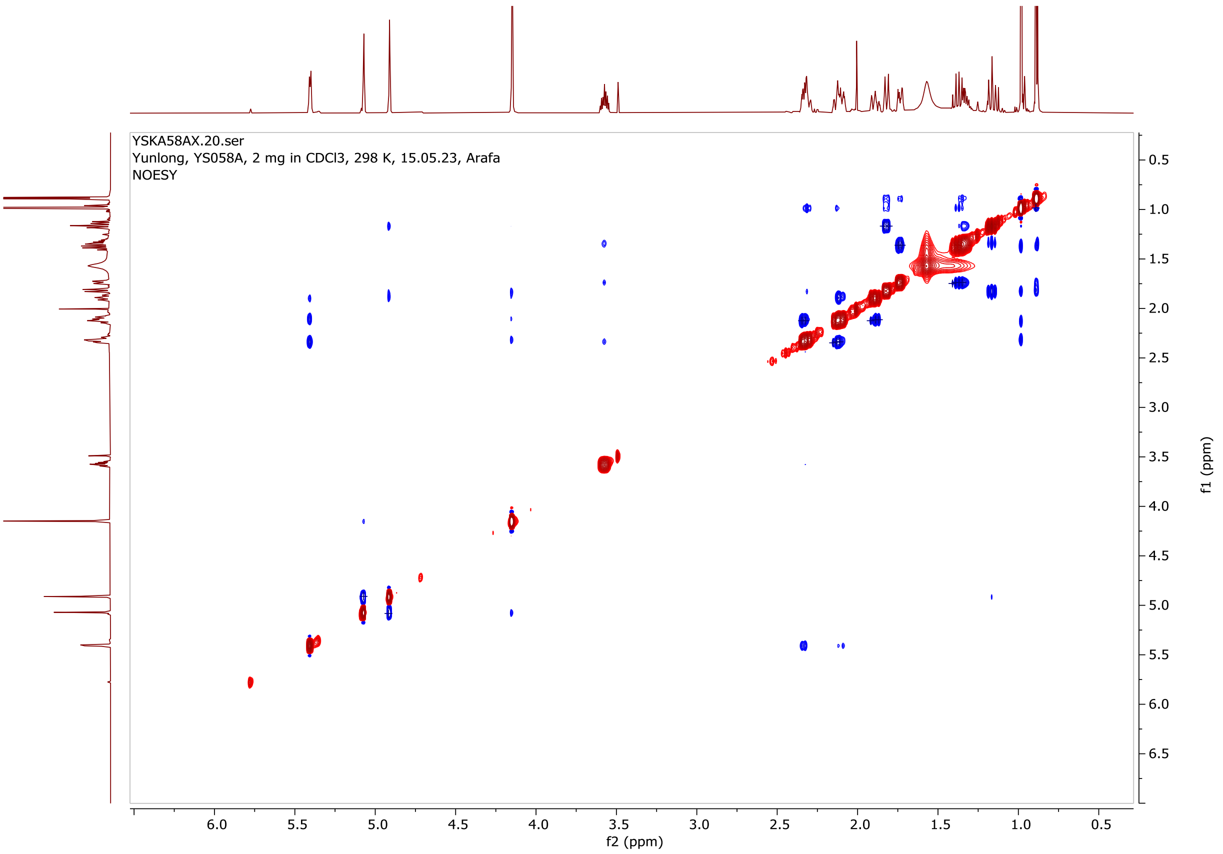

Figure S132 NOESY-spectrum of **22** recorded at 600 MHz in $CDCl_3$

Compound 23, 24

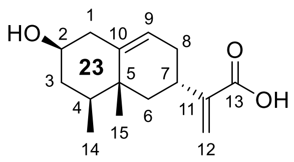

Chemical Formula: $C_{15}H_{22}O_3$
Exact Mass: 250.1569

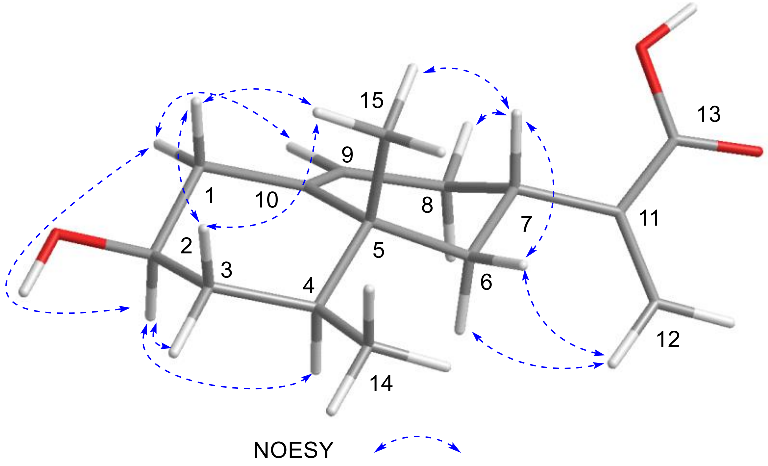

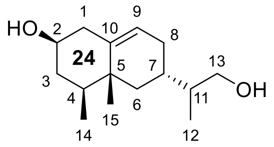

Chemical Formula: $C_{15}H_{26}O_2$
Exact Mass: 238.1933

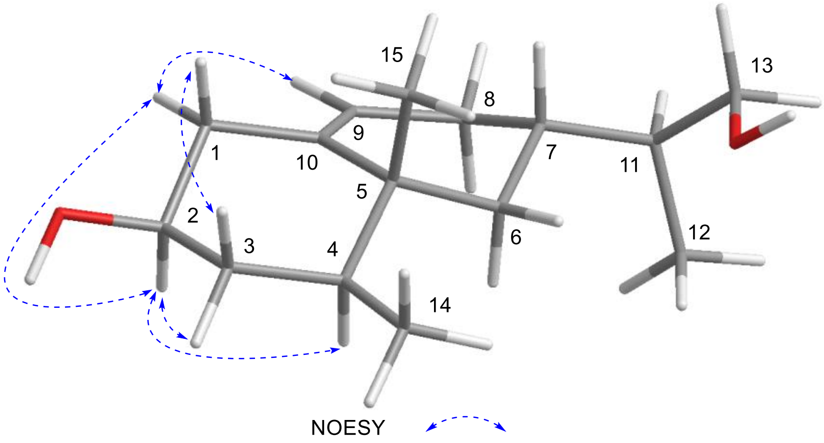

**Compound 23**				
**Pos.**	**δ_C / ppm**	**δ_H / ppm (*J*/Hz)**	**^1H-^1H COSY**	**HMBC (H-C)**
**1**	42.6	2.10, 1H, m;<br>2.28, 1H, ddd (12.9, 5.1, 2.3)	1, 2<br>1, 2, 3	2, 3, 9, 10<br>2, 3, 5, 9, 10
**2**	71.6	3.45, 1H, m	1, 3	1, 3
**3**	40.8	1.39, 1H, m;<br>1.70, 1H, m	3, 2, 4<br>3, 2, 4, 1	1, 2, 4, 10,<br>1, 2, 4, 5, 10
**4**	42.7	1.31, 1H, m	14, 3	3, 5, 6, 14, 15
**5**	39.3			
**6**	44.9	1.13, 1H, m;<br>1.84, 1H, m	6, 7<br>6, 7	4, 5, 7, 15<br>5, 7, 15
**7**	33.3	2.79, 1H, m	6, 8	5, 6, 8, 11, 12, 13
**8**	32.9	1.88, 1H, m;<br>2.14, 1H, m	8, 7<br>8, 7, 9	7, 9, 10, 11<br>7, 9, 10, 11
**9**	122.2	5.42, 1H, m	8	1, 5, 7
**10**	142.9			
**11**	147.5			
**12**	123.1	5.55, 1H, dd (1.3, 1.3);<br>6.17, 1H, d (1.2)	12, 7<br>12	7, 13<br>7, 11, 13
**13**	170.7			
**14**	15.8	0.89, 3H, d (6.4)	4	3, 4, 5
**15**	18.3	1.03, 3H, s		4, 5, 6, 10

Table S27 Summarized NMR signals for ^{13}C, ^1H, ^1H-^1H COSY, HMBC for **23** recorded in CD_3OD

**Compound 24**				
**Pos.**	**δ_C / ppm**	**δ_H / ppm (*J*/Hz)**	**^1H-^1H COSY**	**HMBC (H-C)**
**1**	42.7	2.12, 1H, m;<br>2.26, 1H, ddd (15.5, 5.5, 2.3)	1, 2<br>1, 2, 3	2, 3, 9, 10<br>2, 3, 5, 9, 10
**2**	71.7	3.45, 1H, m	1, 3	1, 3
**3**	40.9	1.39, 1H, m;<br>1.70, 1H, m	3, 2<br>3, 2, 1	2<br>2
**4**	42.8	1.31, 1H, m	14	3, 5, 6, 14, 15
**5**	38.8			
**6**	43.6	0.99, 1H, m;<br>1.71, 1H, m	6, 7<br>6, 7	5, 7, 15<br>5, 7, 15
**7**	32.8	1.75, 1H, m	6, 11	6, 8, 11
**8**	29.0	1.71, 1H, m;<br>1.90, 1H, m	8, 9<br>8, 9	7, 9, 10, 11<br>7, 9, 10, 11
**9**	122.7	5.38, 1H, m	8	1, 5
**10**	142.8			
**11**	41.5	1.51, 1H, m	7, 13	7, 8, 12, 13
**12**	13.3	0.88, 3H, d (6.4)	11	7, 13
**13**	66.4	3.38, 1H, dd (10.7, 6.9);<br>3.55, 1H, dd (10.7, 5.9)	13, 11<br>13, 11	7, 11, 12<br>7, 11, 12
**14**	15.8	0.89, 3H, d (6.4)	4	3, 4, 5
**15**	18.4	0.97, 3H, s		5, 6, 10

Table S28 Summarized NMR signals for ^{13}C, ^1H, ^1H-^1H COSY, HMBC for **24** recorded in CD_3OD

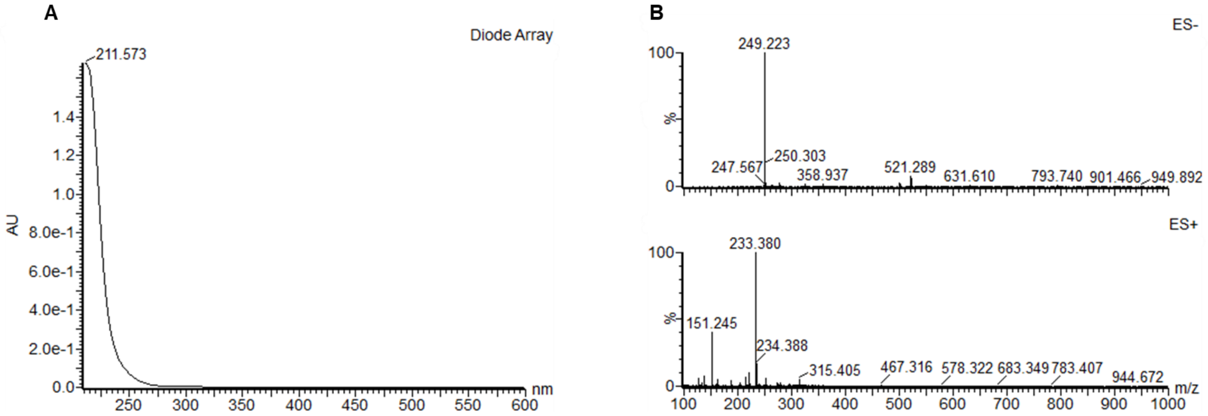

Figure S133 UV-absorption **(A)** and fragmentation pattern **(B)** of **23** in ES$^+$ TIC (bottom) and ES$^-$ TIC (top) by LR-LCMS

Elemental Composition Report

Page 1

Single Mass Analysis

Tolerance = 20.0 PPM / DBE: min = -1.5, max = 50.0
Element prediction: Off
Number of isotope peaks used for i-FIT = 3

Monoisotopic Mass, Even Electron Ions
27 formula(e) evaluated with 2 results within limits (all results (up to 1000) for each mass)
Elements Used:
C: 0-31 H: 0-45 O: 0-3 Na: 0-1

Sun QTof Premier HAB321
YS 059A, neg 635 (6.493) AM (Cen,4, 80.00, Ht,10000.0,554.26,0.70,LS 10) 1: TOF MS ES- 5.86e+001

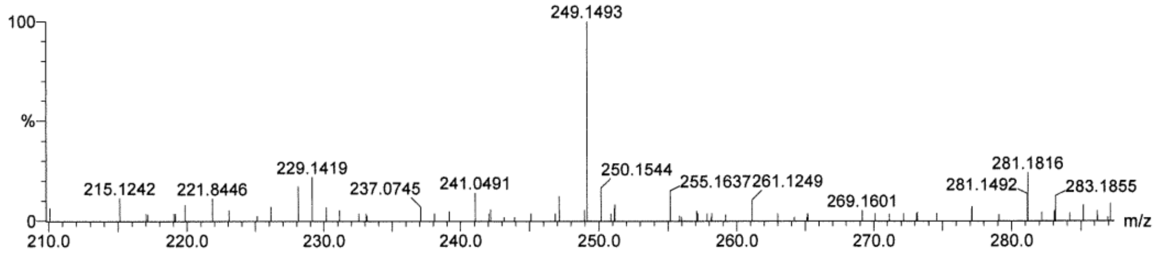

Minimum:				-1.5			
Maximum:		5.0	20.0	50.0			
Mass	Calc. Mass	mDa	PPM	DBE	i-FIT	i-FIT (Norm)	Formula
249.1493	249.1491	0.2	0.8	5.5	10.1	0.3	C15 H21 O3
	249.1467	2.6	10.4	2.5	11.1	1.3	C13 H22 O3 Na

Figure S134 HRMS data for **23**; *m/z* (M - H)$^-$ calc. mass is 249.1491, 249.1493 was found

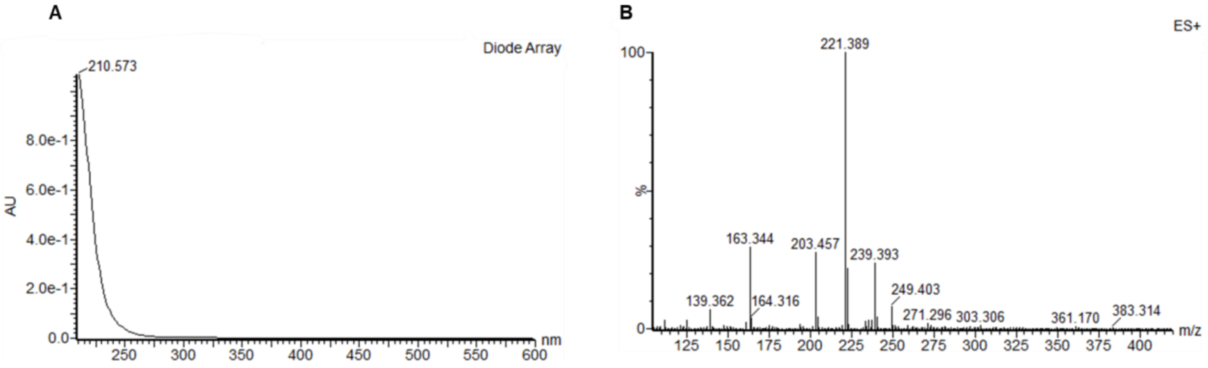

Figure S135 UV-absorption **(A)** and fragmentation pattern **(B)** of **24** in ES^+ TIC by LR-LCMS

Elemental Composition Report

Page 1

Single Mass Analysis

Tolerance = 40.0 PPM / DBE: min = -1.5, max = 50.0
Element prediction: Off

Monoisotopic Mass, Odd and Even Electron Ions
27 formula(e) evaluated with 1 results within limits (up to 50 closest results for each mass)
Elements Used:
C: 0-25 H: 0-35 O: 0-4 Br: 0-1

Sun
YS 059B 2942 (12.262) AM (Cen,5, 70.00, Ar,5000.0,190.00,1.00); Cm (2940:2947)

TOF MS CI+
1.50e+002

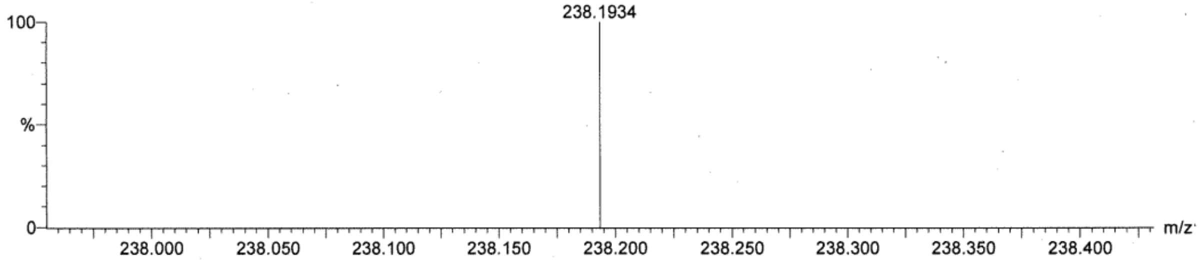

Minimum:				-1.5		
Maximum:		5.0	40.0	50.0		
Mass	Calc. Mass	mDa	PPM	DBE	i-FIT	Formula
238.1934	238.1933	0.1	0.4	3.0	5546081.5	C15 H26 O2

Figure S136 HRMS data for **24**; M calc. mass is 238.1933, 238.1934 was found by HR-GCMS

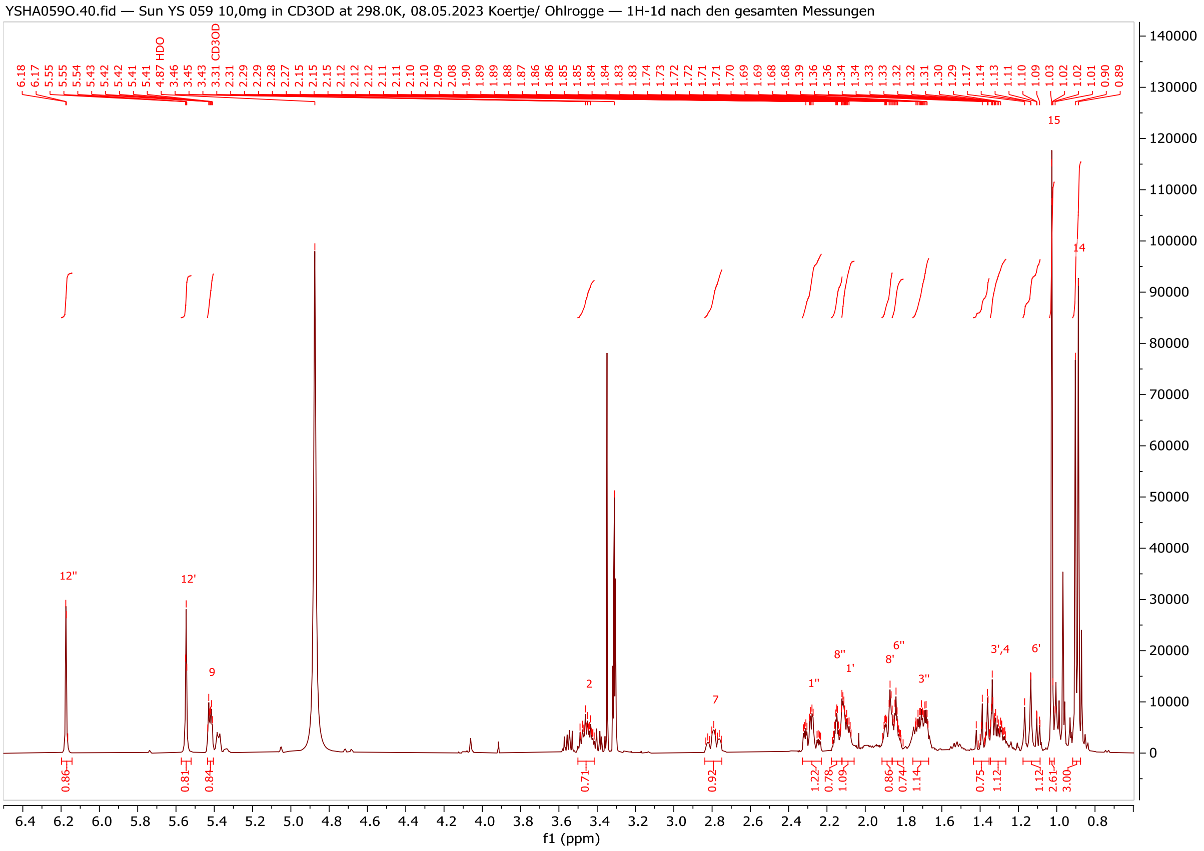

Figure S137 ^{1}H-NMR of **23** and **24** mixture recorded at 400 MHz in CD_3OD. The proton positions of **23** were labelled in red.

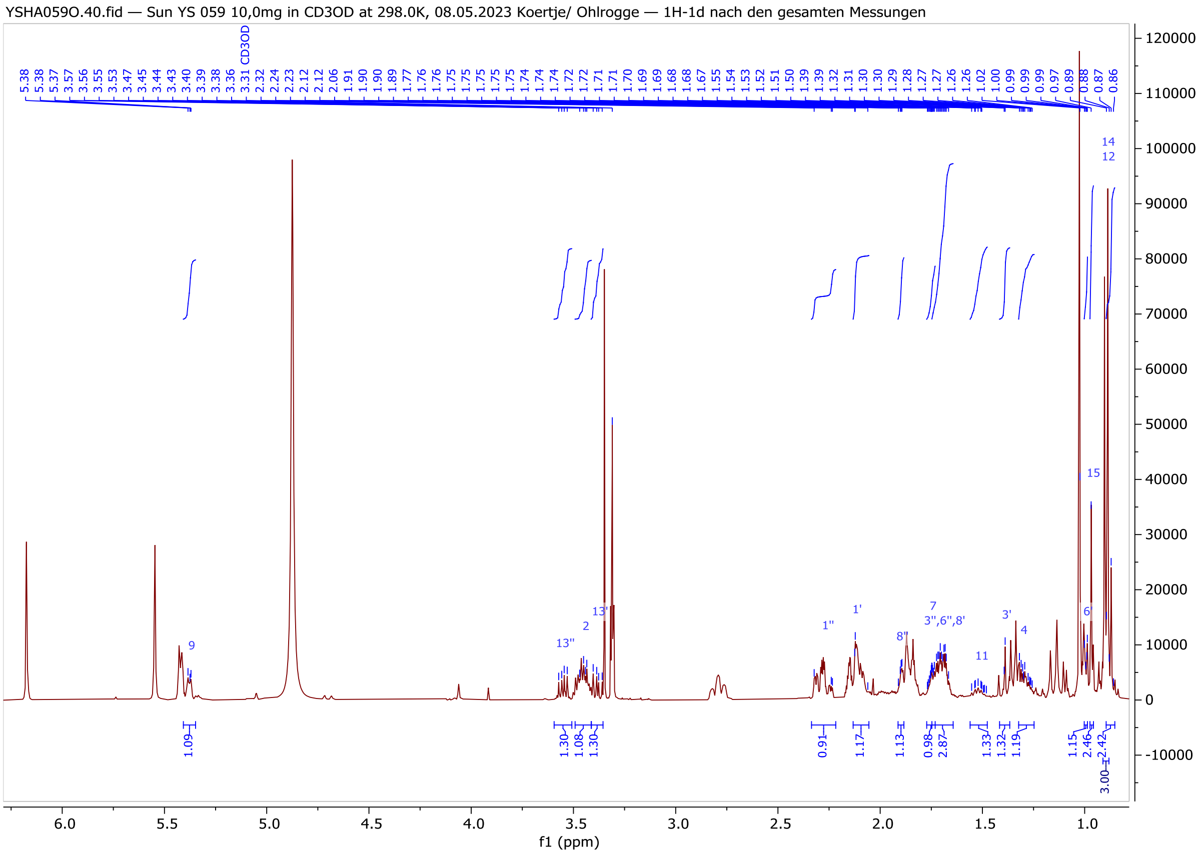

Figure S138 ^{1}H-NMR of **23** and **24** mixture recorded at 400 MHz in CD_3OD. The proton positions of **24** were labelled in blue.

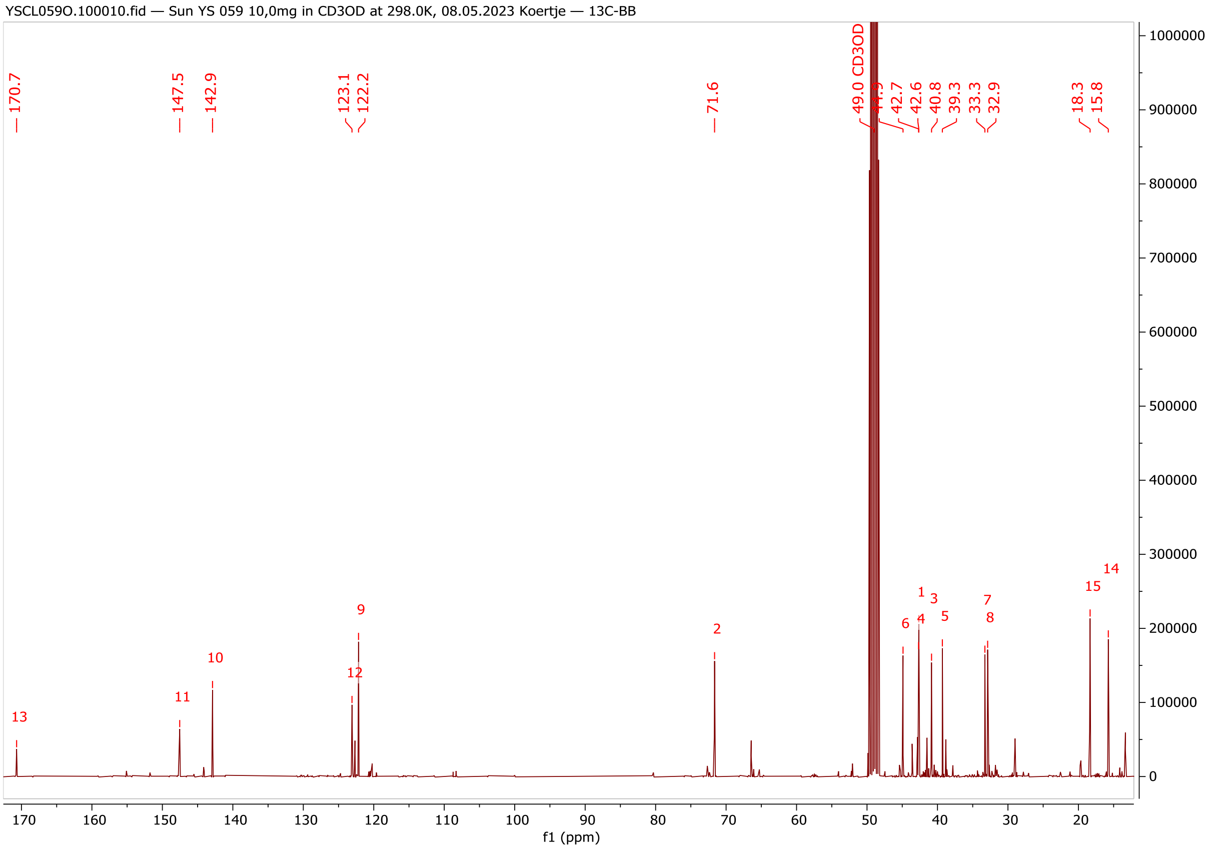

Figure S139 ^{13}C-NMR of **23** and **24** mixture recorded at 100 MHz in CD_3OD. The carbon positions of **23** were labelled in red.

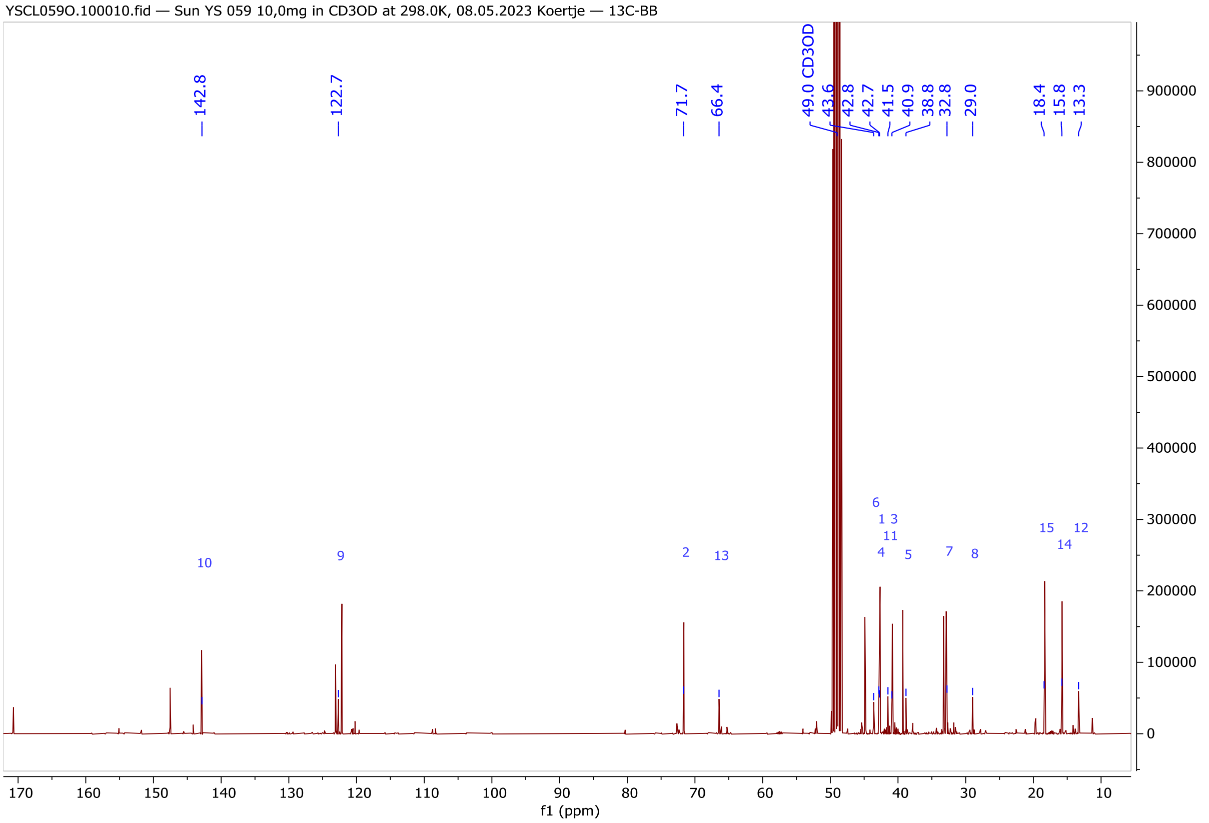

Figure S140 ^{13}C-NMR of **23** and **24** mixture recorded at 100 MHz in CD_3OD. The carbon positions of **24** were labelled in blue.

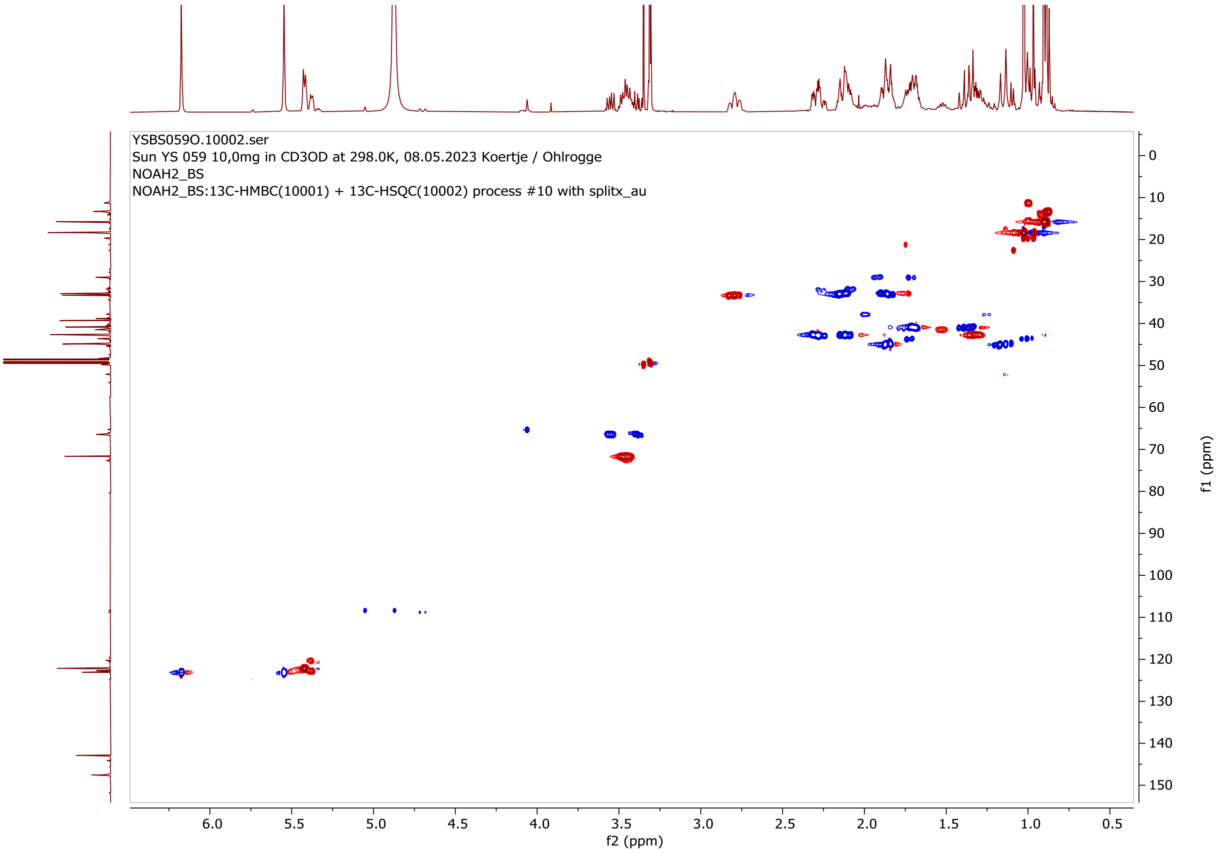

Figure S141 HSQC-spectrum of **23** and **24** mixture recorded at 400, 100 MHz in CD_3OD

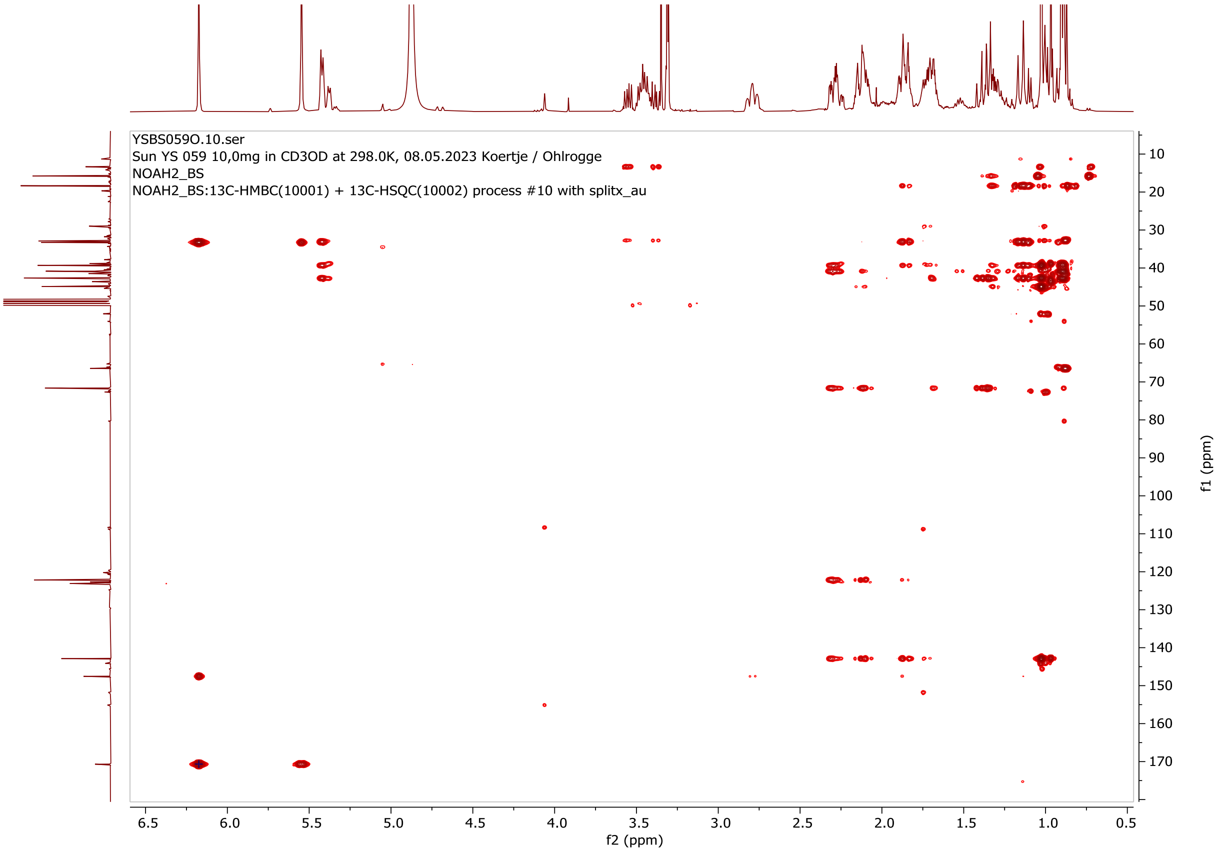

Figure S142 HMBC-spectrum of **23** and **24** mixture recorded at 400, 100 MHz in CD_3OD

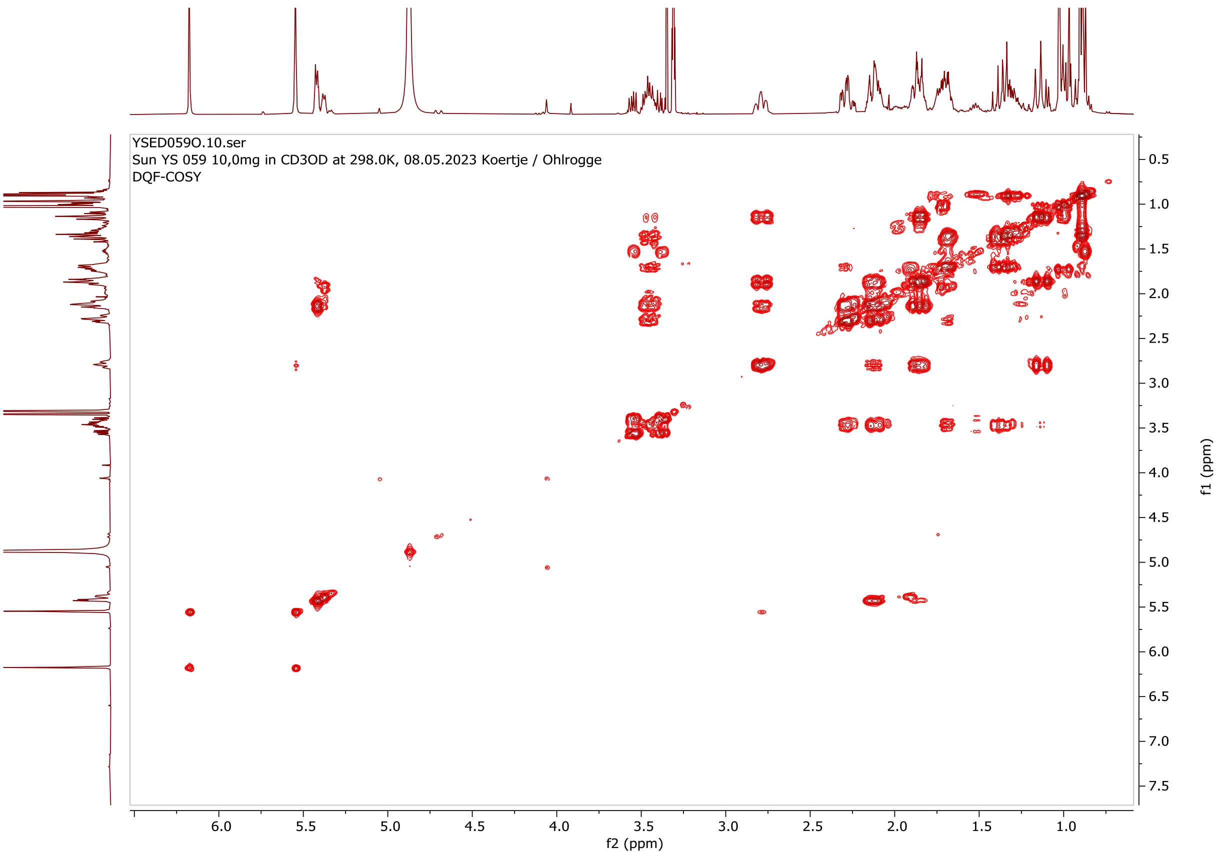

Figure S143 ^{1}H, ^{1}H-COSY-spectrum of **23** and **24** mixture recorded at 400 MHz in CD_3OD

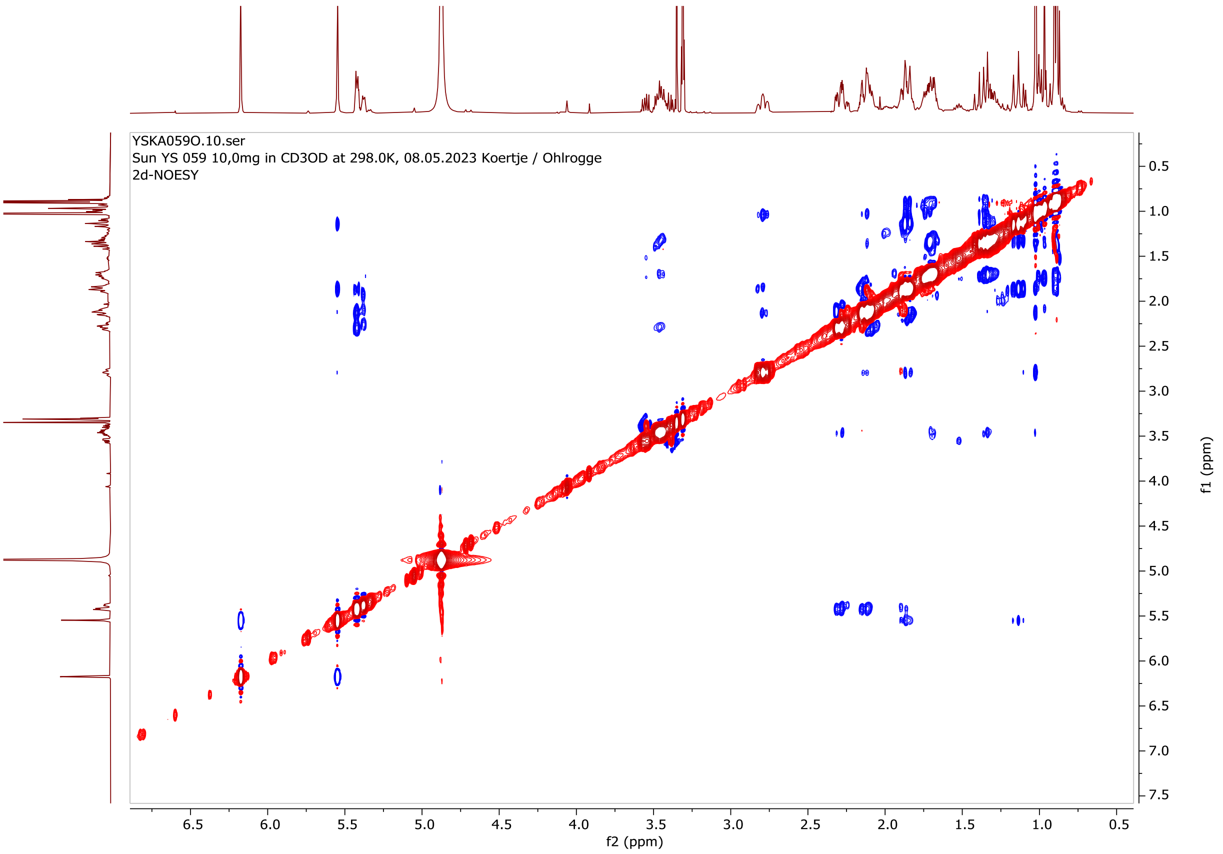

Figure S144 NOESY-spectrum of **23** and **24** mixture recorded at 400 MHz in CD_3OD

6. References

1 D. S. Tian, E. Kuhnert, J. Ouazzani, D. Wibberg, J. Kalinowski and R. J. Cox, *Chem Sci*, 2020, **11**, 12477–12484.

2 R. Nofiani, K. de Mattos-Shipley, K. E. Lebe, L. C. Han, Z. Iqbal, A. M. Bailey, C. L. Willis, T. J. Simpson and R. J. Cox, *Nature Communications 2018 9:1*, 2018, **9**, 1–11.

3 E. Kuhnert, F. Surup, V. Wiebach, S. Bernecker and M. Stadler, , DOI:10.1016/j.phytochem.2015.06.002.

4 Z. Cheng, J. Zhao, D. Liu, P. Proksch, Z. Zhao and W. Lin, *J Nat Prod*, 2016, **79**, 1035–1047.

5 Z. Y. Huang, Q. Y. Wu, C. X. Li, H. L. Yu and J. H. Xu, *J Agric Food Chem*, 2022, **2022**, 5868.

6 B. Felicetti and D. E. Cane, *J Am Chem Soc*, 2004, **126**, 7212–7221.
